# Small molecule/ML327 mediated transcriptional de-repression of E-cadherin and inhibition of epithelial-to-mesenchymal transition

**DOI:** 10.18632/oncotarget.4473

**Published:** 2015-06-10

**Authors:** Hanbing An, Sydney L. Stoops, Natasha G. Deane, Jing Zhu, Jinghuan Zi, Connie Weaver, Alex G. Waterson, Andries Zijlstra, Craig W. Lindsley, Robert Daniel Beauchamp

**Affiliations:** ^1^ Department of Surgery, Nashville, Tennessee, USA; ^2^ Department of Pharmacology, Nashville, Tennessee, USA; ^3^ Vanderbilt-Ingram Cancer Center, Nashville, Tennessee, USA; ^4^ Vanderbilt Institute of Chemical Biology, Nashville, Tennessee, USA; ^5^ Department of Pathology, Microbiology and Immunology, Nashville, Tennessee, USA; ^6^ Vanderbilt Center for Neuroscience Drug Discovery, Nashville, Tennessee, USA; ^7^ Department of Cancer Biology, Nashville, Tennessee, USA; ^8^ Department of Cell and Developmental Biology, Vanderbilt University Medical Center, Nashville, Tennessee, USA

**Keywords:** small molecule, E-cadherin, EMT

## Abstract

Transcriptional repression of E-cadherin is a hallmark of Epithelial-to-Mesenchymal Transition (EMT) and is associated with cancer cell invasion and metastasis. Understanding the mechanisms underlying E-cadherin repression during EMT may provide insights into the development of novel targeted therapeutics for cancer. Here, we report on the chemical probe, ML327, which de-represses E-cadherin transcription, partially reverses EMT, and inhibits cancer cell invasiveness and tumor cell migration *in vitro* and *in vivo*. Induction of E-cadherin mRNA expression by ML327 treatment does not require *de novo* protein synthesis. RNA sequencing analysis revealed that ML327 treatment significantly alters expression of over 2,500 genes within three hours in the presence of the translational inhibitor, cycloheximide. Network analysis reveals Hepatocyte Nuclear Factor 4-alpha (HNF4α) as the most significant upstream transcriptional regulator of multiple genes whose expressions were altered by ML327 treatment. Further, small interfering RNA-mediated depletion of HNF4α markedly attenuates the E-cadherin expression response to ML327. In summary, ML327 represents a valuable tool to understand mechanisms of EMT and may provide the basis for a novel targeted therapeutic strategy for carcinomas.

## INTRODUCTION

Cancer is a leading cause of death in the United States, and the vast majority of these cancers are of epithelial cell origin, or carcinomas [1]. Over 90% of cancer deaths related to solid malignancies are due to metastatic dissemination of cancer to secondary organs [2]. A hallmark of tumor malignancy and a requirement for metastasis is the acquired ability of cells to detach from the primary tumor mass and invade into surrounding stromal tissues. Epithelial-cadherin (E-cadherin, encoded by the *CDH1* gene) is a key component of the adherens junction complex and plays a pivotal role in epithelial tissue architecture and cell differentiation. Since most solid tumors are carcinomas that are derived from epithelial cells/tissues that predominantly express E-cadherin, the capacity of these cells to undergo neoplastic transformation and to metastasize is often associated with the loss of expression of this protein [3, 4].

Loss of E-cadherin expression can be due to mutational inactivation of the *CDH1* gene (as in familial gastric cancer syndrome) [5, 6], but more frequently the loss of expression is due to transcriptional inhibition or epigenetic silencing. EMT is a developmentally regulated process whereby epithelial cells undergo coordinated reprogramming of their gene expression and lose the epithelial characteristics of tight cell-cell adhesiveness and apical-basal polarity while gaining mesenchymal properties that include increased motility and capacity for invasion through the basement membrane [7, 8]. Several developmentally important transcriptional regulatory proteins, such as ZEB1, ZEB2, Snai1, Snai2/SLUG, TWIST 1, and E47/TCF3, induce EMT and are directly involved in repression of E-cadherin expression [9].

Because of the strong association of decreased E-cadherin expression and EMT, we undertook a phenotypic small molecule screen to identify compounds that could elevate E-cadherin in an E-cadherin-low, and metastatic, colon cancer cell line as we have previously reported [10]. A focused medicinal chemistry optimization effort generated over three hundred analogs of the parent phenotypic screening hit that increased E-cadherin expression. Secondary and tertiary screening assays demonstrated that the optimized compound, herein referred to as ML327, de-represses E-cadherin expression in human SW620inv colon and H520 lung cancer cells and inhibits cell invasion in culture with little to no cytotoxicity at effective concentrations. Importantly, ML327 reverses TGF-β induced EMT in cell culture and inhibits cancer cell motility *in vivo*. Furthermore, we find that ML327 alters expression of numerous genes in addition to *CDH1*, and functional gene enrichment analysis shows that HNF4α is significantly implicated as the upstream regulator for numerous differentially expressed genes. Thus, we have developed ML327 from a phenotypic screen, and determined multiple mechanistic features of its activity. We conclude that ML327 provides a new direction for probing the biological process of EMT and may yield therapeutic benefit by disrupting carcinoma progression through a unique mechanism of action.

## RESULTS

### Small-molecule ML327 inhibits tumor cell invasion, but not tumor cell viability

**Figure 1:**
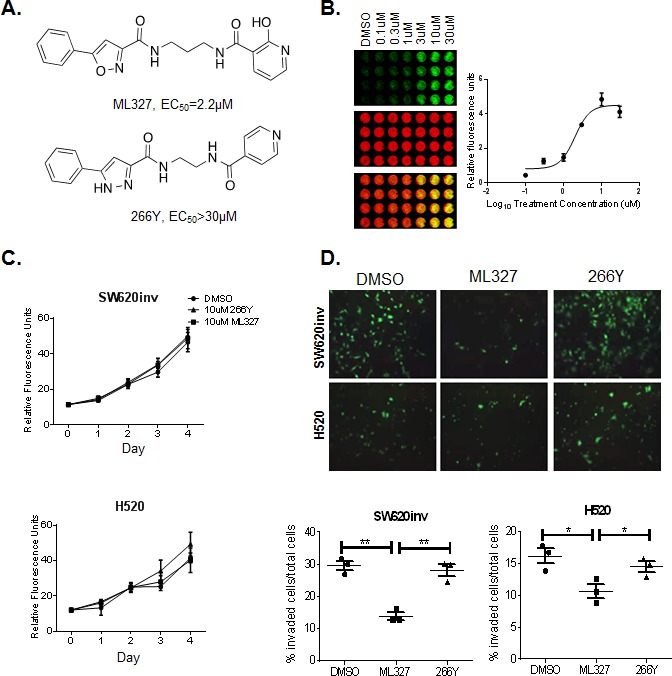
Treatment with ML327 reduces cancer cell invasive potential but has no effect on cell viability **A.** Chemical structures of ML327 and its inactive analogue, 266Y. Effective dose is listed for each compound. **B.** Representative in cell western (ICW) plate showing concentration-dependent changes in E-cadherin protein (green) relative to β-actin (red) following treatment with ML327 concentration as indicated. The graph shows mean values with standard error bars from 3 replicate plates. **C.** SW620inv and H520 cells were cultured in the presence of 10μM ML327, 10μM 266Y or DMSO for up to 4 days. Individual wells (*n* = 4 per group) were harvested for DNA content measures by fluorometry at 24 hour intervals. Mean fluorescence units (FU) is graphed with standard deviations for replicate wells in a representative experiment. The graphs are representative of at least three separate experiments with similar results. **D.** TOP: Images (200x magnification) of invading fluorescently labeled SW620inv and H520 cells cultured on Matrigel-coated transwells in the presence of 10μM ML327, 10μM 266Y or DMSO. BOTTOM: The proportion of stained cells that invaded through the transwell is graphed with a bar indicating the mean value and the whiskers indicating the standard deviation for 3 replicate wells in a representative experiment, statistical significance was calculated using unpaired *t* test, ** indicates *p* < 0.005, * indicates *p* < 0.05. The graphed data is representative of at least three separate experiments with similar results.

Our previous studies identified novel pharmacology for a series of isoxazole-based compounds in restoring E-cadherin protein to the surface of selected aggressive colon and lung cancer cells and inhibiting cancer cell invasion [10]. However, this first generation of compounds lacked the physiochemical properties to enable mechanistic studies. Additional optimization resulted in a new sub-series of compounds with improved potency and efficacy as well as more suitable physicochemical properties. The new analog sub-series is exemplified by *N*-(3-(2-hydroxynicotinamido) propyl)-5-phenylisoxazole-3-carboxamide, which we have named ML327, a Molecular Libraries Probe Center Network (MLPCN) probe (Figure 1A). The medicinal chemistry effort also uncovered structurally related molecules with minimal E-cadherin de-repression activity, such as the phenylpyrrole 266Y, (2-(5-phenyl-1*H*-pyrazole-3-carboxamido) ethyl) isonicotinamide trifluoroacetate), which serves as a negative control besides vehicle DMSO in our experiments described below.

To increase the signal to noise ratio in our experiments, we utilized SW620 colon cancer cells (herein referred to as “SW620inv”) that were selected for high invasive potential and low E-cadherin expression by passage through Matrigel, and all the selected clones shown E-cadherin restoration by Trichostatin A (TSA), a known histone deacetylase (HDAC) inhibitor, as parental SW620 (Supplementary Figure 1). Next, a quantitative in-cell Western (ICW) assay was used to determine an EC_50_ value of 2.22 μM +/− 0.25 for ML327 activity in SW620inv cells (Figure 1A- 1B), a 2-5 fold improvement over the first generation tool [10].

Since cell surface E-cadherin expression is associated with intact epithelial junctions and epithelial homeostasis, we examined the effect of ML327 on cell viability and cell invasive potential. To assess the effect of ML327 on cell viability, SW620inv and H520 cells were grown in the presence of 10μM ML327, 10μM 266Y or DMSO for up to four days and DNA content was measured at 24 hour intervals. We found no differences in the DNA content of these treatment groups, indicating that ML327 has no measurable effect on cell viability in either the SW620inv or H520 cells in 2D culture conditions (Figure 1C). To assess the effect of ML327 on cell invasion, SW620inv and H520 cells were grown in the presence of 10μM ML327, 10μM 266Y or DMSO for 48 hours, allowing for invasion through Matrigel covered chambers. We found that ML327 reduced SW620inv cell invasion through Matrigel by ∼60% and reduced H520 cell invasion by ∼30% in these *in vitro* assays (Figure 1D). These experiments demonstrated that ML327 significantly inhibits invasion of these cell lines with no effect on cell viability.

### ML327 partially reverses TGF-β-induced EMT

**Figure 2:**
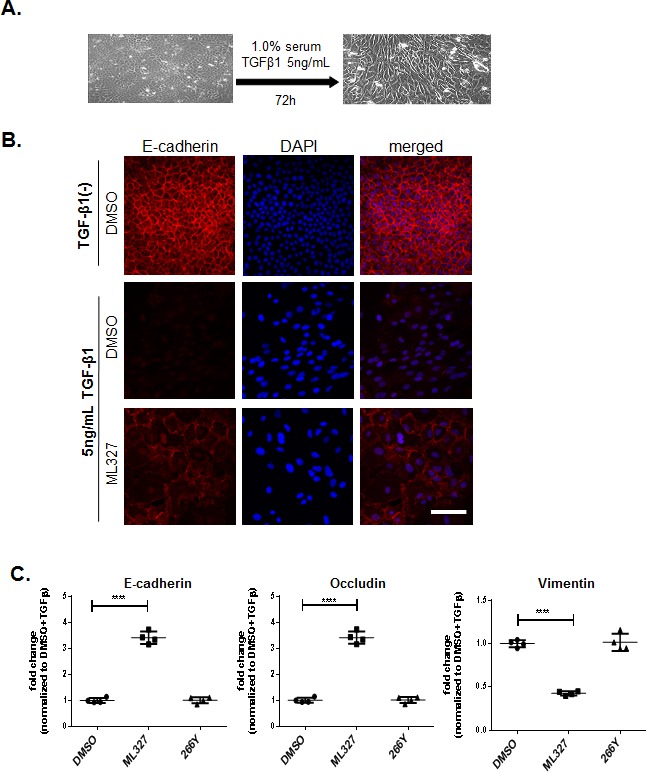
Treatment with ML327 partially reverses TGF-β-induced EMT **A.** Bright field microcopy images (200x magnification) showing the cell morphology after 72 hours TGF- β1 treatment. **B.** Immunofluorescence images showing E-cadherin (red) expression and localization in NMuMg cells following treatment without or with 5ng/mL TGF-β1 for 72 hours, then adding either DMSO, or 10 μM ML327 for an additional 48 hours. Nuclei (blue) are labeled with DAPI. Bar = 100 μM. **C.** Relative levels of E-cadherin, Occludin and Vimentin specific mRNA species in NMuMg cells following treatments shown in **B.**, the graph shows mean values with standard error bars from 4 replicate wells in a representative experiment. Statistical significance was calculated using unpaired *t* test **** indicates *p* < 0.0001. These experiments have been done at least three separate times with similar results.

To examine the effect of ML327 on EMT, we studied its activity in the classical model system of TGF-β induced EMT in NMuMG mouse mammary epithelial gland cells. TGF-β induced morphological changes in NMuMG cells consistent with EMT [11, 12], (Figure 2A). To determine whether ML327 can reverse EMT induced by TGF-β in NMuMG cells, we treated cells with or without TGF-β1 for 72 hours and then allowed the cells to continue to grow under the same TGF-β1 (+/−) treatment conditions in the presence or absence of ML327 for additional 48 hours. As a result of EMT, the cells adopt a spindlelike shape, and appeared to increase in size (Figure 2B), which is also consistent with a previous report [13]. We found that ML327 partially restored E-cadherin expression at the plasma membrane in NMuMG cells induced to undergo EMT by TGF-β1 treatment (Figure 2B). We also found that treatment with ML327 increased expression of both E-cadherin (*CDH1*) and Occludin (tight junction protein) mRNA levels and decreased expression of Vimentin mRNA in NMuMG TGF-β1 treated cells, relative to cells treated with DMSO or 266Y (Figure 2C). These results are consistent with the conclusion that ML327 reverses typical molecular features of EMT in this experimental system.

### ML327 inhibits tumor cell migration *in vivo*

**Figure 3:**
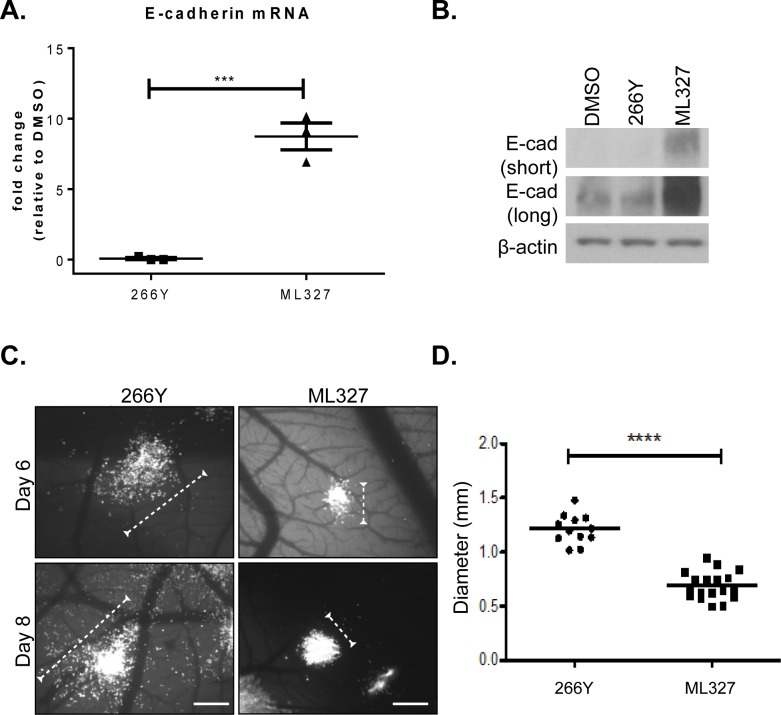
Treatment with ML327 inhibits tumor cell migration *in vivo* **A.** Relative E-cadherin mRNA expression in HEp3 cells following 6 hours treatment with either 266Y or ML327, the graph shows mean values with standard error bars from 3 replicate wells in a representative experiment, statistical significance was calculated using unpaired *t* test, *** indicates *p* < 0.001. The grafted data is representative of three separate experiments with similar results. **B.** Western blot showing E-cadherin (E-cad) protein expression (short and long exposure) in HEp3 cells following treatment with DMSO, 266Y or ML327 for 24 hours. **C.** Avian embryos were treated with compounds 266Y or ML327 24 hours after intravenous injection of GFP-expressing HEp3 cells. Images were acquired using a fluorescent stereoscope at 50X magnification. Representative images are shown, scale bar = 500 μm. **D.** Graph shows quantification of colony diameter at 6 days post injection. The data represent a total of 30 embryos from two independent experiments: 12 treated with 266Y and 18 treated with ML327. Each data point on the scatterplot represents the geometric mean diameter of 6-10 colonies analyzed per embryo, statistical significance was calculated using Mann Whitney U test, **** indicates *p* < 0.0001. The graphed data is representative of two separate experiments with similar results.

To determine the effect of ML327 on migratory behavior of cancer cells in a more relevant physiological system we used a well characterized chick chorioallantoic membrane (CAM) assay. HEp3 (human epidermoid carcinoma) cells were selected as an E-cadherin-low GFP-labeled carcinoma cell line that has been characterized for invasive and metastatic behavior when injected intravascularly into chicken embryos [14]. We determined that ML327 induced an 8-fold increase in E-cadherin mRNA by 6 hours after treatment in the HEp3 cells (Figure 3A) and a significant increase in E-cadherin protein expression by 24 hours after ML327 treatment (Figure 3B) as compared with DMSO or 266Y control treatments that did not alter E-cadherin expression.

In the CAM experimental model system, 100,000 GFP-labeled HEp3 cells were injected intravascularly into 10 day old chicken embryos and allowed to migrate and form tumor colonies as previously described [14]. Embryos were treated with ML327 or 266Y at 24 hours after tumor cell injections and colony formation was assessed by fluorescence imaging at 6 and 8 days post-injection. We observed measurable and reproducible inhibition of tumor cell motility at both time points, as reflected in the limited number of single cells dispersing from the colonies when the embryos are treated with ML327 (Figure 3C-3D, Supplementary Figure 2A), suggesting that ML327 has the ability to impair cancer cell migration *in vivo*.

### Small-molecule ML327 modulates E-cadherin mRNA levels independently of de novo protein synthesis

**Figure 4:**
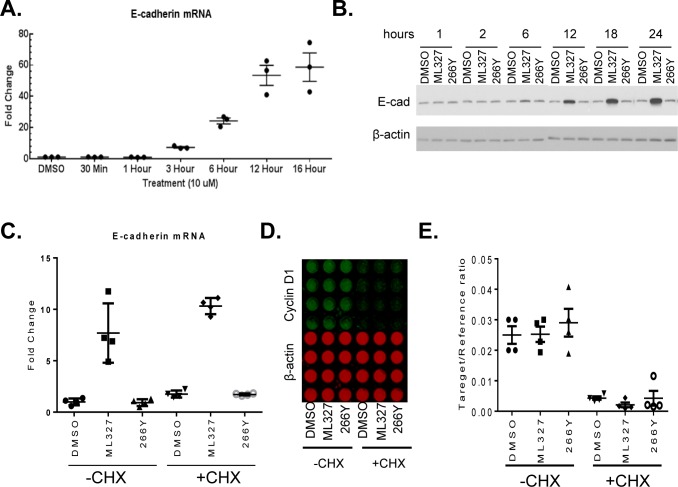
ML327 increases E-cadherin expression in SW620inv colon cancer cells **A.** Quantitative PCR results for E-cadherin specific mRNA in SW620inv cells following treatment with ML327 at the indicated time periods (data points represent results from3 biological replicates). Fold change relative to DMSO treatment is determined by the formula log2^−ΔΔCp^. **B.** Western blot shows time dependent changes in SW620inv E-cadherin (E-cad) protein expression relative to β-actin following treatment with DMSO, 10 μM ML327, or 266Y. **C.** Quantitative PCR analysis of E-cadherin mRNA in SW620inv cells following 1 hour with or without CHX pretreatment, then adding DMSO, ML327, or 266Y for another 6 hours. Fold change in E-cadherin mRNA, relative to DMSO without CHX is calculated by the formula log2^−ΔΔCp^. Graphed data represent four independent experiments. **D.** ICW plate matched to **C.** showing quantification of cyclin D1 protein (green) and β-actin (red). **E.**The graph shows quantification of relative cyclin D1 signal matched to **D.**.

We previously reported that treatment of SW620 cells with first generation molecules resulted in significant induction of E-cadherin mRNA by 24 hours after exposure [10]. Here, we extended these studies to determine the effect of ML327 at the transcriptional level. We found that ML327 induced significant increases in E-cadherin mRNA levels by as early as 3 hours after treatment in the SW620inv cells (Figure 4A). By 6 hours post-treatment, ML327 increased E-cadherin mRNA expression 25-fold in SW620inv cells and 12-fold in H520 cells (Supplementary Figure3A). We also determined the kinetics of altered E-cadherin expression by ML327 in SW620inv cells by Western blot. By 6 hours post-treatment with ML327, E-cadherin protein levels in SW260inv cells were increased when compared with DMSO vehicle or 266Y treatment. The E-cadherin protein levels continued increasing through 12, 18, and 24 hours post-treatment (Figure 4B). Similar results were obtained in H520 cells (Supplementary Figure 3B). In addition, we found that ML327 increased E-cadherin expression in two other lung cancer cell lines (H460, H661) and in 2 non-transformed cell lines (HEK293T, HMEC1 (human microvascular endothelial cell)) (Supplementary Figure 3C).

Since ML327 increased E-cadherin mRNA levels within 3 hours, we determined whether *de novo* protein synthesis was required for the effect of ML327 on E-cadherin mRNA expression. For these experiments, we treated cells with cycloheximide, to block protein translation, 1 hour prior to application of ML327 and evaluated expression of E-cadherin mRNA at 6 hours post-treatment. Levels of cyclin D1 protein, which has a very short half-life of ∼20 minutes [15], were assessed as a positive control for the effectiveness of cycloheximide treatment. Cycloheximide treatment did not prevent the increase in E-cadherin mRNA levels in response to the ML327 treatment (Figure 4C). As expected, cyclin D1 protein levels were markedly reduced within the 7 hour experimental interval, but the more stable β-actin protein levels were not significantly altered (Figure 4D-4E). Thus, we conclude from these results that *de novo* protein synthesis is not required for the ML327-induced increase in E-cadherin mRNA expression.

### ML327 activates transcription at the *CDH1* promoter

**Figure 5:**
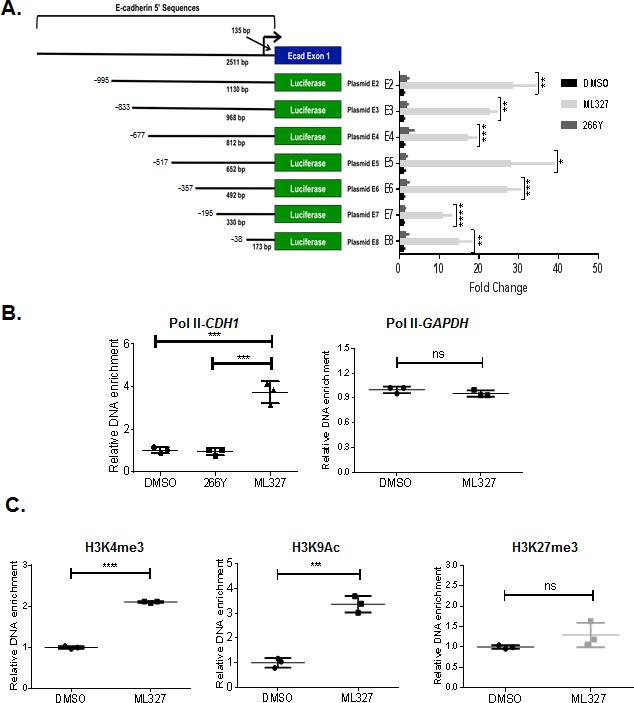
ML327 activity exerts its effect at a proximal region of the E-cadherin promoter **A.** Diagram of E-cadherin promoter reporter plasmids (E2-E8), and the luciferase activity in SW620inv cells transfected with E2-E8 and treated immediately with DMSO, 10 μM ML327, or 266Y for 24 hours. All samples are normalized to DMSO control for each plasmid transfection group. Statistical significance was calculated using a two-way ANOVA (Holm-Sidak method): **** = *p* < 0.00005, *** = *p* < 0.0005, ** = *p* < 0.005, * = *p* < 0.05. Data points represent technical replicates (n = 3) from a representative experiment. The graphed data are representative of three separate experiments with similar results. **B.** Results of ChIP assay demonstrating Polymerase II (Pol II) association with the proximal region (−76/64) of the *CDH1* promoter, or *GAPDH* promoter following 4 hours treatment of SW620inv cells with either DMSO, 10μM ML327, or 10μM 266Y (results from a representative experiment with *n* = 3 technical replicates shown), statistical significance was calculated using unpaired t test, *** indicates *p* < 0.001, ns indicates *p* > 0.05. The graphed data are representative of three separate experiments with similar results. **C.** Results of ChIP assay demonstrating H3K4me3, H3K9Ac, and H3K27me3 association with the proximal region (−76/64) of the *CDH1* promoter following 4 hours treatment of SW620inv cells with either DMSO, or 10μM ML327 (results from a representative experiment with *n* = 3 technical replicates shown), statistical significance was calculated using unpaired *t* test, **** indicates *p* < 0.0001, *** indicates *p* < 0.001, ns indicates *p* > 0.05. The graphed data are representative of three separate experiments with similar results.

To determine if the mechanism of ML327 action involves altered E-cadherin transcription, we conducted experiments using a series of human *CDH1* promoter driven reporter constructs. The full-length *CDH1* promoter (1130bp) encodes consensus binding sequences for several known transcription factors, including AML1, HNF3, P300, SP1, and SNAI [16]. We found that ML327 induces a > 20-fold increase in full-length reporter activation over 266Y. Serial deletion of the distal regions of the promoter reduced the ML327 effect on reporter activation from ∼30-fold to between 10- and 20-fold above control. Diminution of activity was most pronounced when the region between −357bp to −195bp, containing consensus binding sequences for HNF3, P300, AML1 and E-box binding protein (Snail, Slug, Zeb1, and Zeb2) transcription factors was deleted. Importantly, significant ML327-responsive activity was retained with the proximal (173bp) region of the E-cadherin promoter (Figure 5A, Supplementary Figure 4).

Repression of E-cadherin expression involves various epigenetic mechanisms that may include histone modification and/or DNA methylation [17]. Histone modifications, such as methylation, acetylation, and ubiquitination, are linked to gene activation or silencing depending on the precise nature and position of the modification. Promoters of expressed genes are commonly associated with active histone marks, such as Histone3 (H3) lysine 4 methylation (H3K4me, H3K4me2, or H3K4me3) and H3 lysine 9 acetylation (H3K9Ac). In contrast, the chromatin-containing transcriptionally silenced or repressed genes are enriched with binding of histone repressive marks, including H3 lysine 27 trimethylation (H3K27me3) and H3 lysine 9 methylation (H3K9me2, or H3K9me3) [9]. Since binding of specifically modified histones and RNA polymerase II (Pol II) are markers of active gene transcription, we determined whether ML327-induced E-cadherin expression is associated with increased Pol II and activated histone marks in chromatin associated with the promoter of *CDH1* gene. Chromatin immunoprecipitation (ChIP) assays were performed using antibodies against Pol II, H3K4me3, H3K9Ac, and H3K27me3 in SW620inv cells treated with DMSO, 266Y or ML327 for 4 hours. We observed significantly enriched binding of Pol II (4.6-fold), H3K4me3 (2.1-fold), and H3K9Ac (3.4-fold) to the *CDH1* promoter after treatment of SW620inv cells with ML327, and there was no significant difference in H3K27me3 binding after treatment of cells with ML327 (Figure 5B-5C). In contrast, the inactive compound 266Y did not show any effect on Pol II occupancy on the *CDH1* proximal promoter (Figure 5B). In the same experiment, there was no difference binding of Pol II to the GAPDH promoter after treatment of cells with ML327 (Figure 5B). These data suggest that ML327 activates transcription of *CDH1* and that the *cis*-acting elements for this response are present in the proximal 5′ region of the *CDH1* promoter.

### ML327 reprograms gene expression, implicating regulation of HNF4α

**Figure 6:**
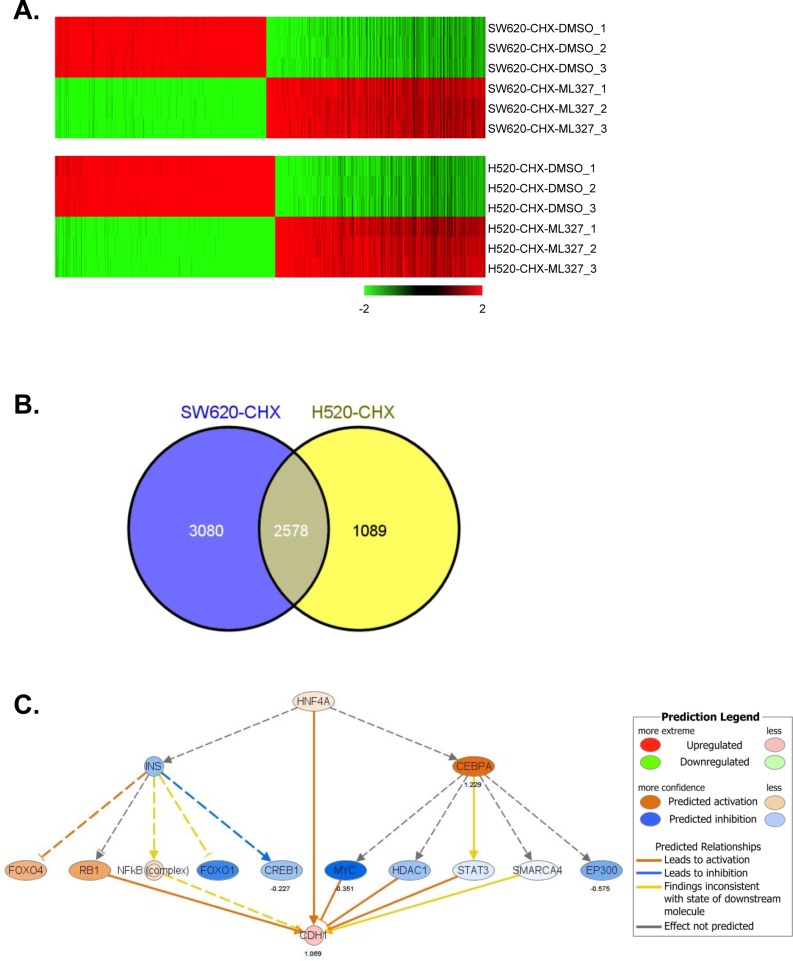
ML327 alters gene expression in a pattern implicating HNF4α **A.** Heat map of 5658 genes *(FDR < 0.0001)* was significantly altered in SW620inv cells (2881 upregulated genes, 2777 down-regulated genes), and 3667 genes *(FDR < 0.0001)* was altered in H520 cells (1792 upregulated genes, 1875 down-regulated genes) in a comparison (CHX-ML327 versus CHX-DMSO), each cell line comparison has three biological replicates. **B.** Venn diagram showing the intersection of the lists of differentially expressed genes in SW620inv and H520 cells. **C.** HNF4α network from the Ingenuity analysis based on the common differentially expressed genes. Oval nodes indicate transcription factors, solid lines connecting nodes indicate direct interactions and dashed lines indicate indirect interactions. Arrows indicate activation and stops indicate inhibition.

We performed RNA sequencing (RNAseq) analysis to evaluate the effect of ML327 on global gene expression patterns in SW620inv and H520. For these experiments, cells (n = 3 per group) were exposed to either DMSO or ML327 in the presence of cycloheximide for 3 hours prior to harvest. To identify genes differentially expressed in ML327-treated cells compared to DMSO-treated cells, we performed count-based differential expression analysis [18]. Using a cutoff of FDR < 0.0001, we found that expression of 5658 genes was significantly altered in SW620inv cells (2881 upregulated genes, 2777 down-regulated genes) and that expression of 3667 genes was significantly altered in H520 cells (1792 upregulated genes, 1875 down-regulated genes) (Figure 6A, supplementary data report 1-2). To further control for false-positive discoveries and focus on a common mechanism in both SW620inv and H520 cells, we took the intersection of the lists of differentially expressed genes in these two cell lines to derive a list of 2578 differentially expressed genes that were in common for both cell lines (Figure 6B). Among these 2578 differentially expressed genes, ∼40% were upregulated and 60% down-regulated in both lines, the top 50 up-regulated or down-regulated genes were listed (Supplementary table). In total, 953 genes, including *CDH1*, were upregulated in both lines. Differential expression of four of the genes identified by RNAseq was confirmed in both SW620inv and H520 cells by qRT-PCR (Supplementary Figure 5). SNAIL/SLUG (*SNAI1/2*) proteins are zinc finger “E-box” binding transcription factors expressed in cells known to regulate EMT and to participate in *CDH1* repression [19]. However, in our study, we observed that both SNAIL1 and Snail2/Slug mRNA levels were increased by ML327 treatment (Supplementary Figure 5). SNAIL1 protein levels were markedly increased in SW620inv cells after treatment with ML327 for 3 hours, while SLUG/SNAIL2 protein levels were minimally changed (Supplementary Figure 6A). Interestingly, we inhibited SNAIL1 expression by small interfering RNA (siRNA) mediated depletion in SW620inv cells, then treated with DMSO or ML327 for 24 hours and observed that E-cadherin protein levels were not increased after marked depletion of SNAIL1. E-cadherin protein levels were increased similarly by ML327 treatment regardless of SNAIL1 depletion by siRNA. ML327 treatment increased Snail1 protein levels in the presence of Snail1 siRNA, but the level of Snail1 protein increase was reduced in comparison with the scrambled siRNA control conditions (Supplementary Figure 6B). It has been previously reported that SNAIL1 binds to its own promoter to repress transcription [20]. Our data suggest that a similar mechanism of de-repression of *CDH1* and *SNAI1* gene transcription may be involved in the response to ML327. Our data also suggest that ML327 increases E-cadherin expression despite a paradoxical increase in binding of SNAIL to the E-cadherin promoter, and that SNAIL1 does not appear to be an important contributor to *CDH1* transcriptional repression in these SW620 cancer cells.

Functional enrichment analysis of our RNAseq data by Ingenuity Pathway Analysis (IPA) showed that significantly more differentially expressed genes were involved in protein ubiquitination (255 genes, right-tailed Fisher's Exact Test, *p* < 1.3 × 10^−13^), gene expression (645 genes, right-tailed Fisher's Exact Test, *p* < 1.3 × 10^−4^), RNA post-transcriptional modification (115 genes, right-tailed Fisher's Exact Test, *p* < 2.3 × 10^−4^) and in the process of embryonic development (575 genes, right-tailed Fisher's Exact Test, *p* < 1.5 × 10^−7^). Hepatocyte nuclear factor 4α (HNF4α), TP53 and Nuclear protein transcriptional regulator 1 (NUPR1) were the top three upstream regulators significantly implicated for common differentially expressed genes found in this analysis, including *CDH1* (right-tailed Fisher's Exact Tests, *p* < 2 × 10^−27^, *p* < 3 × 10^−15^ and *p* < 2 x10^−14^, respectively) (Figure 6C, Supplementary Figure 7A and 7B).

**Figure 7:**
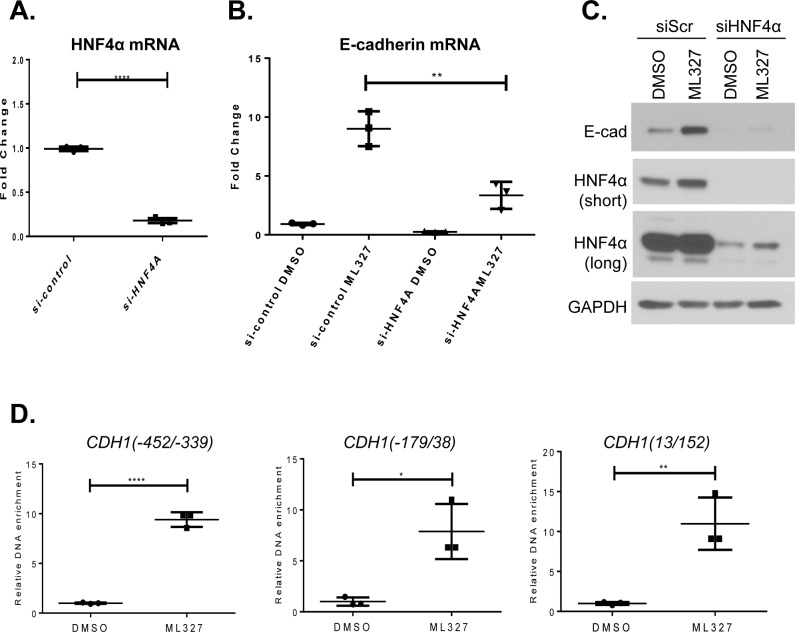
HNF4α is associated with ML327 activity **A.** Quantitative PCR analysis of HNF4α mRNA in SW620inv cells following transient transfection with either ON-target plus Non-targeting Pool control siRNA or HNF4α specific ON-target plus SMART pool siRNA for 48 hours. Fold change relative to si-control is determined by the formula log2^−ΔΔCp^, statistical significance was calculated using unpaired *t* test, *** indicates *p* < 0.001(results from a representative experiment with *n* = 3 technical replicates are shown). The graphed data are representative of three separate experiments with similar results. **B.** Quantitative PCR results for E-cadherin specific mRNA in SW620inv cells following HNF4α knock down with siRNA for 48 hours, then treatment with DMSO or 10μM ML327 for 6 hours, Fold change relative to si-control with DMSO treatment is determined by the formula log2^−ΔΔCp^, statistical significance was calculated using unpaired t test, ** indicates *p* < 0.01(results from a representative experiment with *n* = 3 technical replicates are shown). The graphed data are representative of three separate experiments with similar results. **C.** Western blot showing the effect of HNF4α siRNA mediated knock-down (siScr = control, 48hr. recovery following transfection) on E-cadherin (Ecad) protein expression following treatment with DMSO or 10μM ML327 for 6 hours. HNF4α (short and long exposures) and E-cadherin protein levels are shown. **D.** Relative enrichment of HNF4α binding to the proximal region of the *CDH1* promoter following 4 hour treatment of SW620inv cells with either DMSO, or 10μM ML327 (results from a representative experiment with *n* = 3 technical replicates are shown), statistical significance was calculated using unpaired *t* test, **** indicates *p* < 0.0001, ** indicates *p* < 0.01, * indicates *p* < 0.05. The graphed data are representative of at least three separate experiments with similar results.

To determine whether ML327 induction of E-cadherin involves HNF4α activity, we first inhibited HNF4α expression by small interfering RNA-mediated depletion. Both mRNA and protein expression levels of HNF4α were markedly reduced by specific HNF4α targeting siRNA (Figure 7A and 7C). The effect of ML327 on E-cadherin mRNA expression was significantly reduced from 9-fold to less than 3-fold increase after the depletion of HNF4α expression (Figure 7B). Loss of HNF4α also significantly diminished the effect of ML327 on the E-cadherin protein levels as shown by Western blot (Figure 7C, Supplementary Figure 8). Interestingly, we observed that the HNF4α protein level was elevated after 6 hours ML327 treatment, comparing the control siRNA treated with DMSO, or ML327 (Figure 7C). Chromatin immunoprecipitation (ChIP) analysis of HNF4α protein binding to several *CDH1* promoter regions containing predicted HNF4α binding sites (Supplementary Figure 4) was performed in SW620inv cell lysed after treatment with DMSO or ML327 for 4 hours. We observed significantly enriched binding (6-10 folds) of HNF4α protein to the *CDH1* promoter at three different consensus binding regions, but only a slight (1.7-fold) increase in binding of HNF4α to the *GAPDH* promoter region after the same treatment. (Figure 7D, Supplementary Figure 9). Interestingly, we observed that suppression of HNF4α expression attenuated, but did not completely prevent the induction of E-cadherin expression in response to ML327 (Figure 7B and 7C). Furthermore HNF4α siRNA did not inhibit the ML327 induction of other target genes identified above, including *Snail* and *NFATC2* mRNAs (Supplementary Figure 10), demonstrating that HNF4α is not likely to be the direct target or primary effector downstream of the ML327 action in cells. Taken together, the above results confirm that HNF4α is an important transcriptional enhancer of E-cadherin expression whose activity is necessary for the maximum induction of E-cadherin in response to ML327 and that the effects of HNF4α are likely downstream of the direct molecular target of ML327.

## DISCUSSION

Achieving chemical control of EMT is an attractive goal for experimental cancer therapeutics. We have previously described a series of experiments using high throughput whole cell screening to identify and characterize a chemical compound that restores E-cadherin in colon cancer and lung cancer cells in which E-cadherin is transcriptionally repressed [10]. In the current report, we find that an efficacy-improved compound, ML327, induces E-cadherin transcription and inhibits cell invasion in culture, with little to no toxicity at effective concentrations in SW620inv and H520 cells. ML327 E-cadherin inducing responses were observed in a variety of cancer and non-transformed cells, including the HMEC1 cells that are endothelial in origin. Furthermore, our data demonstrate that ML327 treatment alters the transcription of multiple genes without the need for *de novo* protein synthesis. This suggests that the direct intracellular target of the compound is an important central regulatory node that coordinates or modulates the activation of several downstream transcriptional networks.

Tumor cell dissemination is critical in cancer progression and involves multiple processes that lead to the dissemination of metastases to remote loci. Epithelial to mesenchymal transition is characterized by decreased cell-cell adhesion, increased motility, and invasive properties that may allow carcinoma cells to detach from the primary tumor and invade surrounding tissue, through collective or individual cell migration [7]. To examine the effect of ML327 on EMT, we studied its activity in the classical model system of TGF-β induced EMT in NMuMG mouse mammary epithelial gland cells. Our data indicated that ML327 partially reverses typical molecular features of established EMT in this experimental system.

Cell migration and metastasis are key features of aggressive tumors that are difficult to study *in vivo*, as they not readily accessible within the body of a cancer patient or an experimental animal. *In vitro* studies such as invasion assays and migration assays can model some aspects of these processes, but have inherent limitations that may miss important aspects of the processes as they occur *in vivo*. The CAM assay is a quantifiable *in vivo* model to study metastases and tumor cell migration that overcomes many limitations of the *in vitro* studies [14, 21]. We determined that ML327 inhibits cancer cell motility *in vivo* as reflected by both reduced colony size and a pronounced inhibition of single cell migration from metastatic colonies forming in the chick embryo.

Epigenetic regulation (DNA methylation, histone modification, and microRNAs) has been shown to play a key role in controlling EMT processes. Epigenetics is an important area for potential therapeutic targeting in cancer [9]. The inhibitors of DNMT and HDAC have been shown in preclinical studies to selectively target cancer cells with suppression of metastasis that has been experimentally correlated with E-cadherin upregulation [22, 23]. In fact, we observed increased levels of E-cadherin in response to the HDAC inhibitor, trichostatin A (as shown in Supplementary Figure 1A). In this study, we observed significantly enriched binding of histone active marks (H3K4me3 and H3K9Ac) to the *CDH1* promoter after treatment of SW620inv cells with ML327. Suspecting that ML327 was functioning as a direct HDAC inhibitor, we screened ML327 activity in HDAC inhibition assays (HDAC class I, II, and IV) [10] and sirtuin (HDAC class III) (H.A., unpublished results), but found no evidence for direct inhibition by ML327. We have also shown that ML327 can restore E-cadherin expression in human microvascular endothelial cell-HMEC-1 (supplementary Figure 3), but E-cadherin expression could not be restored by treatment with DNMT inhibitor, 5-azacytidine in the HMEC-1 cells (unpublished observations, H.A.). Even though our observations suggest either direct or indirect regulation of epigenetic processes, further research will be required to identify the direct target of ML327.

In this study, RNAseq analyses were conducted to evaluate the effect of ML327 on global gene expression patterns in SW620inv and H520 and to provide inferential clues as to the direct intracellular target of the compound. Functional enrichment analysis by IPA showed that HNF4α was the top significantly implicated upstream transcriptional regulator for common differentially expressed genes found in this analysis, including *CDH1*. HNF4α has previously been reported to be a central regulator of cell adhesion protein expression in the liver parenchyma of the mouse [24]. Expression of HNF4α has also been linked to regulation of E-cadherin expression in previous studies [25]. We identified twenty-one potential HNF4α binding sites within 1000 bp upstream and downstream of the *CDH1* transcription initiation site using TRANSFAC *in silico* analysis. We found that ML327 increased HNF4α binding to the *CDH1* promoter within a time-frame consistent with the induction of E-cadherin mRNA. Importantly, RNAi knockdown of HNF4α expression significantly, but incompletely, diminished the effect of ML327 on the E-cadherin mRNA and protein levels. However, we observed that knocking down HNF4α had no effect on the increases in Snail1 or NFATc2 mRNAs that were induced by ML327 treatment. Taken together, these data lead us to conclude that HNF4α is not likely to be the direct intracellular molecular target of ML327, but its actions are downstream of the direct target. Ongoing research is aimed at identifying the direct molecular target of ML327 upstream of the multiple genes that are transcriptionally activated in response to the chemical probe.

Identification of the direct molecular target for ML327 is likely to yield important information regarding coordinated regulation of broad transcriptional networks. We anticipate that it will also enable improved understanding and targeting of the pathological EMT process that could be exploited for therapeutic potential in cancer. Therefore, ML327 represents a novel chemical structure with potential for continued pharmacological optimization toward the development of novel therapeutics. The compound ML327 is freely available from the Molecular Library Probe Center Network (MPLCN).

## MATERIALS AND METHODS

### Chemistry

See supplementary materials and methods.

### Cell culture

The SW620 colorectal adenocarcinoma cell line, the H520 lung squamous carcinoma cell line, and the NMuMG murine mammary epithelial cell line, were obtained from the American Type Culture Collection (ATCC) (Manassas, VA). The Hep3 human epidermoid carcinoma cell line were maintained as described previously [26]. All cells were maintained on a humidified atmosphere of 5% CO2 in air at 37°C. SW620 and H520 cells were routinely cultured in RPMI 1640 media supplemented with 10% fetal bovine serum (FBS) (Atlanta Biologicals, Norcross, GA) and 1% penicillin/streptomycin (Corning, Manassas, VA). NMuMG and HEp3 cells were cultured in DMEM media (Life Technologies, Grand Island, NY) supplemented with 10% FBS, 1% L-glutamine and 1% Penicillin/Streptomycin, as above.

### RNA isolation, RT-PCR, and quantitative real-time PCR

Total RNA was isolated from cells using RNeasy kits (Qiagen, Valencia, CA), according to the manufacturer's instructions. RNAs were reverse transcribed using Transcriptor Universal cDNA Master mix. cDNA was analyzed using a quantitative PCR (qPCR) with LightCycler® 480 Instrument II. All PCR instrumentation and reagents were obtained from Roche Biologicals (Basel, Switzerland) with the exception of primers, which were obtained through IDT Technologies (Coralville, Iowa). For primer sequences, see supplementary materials and methods.

### Protein expression

SW620inv cells were used for ICW analysis as described [10]. Whole cells were analyzed in RIPA buffer (150mM NaCl, 1% NP-40, 0.5% Na deoxycholate, 0.1% SDS, 50mM Tris-Cl, pH = 8.0) supplemented with a protease inhibitor cocktail consisting of 1μg/mL aprotinin, 1μg/mL leupeptin, 3μg/mL Pepstatin, 1mM NaVO3, 1mM NaF, 0.5 μM DTT (Sigma Chemical, St. Louis, MO). Protein was separated by 10-15% polyacrylamide gels, depending on the size of the protein of interest. Antibodies used in this study are as follows: E-cadherin (BD Transduction Laboratories, Franklin Lakes, NJ), SNAIL and SLUG (Cell Signaling), β-actin and GAPDH (Sigma Chemical), HNF4α and both anti-mouse and anti-rabbit secondary antibodies are from Santa Cruz. Western blots have been done by regular ECL detection (Millipore) or Odyssey IR imaging system (LI-COR Biosciences) [10].

### Plasmid constructs

The 1.4 kb E-cadherin promoter plasmid (E1) and deletion plasmids E2- E8 were gifts from Dr. Eric Fearon at The University of Michigan [16].

### Luciferase reporter assay

SW620inv cells were co-transfected with luciferase-based reporter plasmid plus a CMV-β-galactosidase plasmid. Transfected cells were treated for 24 hours with DMSO, 10μM ML327, or 10μM 266Y. Luciferase assays were performed as previously described [27]. Activities of luciferase and β-galactosidase were measured in triplicate using the Luciferase Assay System and the β-Galactosidase Enzyme Assay System (Promega, Madison, WI), respectively.

### Chromatin immunoprecipitation (ChIP)

ChIP was performed using Magna ChIPTM A/G kit (Millipore) according to the manufacturer's instructions. Antibodies used in this study are as follows: H3K9Ac, H3K4Me3, and Pol II (Millipore, Billerica, MA). The precipitated DNA was quantified by qPCR as described above. The relative DNA enrichment was determined by the formula 2*ct(IP)-ct(ref)*. For primer sequences, see supplementary materials and methods.

### Immunofluorescence analysis

NMuMg cells (5 × 104/well) were seeded on 8-well chamber slides for 24 hours prior to treatment. After treatments, the cells were rinsed with PBS and fixed with 100% methanol for 15 min at 4°C. The cells were rinsed with PBS and blocked and permeabilized with 2% BSA and 0.2% TritonX-100 in PBS. The cells were then incubated with anti-E-cadherin antibody (BD Transduction Laboratories) and diluted in (1:50) 1% BSA in PBS blocking solution overnight at 4°C. After 3 washes with PBS, the cells were incubated with appropriate secondary antibodies conjugated to fluorescein (1:200; Sigma, St. Louis, MO) or Texas Red (1:500; Invitrogen, Carlsbad, CA) and DAPI (1:2000, Sigma, St. Louis, MO) for 40 min at RT. The cells were washed 3 times with PBS and then mounted with Vectashield mounting medium (Vector Laboratories, Burlingame, CA). Images were captured on an Olympus FV-1000 fluorescent microscope.

### CAM tumor growth assay

Fertilized eggs were obtained from Tyson Foods. Embryonated eggs were handled in accordance with institutional and federal guidelines. The head and neck carcinoma cell HEp3 expressing GFP (100,000 in 100μl PBS) was injected into the allantoic vein of day 10 chick embryos. At 24 hours post injection the chicks were randomized and treated with either compound ML327 or 266Y. Each compound was applied by intra-allantois injection of the compound emulsified into corn oil. Colony formation was imaged at 4, 6 and 8 days post-injection using a fluorescent stereoscope at 50X magnification. Quantitative comparison of colony size was performed at 6 days post injection by measuring the diameter of the colony.

### RNAseq analysis

RNA from SW620inv and H520 cancer cells (n = 3 per group) exposed to either DMSO or ML327 in the presence of cycloheximide for 3 hours was collected using RNeasy kits. Processing of RNA using a TruSeq Stranded mRNA sample prep kit was conducted according to the manufacturer's instructions (Illumina, San Diego, CA). Approximately 27-36 million 50 base pair single-end reads were generated, per sample. We mapped the reads to the human genome hg19 using TopHat-2.0.10 [28]. About 96% or more of the reads were mapped to the genome. Then, following the method of Anders [29], we counted the number of reads that fall into annotated genes by samtools-0.1.19 [30] and HTSeq-0.5.4p5 [31]. Finally, we performed count-based differential expression analysis using edgeR_3.4.2 [18] which implements general differential analyses based on the negative binomial model.

### Inhibition of HNF4α

RNAi studies were performed using SNAIL1 and HNF4A-specific ON-target plus SMART pool siRNA or ON-target plus Non-targeting Pool (Thermo Scientific). Specific siRNA sequences are listed in the supplementary materials and methods.

## SUPPLEMENTARY MATERIAL AND METHODS FIGURES AND TABLE






