# Supplementary material for: Small molecule/ML327 mediated transcriptional de-repression of E-cadherin and inhibition of epithelial-to-mesenchymal transition
Source: Oncotarget. 2015 Jun 10;6(26):22934–48. doi: 10.18632/oncotarget.4473 (PMC4673210; doi:10.18632/oncotarget.4473)
Supplement: Supplementary file 1 [file oncotarget-06-22934-s001.pdf]

Small molecule/ML327 mediated transcriptional de-repression of E-cadherin and inhibition of epithelial-to-mesenchymal transition

Supplementary Material and Methods

Chemistry: **Probe preparation**

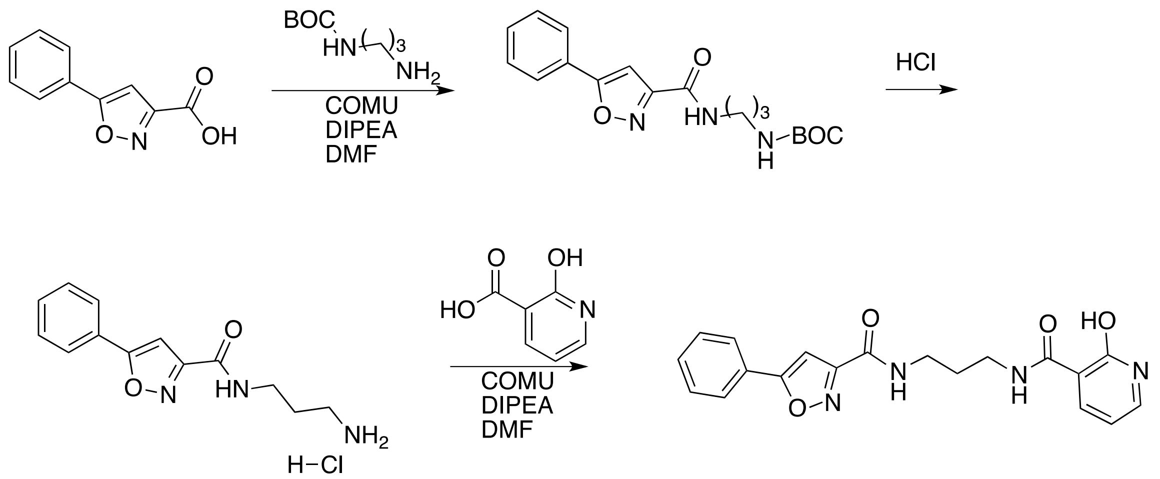

***tert*-Butyl (3-(5-phenylisoxazole-3-carboxamido)propyl)carbamate:** To a solution containing 1.0 g (5.29 mmol) of 3-phenylisoxazole-3-carboyxlic acid and 0.89 g (5.56 mmol) of *tert*-butyl (3-aminopropyl)carbamate in 10mL of DMF was added 2.5 g (5.82 mmol) of (1-Cyano-2-ethoxy-2- oxoethylidenaminooxy)dimethylamino-morpholino-carbenium hexafluorophosphate (COMU**),** followed by 2.0 mL (11.1 mmol) of diisopropylethyl amine (DIPEA). The reaction mixture was allowed to stir at room temperature overnight. The solvents were removed under reduced pressure and the residue was subjected to silica gel chromatography to give 1.55g (85%) of *tert*- butyl (3-(5-phenylisoxazole-3-carboxamido)propyl)carbamate as a yellow solid: : >98% pure by liquid chromatography mass spectrometer (LCMS) at 214 nM; Electrospray ionization (ESI) m/z = 290.2 $[M – C_4H_8]^+$.

***N*-(3-Aminopropyl)-5-phenylisoxazole-3-carboxamide:** A mixture containing 1.5 g

(4.35 mmol) of *tert*-butyl (3-(5-phenylisoxazole-3-carboxamido)propyl)carbamate, 50 mL of dichloromethane (DCM), and 10 mL of a 4.0M solution of HCl in dioxane was allowed to stir at room temperature for 1 hour. The solvents were removed under reduced pressure to give 1.22 g (100%) of *N*-(3-aminopropyl)-5-phenylisoxazole-3-carboxamide, which was used with no further purification: > 95% pure by LCMS at 254 nM; (ESI) *m*/*z* = 246.30 $[M + H]^+$.

***N*-(3-(2-Hydroxynicotinamido)propyl)-5-phenylisoxazole-3-carboxamide (ML327):** To a solution containing 0.3 g (0.278 mmol) of *N*-(3-aminopropyl)-5-phenylisoxazole-3-carboxamide hydrochloride salt, 0.156 g (1.12 mmol) of 2-hydroxynicotinic acid, and 5 mL of DMF was added 0.50 g (1.17 mmol) of COMU followed by 289 µL (1.6 mmol) of DIPEA. The reaction mixture was allowed to stir at room temperature for 2 hours. The solvents were removed under reduced pressure. The residue was subjected HPLC purification to give 92 mg (25%) of *N*-(3-(2- hydroxynicotinamido)propyl)-5-phenylisoxazole-3-carboxamide as a white solid: >95% pure by LCMS at 214nM; ^{1}H NMR (400MHz, d^6-DMSO): δ 9.81 (t, 1H, *J* = 6 Hz), 8.87, (t, 1H, *J* = 5.6 Hz), 8.32 (dd, 1H, *J* = 7.2, 2 Hz), 7.93-7.91 (m, 2H), 7.69-7.67 (m, 1H), 7.56-7.53 (m, 3H), 7.34 (s, 1H), 6.45 (t, 1H, *J* = 13.2 Hz), 3.37-3.28 (m, 4H), and 1.77-1.73, m, 2H); ^{13}C NMR (125MHz, d^6-DMSO): δ 170.7, 163.8, 132.6, 160.1, 158.8, 144.3, 139.7, 131.2, 129.7, 126.7, 126.2, 120.8, 106.6, 100.3, 37.1, 36.6, and 29.6. HRMS calcd for $C_{18}H_{18}N_5O_2$: 366.1461, found 366.1464.

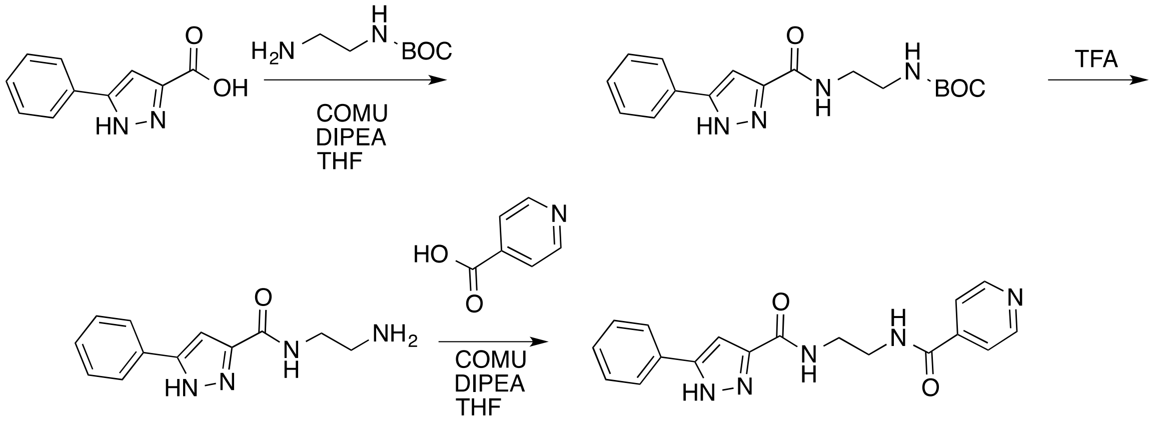

***tert*-Butyl (2-(5-phenyl-1*H*-pyrazole-3-carboxamido)ethyl)carbamate:** To a solution containing 0.5 g (2.66 mmol) of 5-phenyl-1*H*-pyrazole-3-carboxylic acid, 0.45 g (2.79 mmol) of *tert*-butyl (2-aminoethyl)carbamate, and 10 mL of THF was added 1.2 g (2.92 mmol) of COMU followed by 720 µL (3.99 mmol) of DIPEA. The reaction mixture was allowed to stir at room temperature overnight. The solvents were removed under reduced pressure and the residue was subjected to silica gel chromatography to give 380mg (43%) of *tert*-butyl (2-(5-phenyl-1*H*- pyrazole-3-carboxamido)ethyl)carbamate as a white solid, >98% pure by LCMS at 214nM, ^{1}H- NMR (400 MHz, CD_3OD): δ 7.70 (d, *J* = 6.1 Hz, 2H), 7.45 (t, *J* = 6.7 Hz, 2H), 7.39-7.36 (m, 1H),7.02 (s, 1H), 3.46 (t, *J* = 6.0 Hz, 2H), 3.28 (t, *J* = 6.2 Hz, 2H), 1.42 (s, 9H); (ESI) *m*/*z* = 331.2 $[M+H]^+$.

***N*-(2-Aminoethyl)-5-phenyl-1*H*-pyrazole-3-carboxamide trifluoroacetate:** A solution containing 0.380 g (1.15 mmol) of *tert*-butyl (2-(5-phenyl-1*H*-pyrazole-3-carboxamido)ethyl)carbamate, 10 mL of DCM, and ~3 mL of TFA was allowed to stir at room temperature for 6 hours. The solvents were removed under reduced pressure to give a white solid, which was used without additional purification: >96 % pure by LCMS at 214nM, (ESI) *m*/*z*= 231.30 $[M + H]^+$.

***N*-(2-(5-Phenyl-1*H*-pyrazole-3-carboxamido)ethyl)isonicotinamide**

trifluoroacetate (266Y): To a solution containing 0.1 g (0.29 mmol) of *N*-(2-aminoethyl)-5-phenyl-1*H*-pyrazole-3- carboxamide trifluoroacetate, 0.038 g (0.305 mmol) of isonicotinic acid, and 5 mL of THF was added 0.137 g (0.319 mmol) of COMU followed by 79 µL (0.43 mmol) of DIPEA. The reaction mixture was allowed to stir at rt overnight, quenched by the addition of saturated aqueous NH_4Cl, and extracted with DCM. The solvents were removed under reduced pressure and the residue was subjected to HPLC to give 59 mg (45%) of *N*-(2-(5-Phenyl-1*H*-pyrazole-3-carboxamido)ethyl)isonicotinamide trifluoroacetate as a white solid: >98% pure by LCMS at 214nM, ^{1}H-NMR (400 MHz, d^6-DMSO): δ 8.97 (brs, 1H), 8.79 (d, *J* = 6.0 Hz, 2H), 8.46 (brs, 1H), 7.86 (dd, *J* = 4.8 and 1.4 Hz), 7.77 (d, *J* = 8.4 Hz, 2H), 7.44 (t, *J* = 7.4 Hz, 2H), 7.34 (t, *J* = 7.4 Hz, 1H), 7.10 (s, 1H), 3.47-3.43 (m, 4H); ^{13}C NMR (125MHz, d^6-DMSO): δ 164.5, 161.1, 158.7, 158.4, 158.1, 148.8, 143.1, 129.0, 128.2, 125.2, 122.0, 102.4, 38.2; (ESI) *m/z* = 336.20 $[M + H]^+$.

Cell culture

For DNA content measures in culture, cells were plated at 2500 cells/well in a 96-well plate and allowed to attach for 24hrs at 37°C. Treatments were added at t=0 in RPMI + 10% FBS + P/S. Cells were fixed in 100% MeOH for 20 minutes at 4°C, washed with PBS, and stored in PBS at 4°C. For the Syto60 assay, the cells were incubated with 1/20K Syto60 (Invitrogen) in PBS for 30 minutes at RT. The cells were then washed with PBS and emissions at 680nm were read on an Odyssey plate reader (LI-CORE Biosciences). For cell invasion studies, cells were treated for 24 hours with a 10 µM concentration of either ML327 or 266Y, then plated in a matrigel-covered invasion chamber. Cells were allowed 48 hours to invade, then stained with Calcein AM (Life

Technologies, Inc.) and counted using a fluorometer. The proportion of invading cells was calculated, normalized to the DMSO treated controls. Protein stability assays in SW620inv cells were performed by pretreating the cells with 10μM ML327 or DMSO for 2 hours, and subsequently treating with 200 μg/ml cycloheximide (CHX). Cells were harvested at various time points after treatment with CHX. Western blot analysis was performed with anti-HNF4α antibody (Santa Cruz). Dimethylsufoxide (DMSO), cycloheximide (CHX), and Trichostatin A (TSA) were obtained commercially (Sigma Chemical, St. Louis, Mo.).

Primers

E-cadherin (F: TTG ACG CCG AGA GCT ACA C, R: GTC GAC CGG TGC AAT CTT, UPL probe 80), PMM1 (F: TTC TCC GAA CTG GAC AAG AAA, R: CTC TGT TTT CAG GGC TTC CA, UPL probe 7), Occludin (F: AGG AAC CGA GAG CCA GGT, R: GGA TGA GCA ATG CCC TTT AG, UPL probe 84), Vimentin (F: GAC CAG CTA ACC AAC GAC AAA, R: GTC GAC CGG TGC AAT CTT, UPL probe 39).

The following primers were used for the ChIP assay: *CDH1* (-76/64) forward: 5'-GTG AAC CCT CAG CCA ATC AGC GGT-3'; reverse: 5'-GGA GCG GGC TGG AGT CTG AAC TG-3'. *CDH1* (-452/-339) forward: 5'- CAG CTA CTA GAG AGG CTG GGG CC- 3'; reverse: 5'- AGA TGG GGC TCA CTC TTT CAC CC-3'. *CDH1* (-179/38) forward: 5'-ACT CCA GGC TAG AGG GTC ACC-3'; reverse 5'- CCG CAA GCT CAC AGG TGC TTT GCA GTT CC-3'. *CDH1* (13/152) forward: 5'- ACT GCA AAG CAC CTG TGA GCT TGC G-3'; reverse 5'- GCC GAG AGG CTG CGG CTC CAA G-3'. Pol II and control GAPDH primers were supplied with the Magna ChIP™ A/G kit.

SNAIL ON-TARGETplus SMARTpool siRNA Target Sequences

1) GCGAGCUGCAGGACUCUAA
2) AAUCGGAAGCCUAACUACA
3) GUGACUAACUAUGCAAUAA
4) GAGUAAUGGCUGUCACUUG

HNF4A ON-TARGETplus SMARTpool siRNA Target Sequences

1) GACCGGAUCAGCACUCGAA
2) CGGAAGAACCACAUGUACU
3) GGCUGGCAUGAAGAAGGA
4) CCAAGUACAUCCCAGCUUU

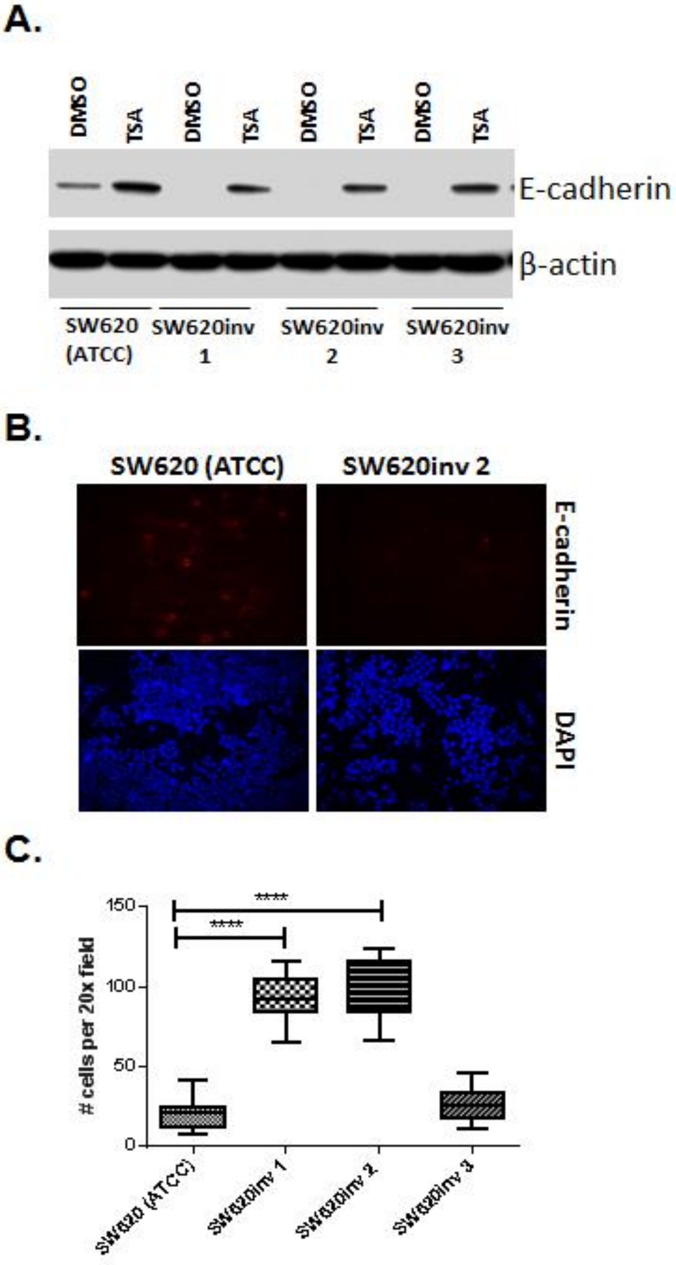

Supplementary Figure 1: Characterization of highly invasive "SW620inv" cells. SW620 (passage 92) from ATCC and 3 independently prepared populations of SW620inv cells (1, 2, 3) were treated with either DMSO or 0.33μM TSA in serum free culture media and cultured for 24 hours. **(A)** Western blot showing E-cadherin and β-actin from 24 hour protein lysates. **(B)** Immunofluorescence of E-cadherin (red) relative

to cell nuclei (blue) comparing SW620 and SW620inv population B. Images are taken at 200x magnification. **(C)** Matrigel transwell invasion assay of SW620 cells compared with 4 independent populations of SW620inv. The number of invading cells is graphed for each group. **** = p<0.00005, ** = p<0.005 (Student’s t-test).

A.

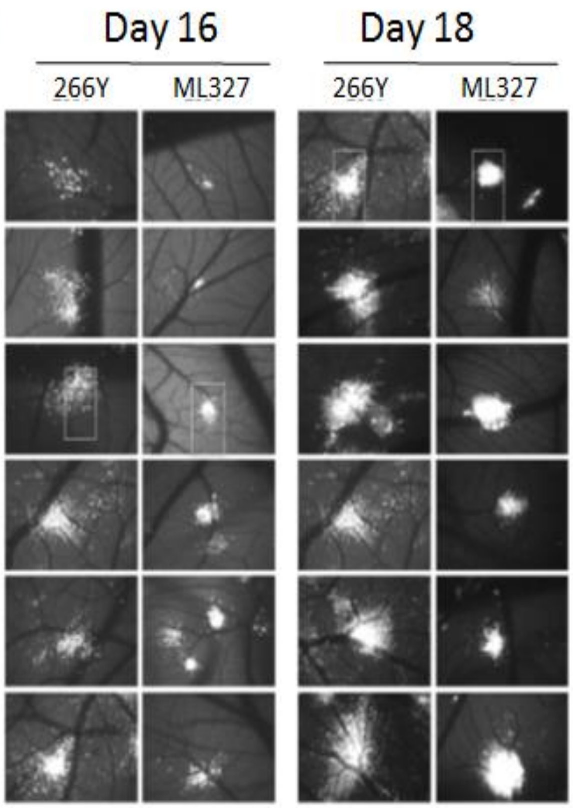

Supplementary Figure 2: Inhibition of tumor cell migration *in vivo*. (A) Microscopic images (50x magnification) of colonies formed in a representative embryo at day 16 and day 18 following injection.

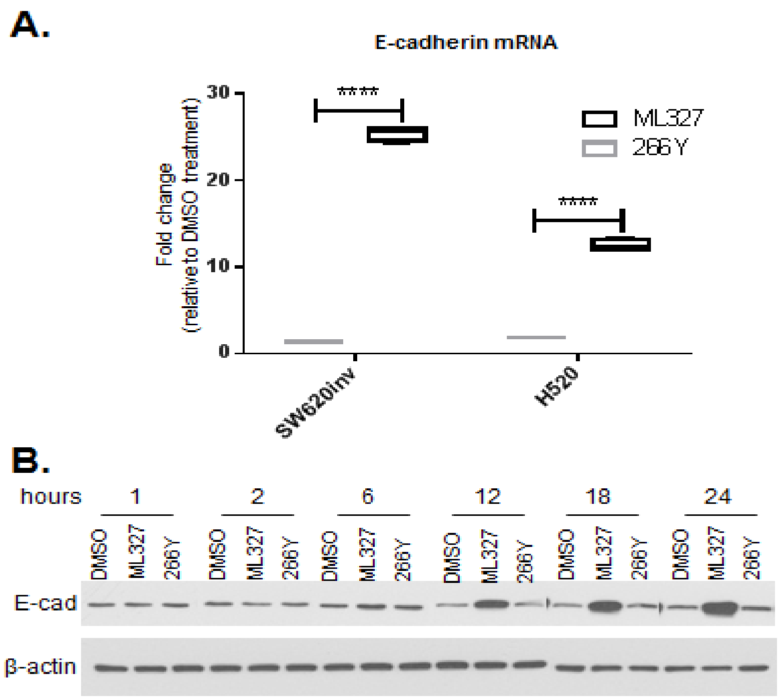

Supplementary Figure 3: Treatment with ML327 increases E-cadherin mRNA and protein expression in both transformed and non-transformed cells. (A) Quantitative PCR results for E-cadherin specific mRNA in SW620inv and H520 cells following treatment for 6 hours with 10 μM ML327, fold change calculated as above. **(B)** Western blot shows time dependent changes in H520 E-cadherin protein expression relative to β-actin following treatment with DMSO, 10 μM ML327, or 266Y. **(C)** Western blot shows induction of E-cadherin protein expression in lung cancer (H460, H661) and in non-transformed (293T, HMEC-1) cells following treatment with 10 μM ML327 for 24 hours.

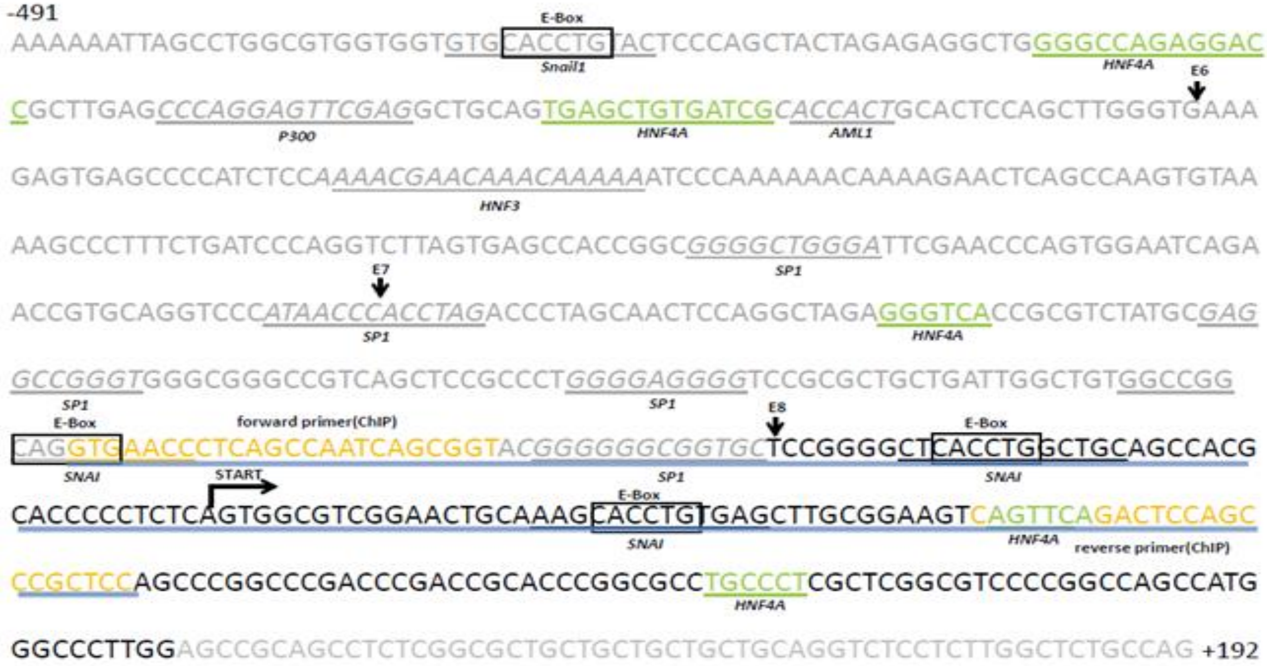

Supplementary Figure 4: Features of the human E-cadherin promoter region implicated in ML327 studies. Schematic showing the sequence of the -491 to +138bp promoter region of the human E-cadherin promoter (adapted from Liu, et al.[14]). Downward pointing arrows with base pair (bp) position numbers indicating the boundaries of plasmids E6, E7 and E8 are indicated. The transcriptional start site (codon AGT) is marked with a bold arrow, E-boxes (CACCTG or CAGGTG) are designated by boxes and SNAI binding sites are underlined and indicated below the sequence. HNF4α binding sites are underlined and indicated with light green sequence. The sequence of the PCR product used in the ChIP assays (139bp product from -76bp to +64bp) is indicated by a bold blue underline. The sequence in yellow font represents primers for ChIP assays. Other consensus binding sequences for additional transcription factors of interest including P300, AML1, HNF3 and SP1 are underlined and indicated by italics. The sequence with dark black typing represents E-cadherin regulatory sequences E8 (-38/+135).

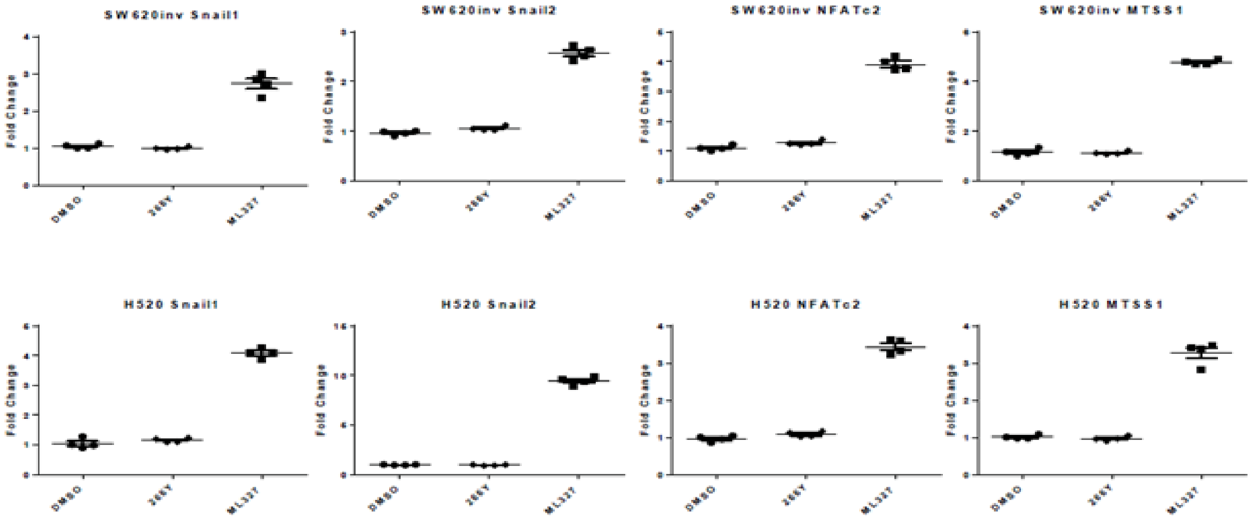

Supplementary Figure 5: Validation of global increased expression of developmental genes implicated by expression profiling. Four upregulated genes (Snail1, Snail2, NFATc2, and MTSS1) from SW620inv_CHX and H520_CHX RNAseq datasets were selected to check the mRNA levels by real time q-PCR in each RNAseq samples, fold change calculated as above.

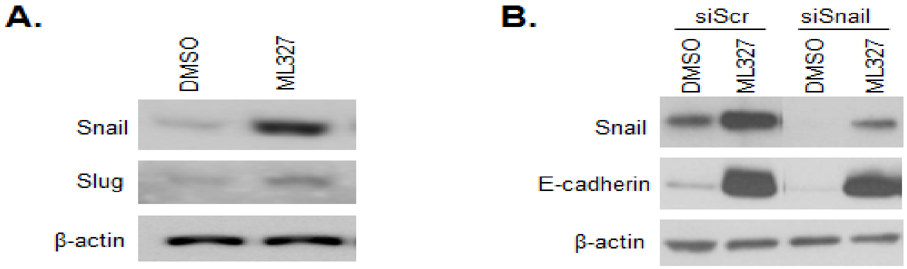

Supplementary Figure 6: SNAIL protein expression is affected by ML327, and depletion of SNAIL does not increase E-cadherin expression in SW620inv cells. **(A)** Western blot showing the Snail and Slug proteins level in SW620inv cells following 3 hours treatment with DMSO or 10 μM ML327. **(B)** Western blot showing the effect of Snail siRNA mediated knock-down (siScr = control, 48hr. recovery following transfection) on E-cadherin protein expression following treatment with DMSO or 10μM ML327 for 24 hours. Snail and E-cadherin protein levels are shown.

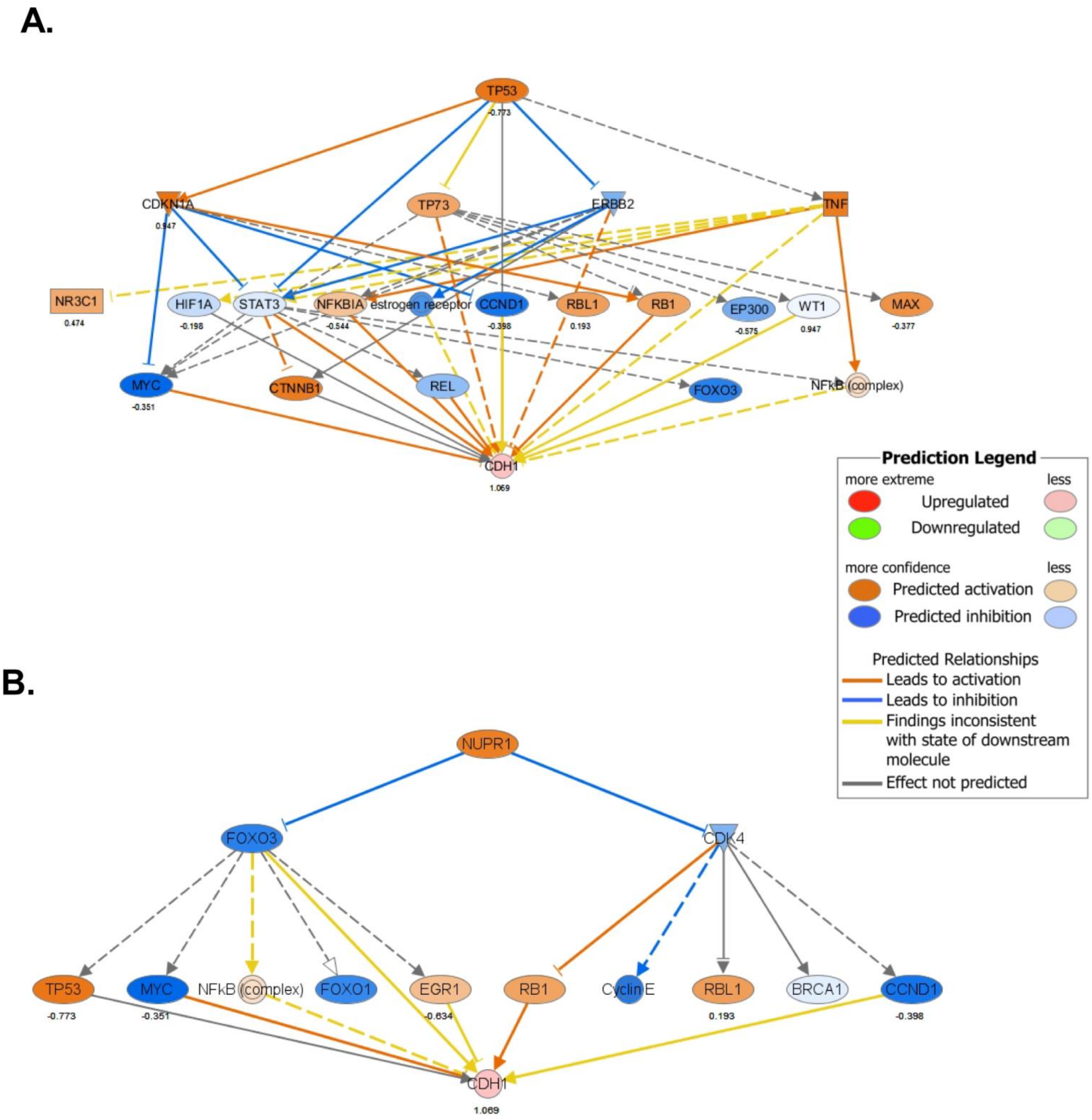

Supplementary Figure 7: TP53 and NUPR1 are additional potential upstream regulators of network perturbations elicited by ML327.

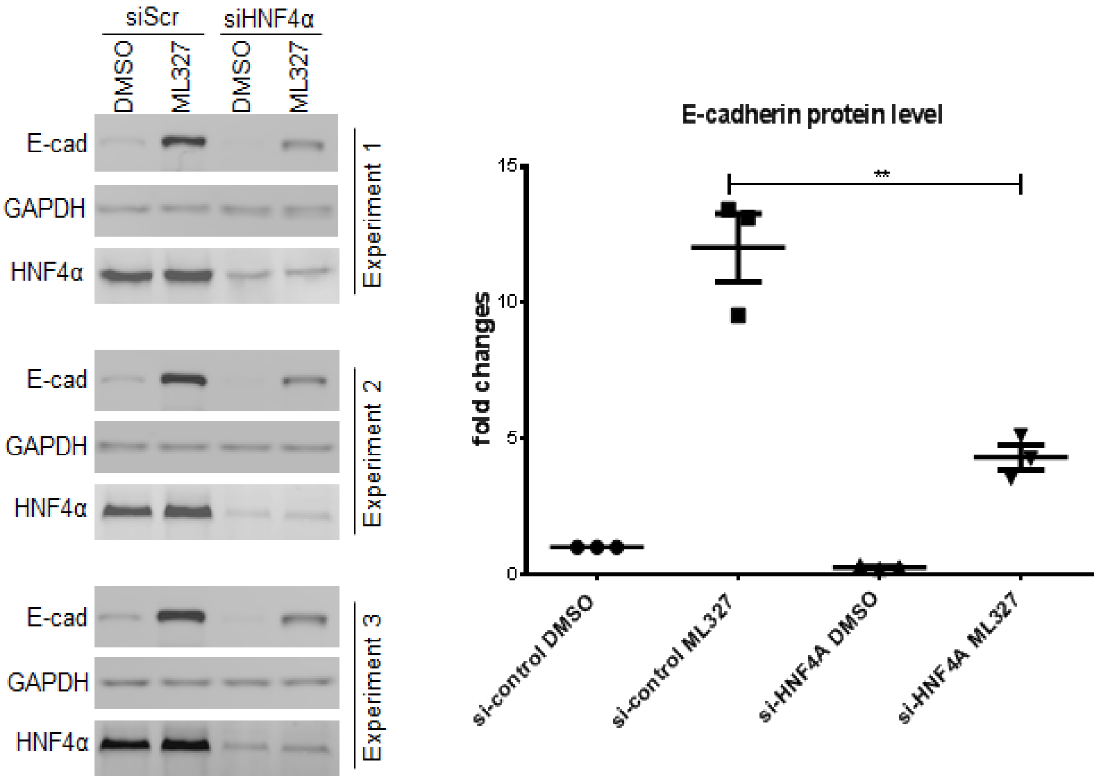

Supplementary Figure 8: HNF4α diminishes the effect of ML327 on restoring E-cadherin protein. LEFT, three independent Western blots showing the effect of HNF4α siRNA mediated knock-down (siScr = control, 48hr. recovery following transfection) on E-cadherin (Ecad) protein expression following treatment with DMSO or 10μM ML327 for 24 hours. E-cadherin and HNF4α protein levels are shown. RIGHT, the graph shows the quantification fold change of E-cadherin protein levels from 3 independent experiments matching left panel with the Odyssey IR imaging system. Statistical significance was calculated using unpaired t test, ** indicates $p<0.01$.

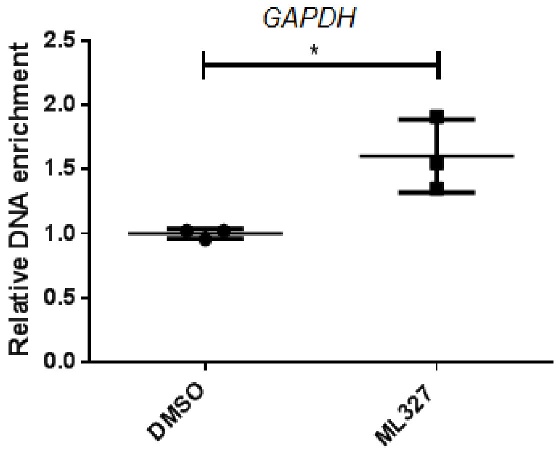

Supplementary Figure 9: ML327 does not significantly alter HNF4α binding to *GAPDH* promoter. Results of ChIP assay demonstrating HNF4α association with the *GAPDH* promoter following 4 hours treatment of SW620inv cells with either DMSO, or 10μM ML327 (results from a representative experiment with n=3 technical replicates shown). Statistical significance was calculated using unpaired t test, * indicates $p<0.05$. The grafted data is representative of at least three separate experiments with similar results.

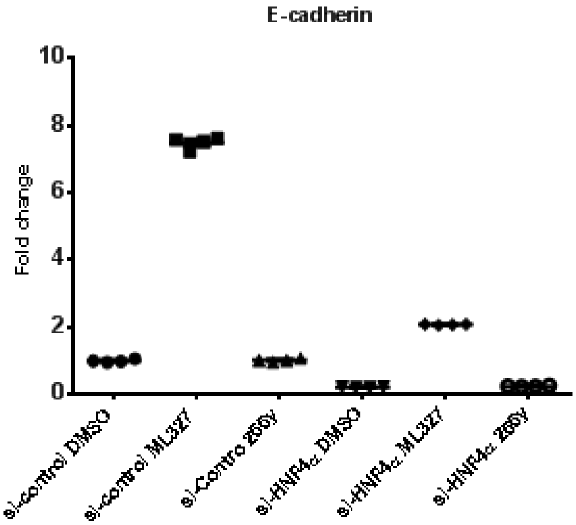
E-cadherin
Fold change
10
8
6
4
2
0
si-control DMSO
si-control ML327
si-Contro Z56y
si-HNF4α DMSO
si-HNF4α ML327
si-HNF4α Z56y

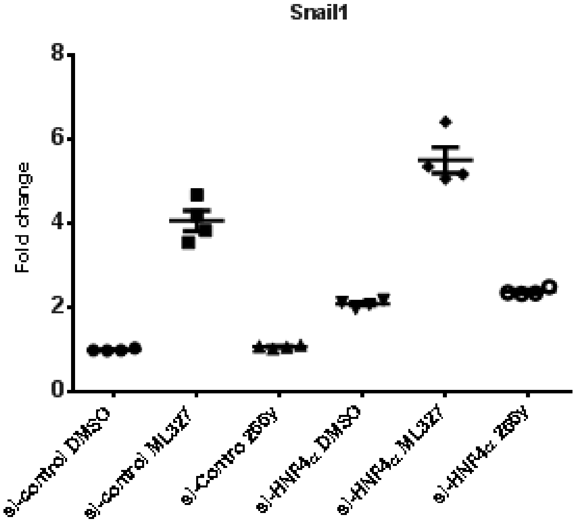
Snail1
Fold change
8
6
4
2
0
si-control DMSO
si-control ML327
si-Contro Z56y
si-HNF4α DMSO
si-HNF4α ML327
si-HNF4α Z56y

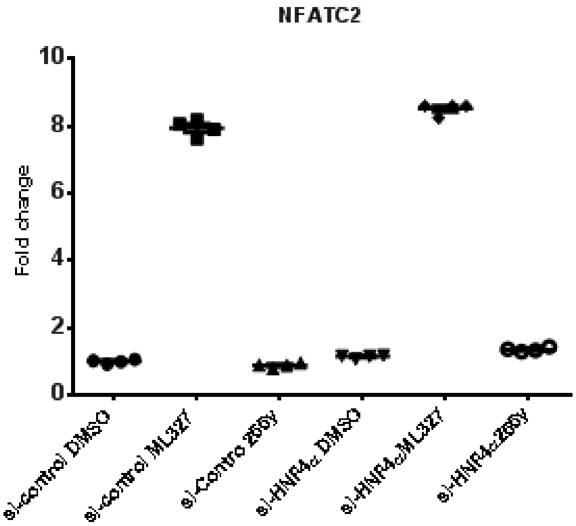
NFATC2
Fold change
10
8
6
4
2
0
si-control DMSO
si-control ML327
si-Contro Z56y
si-HNF4α DMSO
si-HNF4α ML327
si-HNF4α Z56y

Supplementary Figure 10: HNF4α is associated with ML327 activity towards E-cadherin, but not other ML327 effected targets. Quantitative PCR results for E-cadherin, Snail1, and NFATC2 specific mRNA in SW620inv cells following HNF4α knock down with siRNA for 48 hours, then treatment with DMSO, 10μM ML327 or 266Y for 6 hours, Fold change relative to si-control with DMSO treatment is determined by the formula $\log 2^{-\Delta\Delta Cp}$.

Supplementary Table. The top 50 up-regulated or down-regulated genes from the common differentially expressed genes in SW620inv and H520

Up-regulated genes	Down-regulated genes
DMRT2	*LDLR*
EOMES	*ID2*
L3MBTL3	*MED26*
SLC30A2	*CCNL1*
SIX1	*BRD2*
PCDH20	*CLK1*
RREB1	*HSPA8*
BCL11B	*TOB2*
ZNF365	*ZBTB2*
CCDC71L	*PCF11*
ISL1	*AXIN2*
GATA3	*RBM39*
SHC4	*ZNF408*
SLC40A1	*CCNT2*
OTX1	*KDM6B*
CLU	*SNIP1*
SHOX2	*SDE2*
FOXC1	*KANSL2*
RAB39B	*RSRC2*
CCND2	*PABPC4*
FLRT3	*SERTAD3*
HSPA2	*TXNIP*
PCDH1	*EIF5*
CYP2U1	*SIRT1*
GATA6	*PPRC1*
RGS16	*BRD1*
C2CD4A	*KPNA2*
SYNM	*YTHDF1*
LOX	*SETD5*
SIPA1L2	*AKAP8*
IL10RA	*ZNF367*
SVEP1	*ZNF394*
SYBU	*DNAJA1*
ZFHX2	*NXF1*
GFI1	*TOP1*
CEBPA	*PPP1R15B*
HCP5	*WHAMM*
NPPC	*EIF1AD*
IRF6	*LOC100507217*
SEMA6B	*ANKRD13C*
CDH1	*KMT2E*
KCTD12	*SPATA2*
BHLHE41	*TBCC*
HEY2	*FASTKD5*
TMEM151A	*CKS2*
ABCB1	*ZFX*
FZD10	*RBM5*
TMCC2	*ZCCHC3*
PRICKLE1	*SMEK1*
PAX9	*RNF10*
DMRT2	*LDLR*
