# Supplementary material for: Small molecule/ML327 mediated transcriptional de-repression of E-cadherin and inhibition of epithelial-to-mesenchymal transition
Source: Oncotarget. 2015 Jun 10;6(26):22934–48. doi: 10.18632/oncotarget.4473 (PMC4673210; doi:10.18632/oncotarget.4473)
Supplement: Supplementary file 2 [file oncotarget-06-22934-s002.pdf]

SW620_CHX ML327 treatment differential expressed genes from RNA seq analysis

gene	logFC	FDR
ARC	2.650365	0
SYT11	1.627168	0
IER3	-1.06493	3.60E-312
PPP1R15A	-1.03745	7.97E-294
MAP1B	2.166502	8.82E-287
BHLHE41	3.912041	1.66E-280
HSPA8	-1.17217	1.51E-279
VGF	3.380987	1.43E-265
AXIN2	-1.11341	3.25E-258
BMP4	-1.06206	1.06E-252
MAP1A	2.566028	3.50E-240
PCDH1	2.454781	4.70E-235
MYC	-1.05862	2.24E-213
IGFBP3	3.902902	1.48E-211
SYNM	2.824935	2.21E-209
CCND2	5.115318	9.71E-200
CDH1	1.668249	5.92E-190
CXCR4	3.81936	3.61E-184
EGR4	0.935614	1.24E-180
SLC38A2	-0.97812	6.53E-180
AMIGO2	-1.33086	8.80E-180
SOX2	-1.0326	3.20E-177
KDM6B	-1.16994	3.37E-177
ZNF365	4.084967	5.56E-176
HSPA2	2.289826	6.10E-171
TM4SF1	-0.93119	1.13E-170
KLF6	-1.37745	1.34E-170
NRIP3	1.872241	5.16E-170
DLX3	1.29608	4.90E-169
CLU	1.227037	4.84E-165
SOX4	-0.78472	4.19E-164
SSFA2	-0.82225	6.77E-164
AMOTL2	-0.79346	2.09E-163
APLN	1.978667	3.34E-163
RREB1	1.198714	3.02E-162
ARL4A	-0.85065	2.73E-160
CYR61	-0.94366	6.29E-160
FLNC	2.253943	1.03E-159
FOXQ1	-1.029	4.52E-158
PCDH20	4.245841	9.97E-158
PHF23	-1.18753	6.10E-157
PLK2	-0.91402	8.41E-157
TFAP2C	3.247169	4.15E-156
WEE1	-1.36501	2.43E-155
AJUBA	-0.75215	5.09E-155

DUSP6	-0.92697	3.51E-154
ZFP36L1	-0.87931	4.33E-154
MCAM	0.946027	3.70E-149
HIC1	3.295382	1.80E-146
L3MBTL3	1.773138	2.42E-143
SEMA6B	2.01536	8.30E-143
SLC30A2	2.827545	3.61E-139
SERPINB9	1.028621	2.14E-137
SYNPO	2.182134	2.91E-137
EOMES	1.723501	2.04E-136
CCDC71L	1.45189	3.45E-136
WNT7B	2.503306	1.15E-135
GATA6	1.956961	3.34E-135
CEBPA	1.915447	3.31E-134
PPP1R15B	-0.8517	2.51E-133
TMCC2	2.35053	3.27E-131
FZD10	2.146094	8.04E-131
DUSP8	1.361701	8.59E-131
TIPARP	-0.78776	1.42E-130
METRNL	2.353747	1.63E-130
ID2	-0.76149	1.11E-129
SYN1	2.548232	1.39E-129
C11orf96	2.018605	2.37E-129
LDLR	-0.9629	3.45E-129
LBH	2.005099	2.49E-128
TOB2	-0.94568	1.18E-123
NOTCH2	1.721485	2.95E-123
GLIS2	2.205988	1.04E-122
IL10RA	3.551073	1.23E-118
MBIP	-1.05271	1.96E-117
ZCCHC3	-0.83862	2.19E-116
SERTAD3	-1.98579	2.26E-116
RASSF10	3.127944	2.74E-116
SNAI1	1.695972	3.75E-116
BCORL1	0.982444	4.93E-116
YOD1	-1.16866	1.97E-115
SGK223	-0.7062	3.98E-113
NUAK2	-0.92227	1.17E-112
ZFHX2	2.195143	1.22E-112
CDK5R2	2.978172	7.16E-112
PDE4DIP	1.257138	1.00E-111
SRXN1	0.867651	1.59E-111
RBM39	-0.69989	2.71E-111
LOX	3.889973	3.04E-110
SNPH	2.306581	3.21E-110
POU3F2	2.20535	2.56E-109
EIF5	-0.67886	4.87E-109

GFI1	2.154735	5.55E-109
NXF1	-0.78972	9.27E-109
ABCB1	1.745137	2.14E-108
SLC2A3	-0.69725	1.22E-107
DDIT4	1.860964	3.14E-107
RAB39B	1.674998	2.29E-106
NFE2L2	-0.64993	2.79E-106
EPHA2	-0.5284	6.76E-106
RLF	-0.85124	7.87E-103
TIMP3	2.36966	9.72E-103
NUAK1	1.29762	1.23E-101
PIP5K1A	-0.69992	1.33E-101
NRARP	1.532505	2.21E-101
GATA2	1.673138	5.31E-101
CHST3	2.306882	2.19E-100
NFATC2	1.006884	1.04E-99
PADI2	3.679312	2.23E-99
AIM1L	0.877139	5.60E-99
KCNK5	1.395471	5.83E-97
CCNL1	-0.66005	5.98E-96
ARID3B	-0.75695	1.12E-95
PODXL	1.198234	1.42E-95
CSTF1	-0.76645	4.51E-95
NGFR	0.724804	6.23E-95
FA2H	1.444372	8.91E-95
FOSL1	-0.95203	1.24E-94
THEMIS2	2.149818	1.55E-94
ARL4C	1.337423	2.30E-94
TRIM8	-0.75064	2.34E-94
PCF11	-0.7769	3.86E-93
LMX1B	2.481971	5.25E-93
FGF19	0.720324	6.73E-93
NAT8L	2.638474	1.29E-92
SIK1	-0.76442	1.77E-92
RASSF8	1.421965	1.97E-92
IRF2BP2	-0.78873	2.79E-91
RGAG4	1.939765	3.75E-91
RCOR2	1.129548	9.11E-91
PDE4A	1.711782	9.30E-91
ITGA3	0.850196	1.89E-90
NOL4L	1.103302	5.81E-90
CORO2A	1.851058	8.52E-89
FASTKD5	-0.83339	6.98E-88
B3GNT3	1.226466	9.56E-88
TBX21	3.24012	3.41E-87
ADCY9	1.337674	5.27E-87
KIAA0513	2.100627	6.18E-87

SHC4	2.065933	6.18E-87
PDP1	-0.72989	1.24E-86
ZNF408	-1.00408	1.71E-86
SDC3	0.773402	1.59E-85
RASSF5	1.54221	1.85E-85
FOXC1	1.136661	2.04E-85
FZD1	3.017333	2.34E-85
CLDN4	0.829727	6.87E-85
KCTD12	4.324034	6.24E-84
TOP1	-0.57146	2.43E-83
ADAMTS15	1.344771	3.59E-83
TLX1	3.345019	5.54E-82
IL11	2.417742	7.17E-82
ASPHD2	3.66298	7.35E-82
ZFP90	1.346713	7.77E-82
BRD2	-0.54984	1.12E-81
BIRC3	0.951965	2.35E-81
SYBU	1.397202	6.50E-81
PPRC1	-0.68643	1.30E-80
GATA3	1.426726	1.54E-80
KANSL2	-0.71406	1.71E-80
SETD1B	-1.17844	1.77E-80
SERTAD1	-0.62782	1.92E-80
PHLDA1	-0.47085	2.48E-80
TNFSF9	2.339988	4.04E-80
SLC40A1	2.576093	4.05E-80
SLC16A14	1.363519	1.49E-79
CDC25A	-0.89009	6.14E-79
SLC52A3	1.788216	9.41E-79
SYT12	2.639516	1.32E-78
PAX9	1.818765	2.68E-77
SLC2A1	-0.52506	2.68E-77
TMEM2	-0.76867	5.66E-77
SIRPA	1.19547	7.56E-77
PTPRU	1.796754	1.10E-76
YTHDF2	-0.70515	1.23E-76
RGS3	1.034799	1.64E-76
C6orf62	-0.70039	1.65E-76
CRKL	-0.6072	2.01E-76
ZBTB2	-0.83432	5.34E-76
ATMIN	-0.60888	5.86E-76
BCAR3	1.379745	6.56E-76
NEDD9	1.275758	8.25E-76
TMEM151A	4.343774	9.99E-76
ELOVL7	1.400271	1.34E-75
CDKN1A	1.084729	2.18E-75
DLL4	1.261897	3.56E-75

ZCCHC12	2.360678	1.12E-74
TGM2	1.126341	1.40E-74
CLK1	-0.75323	3.27E-74
MMP15	1.794649	3.34E-74
FAM167A	1.400547	5.40E-74
COL4A2	0.973694	5.51E-74
TUBA1A	0.816121	5.81E-74
VDR	1.241932	6.72E-74
PHOSPHO1	3.229714	1.17E-73
KPNA2	-0.51682	3.14E-73
NR4A3	1.287988	8.71E-73
CLDN3	1.534643	9.45E-73
FN1	1.026129	2.36E-72
ITGA5	0.916131	2.36E-72
WDR26	-0.52603	2.46E-72
FZD4	1.701472	3.62E-72
CASZ1	0.888963	5.11E-72
PDX1	2.528712	8.29E-72
CCNT2	-0.78726	1.15E-71
SLC4A8	1.423735	1.45E-71
LFNG	1.21788	2.71E-71
NPTXR	1.228606	4.58E-71
MRPL44	-0.70372	6.54E-71
EAF1	-0.61808	8.77E-71
HS6ST1	0.927176	9.07E-71
COL12A1	3.320849	1.57E-70
UNC5B	2.803963	4.49E-70
SOX9	-0.6036	4.83E-70
CELSR2	1.06542	6.45E-70
DIRAS2	2.156951	1.19E-69
PNRC2	-0.53024	1.75E-69
RNF10	-0.52069	1.87E-69
DLG3	0.682536	9.51E-69
SOWAHA	1.632264	1.69E-68
CX3CL1	1.694253	2.61E-68
SHOX2	2.868561	2.63E-68
BRD1	-0.65218	5.54E-68
SIPA1L2	3.008343	6.29E-68
LRP4	0.962302	8.05E-68
EIF1AD	-0.88139	9.32E-68
ZCCHC24	0.945216	1.47E-67
DLL1	3.68863	1.52E-67
RIOK3	-0.54606	1.89E-67
FOXD1	1.327961	1.99E-67
SBDSP1	-0.56692	4.71E-67
BDNF	4.533067	5.61E-67
BCL2L1	-0.5036	8.00E-67

CEBPB	1.423068	8.07E-67
HSPA5	-0.46152	1.08E-66
SLC16A9	1.216942	1.61E-66
CCDC184	6.168064	4.41E-66
POU4F1	1.855421	5.27E-66
PLAU	0.782388	6.81E-66
IER5L	0.923147	1.52E-65
SOX8	1.901316	1.94E-65
RSPRY1	-0.65811	2.46E-65
DAB2IP	0.858434	5.33E-65
FSTL3	1.26202	7.09E-65
MAFG	-0.63389	7.20E-65
OTUB2	2.244147	7.78E-65
STX11	4.258478	1.23E-64
C11orf45	1.538672	2.24E-64
CAMK2N1	2.123033	2.29E-64
PTCH2	1.968816	3.31E-64
C15orf62	-0.91858	4.20E-64
SRF	-0.54052	5.36E-64
SLC16A6	-0.77984	1.06E-63
LARP4B	-0.53975	1.21E-63
SIRT1	-0.56401	2.07E-63
DDX28	-0.86761	3.41E-63
H1F0	0.506128	3.70E-63
TXNIP	-0.55941	4.51E-63
PHF21A	0.694028	4.60E-63
CHSY1	1.168536	4.98E-63
TPPP	1.394055	5.46E-63
RSRC2	-0.58075	7.27E-63
IPMK	-0.77988	8.00E-63
BCL11B	3.884053	1.07E-62
HOXD8	2.060532	1.30E-62
CRK	-0.54621	1.54E-62
RBM5	-0.48887	7.59E-62
FGFR3	1.916868	8.65E-62
DCBLD2	-0.45576	2.06E-61
DDX20	-0.72432	2.40E-61
SNIP1	-0.90185	2.55E-61
ZNF367	-1.41564	4.21E-61
PCNXL4	-0.60057	4.52E-61
DCAF12	-0.66656	5.40E-61
LOC652276	-0.98927	5.79E-61
FUT1	2.031204	7.71E-61
TNFRSF1B	0.668207	8.39E-61
ADM	1.058646	8.59E-61
DMRT2	1.640708	9.28E-61
CBX4	0.50145	1.20E-60

EGLN3	2.22401	2.14E-60
ALOXE3	1.272394	3.12E-60
SLC20A2	-0.68207	4.17E-60
KLF15	2.128434	6.84E-60
MEX3B	2.241338	1.60E-59
GRAMD1B	0.826928	1.94E-59
SCAMP5	1.573376	2.44E-59
UBC	-0.59348	2.57E-59
KAZN	1.810439	3.43E-59
JAG2	0.772265	3.74E-59
TPBG	1.875606	4.25E-59
RAB11FIP5	1.433299	4.49E-59
NRROS	1.617089	4.55E-59
DNAJA1	-0.57271	4.85E-59
SMNDC1	-0.78697	9.20E-59
NFIC	0.714493	1.67E-58
PFKFB3	0.56334	2.05E-58
SGK1	1.83298	2.17E-58
AKIRIN1	-0.46952	2.26E-58
ZNF618	1.037476	2.57E-58
RSBN1	-0.79372	6.87E-58
GDF11	0.755022	8.18E-58
CMTM3	0.694266	1.20E-57
IRF6	1.358305	1.55E-57
CKS2	-0.66326	1.61E-57
AURKA	-0.57739	3.07E-57
C1orf115	0.921723	3.50E-57
SBDS	-0.53645	3.90E-57
ZNF217	0.577449	7.72E-57
DIXDC1	0.942707	1.33E-56
CDS1	3.050601	1.39E-56
EFR3B	0.969453	1.51E-56
TEX19	3.16962	3.10E-56
JMJD1C	-0.58842	3.39E-56
RBM48	-0.92473	3.54E-56
CNN2	-0.43242	5.11E-56
SMEK1	-0.54873	5.80E-56
EIF4A2	-0.41542	1.85E-55
LRRC8C	1.316397	1.93E-55
PGF	2.051714	2.14E-55
SPRED2	-0.62148	2.21E-55
TSC22D1	-0.52494	3.29E-55
MED26	-0.81294	4.39E-55
ZIC2	3.509152	4.66E-55
LOC1005072	-0.53647	5.48E-55
C2CD4A	4.614113	8.63E-55
C16orf70	-0.61378	1.37E-54

FLNB	0.530794	1.90E-54
SIX1	1.177461	2.18E-54
PMP22	1.680541	2.19E-54
MAFF	-0.50174	2.57E-54
ESAM	2.511014	2.84E-54
FGF18	1.114979	2.86E-54
NACC1	-0.66193	4.38E-54
TERF2IP	-0.54605	5.14E-54
EZR	-0.38648	5.17E-54
KBTBD2	-0.65024	6.64E-54
CDKN1B	-0.69048	8.64E-54
FAM46C	1.906633	1.15E-53
CHRM4	3.252852	1.20E-53
HEY2	2.039343	1.25E-53
NR3C1	1.755255	1.37E-53
SLC44A2	0.755367	1.46E-53
ZNF394	-1.04755	1.78E-53
OSBP	-0.44499	2.25E-53
SOCS2	0.983301	2.39E-53
RNF19A	-0.72554	2.94E-53
RTN4RL2	2.602568	3.38E-53
THBS1	-0.60418	3.43E-53
EHD2	3.223981	3.66E-53
KIAA1462	1.515011	3.74E-53
MAFA	2.61587	4.96E-53
GADD45G	2.846598	5.18E-53
C3orf38	-0.69922	8.69E-53
MARVELD3	1.928335	1.16E-52
RNF4	-0.59637	1.35E-52
VCPKMT	-1.16434	1.36E-52
FOXA1	6.506035	1.64E-52
AAR2	-0.56346	2.67E-52
CELSR3	0.806953	2.86E-52
KLF10	-0.49355	4.30E-52
CYP2U1	1.543171	5.90E-52
DR1	-0.50752	6.07E-52
SLAIN1	0.963228	7.89E-52
LPHN1	0.924979	9.43E-52
SBK1	1.182207	1.01E-51
ZIC5	5.172576	1.02E-51
CRY1	-0.72685	1.13E-51
AXIN1	-0.61551	1.59E-51
AKAP5	1.698522	1.99E-51
FAM46A	0.930371	1.99E-51
TMEM51	2.197451	2.16E-51
CYP24A1	2.307179	3.29E-51
SMG8	-0.69364	3.31E-51

ARHGAP23	-0.50194	3.90E-51
MSL1	-0.72927	4.44E-51
PAQR8	0.793112	4.79E-51
SETD5	-0.6027	6.75E-51
ARHGDIB	0.954807	7.62E-51
PRICKLE1	5.974593	9.95E-51
NUFIP2	-0.62468	1.37E-50
GPBP1L1	-0.63857	1.49E-50
RASGRP1	3.11845	1.74E-50
RGS16	1.725331	1.80E-50
YTHDF1	-0.60846	1.82E-50
RUNX3	0.654273	2.06E-50
SP6	1.450548	2.31E-50
AMOT	1.044634	2.52E-50
BLCAP	-0.62597	4.36E-50
WHAMM	-0.66255	8.18E-50
CKAP4	1.130647	1.07E-49
EHF	0.593371	1.08E-49
BMP2	5.956867	1.30E-49
MFSD2A	1.152369	1.40E-49
ZFX	-0.52759	1.64E-49
ANKRD13C	-0.59666	1.86E-49
SYVN1	-0.64031	2.12E-49
TARDBP	-0.46473	2.21E-49
ITPKB	1.821017	2.28E-49
TBCC	-1.12117	2.28E-49
COL1A1	1.44323	2.81E-49
EPAS1	3.533819	3.04E-49
AKAP8	-0.61043	3.05E-49
ARHGEF26	1.101604	3.18E-49
RRP8	-0.56365	3.44E-49
ISL1	3.293561	4.94E-49
SPATA2	-0.80733	5.06E-49
TUBB2B	2.332755	6.17E-49
CSF1	3.042502	6.87E-49
BTG3	1.024002	8.40E-49
HPCAL4	3.532658	9.11E-49
FAM84B	2.246308	9.41E-49
RBM47	0.704643	1.34E-48
N4BP3	2.743374	1.43E-48
HCP5	1.601938	2.36E-48
SDE2	-0.71851	2.66E-48
RAET1K	1.306768	2.66E-48
NPPC	3.590912	2.81E-48
NAGS	3.250369	2.85E-48
PCBP1	-0.4407	3.53E-48
LAMB3	0.776166	3.75E-48

VEGFA	1.024837	4.09E-48
PABPC4	-0.44739	1.65E-47
KMT2E	-0.67876	1.77E-47
MT1H	1.193114	2.47E-47
SVEP1	1.068523	2.82E-47
GPR180	0.858024	3.67E-47
EFNA3	3.674003	3.80E-47
CDR2L	0.641817	4.43E-47
SCN8A	0.887884	4.62E-47
VCPIP1	-0.80762	5.10E-47
RAG1	1.16313	5.48E-47
PRR15	1.91025	8.73E-47
STOX1	0.905703	1.03E-46
ELL	-0.80181	1.05E-46
CCDC160	3.91559	1.13E-46
UGDH-AS1	0.600014	1.31E-46
FILIP1L	0.600036	1.32E-46
EXOC8	-0.68697	1.43E-46
KLHDC7A	0.828057	1.75E-46
DLX6	1.992559	1.79E-46
RLIM	-0.54131	1.81E-46
DAPK3	-0.59043	1.82E-46
TCEB3	-0.61306	2.20E-46
POLR1C	-0.9237	2.50E-46
FAM131B	4.349047	2.58E-46
FLRT3	3.818009	2.73E-46
COQ10B	-0.63173	2.78E-46
OTX1	2.095089	3.04E-46
ESYT3	1.329614	3.04E-46
ZXDB	-0.71865	3.08E-46
FAM78A	4.418081	3.23E-46
DDX3X	-0.53588	3.46E-46
HIST1H1D	3.646283	3.51E-46
SH3PXD2B	1.026093	4.29E-46
KLF13	0.945802	4.65E-46
TMEM169	1.147016	6.13E-46
EPOR	1.258138	8.02E-46
KCNC1	3.88554	8.13E-46
ANGPTL4	3.270411	8.75E-46
ZYX	-0.45198	8.92E-46
EVX1	4.956275	1.12E-45
NOTCH1	0.564728	1.31E-45
KRT18	-0.48932	1.40E-45
USP27X	2.212875	1.64E-45
TBC1D2B	0.637066	1.78E-45
VIPR1	1.97485	1.88E-45
TLR4	1.410435	2.13E-45

GDPD1	1.467513	2.52E-45
FAM105A	2.386459	2.91E-45
FZD8	5.780443	2.96E-45
RAB5A	-0.48565	3.16E-45
CNOT4	-0.51947	3.85E-45
HEYL	1.501319	4.09E-45
ZNF3	-0.6038	4.53E-45
FHOD1	0.587509	4.88E-45
MAX	-0.49831	5.09E-45
ZNF207	-0.37655	5.09E-45
RAB5B	-0.52177	5.56E-45
RTN4RL1	4.305188	5.63E-45
SPATA12	1.151104	6.01E-45
TOR1AIP2	-0.57484	6.34E-45
GADD45B	-0.44799	6.93E-45
GEM	4.40149	8.69E-45
PPARGC1B	2.839068	9.42E-45
TRIB2	1.074785	1.23E-44
C3orf58	4.961904	1.24E-44
AHNAK2	2.214201	1.26E-44
CCNT1	-0.61155	1.36E-44
SOWAHB	3.05095	1.36E-44
PVRL2	-0.49714	1.82E-44
ADRBK2	2.157625	1.94E-44
CRABP2	0.802679	1.97E-44
ELFN2	1.567816	2.33E-44
IFFO2	0.836139	3.38E-44
YY1AP1	-0.49691	3.88E-44
ZNF35	-0.847	4.56E-44
DDIT3	-0.91877	5.22E-44
RAI2	3.355455	5.64E-44
AIM1	2.022403	6.52E-44
NKX2-8	4.694615	9.77E-44
NDRG4	1.090582	1.03E-43
MN1	2.748593	1.17E-43
CTDSP2	-0.44463	1.17E-43
NCEH1	-0.50723	1.51E-43
FBXO28	-0.56224	1.54E-43
TRIB1	-0.54464	1.57E-43
RNF43	-0.51527	2.08E-43
DKK1	-0.62365	2.13E-43
TFIP11	-0.68587	2.43E-43
HIAT1	-0.58147	2.67E-43
RBBP6	-0.50284	2.69E-43
CCND1	-0.38423	3.73E-43
SPTY2D1	-0.53713	4.98E-43
C5orf51	-0.42671	5.26E-43

KIF5A	1.479679	7.80E-43
SP9	2.10589	8.64E-43
ADAM9	-0.44022	9.22E-43
KCTD5	-0.48145	1.07E-42
ARX	5.178007	1.10E-42
PALLD	0.510203	1.10E-42
RASAL1	1.321277	1.28E-42
VLDLR	1.886781	1.31E-42
AKAP12	-0.57217	1.56E-42
DRAM1	1.34536	1.62E-42
BRPF1	-0.68833	1.77E-42
ARHGEF18	-0.47354	1.90E-42
GSG2	-0.93024	1.96E-42
QTRTD1	-0.49441	2.12E-42
COL2A1	1.449186	2.43E-42
ZNF282	-0.47811	2.47E-42
TRA2B	-0.4323	2.49E-42
SEMA7A	0.813421	2.60E-42
IPPK	-0.77724	2.85E-42
CITED4	1.376463	3.18E-42
BRD8	-0.54528	4.06E-42
FAM101B	0.93029	5.26E-42
DNAJB1	-0.49217	7.26E-42
MEIS2	0.990606	7.52E-42
MAST3	0.959477	8.59E-42
9-Mar	1.484844	9.01E-42
CCNB1	-0.3854	1.20E-41
ZNF185	1.146051	1.41E-41
HHEX	1.061206	1.64E-41
AREL1	-0.60209	1.68E-41
SOX18	1.518305	1.68E-41
ADRM1	-0.39768	1.86E-41
TFCP2L1	0.710563	1.86E-41
TMEM115	-0.81738	1.94E-41
CDK16	-0.53489	2.19E-41
SF3B4	-0.52634	2.24E-41
BAG5	-0.53699	3.15E-41
ZNF263	-0.56961	3.58E-41
TSKU	1.747217	3.66E-41
ZDHHC5	-0.44187	3.92E-41
TSHZ3	5.724154	4.05E-41
ZNF830	-0.85314	4.20E-41
NETO2	0.549007	4.55E-41
LEPREL2	1.514665	4.59E-41
NIPAL1	2.091076	4.74E-41
LATS1	-0.58219	5.46E-41
TMEM11	-0.6574	6.47E-41

MAP2K4	-0.50081	6.87E-41
OXTR	1.499511	7.23E-41
PAK6	1.203789	7.43E-41
PDE12	-0.44726	7.63E-41
EP300	-0.49424	7.65E-41
PRKAG2	0.896911	7.96E-41
NIP7	-0.42766	8.92E-41
MSL2	-0.54865	9.07E-41
ST5	0.611847	9.22E-41
BRAP	-0.48317	1.03E-40
ID3	2.448114	1.08E-40
PPP1R14C	1.487671	1.10E-40
BATF2	1.860804	1.25E-40
CWC25	-0.57983	1.36E-40
CCDC85C	0.606302	1.55E-40
CCDC174	-0.54746	1.57E-40
SCD5	0.797285	1.60E-40
SPRY2	-0.50874	1.88E-40
LRRC75A	1.297297	2.06E-40
SETD1A	-0.65281	2.14E-40
ARHGEF40	1.018458	2.45E-40
HNRNPA0	-0.42219	2.46E-40
WNT11	0.969412	3.51E-40
NACAD	2.836684	3.75E-40
TPPP3	1.107745	3.75E-40
ZNF853	1.375782	4.40E-40
BHLHE40	-0.47416	4.61E-40
GPBP1	-0.48804	5.16E-40
NCOA7	0.886062	6.28E-40
FOXJ1	2.105943	7.02E-40
HYLS1	0.846922	7.22E-40
PDLIM1	0.679783	7.73E-40
CYP1A1	1.431245	7.74E-40
IRS1	0.7552	7.79E-40
A4GALT	2.700199	7.91E-40
C1orf52	-0.70101	8.38E-40
RBM15B	-0.45697	9.22E-40
LHX6	0.590979	9.36E-40
NUP50	-0.48839	1.16E-39
PAQR7	1.417541	1.29E-39
MTA2	-0.40055	1.37E-39
RUNDC1	-0.57301	1.39E-39
NIPAL4	2.815631	1.43E-39
WNT1	8.64182	1.55E-39
LAMA3	0.933389	1.65E-39
M6PR	-0.43035	1.84E-39
MAMLD1	0.836257	1.98E-39

HLA-B	0.658428	2.01E-39
ZCCHC8	-0.54028	2.04E-39
LZTS1	3.664592	2.11E-39
LOC1005066	1.029183	2.46E-39
FBXO32	-1.35159	2.51E-39
CLCN5	0.891698	2.63E-39
DCTN5	-0.45834	2.86E-39
YRDC	-0.52779	3.08E-39
IFIT5	0.816094	3.88E-39
SPTBN2	0.536936	3.88E-39
ZNF710	0.778327	4.48E-39
FGD6	0.602502	4.54E-39
NECAP1	-0.55867	4.86E-39
CHMP1B	-0.5832	5.25E-39
TUSC2	-0.77811	5.49E-39
ASAP3	2.029242	5.78E-39
TFAP2A	1.987363	6.15E-39
PLAGL2	-0.50206	7.05E-39
SH3D19	-0.46952	7.28E-39
CRNKL1	-0.49608	7.36E-39
GPR3	1.146909	9.38E-39
BCAR1	-0.43694	9.72E-39
TUBB4B	-0.33836	1.06E-38
FGFR1OP2	-0.45697	1.19E-38
ATF4	-0.3416	1.31E-38
CCRN4L	-0.81778	1.56E-38
PTPRS	1.133607	1.57E-38
ITPRIP	0.555099	1.60E-38
TUBB6	0.603882	1.61E-38
HSPB8	3.908833	1.68E-38
FAM102B	1.194863	2.01E-38
PCK1	2.606848	2.10E-38
CBLL1	-0.50831	2.17E-38
CHGB	0.584853	2.22E-38
RPL22L1	-0.77458	2.38E-38
KIF5B	-0.45586	2.39E-38
CLDN23	2.433049	2.56E-38
MT1G	0.660134	2.81E-38
SNN	1.534534	3.19E-38
C15orf39	0.656237	3.94E-38
AKAP10	-0.56495	6.71E-38
FOXA3	-0.57188	7.22E-38
PDE4D	-0.45403	7.85E-38
FOXO6	1.116766	8.24E-38
C11orf82	-0.63421	9.28E-38
CXCL6	2.10452	9.29E-38
BAI2	0.668692	9.53E-38

ETAA1	-0.67522	1.03E-37
ITGA2	1.363386	1.21E-37
MCL1	-0.36752	1.32E-37
MYEOV	1.35073	1.38E-37
OCLN	0.662656	1.61E-37
CAPN5	0.783111	1.78E-37
DLC1	4.158501	1.83E-37
TNFRSF12A	-0.45514	1.83E-37
KCNMB3	0.98259	1.98E-37
CDC42SE1	-0.45753	2.16E-37
IFNLR1	1.098945	2.17E-37
SYNJ1	-0.59544	2.48E-37
ABHD15	1.127989	2.53E-37
SRC	0.574608	2.58E-37
PXDC1	0.934056	2.71E-37
INTS6	-0.39963	3.20E-37
MAML1	-0.44063	3.50E-37
DBF4	-0.56471	3.50E-37
WDR82	-0.45619	3.53E-37
LDLRAP1	0.893041	3.54E-37
RRAD	2.535216	3.58E-37
GCC1	-0.70496	3.78E-37
RAF1	-0.51262	3.79E-37
GNE	1.400764	5.69E-37
FAM103A1	-0.58489	6.11E-37
ZNF184	0.636384	6.43E-37
ARPC5L	-0.50083	6.68E-37
SEC24A	-0.55533	8.11E-37
CDCA8	-0.48883	1.03E-36
PLXND1	0.481918	1.07E-36
JDP2	1.164271	1.10E-36
PDGFA	-0.68921	1.15E-36
TRIM15	0.871891	1.27E-36
SLCO4A1	0.516816	1.27E-36
UTP15	-0.51863	1.30E-36
NGRN	-0.45297	1.67E-36
EGR2	0.607369	2.40E-36
WTAP	-0.39413	2.50E-36
MALL	0.78389	2.54E-36
KCNS3	2.903071	2.64E-36
TRMT10C	-0.72709	2.67E-36
MISP	-0.39756	2.71E-36
LATS2	-0.50456	3.00E-36
PPP1R10	-0.43755	3.04E-36
PLAT	1.179116	3.25E-36
PPP2R5B	0.843102	3.35E-36
KMT2B	-0.5961	3.44E-36

RPRD2	-0.50519	3.61E-36
FZD7	1.808157	3.72E-36
SHB	-0.50631	4.45E-36
BCL9L	-0.3559	4.64E-36
SFRP4	3.485728	5.00E-36
MLKL	0.545826	5.03E-36
UBE2G1	-0.45719	5.05E-36
TRIM29	0.534794	5.29E-36
NCOA4	-0.36501	5.49E-36
LOC730101	0.827151	5.80E-36
KDM2A	-0.44511	6.00E-36
TINAGL1	0.593221	6.01E-36
SLC35E4	0.643695	6.56E-36
AKAP8L	-0.51933	6.94E-36
IL17RD	0.989126	7.37E-36
INTS5	-0.5168	7.47E-36
PAPOLG	-0.58781	8.51E-36
POM121C	-0.45345	9.41E-36
POM121	-0.48185	9.79E-36
FEM1A	-0.55142	9.80E-36
TMF1	-0.44563	1.09E-35
MPHOSPH10	-0.59458	1.10E-35
CDC42EP1	0.471415	1.21E-35
GPATCH3	-0.76021	1.33E-35
OTUD7B	-0.54446	1.45E-35
TTBK1	2.936459	1.50E-35
PPIL4	-0.4949	1.54E-35
APOLD1	1.461141	1.71E-35
MALT1	-0.48959	1.74E-35
HIST1H1C	0.683163	1.94E-35
SLC1A4	1.259945	1.99E-35
EPHB4	1.159036	2.11E-35
ZNF697	-0.49862	2.23E-35
WASL	-0.75184	2.41E-35
MEF2D	-0.61075	2.46E-35
EFNB2	-0.34478	2.78E-35
ADORA1	2.16436	3.01E-35
ADRA2C	0.91098	3.02E-35
BRIX1	-0.39192	3.33E-35
LEMD3	-0.64188	3.81E-35
DLX2	1.038836	4.05E-35
ASB8	-0.55669	4.23E-35
FAM43A	0.616031	4.27E-35
FAM84A	8.554732	4.34E-35
URB2	-0.74394	4.49E-35
CSRNP3	1.132697	4.86E-35
CALCB	5.934292	5.02E-35

BMP6	3.394123	5.02E-35
ZNF410	-0.49368	5.23E-35
H6PD	0.571631	6.69E-35
ETF1	-0.39492	7.03E-35
CYP26A1	2.325108	7.09E-35
ZNF324	-0.92327	7.41E-35
GJB3	1.307089	7.65E-35
PRCC	-0.48842	9.07E-35
WDR74	-0.54249	9.20E-35
FZD2	2.834122	1.11E-34
WDR20	-0.63313	1.15E-34
GSN	0.848619	1.17E-34
VAMP3	-0.52451	1.17E-34
KDM6A	-0.44879	1.22E-34
MANSC1	0.850666	1.22E-34
SLC27A2	0.742662	1.27E-34
ZNF114	2.315219	1.31E-34
YTHDF3	-0.41912	1.46E-34
G0S2	2.395228	1.59E-34
CGN	0.559853	1.68E-34
UHMK1	-0.43375	1.72E-34
PRPF38B	-0.45753	1.83E-34
HERC6	0.874811	2.00E-34
SRPR	-0.36956	2.34E-34
TNFSF15	1.561231	2.47E-34
EXOC3	-0.4591	2.83E-34
GJA3	2.431466	3.23E-34
SLC2A10	1.878832	3.28E-34
DHX8	-0.40518	4.03E-34
PPARGC1A	2.265321	4.60E-34
IGHMBP2	-0.50111	5.01E-34
MAB21L3	0.716989	5.07E-34
PTPN22	0.743016	5.62E-34
MX1	1.215588	5.63E-34
CPEB2	3.195078	6.21E-34
CEND1	3.751381	6.54E-34
SHH	3.214881	6.95E-34
NOM1	-0.41283	6.98E-34
GLIPR2	1.581608	7.66E-34
ARIH1	-0.4179	8.53E-34
TMEM221	3.114087	8.56E-34
CD274	2.794073	9.94E-34
CENPC	-0.53714	1.02E-33
BUD13	-0.50282	1.04E-33
APBB1	0.851263	1.09E-33
RPRM	8.444598	1.42E-33
THSD1	1.946311	1.43E-33

SCML2	0.807358	1.58E-33
USP38	-0.47046	1.92E-33
PLK3	-0.5455	1.92E-33
CANT1	-0.54673	2.12E-33
ARID4A	-0.41194	2.23E-33
NR2E1	2.021442	2.47E-33
PLAUR	1.207485	2.67E-33
HCN4	5.398803	2.72E-33
NR1D1	-0.7534	2.84E-33
TWF1	-0.45955	2.91E-33
CSNK1D	-0.33607	3.05E-33
TMEM9B	-0.52833	3.28E-33
MT1X	0.417408	3.28E-33
GTF2H1	-0.45335	3.31E-33
C1QTNF6	1.287933	3.36E-33
SART3	-0.36826	3.42E-33
WIPF3	1.700901	3.76E-33
PLEKHO2	0.552932	4.23E-33
MAD2L1BP	-0.57183	4.44E-33
HCCS	-0.43312	4.78E-33
CAB39	-0.42502	5.10E-33
MBLAC2	1.135424	5.22E-33
ST3GAL5	1.898464	5.60E-33
EPPK1	3.05236	5.73E-33
KCNH3	1.121281	6.03E-33
ALDH3B1	0.888201	6.07E-33
SLC30A1	-0.3666	6.08E-33
SMO	1.460246	6.47E-33
EPHA1	3.487117	6.99E-33
RBM22	-0.41299	7.35E-33
NEURL1B	1.296689	7.87E-33
MED17	-0.56665	8.00E-33
TSPAN15	0.498317	8.09E-33
MOB3A	0.779541	8.35E-33
CREB3L2	0.534491	8.59E-33
RIMKLA	1.333878	9.43E-33
OLFM1	3.322402	9.50E-33
ATP2A3	1.757927	1.02E-32
FGD4	0.747003	1.23E-32
ZBTB38	-0.39016	1.25E-32
MAFB	2.726404	1.30E-32
HPS6	-1.04297	1.34E-32
TRA2A	-0.45065	1.34E-32
GAB2	0.705408	1.43E-32
CBLN1	4.012257	1.51E-32
FGA	1.280034	1.54E-32
CACNG4	0.492612	1.54E-32

AFAP1L2	0.602149	1.57E-32
EPC1	-0.4067	1.57E-32
MXD1	-0.83912	1.60E-32
TUBB2A	0.90681	1.84E-32
UBE2Z	-0.41071	2.11E-32
MOSPD1	-0.44169	2.17E-32
DNAJA2	-0.41427	2.19E-32
C7orf43	-0.63086	2.27E-32
RABGGTB	-0.3933	2.40E-32
CYTH4	1.987002	2.45E-32
EFNB1	0.5782	2.48E-32
AMMECR1L	-0.45522	2.62E-32
FTSJ1	-0.34866	2.71E-32
BAG4	-0.59079	2.77E-32
SRSF6	-0.34594	3.02E-32
SRRM3	3.530597	3.17E-32
HSPA14	-0.40916	3.32E-32
QRICH1	-0.42946	3.32E-32
SDAD1	-0.40766	3.66E-32
IL4R	0.85562	3.75E-32
TMEM56	0.714083	4.18E-32
TMEM127	-0.53389	4.64E-32
WAC	-0.37117	4.77E-32
ZNF266	-0.61038	5.14E-32
PAX5	3.047329	5.43E-32
LINC00657	-0.44869	6.24E-32
TCHH	2.005866	6.37E-32
EIF2S1	-0.34561	6.40E-32
SCAF4	-0.37969	6.52E-32
FUBP1	-0.34765	6.52E-32
VMA21	-0.43474	6.98E-32
PPP2CA	-0.32902	7.84E-32
AHRR	-0.40506	7.84E-32
YTHDC1	-0.35647	8.47E-32
LAMC2	0.645519	8.60E-32
FHL1	2.52	8.96E-32
KCTD21	0.959663	1.29E-31
CCDC137	-0.45203	1.34E-31
GNL3	-0.37378	1.42E-31
FNBP4	-0.36822	1.42E-31
CAPRIN2	0.772894	1.43E-31
MTPAP	-0.4976	1.47E-31
YY1	-0.42173	1.47E-31
ZDHHC22	1.299336	1.51E-31
DACT1	3.618402	1.81E-31
MKRN1	-0.39281	1.88E-31
MIR22HG	-1.27714	2.03E-31

SRP68	-0.34196	2.03E-31
CLSTN3	0.593878	2.64E-31
FEN1	-0.4911	2.89E-31
MSX2	0.529061	2.92E-31
ZFAND5	-0.35675	3.22E-31
C1RL	0.658158	3.23E-31
SLC46A1	0.870261	3.29E-31
SOX7	1.967184	3.41E-31
MATN1-AS1	1.045939	3.99E-31
CRAMP1L	-0.61493	4.16E-31
TGS1	-0.44472	4.19E-31
MAP6	2.533799	4.57E-31
RBM24	2.80944	4.85E-31
BCL6	0.68319	6.03E-31
SIN3A	-0.67885	6.33E-31
INPP5J	1.031656	6.34E-31
ZFP36L2	0.562607	6.52E-31
C2orf49	-0.64316	6.68E-31
SNHG16	-0.51479	7.30E-31
TSR1	-0.39093	7.36E-31
TIAL1	-0.37389	7.43E-31
WSCD1	3.438958	7.75E-31
OLFML2A	1.337034	7.85E-31
KDM7A	-0.44204	8.14E-31
SRRM1	-0.42705	8.35E-31
SBNO1	-0.52287	8.53E-31
IL1RAP	1.330949	8.56E-31
ZNF488	1.044797	9.10E-31
RASSF7	-0.51388	9.47E-31
WNT5B	3.39631	1.08E-30
FZD9	1.748664	1.12E-30
POLR3C	-0.46203	1.17E-30
B3GNT9	0.871125	1.18E-30
HAGLR	2.925274	1.22E-30
SNX18	0.762659	1.56E-30
AP1G1	-0.39814	1.64E-30
C16orf54	0.881201	1.72E-30
PGM2L1	0.680875	1.76E-30
LINC00265	0.597502	1.79E-30
UBE2D3	-0.36885	1.90E-30
PHC2	-0.39761	1.94E-30
UTP3	-0.53263	2.28E-30
MEIS3	1.103634	2.30E-30
SLC30A7	-0.6642	2.30E-30
HJURP	-0.54416	2.84E-30
GPR114	0.746589	2.96E-30
CDON	1.237642	3.07E-30

NUP153	-0.40726	3.08E-30
ZNF581	-1.17271	3.13E-30
AHDC1	0.5428	3.25E-30
GPR176	3.438297	3.32E-30
GJC1	1.380997	3.67E-30
ZNF654	-0.54357	5.01E-30
SIRT7	-0.51516	5.10E-30
ARID1A	-0.54634	5.13E-30
EFCAB14	-0.48646	5.26E-30
RPUSD2	-0.76137	5.27E-30
ANK1	1.143353	5.66E-30
ETS2	0.405945	5.75E-30
RTN4R	1.278667	6.21E-30
UTP14A	-0.33982	6.21E-30
ZRANB1	-0.46731	7.41E-30
SDC1	0.504884	8.01E-30
SLC7A6OS	-0.40715	8.26E-30
UBOX5	-0.69508	8.65E-30
SPSB1	0.646138	9.25E-30
SLC25A30	0.934297	9.51E-30
AKIRIN2	-0.44349	9.75E-30
CCDC136	1.037695	9.75E-30
DZIP1L	1.070864	1.01E-29
PHLDA3	1.459148	1.16E-29
ISM2	4.25975	1.17E-29
CASP2	-0.35989	1.23E-29
DGAT2	0.847469	1.26E-29
ACHE	3.267661	1.35E-29
MTERFD2	-0.58104	1.36E-29
ST14	0.846194	1.39E-29
PIK3CD	0.674781	1.49E-29
LHX9	2.539111	1.55E-29
SEMA4B	0.681788	1.63E-29
SH2D4A	0.771463	1.74E-29
LTV1	-0.40956	1.88E-29
VPS11	-0.45974	1.90E-29
EMR2	0.487917	2.14E-29
MCPH1	-0.59819	2.32E-29
HUNK	0.676964	2.51E-29
IREB2	-0.43169	2.56E-29
ATXN1L	-0.53151	2.69E-29
RBM7	-0.43456	2.86E-29
ESRP1	0.510178	3.44E-29
SDHAF2	-0.50733	3.48E-29
KDELR3	0.782291	3.50E-29
C11orf57	-0.54357	4.13E-29
ICAM1	1.317945	4.44E-29

MED15	-0.42735	4.79E-29
F5	1.201612	5.06E-29
ARF6	-0.34932	5.29E-29
ADAMTS9	0.826271	5.69E-29
H2AFX	-0.47428	6.08E-29
DTX4	3.093565	6.51E-29
RND3	-0.6619	6.66E-29
ZNF232	0.812063	7.53E-29
TRPC1	0.94353	7.76E-29
MORC4	0.509033	8.29E-29
DUSP16	0.519585	8.52E-29
ZDHHC7	-0.61648	8.60E-29
SEMA4F	0.803661	8.81E-29
PFKFB4	0.663165	8.95E-29
NDST1	0.768709	9.60E-29
NHLRC1	2.223097	1.00E-28
AK6	-0.57597	1.02E-28
PLLP	0.532623	1.06E-28
BCL10	-0.49519	1.11E-28
MFAP1	-0.43918	1.19E-28
LAT2	0.865368	1.27E-28
FBXO30	-0.45475	1.31E-28
SLC16A2	1.917787	1.32E-28
TRERF1	0.590998	1.35E-28
CD24	0.366002	1.43E-28
CBX2	0.607052	1.45E-28
THUMPD1	-0.4085	1.49E-28
PSAPL1	2.540254	1.59E-28
KITLG	1.18877	1.60E-28
SMIM3	1.178961	1.77E-28
GRWD1	-0.41254	1.90E-28
SRSF2	-0.32059	1.94E-28
MIXL1	3.157199	1.99E-28
LINS	-0.74036	2.12E-28
MTRF1L	-0.40223	2.20E-28
SLC9A7	0.623447	2.44E-28
GNAZ	1.341643	2.57E-28
MTF2	-0.57201	2.58E-28
GNG2	0.61971	2.60E-28
KDM5A	-0.39495	2.72E-28
IP6K2	-0.41613	2.79E-28
PDLIM5	-0.36076	2.81E-28
JMJD6	-0.43089	2.88E-28
BMP5	-0.61154	2.90E-28
LRRC10B	6.022919	2.99E-28
SURF6	-0.39096	3.05E-28
FRS2	-0.78868	3.15E-28

NPAT	-0.5619	3.17E-28
DCHS1	1.833809	3.22E-28
KIF23	-0.47051	3.26E-28
F2R	1.927027	3.43E-28
EPB41L4B	0.579732	3.53E-28
HES2	2.711369	4.24E-28
MCIDAS	1.534106	4.49E-28
PNMA2	2.101436	4.80E-28
TNFAIP2	0.633898	5.36E-28
RHOV	1.239688	5.92E-28
ITK	0.628145	6.81E-28
ZNF777	-0.35226	6.86E-28
HDX	0.82731	7.04E-28
RBM43	1.837652	7.04E-28
TGFB2	1.29522	7.14E-28
SH3TC2	1.041895	7.30E-28
PCSK9	0.70326	7.49E-28
GBX2	1.47371	8.34E-28
SLFN5	1.390018	8.40E-28
GORASP2	-0.3654	8.44E-28
NGDN	-0.44407	8.47E-28
WAPAL	-0.3592	8.83E-28
ENC1	0.511746	9.29E-28
VEZF1	-0.45664	9.95E-28
RANBP10	-0.32786	1.01E-27
TSPAN12	1.166725	1.03E-27
ACVR1C	2.131035	1.05E-27
ECT2	-0.38053	1.20E-27
ARL4D	0.990592	1.33E-27
ARHGEF37	2.629303	1.47E-27
ZUFSP	-0.53123	1.54E-27
RELA	-0.35547	1.60E-27
C10orf2	-0.60356	1.63E-27
VPS37B	-0.41472	1.63E-27
FLJ32255	1.609771	1.64E-27
NOP2	-0.33053	1.76E-27
TRIM2	0.951952	1.91E-27
PVRL1	0.442741	1.92E-27
TRIM16	1.130261	1.96E-27
PAG1	0.942982	2.06E-27
TRAPPC6B	-0.41843	2.06E-27
MFAP3L	2.836338	2.07E-27
DNTTIP2	-0.3806	2.49E-27
P2RY2	1.51521	2.60E-27
CREB5	0.801316	2.61E-27
RQCD1	-0.38171	2.74E-27
TM2D2	-0.54221	2.90E-27

GLI3	1.063538	3.25E-27
C9orf152	0.646771	3.36E-27
DOK4	0.543198	3.81E-27
SPEN	-0.43588	3.81E-27
MON1B	-0.36078	3.85E-27
NPY1R	6.254827	4.28E-27
ATF7IP	0.328366	4.80E-27
KIN	-0.56646	4.90E-27
ANKRD28	-0.44305	5.20E-27
WBP11	-0.33987	5.75E-27
MMP24	0.807875	6.31E-27
NFX1	-0.3812	6.57E-27
RNF182	2.758878	6.91E-27
TMEM170B	2.840175	6.92E-27
USP51	2.052078	6.99E-27
HOXD13	3.179625	7.11E-27
OXR1	-0.37072	7.35E-27
PHLDB1	0.39907	7.36E-27
SLC35A2	-0.4426	7.48E-27
SLC25A53	1.0888	7.73E-27
TNFAIP1	-0.47498	7.96E-27
SLC8B1	0.824483	8.35E-27
DHRS2	1.121481	8.57E-27
MEPCE	-0.40154	8.64E-27
WDR24	-0.77029	9.07E-27
TOPORS	-0.55886	9.43E-27
ERRFI1	-0.40014	9.50E-27
PRPF4	-0.40065	9.74E-27
DYNC1LI2	-0.32899	9.76E-27
SLC45A3	0.768046	1.01E-26
TMEM41A	-0.4983	1.03E-26
BEND3	0.997266	1.04E-26
MIEF1	-0.39402	1.07E-26
TRMT6	-0.36071	1.07E-26
TMOD2	1.525415	1.12E-26
CELF1	-0.31461	1.27E-26
KLHL18	-0.4482	1.27E-26
GAPVD1	-0.38792	1.33E-26
HCFC1	-0.43605	1.54E-26
CNOT3	-0.42059	1.57E-26
ABT1	-0.50536	1.62E-26
TMEM184C	-0.50726	1.64E-26
TRAF3IP2	0.558686	1.67E-26
STK35	-0.39371	1.68E-26
TRIM11	-0.38844	1.82E-26
HAS2	-0.6983	1.88E-26
CXorf57	0.556815	1.98E-26

RFWD3	-0.3419	2.06E-26
SHF	0.704699	2.09E-26
SFPQ	-0.29874	2.10E-26
STK40	-0.37989	2.23E-26
MRPS2	-0.41271	2.31E-26
RIMS3	1.610252	2.34E-26
RBM15	-0.62198	2.38E-26
AMD1	-0.3411	2.61E-26
ZBTB43	-0.85749	2.62E-26
FAM110A	0.666733	2.62E-26
RIOK2	-0.49713	2.64E-26
PHLDB3	0.638293	2.79E-26
PUM1	-0.38055	2.80E-26
PTPN14	-0.463	2.97E-26
GCC2	-0.37596	3.03E-26
CDK12	-0.39972	3.07E-26
MUL1	-0.44962	3.09E-26
KDM5B	0.494002	3.36E-26
CSNK1A1	-0.35252	3.37E-26
MED9	-0.93454	3.54E-26
SQLE	0.517668	3.68E-26
ZPR1	-0.31474	3.69E-26
CHST6	1.453928	3.83E-26
CEBPZ	-0.33701	4.01E-26
CRYBG3	0.602371	4.56E-26
IRS2	0.394628	4.58E-26
ATP8B2	1.653233	5.21E-26
PPP2R2D	-0.50483	5.27E-26
ELAVL2	0.637294	5.31E-26
RPP38	-0.5114	5.33E-26
NTF3	0.786707	5.43E-26
IGF2BP1	-0.42246	5.51E-26
TSPYL1	-0.43381	5.58E-26
LIN54	-0.59537	5.62E-26
LOC1019289	0.666226	5.64E-26
GPR132	0.952988	5.95E-26
ZNF532	0.51145	6.08E-26
C16orf91	-0.6857	6.15E-26
FNDC4	2.36778	6.16E-26
C2orf72	0.918383	6.45E-26
HAP1	1.486149	6.73E-26
NRG2	2.702784	6.75E-26
CHERP	-0.38897	6.99E-26
SLC10A3	-0.58365	7.70E-26
ARFGAP2	-0.33362	7.71E-26
ABCA3	1.303935	7.85E-26
TBX1	3.022054	8.03E-26

BRF2	-0.56683	8.04E-26
SFSWAP	-0.42992	8.11E-26
S1PR2	1.337123	8.19E-26
ARRDC3	-0.39135	8.30E-26
CDH3	4.108876	8.31E-26
TNFRSF1A	-0.65932	8.37E-26
EFNA2	1.474749	8.72E-26
ISLR2	4.271937	8.72E-26
ZNF12	-0.57165	8.82E-26
ZNF92	-0.4835	8.89E-26
TJP3	2.1702	8.93E-26
BFAR	-0.48009	9.27E-26
VDAC2	-0.31462	9.85E-26
LAPTM4A	-0.47261	1.08E-25
EIF4E3	1.853034	1.08E-25
SCYL1	-0.4533	1.08E-25
RC3H1	-0.64822	1.11E-25
DIDO1	-0.40227	1.12E-25
ARID4B	-0.46152	1.13E-25
ITGB7	0.624257	1.16E-25
HPSE	2.26749	1.17E-25
LOC1001290	0.393056	1.19E-25
CCNK	-0.3778	1.20E-25
ZBTB11	-0.42797	1.22E-25
ZNF267	-0.63755	1.32E-25
ABHD17B	-0.61433	1.36E-25
PPP4R4	2.610146	1.43E-25
RAB11FIP1	0.394857	1.47E-25
CCDC12	-0.54619	1.50E-25
ING2	-0.63154	1.51E-25
VSNL1	0.535698	1.74E-25
EDN2	2.497338	1.85E-25
PLEKHG2	0.428785	1.93E-25
IL21R	2.865036	2.01E-25
WT1	0.479546	2.02E-25
SREK1	-0.38786	2.05E-25
GPR56	0.295875	2.08E-25
GLCCI1	0.800612	2.10E-25
ACACB	0.704135	2.15E-25
AZIN1	-0.3165	2.40E-25
OGDHL	1.655844	2.45E-25
SRSF3	-0.32204	2.49E-25
FBXO11	-0.36472	3.05E-25
MSL3P1	0.68161	3.05E-25
NPEPPS	-0.32599	3.18E-25
SLC20A1	-0.30211	3.22E-25
KDM5C	-0.3433	3.44E-25

KCNQ1OT1	0.606328	3.63E-25
PLOD2	0.847602	3.74E-25
SEC24C	-0.33102	3.95E-25
APH1B	1.217488	4.16E-25
ZNF574	-0.69833	4.27E-25
RHOU	2.921696	4.27E-25
CERS2	-0.33718	4.81E-25
IRF5	0.873257	4.92E-25
C10orf118	-0.52619	4.94E-25
NAA60	-0.45038	5.05E-25
SEMA4D	1.584216	5.15E-25
IFRD1	-0.36073	5.36E-25
RFX2	0.73172	5.89E-25
SMIM15	-0.34542	5.94E-25
NGEF	0.693123	6.02E-25
CAMK2N2	2.062496	6.17E-25
FBXL3	-0.34788	6.35E-25
SELT	-0.43903	6.38E-25
PTGS1	1.896689	6.42E-25
PUM2	-0.34576	6.42E-25
DDAH1	0.652128	6.52E-25
CBR1	0.566062	6.64E-25
NKX3-1	0.826225	6.85E-25
DYRK1A	-0.51174	7.65E-25
SOX1	4.46999	7.81E-25
OXGR1	1.646277	7.81E-25
ICAM5	1.994045	7.85E-25
DLX1	1.128516	7.86E-25
WTIP	0.952714	8.38E-25
ONECUT2	0.984402	8.41E-25
NFATC1	1.335021	8.66E-25
MAFK	-0.55799	8.75E-25
TTC39A	1.447195	1.01E-24
COG3	-0.44776	1.01E-24
NR2F2	0.403325	1.02E-24
CRY2	-0.61035	1.04E-24
PPP6R1	-0.3452	1.04E-24
UBTF	-0.40263	1.21E-24
DDX52	-0.35235	1.22E-24
LRRN4	1.321089	1.25E-24
TRIM65	0.549467	1.25E-24
CLK3	-0.36517	1.26E-24
TRAM2	0.492181	1.34E-24
STC2	-0.36602	1.40E-24
WDR45B	-0.33394	1.44E-24
WFS1	-0.37128	1.53E-24
TMC5	0.909531	1.55E-24

STYK1	2.255241	1.61E-24
HNRNPDL	-0.27984	1.72E-24
LRFN1	1.860182	1.77E-24
SON	-0.32813	1.84E-24
CFL2	-0.7855	1.89E-24
HK1	0.391077	1.90E-24
KIAA0753	-0.41649	1.92E-24
L1CAM	0.372773	2.05E-24
NEXN	1.292916	2.10E-24
SOX14	4.421241	2.15E-24
FAM126A	0.760783	2.20E-24
RGS9	1.998462	2.39E-24
KLK6	0.447269	2.41E-24
ZNF441	-0.49451	2.42E-24
JHDM1D-AS1	0.816699	2.57E-24
CILP2	2.26086	2.59E-24
BLZF1	-0.49606	2.85E-24
NPTX2	3.150892	2.86E-24
YAP1	-0.28985	2.88E-24
TAF5	-0.76113	2.99E-24
PCYOX1L	0.901546	3.00E-24
TSPAN2	0.853471	3.09E-24
GLS2	0.7765	3.21E-24
LITAF	0.564019	3.36E-24
SOGA3	0.98493	3.37E-24
HOXB13	1.034017	3.55E-24
CISH	1.292575	3.75E-24
HOXC5	0.74676	3.75E-24
SPOCK2	1.919518	3.78E-24
C2orf81	1.021352	3.86E-24
PDE5A	3.190246	4.05E-24
SCN3B	1.365965	4.07E-24
ZNF778	-1.04017	4.07E-24
HOMER1	0.404485	4.28E-24
COPS2	-0.34359	4.40E-24
TSC22D2	-0.41923	4.58E-24
NKX2-1	0.831597	4.61E-24
PPP6C	-0.3563	4.86E-24
XYLT2	0.951144	4.93E-24
HNF4A	0.431937	5.16E-24
PRF1	0.511658	5.16E-24
FAM175B	-0.45632	5.31E-24
DNAJC30	-0.76943	5.78E-24
SORL1	0.457071	5.86E-24
IRX2	1.216395	5.94E-24
ZNF202	-0.47924	6.52E-24
RNF111	-0.46354	6.86E-24

TMEM87A	-0.39155	6.86E-24
EGR1	0.286142	7.52E-24
KIAA1586	-0.5262	7.86E-24
CACNA2D4	0.458322	8.01E-24
KCNJ2	2.014863	8.28E-24
DNMT3B	0.746646	8.33E-24
BMP8A	2.050226	8.49E-24
NOL11	-0.31368	8.50E-24
ATG101	-0.46961	8.51E-24
PALB2	-0.59132	8.51E-24
NUDT16P1	1.290814	9.06E-24
MRPL46	-0.50733	9.40E-24
TBC1D20	-0.44751	9.41E-24
FAM73B	-0.54205	9.60E-24
SLC16A1	-0.38845	9.84E-24
STEAP2	0.601844	1.03E-23
GLRX	0.709245	1.05E-23
FHDC1	0.538764	1.06E-23
PFN2	0.374182	1.07E-23
PHRF1	-0.3499	1.12E-23
CALM2	-0.33627	1.14E-23
MRFAP1	-0.32698	1.16E-23
PPP1R3B	0.741704	1.22E-23
TGFBR3	0.807551	1.25E-23
KIF21B	1.159366	1.28E-23
TNFAIP3	0.546424	1.28E-23
NKX3-2	2.661914	1.28E-23
SLC22A23	1.153946	1.32E-23
HTR7P1	1.170871	1.33E-23
KBTBD11	3.752785	1.41E-23
DPY19L2P2	1.125732	1.48E-23
TOMM70A	-0.3781	1.51E-23
ZNF764	-0.65971	1.53E-23
LOXL4	0.449275	1.54E-23
AFAP1	0.733924	1.57E-23
RICTOR	-0.40524	1.65E-23
PDK4	1.768761	1.76E-23
KLF9	2.237464	1.80E-23
HOXB9	0.388382	1.82E-23
HKDC1	0.830521	1.87E-23
ENO2	0.371922	1.90E-23
CHGA	4.150162	1.94E-23
ABCF3	-0.43394	1.97E-23
FAM220A	0.665451	2.01E-23
TRIM47	0.561974	2.01E-23
CCNL2	-0.57264	2.14E-23
TRAF4	-0.48505	2.19E-23

PLEKHH1	0.61725	2.30E-23
C16orf72	-0.43361	2.98E-23
GALNT6	0.563986	2.99E-23
RBM33	-0.41342	3.00E-23
RAD23B	-0.30444	3.00E-23
LSM14B	-0.37989	3.13E-23
B4GALNT1	1.31446	3.15E-23
PPP1R3D	0.908352	3.23E-23
SUPT6H	-0.31602	3.46E-23
ANKS4B	1.482371	3.55E-23
SHROOM3	0.509148	3.65E-23
UBE4A	-0.32007	3.70E-23
MACC1	-0.37123	3.74E-23
SLC7A6	-0.39711	3.87E-23
GABPB1	-0.49252	3.90E-23
ST3GAL2	0.432128	3.91E-23
POU3F1	2.564736	3.95E-23
FAM126B	-0.6927	3.98E-23
KLHDC10	-0.50695	4.02E-23
C14orf119	-0.38108	4.09E-23
FRMD4B	0.783396	4.16E-23
DCUN1D3	-0.62363	4.21E-23
CYP26B1	1.975625	4.27E-23
NCSTN	-0.33011	4.32E-23
RANBP6	-0.57766	4.77E-23
CNTNAP1	1.864448	4.84E-23
ABLIM1	0.480336	5.27E-23
RBBP5	-0.43923	5.28E-23
HR	0.864781	5.29E-23
RAP2C	-0.42687	5.71E-23
SAFB2	-0.35021	5.77E-23
SYP	0.703218	5.78E-23
NRBF2	-0.36154	5.86E-23
TAOK2	-0.38889	6.10E-23
SULF2	0.432397	6.20E-23
ZNF516	1.048755	6.49E-23
HGS	-0.35853	6.85E-23
JAKMIP2	1.709125	7.23E-23
TSPAN14	0.49711	7.23E-23
MED12L	1.443781	7.66E-23
STAU1	-0.29469	7.75E-23
TBCEL	-0.54904	7.95E-23
BET1L	-0.32692	8.08E-23
TBC1D8B	0.977685	8.36E-23
ESCO1	-0.38645	8.55E-23
ZNF24	-0.35139	8.55E-23
USP8	-0.38231	8.67E-23

FAM91A1	-0.35191	8.71E-23
LDB3	1.592517	9.10E-23
CHRND	0.751115	9.34E-23
FBXO5	-0.48494	9.70E-23
CDCP1	0.36119	1.03E-22
RNF34	-0.36432	1.10E-22
AK4	0.918346	1.11E-22
PPP1R9B	0.429669	1.12E-22
TMEM39A	-0.47475	1.14E-22
TLR6	2.512687	1.14E-22
ELK1	-0.34178	1.17E-22
TMOD1	1.28511	1.30E-22
H2AFJ	0.545738	1.30E-22
HOXA13	2.70656	1.32E-22
RGS2	0.695062	1.35E-22
DDX6	-0.39249	1.41E-22
SMTN	-0.37362	1.50E-22
CLP1	-0.97101	1.55E-22
ENPP1	0.735876	1.67E-22
VANGL2	1.656498	1.72E-22
SLC25A44	-0.4267	1.78E-22
MYADM	-0.35724	1.79E-22
CALHM1	2.61227	1.80E-22
RBM25	-0.34223	1.81E-22
UBALD1	-0.44156	1.83E-22
NOC3L	-0.35663	1.84E-22
HAUS8	-0.6069	2.12E-22
ACTG1	-0.29216	2.12E-22
CRIM1	0.741125	2.17E-22
RRN3	-0.33433	2.49E-22
HPS5	-0.41572	2.60E-22
GGA3	-0.53164	2.60E-22
OAS2	0.477238	2.62E-22
MYL9	0.42189	2.62E-22
ZNF579	-0.83327	2.74E-22
ATAD1	-0.333	2.75E-22
ALG2	-0.42866	2.96E-22
COMP	2.305538	3.08E-22
HIST1H2AG	1.405392	3.15E-22
FBRS	-0.44466	3.27E-22
ZNF322	-0.47365	3.27E-22
SLC1A5	-0.30344	3.36E-22
POLR2A	-0.30404	3.36E-22
RAB15	0.463153	3.40E-22
APBA1	2.614105	3.44E-22
TADA2B	-0.55441	3.64E-22
KANK1	0.474788	3.80E-22

PREX1	0.495152	3.89E-22
TMEM248	-0.32024	3.90E-22
MAP3K7	-0.37021	4.13E-22
EIF2AK3	-0.50227	4.17E-22
USP15	-0.34262	4.40E-22
TRPV3	1.399555	4.43E-22
CACNB3	0.836425	4.45E-22
BOD1	-0.67285	4.63E-22
C1orf27	-0.39997	4.76E-22
RBM34	-0.33863	4.88E-22
HIPK3	-0.47966	5.17E-22
TAF1D	-0.3277	5.18E-22
EIF3J	-0.30084	5.43E-22
ZNF506	-0.45612	5.55E-22
TMEM41B	-0.33618	5.58E-22
BMP8B	1.237544	5.64E-22
HIF1A	-0.31326	5.64E-22
ADRA1B	0.968996	5.91E-22
ADAMTS18	0.62402	5.95E-22
PRDM13	3.258101	6.25E-22
CEP68	0.485933	6.30E-22
PGBD5	3.071509	6.60E-22
SESN3	0.43098	6.62E-22
SLC30A4	1.797065	7.01E-22
CXCL16	2.026943	7.08E-22
HLA-E	0.420185	7.22E-22
CYTH2	-0.3867	7.26E-22
GPX8	0.965212	7.52E-22
C7orf31	0.745595	7.57E-22
TM2D3	-0.73348	7.64E-22
GPRC5A	0.818954	7.89E-22
RPS6KB1	-0.38494	8.06E-22
FAF2	-0.32896	8.34E-22
ARTN	1.236675	8.44E-22
SAP30BP	-0.37514	8.76E-22
ELF2	-0.73822	9.17E-22
SBK3	0.855798	9.36E-22
UGT8	0.956802	9.50E-22
PRSS23	-0.36599	9.81E-22
UGCG	-0.65821	1.01E-21
PCNXL3	-0.37596	1.03E-21
SUV39H1	-0.41552	1.06E-21
ATP6V1D	-0.38007	1.12E-21
PAFAH1B1	-0.28901	1.19E-21
ETNK1	-0.27451	1.20E-21
TTF1	-0.42561	1.20E-21
ZNF79	-0.7156	1.29E-21

ECD	-0.33239	1.32E-21
FAS	2.244031	1.35E-21
RABGEF1	-0.38298	1.40E-21
USP42	-0.50259	1.43E-21
LRRC20	0.530972	1.44E-21
MGAT2	-0.44865	1.46E-21
PLEKHM1	-0.45527	1.47E-21
TRIM6	2.021831	1.49E-21
STOM	1.094651	1.53E-21
CSNK2A2	-0.34486	1.54E-21
IL12A	2.592454	1.57E-21
ULBP1	0.816193	1.58E-21
DPF2	-0.34798	1.59E-21
DNAJC25	-0.5146	1.60E-21
KIF13B	0.379885	1.72E-21
ATOH8	2.139356	1.75E-21
ACBD3	-0.36403	1.76E-21
NEFH	3.036318	2.03E-21
NPR1	2.902284	2.05E-21
ARF4	-0.31177	2.06E-21
GID8	-0.36298	2.06E-21
CUL1	-0.33685	2.07E-21
AHNAK	0.497103	2.09E-21
IL27RA	0.663679	2.15E-21
KL	2.546912	2.20E-21
C19orf26	0.999813	2.23E-21
HSPA12A	0.40048	2.30E-21
RAB1A	-0.33933	2.36E-21
MAPK6	-0.38925	2.40E-21
FZR1	-0.42931	2.65E-21
MRAS	1.12884	2.75E-21
CPLX2	1.251703	2.75E-21
MBTPS1	-0.41989	2.81E-21
YWHAH	-0.29848	2.82E-21
CECR6	1.663406	2.86E-21
WDR43	-0.28737	2.96E-21
LOC1019284	2.062473	3.16E-21
PXYLP1	1.389943	3.22E-21
LPCAT2	0.613121	3.24E-21
FBXW7	-0.69007	3.34E-21
PUS3	-0.52467	3.34E-21
TFEB	2.37963	3.47E-21
GPER1	0.616611	3.51E-21
KCNF1	0.908502	3.65E-21
SLC39A14	0.29175	3.74E-21
RAB14	-0.34767	3.74E-21
RRAGD	1.140559	3.83E-21

FTSJ2	-0.38481	3.90E-21
RCE1	-0.41172	4.03E-21
TRMU	-0.44294	4.42E-21
KPNA4	-0.34487	4.54E-21
ST6GAL1	0.770262	4.61E-21
TNRC6C-AS1	1.594563	4.70E-21
GYLTL1B	0.557853	4.91E-21
RPRD1B	-0.36739	4.98E-21
UBE2J2	-0.37638	4.99E-21
ZBTB46	0.655188	5.18E-21
RAB2A	-0.42616	5.40E-21
AP1S3	0.478147	5.54E-21
ZNF462	2.505668	5.87E-21
RSRP1	-0.48285	6.07E-21
STRIP1	-0.45755	6.25E-21
CLIP2	0.334988	6.32E-21
PPP1R8	-0.34659	6.64E-21
PPM1B	-0.3818	6.79E-21
ZNF821	0.890195	7.01E-21
SRSF4	-0.2798	7.13E-21
OSER1	-0.40486	7.14E-21
MAPKBP1	0.383379	7.35E-21
POLR3G	0.664591	7.49E-21
SENP2	-0.34289	7.52E-21
HOXB3	-0.47062	7.55E-21
ANXA2R	1.536282	7.57E-21
PTPRF	0.465389	7.84E-21
ABCF1	-0.27999	7.88E-21
SAR1A	-0.31682	7.88E-21
PEAR1	1.108012	8.20E-21
SOBP	0.784349	8.47E-21
CALHM3	0.802813	8.54E-21
PTGER4	1.385932	8.67E-21
CWC22	-0.33826	9.07E-21
ARHGEF28	0.479685	9.11E-21
MTSS1	1.46313	9.17E-21
NLRC5	1.083271	9.43E-21
SPRED3	0.906515	9.54E-21
TRPV4	0.749702	9.62E-21
GPATCH1	-0.49985	1.03E-20
IST1	-0.2523	1.07E-20
PIAS4	-0.54251	1.12E-20
GJB2	2.026813	1.13E-20
RPP14	-0.44009	1.18E-20
TBC1D10B	-0.3838	1.20E-20
DUS2	-0.52221	1.21E-20
YIPF5	-0.42378	1.21E-20

FGD5	0.386254	1.22E-20
FAM222A	0.437901	1.23E-20
PRRT3	1.294893	1.30E-20
PKDCC	3.227336	1.30E-20
SOCS3	0.766777	1.31E-20
THBD	2.694949	1.36E-20
3-Sep	1.080156	1.38E-20
HIVEP3	0.720583	1.39E-20
EIF4G2	-0.25854	1.45E-20
CHODL	1.201176	1.49E-20
MIR4697HG	1.950224	1.71E-20
UBN2	-0.45955	1.88E-20
BVES	2.929042	1.89E-20
DLK2	1.476681	1.93E-20
GPR63	0.82619	1.97E-20
PRDM12	3.215652	2.00E-20
ZNF622	-0.34433	2.06E-20
CCDC94	-0.52636	2.07E-20
GPR124	1.953175	2.15E-20
FAM115A	0.326382	2.15E-20
SQSTM1	-0.29291	2.16E-20
BCL11A	1.286692	2.19E-20
SCARF2	1.254253	2.27E-20
MYCL	1.61549	2.27E-20
NIFK	-0.2942	2.32E-20
LURAP1L	2.05842	2.34E-20
CASC10	0.993057	2.39E-20
PPP1R37	-0.42649	2.41E-20
CYFIP2	0.388061	2.44E-20
LSM12	-0.34862	2.47E-20
SCNN1A	0.39964	2.61E-20
ZBTB49	-0.56822	2.77E-20
IL6R	0.827229	2.84E-20
ZHX1	-0.38154	2.87E-20
MAPK8IP1	0.888711	3.03E-20
PSME3	-0.30514	3.09E-20
TMEM136	0.511208	3.19E-20
FAM174B	0.564351	3.19E-20
NRXN2	3.352466	3.21E-20
TBRG4	-0.30015	3.30E-20
GPR126	1.15565	3.32E-20
NYNRIN	2.434259	3.40E-20
SEC23IP	-0.32943	3.41E-20
ATP6V1A	-0.33078	3.44E-20
AKNAD1	1.177978	3.52E-20
CALB2	3.215386	3.76E-20
CASP3	-0.34314	4.06E-20

KIAA1377	0.679296	4.20E-20
HOXC13	0.479214	4.25E-20
LRRC8B	0.541736	4.32E-20
GTF2B	-0.40037	4.33E-20
PTPN13	0.953792	4.34E-20
DHX38	-0.3212	4.46E-20
SRSF10	-0.30795	4.60E-20
ZNF195	-0.3957	4.60E-20
MAPRE3	0.806434	4.60E-20
OSGIN1	0.615571	4.65E-20
ARMC5	-0.40207	4.81E-20
FOXA2	-0.31937	4.84E-20
UMPS	-0.42565	5.04E-20
PRKCQ	1.407751	5.05E-20
PROSER2	0.597312	5.59E-20
STARD7	-0.29277	5.64E-20
RBM11	2.468924	5.67E-20
SCAF8	-0.29483	5.71E-20
PI4KB	-0.30766	5.79E-20
MMADHC	-0.33135	5.83E-20
DSC2	2.00126	6.02E-20
ZBTB14	0.61304	6.18E-20
KIF18A	-0.33432	6.27E-20
PTGER4P2-CI	1.00552	6.27E-20
XAB2	-0.36416	6.34E-20
DUSP15	2.691685	6.42E-20
CD58	0.671473	6.52E-20
SPSB4	3.243006	6.54E-20
GOSR1	-0.36393	6.86E-20
SLC44A1	0.474265	6.95E-20
DGKA	1.113653	7.18E-20
RAB18	-0.34762	7.34E-20
HNRNPH3	-0.26413	7.43E-20
MIDN	-0.33109	7.49E-20
VASH1	1.35031	7.49E-20
C6orf47	-0.68643	7.54E-20
TLL2	2.907552	7.54E-20
FAM168A	0.345402	8.19E-20
SCG2	0.406796	8.23E-20
ZNF606	0.809016	8.59E-20
KLHL5	-0.37687	8.82E-20
FAM76B	-0.37786	9.22E-20
ANKRD33B	1.801422	9.53E-20
RMDN3	-0.42687	9.68E-20
CXorf40A	-0.64364	9.70E-20
TICAM1	0.643023	1.05E-19
MED21	-0.41052	1.15E-19

CLPX	-0.44675	1.15E-19
SLU7	-0.34973	1.20E-19
RNF213	0.491716	1.23E-19
LEPREL4	0.567708	1.23E-19
GALNT12	0.884728	1.25E-19
LETM2	0.576676	1.28E-19
SLC39A9	-0.38284	1.29E-19
MLH3	0.418735	1.41E-19
CDKL2	2.064417	1.45E-19
HLX	2.752923	1.49E-19
TRIM9	0.80832	1.51E-19
KCMF1	-0.36367	1.53E-19
TRIM71	1.908586	1.55E-19
MYH10	0.275552	1.58E-19
MRGPRF	1.660237	1.59E-19
KIF3A	0.514346	1.76E-19
HIPK1	-0.39485	1.82E-19
MAEA	-0.38342	1.87E-19
ANKS6	0.403406	1.89E-19
RAB36	0.69988	1.89E-19
HCFC2	-0.54671	1.90E-19
PPP2R2C	-0.43456	1.93E-19
MRPS18B	-0.39447	1.95E-19
ATG9A	-0.49239	1.97E-19
INPP5F	0.438771	2.07E-19
CNOT6L	-0.42344	2.14E-19
PAFAH1B2	-0.32999	2.18E-19
GPATCH8	-0.32684	2.29E-19
MRC2	2.023703	2.30E-19
TDG	-0.35047	2.35E-19
SMIM10	0.647144	2.35E-19
SPATA5L1	-0.38156	2.38E-19
ADM2	1.602263	2.46E-19
AIF1L	0.420692	2.47E-19
ZFP91	-0.39903	2.49E-19
SEC14L2	0.677599	2.62E-19
SP3	-0.29181	2.65E-19
GTF2E1	-0.49981	2.85E-19
ATF2	-0.38497	2.86E-19
PLCH1	0.732312	2.94E-19
GAS2L1	0.679669	2.97E-19
TLK2	-0.29168	3.04E-19
FAM134B	1.699119	3.13E-19
GTF2F1	-0.26445	3.22E-19
ATP6V0E2-AS	1.160278	3.34E-19
COIL	-0.43668	3.44E-19
EDC4	-0.2796	3.46E-19

GBP2	1.880179	3.61E-19
PCYT1B	0.849014	3.63E-19
C3orf80	2.560611	3.64E-19
GRPEL2	-0.45578	3.70E-19
DHFRL1	0.678038	3.75E-19
CORO1A	0.81096	3.79E-19
PWWP2B	1.246098	4.05E-19
ZBTB47	1.129378	4.15E-19
RND1	0.535156	4.16E-19
LRP11	0.474258	4.17E-19
KRT81	0.534737	4.17E-19
CLGN	1.595441	4.17E-19
USP36	-0.389	4.21E-19
FAM160A2	-0.39274	4.25E-19
SH3BP5	0.49622	4.26E-19
THY1	1.741092	4.27E-19
GIGYF2	-0.35027	4.37E-19
ZHX2	0.567881	4.45E-19
FOSB	-0.35194	4.60E-19
EVA1A	1.075825	4.69E-19
ITPRIPL1	0.88258	4.71E-19
ARHGEF7	-0.31487	4.83E-19
SMAD7	-0.74549	4.83E-19
BCOR	0.321742	4.83E-19
ATG2A	-0.39034	4.84E-19
EIF3A	-0.29835	4.90E-19
HS1BP3	0.678765	4.90E-19
KAT5	-0.2986	4.94E-19
HEY1	1.595775	4.97E-19
MUC13	0.732041	5.15E-19
ZNF469	1.110303	5.37E-19
SERPINA1	0.61532	5.39E-19
LSG1	-0.27963	5.40E-19
GNAI1	-0.42602	5.68E-19
ULBP2	0.714931	5.71E-19
SLC7A4	1.095726	5.73E-19
C1orf226	0.647024	5.78E-19
SLC30A5	-0.33021	5.84E-19
VWF	1.674086	6.06E-19
SATB2	0.73374	6.12E-19
LRRC8A	-0.291	6.13E-19
DGCR14	-0.4741	6.32E-19
TAGLN3	1.981721	6.46E-19
ZBTB4	-0.33976	6.59E-19
MTUS1	1.286521	6.71E-19
BRPF3	-0.2949	7.06E-19
FBXO42	-0.45292	7.13E-19

FBXO41	0.59172	7.35E-19
TESK1	-0.63133	7.37E-19
ARMCX5	-0.39214	7.73E-19
TTC3P1	1.364774	7.85E-19
HIST2H2BE	0.400182	7.86E-19
TOB2P1	1.343616	7.92E-19
GSK3A	-0.33068	8.13E-19
KAT7	-0.36128	8.31E-19
ZDHHC1	1.13057	8.40E-19
LAPTM5	0.765267	8.49E-19
HOXB8	-0.54748	8.60E-19
ARL5B	-0.45332	8.71E-19
CDC37L1	-0.58813	8.75E-19
DAXX	-0.39778	8.92E-19
PRPF3	-0.32228	8.93E-19
C11orf30	-0.44125	9.04E-19
TGFBR2	-0.32845	9.71E-19
ZFP3	0.910846	1.07E-18
RNF25	-0.44053	1.07E-18
LINC01137	1.601213	1.08E-18
SF3B1	-0.25107	1.13E-18
RXRA	0.548705	1.14E-18
PTGFRN	0.581976	1.20E-18
SSTR1	1.312247	1.21E-18
MFN2	-0.27552	1.22E-18
AMPD3	0.55424	1.25E-18
EFNA4	2.57532	1.31E-18
PPM1D	-0.40087	1.35E-18
HMOX1	0.951595	1.36E-18
RNMT	-0.37483	1.39E-18
PLEKHA3	-0.54331	1.46E-18
HID1	0.527459	1.50E-18
MARK3	-0.32954	1.51E-18
NRN1	1.894431	1.51E-18
C1orf116	-0.36284	1.55E-18
FAM160B1	-0.69495	1.59E-18
PIGA	-0.40924	1.64E-18
ADNP	-0.28563	1.66E-18
ZMYND8	-0.2663	1.73E-18
ZNF354A	0.803851	1.76E-18
DPYSL2	0.414326	1.83E-18
GIPR	2.091736	1.91E-18
PTP4A1	-0.27873	1.95E-18
VASP	-0.2695	1.98E-18
GPRIN1	0.428574	2.00E-18
TEAD3	0.577421	2.03E-18
LPAR5	0.98885	2.04E-18

CDK7	-0.37442	2.05E-18
DDX42	-0.24047	2.08E-18
USP22	-0.27028	2.14E-18
THEM6	0.466377	2.19E-18
NXPE3	0.853665	2.28E-18
NBR1	-0.31774	2.29E-18
SURF4	-0.2751	2.35E-18
DDX23	-0.26548	2.36E-18
PRSS35	2.892203	2.41E-18
ADPRH	0.95807	2.55E-18
TCFL5	0.582412	2.56E-18
CCNJ	-0.61261	2.67E-18
ADAMTSL5	0.640777	2.86E-18
G2E3	-0.40468	2.86E-18
HSPG2	0.334003	2.91E-18
C1QTNF1	0.626898	2.92E-18
TACO1	-0.38803	2.92E-18
MED8	-0.43541	2.95E-18
PDP2	0.431913	3.13E-18
P4HA1	0.337763	3.22E-18
CEP76	-0.52491	3.35E-18
KLF4	0.707719	3.43E-18
NRBP1	-0.29791	3.48E-18
EIF2B2	-0.29858	3.51E-18
PHF12	-0.40283	3.74E-18
TNKS1BP1	0.302686	3.78E-18
NCR3LG1	0.753519	4.04E-18
MAK16	-0.39797	4.06E-18
HUS1	-0.31785	4.07E-18
BNIP2	-0.4465	4.09E-18
SDF2	-0.48536	4.14E-18
SOGA1	0.573916	4.15E-18
CCT6P3	-0.454	4.22E-18
ERBB3	0.334739	4.27E-18
CDYL	0.432235	4.28E-18
ZNF641	0.444994	4.28E-18
CTSL	0.680703	4.30E-18
NFAT5	-0.46163	4.45E-18
STARD8	1.817138	4.54E-18
C2CD2L	-0.39333	4.55E-18
TGIF1	-0.5356	4.66E-18
ABTB2	0.376996	4.93E-18
FUT4	-0.4291	4.94E-18
WWTR1	0.452853	4.98E-18
DCLK1	1.244452	4.99E-18
GLI2	2.35425	5.31E-18
RARG	0.579736	5.45E-18

CD83	0.856372	5.57E-18
LGR4	0.625637	5.67E-18
HNRNPUL1	-0.22329	5.72E-18
ADPRHL2	-0.37958	5.73E-18
ACOT4	1.093926	5.83E-18
ZC3H3	-0.52386	5.83E-18
ZNF281	-0.33227	5.90E-18
CDYL2	0.797062	5.98E-18
FBXW11	-0.34706	6.02E-18
TNFRSF11B	1.901392	6.02E-18
TIMM23B	-0.3953	6.04E-18
TSC22D3	0.641262	6.08E-18
LOC284023	0.73136	6.16E-18
MTERF	-0.37919	6.58E-18
CCNG1	-0.36468	6.76E-18
CRMP1	0.43453	6.83E-18
LRP1	0.831903	6.95E-18
PTPN1	-0.26494	7.05E-18
BLACAT1	1.296651	7.55E-18
AP3M1	-0.26861	7.65E-18
REXO1	-0.42712	7.69E-18
MAST1	1.046696	7.75E-18
PDHX	-0.32828	8.07E-18
MPZL1	0.296553	8.10E-18
TACC2	0.349536	8.29E-18
KCNC3	2.814989	8.32E-18
DLG5-AS1	1.395825	8.43E-18
MICA	0.572704	8.62E-18
ZC3H6	0.61974	8.88E-18
PPM1K	0.770282	8.89E-18
RPP25	0.531113	9.09E-18
TIMM22	-0.33806	9.14E-18
CD164	-0.32857	9.14E-18
SEMA3A	-0.29898	9.31E-18
FAM171A2	0.831231	9.52E-18
REST	-0.38097	9.58E-18
SLC38A4	1.210758	9.80E-18
CPSF7	-0.24186	1.02E-17
FAM193A	-0.31491	1.06E-17
HSPA4	-0.25766	1.07E-17
FRAS1	0.449975	1.08E-17
FBXO33	-0.47303	1.11E-17
DSEL	1.184321	1.11E-17
KIAA1522	0.391772	1.11E-17
TSLP	2.537627	1.14E-17
SEMA6A	0.67873	1.17E-17
DNAJC2	-0.30297	1.18E-17

CPNE2	0.462706	1.18E-17
ANKLE2	-0.37688	1.22E-17
TMEM98	0.963404	1.23E-17
ZC3H12C	0.451703	1.23E-17
NOB1	-0.24081	1.30E-17
USP6NL	-0.53475	1.30E-17
SMIM24	2.556496	1.31E-17
BTBD3	0.301256	1.42E-17
NABP1	-0.64614	1.46E-17
AGBL2	1.046203	1.48E-17
ZNF280C	0.325889	1.52E-17
CYP1B1	1.059321	1.52E-17
DOCK3	0.890892	1.53E-17
AKT1S1	-0.55045	1.53E-17
VCP	-0.25435	1.63E-17
NAA50	-0.26148	1.63E-17
4-Sep	1.345843	1.64E-17
ARHGAP18	-0.27042	1.66E-17
TRIM23	-0.52506	1.72E-17
MLF1	0.982065	1.82E-17
ZNF800	-0.49828	1.84E-17
LEPREL1	0.911034	1.86E-17
AMN	2.169609	1.86E-17
CHAMP1	-0.28616	1.88E-17
PDGFRB	1.499995	1.93E-17
GRPEL1	-0.32039	2.00E-17
FBXO38	-0.37671	2.02E-17
TRIM7	1.210528	2.11E-17
RBAK	-0.41097	2.13E-17
DPM1	-0.27706	2.15E-17
ARVCF	0.656341	2.22E-17
OSBPL6	1.110621	2.22E-17
SHISA9	0.308957	2.25E-17
ENPP5	2.59631	2.30E-17
CCDC97	-0.31666	2.33E-17
C14orf132	1.136583	2.33E-17
ITCH	-0.26307	2.39E-17
EPC2	-0.35076	2.44E-17
SHISA2	2.977977	2.48E-17
TSN	-0.3003	2.57E-17
MAP10	2.488348	2.64E-17
ATP2A2	-0.2792	2.66E-17
RPS6KB2	-0.31597	2.69E-17
ENDOD1	0.438742	2.70E-17
IGFBPL1	0.691923	2.72E-17
VPS4A	-0.24259	2.84E-17
KIAA0040	0.524139	2.88E-17

NKIRAS2	-0.34387	2.89E-17
CRISPLD2	1.113341	3.02E-17
SAMD1	0.575511	3.05E-17
RHOBTB1	0.856774	3.11E-17
JUP	0.277769	3.22E-17
TNFAIP8	1.018189	3.30E-17
PHC1	0.417472	3.37E-17
EXOSC6	-0.36291	3.38E-17
CDC42BPG	0.715694	3.38E-17
MAP3K12	1.290181	3.41E-17
ARHGAP11A	-0.31777	3.45E-17
ZNF709	1.068729	3.64E-17
TCEAL3	0.773258	3.69E-17
ZNF540	1.628337	3.74E-17
UBAP2L	-0.27443	3.74E-17
SNX16	0.737921	3.90E-17
POLQ	-0.48359	3.92E-17
TPST1	0.476747	3.93E-17
CTDSP1	-0.35254	3.94E-17
GUCD1	-0.36879	4.12E-17
TRIM37	-0.38465	4.35E-17
SEC16A	-0.27445	4.44E-17
HEXIM1	-0.27708	4.61E-17
CENPQ	-0.80942	4.65E-17
GTF2A1	-0.36998	4.69E-17
LOC1001312	0.539545	4.69E-17
C8orf33	-0.2868	4.87E-17
LRP12	0.732978	4.95E-17
KIF3C	0.353661	4.96E-17
TRIM46	1.918877	4.96E-17
CDK9	-0.3756	5.00E-17
RUNX2	1.033551	5.01E-17
MYSM1	-0.31763	5.10E-17
IFNAR2	0.658843	5.24E-17
RECK	1.070445	5.31E-17
SOX5	0.618343	5.44E-17
NCMAP	2.08042	5.46E-17
IL6ST	-0.31328	5.48E-17
CMTM4	0.551844	5.50E-17
NRAS	-0.30937	5.69E-17
MAPK8IP2	2.166961	5.87E-17
SNX33	0.712323	5.87E-17
SPN	0.460869	6.08E-17
PRR18	1.911289	6.08E-17
ZNF485	0.878679	6.19E-17
CHPF2	-0.39552	6.59E-17
MRPL49	-0.32707	6.62E-17

USP10	-0.27615	6.64E-17
TBC1D1	0.38228	6.87E-17
SMEK2	-0.34697	6.92E-17
ITPR1	0.77925	7.33E-17
ADCY1	2.064045	7.39E-17
TRIP11	-0.38761	7.44E-17
BRSK1	1.141657	7.57E-17
IKBKG	-0.38169	7.59E-17
HS3ST3B1	1.603244	7.72E-17
SLC30A6	-0.37138	8.03E-17
GOLPH3	-0.27847	8.10E-17
ARHGEF10	0.479963	8.11E-17
CYB5D1	-0.46048	8.11E-17
FZD6	0.414806	8.30E-17
JPH3	1.279543	8.53E-17
FAM13C	0.82618	8.63E-17
NSUN2	-0.23254	8.65E-17
BRD4	-0.35391	8.75E-17
INO80D	-0.43181	9.04E-17
TMEM200B	2.554505	9.20E-17
RAB10	-0.26103	9.65E-17
FLJ25758	2.988309	9.83E-17
CAPRIN1	-0.23545	9.85E-17
RAB22A	-0.41123	1.00E-16
7-Mar	-0.26472	1.01E-16
CACTIN	-0.3678	1.04E-16
TTC30B	1.236653	1.05E-16
POLR2D	-0.33776	1.09E-16
PARP12	0.644221	1.09E-16
ATP8B3	0.888911	1.16E-16
KRT17	0.752997	1.20E-16
MAP4K5	-0.29598	1.20E-16
KLHL20	-0.4796	1.22E-16
CCDC69	1.150176	1.22E-16
CREB3L1	2.296996	1.23E-16
TOX4	-0.29188	1.23E-16
MRPL50	-0.49587	1.23E-16
IL22RA1	1.444702	1.23E-16
FAM132B	0.926533	1.24E-16
ID1	-0.6001	1.25E-16
ARG2	0.469733	1.29E-16
SACM1L	-0.39754	1.29E-16
ARFGAP1	-0.2986	1.32E-16
HIST1H1E	1.161177	1.41E-16
TM9SF3	-0.27917	1.42E-16
NPL	0.934424	1.44E-16
ZNF17	-0.72357	1.58E-16

MAP3K1	-0.34231	1.60E-16
MGAT3	0.837763	1.61E-16
GPATCH11	-0.34324	1.64E-16
ZCCHC10	-0.44412	1.69E-16
C2orf47	-0.54305	1.69E-16
RNF26	-0.37454	1.70E-16
GPR162	1.785216	1.71E-16
CDIP1	1.346101	1.79E-16
SYNGR3	2.523736	1.79E-16
ZNF317	-0.31969	1.79E-16
SAP130	-0.3608	1.81E-16
TBKBP1	1.086427	1.83E-16
TBC1D23	-0.31661	1.85E-16
ALKBH1	-0.375	1.95E-16
VPS18	-0.4684	2.03E-16
PTPDC1	0.624108	2.04E-16
ZSWIM8	-0.31627	2.06E-16
SAFB	-0.26432	2.12E-16
UBALD2	-0.43922	2.30E-16
UBAP1	-0.30563	2.32E-16
ATP8B1	0.666746	2.35E-16
SCYL2	-0.30084	2.35E-16
PMM2	-0.29392	2.42E-16
FBXO31	-0.44159	2.47E-16
TIGD2	0.880248	2.53E-16
LRFN3	1.792989	2.56E-16
DSCAML1	1.676236	2.57E-16
NTRK2	0.644508	2.63E-16
ELK3	0.722801	2.67E-16
WBP1L	-0.43524	2.70E-16
CYP2R1	0.767668	2.74E-16
PTRH2	-0.51743	2.77E-16
CWC15	-0.40832	2.82E-16
OAS3	0.358557	2.88E-16
USP1	-0.28736	2.94E-16
IDI1	-0.34413	2.95E-16
TRAPPC10	-0.31893	2.97E-16
C1orf106	0.393518	3.02E-16
STX5	-0.42106	3.11E-16
LHX4	2.043437	3.21E-16
LOC113230	0.830315	3.22E-16
PKIG	0.45539	3.25E-16
PDCD7	-0.30521	3.25E-16
RNF122	1.470247	3.25E-16
ZKSCAN8	-0.28881	3.26E-16
SMPD3	1.482479	3.33E-16
TIAM2	1.255918	3.40E-16

ABCG4	2.57151	3.45E-16
LINC00701	0.986273	3.45E-16
CDC7	0.309982	3.46E-16
PCSK7	-0.29234	3.46E-16
MED20	-0.36699	3.46E-16
RAP1GAP	0.715634	3.46E-16
PSMG3-AS1	1.287115	3.50E-16
CCDC88C	0.355997	3.53E-16
SNX9	-0.27366	3.68E-16
MTO1	-0.39019	3.80E-16
DHCR7	0.319664	3.92E-16
ZNF783	0.46801	3.96E-16
6-Mar	-0.29102	3.99E-16
DLX4	1.267025	4.05E-16
CDV3	-0.26441	4.07E-16
NUP88	-0.2775	4.14E-16
METTL22	-0.52877	4.17E-16
HIST1H2BG	1.07042	4.28E-16
NFYA	-0.38358	4.36E-16
PAPSS2	2.156936	4.45E-16
FBXL12	-0.45231	4.50E-16
ZNF689	-0.42774	4.57E-16
IP6K1	-0.29014	4.69E-16
IQSEC2	1.673786	4.78E-16
SHOC2	-0.31988	4.78E-16
TGFB1I1	0.649542	4.78E-16
PKIA	0.764774	4.80E-16
TMEM203	-0.64574	4.82E-16
ZMYM3	0.281918	4.87E-16
MGC12916	1.813955	4.88E-16
RB1CC1	-0.34123	4.94E-16
HTATIP2	0.489777	4.99E-16
CDC23	-0.30587	4.99E-16
C5orf30	0.644848	5.04E-16
USF1	-0.43832	5.36E-16
PRSS16	0.613609	5.47E-16
CCNF	-0.43475	5.49E-16
MYPOP	0.869561	5.54E-16
GPRC5B	2.600124	5.54E-16
DERL2	-0.3782	5.57E-16
QSOX2	-0.28498	5.63E-16
KLF3	-0.35452	5.71E-16
VAPA	-0.26811	5.71E-16
EPB41L4A	1.810759	5.89E-16
FXR2	-0.30431	5.89E-16
UNC50	-0.41405	5.92E-16
BCAS4	0.516037	6.02E-16

ZFHX3	0.421859	6.12E-16
HNRNPU	-0.23109	6.20E-16
PRRG4	2.238091	6.25E-16
ZKSCAN5	-0.36226	6.47E-16
SPHK1	2.105798	6.57E-16
EMC7	-0.32767	6.58E-16
ATP10D	0.667148	6.74E-16
EMC8	-0.35019	6.77E-16
ZNF791	-0.60387	6.82E-16
FAM169A	0.340827	7.02E-16
NXPH3	1.356467	7.04E-16
ZNF385A	0.405073	7.38E-16
NIPA2	-0.33356	7.54E-16
TNS1	2.065032	7.56E-16
CTCF	-0.24747	7.79E-16
C15orf52	0.561089	7.88E-16
ATG13	-0.26914	7.95E-16
MAP3K11	-0.38332	8.25E-16
NOL8	-0.32695	8.27E-16
FEM1B	-0.31832	8.28E-16
MED1	-0.32455	8.28E-16
INHBB	-0.30004	8.31E-16
RAI14	0.39008	8.42E-16
DNAJC18	0.692524	8.53E-16
H1FX	0.35354	8.56E-16
MIER1	-0.28318	8.56E-16
GPC4	0.544125	8.77E-16
COLCA2	2.584904	8.87E-16
KIAA1244	0.552713	8.94E-16
RASSF3	0.381563	9.16E-16
MAK	1.41314	9.43E-16
PDZD4	1.462782	9.48E-16
FNBP1	0.593873	9.55E-16
MAN1C1	2.446893	9.56E-16
DAGLA	0.418239	1.03E-15
WIZ	-0.38398	1.06E-15
TUBA1C	-0.25904	1.06E-15
ATF7IP2	0.637606	1.10E-15
SUPT7L	-0.33986	1.11E-15
MED4	-0.42849	1.12E-15
SRCIN1	1.162526	1.15E-15
MAPK1IP1L	-0.30306	1.15E-15
METTL13	-0.43066	1.17E-15
MTMR14	-0.35254	1.19E-15
SUZ12	-0.28454	1.20E-15
MORF4L1	-0.27103	1.20E-15
RUNX1T1	1.368892	1.23E-15

C2orf15	0.831101	1.25E-15
KPNA6	-0.31047	1.26E-15
PEG10	0.301891	1.29E-15
LINC00883	1.511062	1.32E-15
GFM2	-0.28818	1.33E-15
LPHN2	0.938726	1.36E-15
TSSC4	-0.46484	1.37E-15
BRD9	-0.26449	1.44E-15
HBS1L	-0.28645	1.45E-15
RNMTL1	-0.38995	1.46E-15
DUSP10	1.058647	1.48E-15
PAPD4	-0.33058	1.53E-15
ZSCAN25	-0.36638	1.54E-15
ARL6IP1	-0.27161	1.56E-15
CUL3	-0.33629	1.62E-15
MOCS3	-0.53359	1.62E-15
PARP8	0.371661	1.63E-15
RRP12	-0.26488	1.64E-15
EMC1	-0.29206	1.65E-15
SCG3	0.882204	1.70E-15
FANCF	0.509073	1.79E-15
TWISTNB	-0.31099	1.80E-15
NOL6	-0.271	1.80E-15
XBP1	-0.36092	1.82E-15
ZNF384	-0.27339	1.92E-15
COPB2	-0.23775	1.93E-15
LOC729732	0.833995	1.94E-15
PLEK2	0.448963	1.94E-15
ARF1	-0.22415	1.96E-15
CYTH3	0.350798	1.97E-15
IRF2BPL	0.436338	1.97E-15
DCTN4	-0.3326	1.98E-15
SLC35F2	0.305322	1.98E-15
ZNF200	-0.52889	2.03E-15
EDARADD	1.10498	2.03E-15
SENP5	-0.3188	2.05E-15
ATXN7L3B	-0.34506	2.06E-15
ZNF136	-0.60564	2.06E-15
MED13	-0.27294	2.19E-15
ADAM19	0.287577	2.23E-15
GPR183	-0.60051	2.24E-15
GPRC5C	-0.36301	2.27E-15
FAM46B	0.913405	2.28E-15
TET1	0.757719	2.30E-15
IGSF5	1.621821	2.30E-15
ZNF425	0.414975	2.34E-15
ZNF212	-0.41226	2.38E-15

ACTB	-0.19315	2.46E-15
FERMT1	0.290698	2.50E-15
KIAA0947	-0.34814	2.50E-15
WWC2-AS2	2.0369	2.52E-15
USP35	0.909392	2.53E-15
SLC41A1	0.374549	2.55E-15
SMARCD3	1.217631	2.58E-15
TRMT44	-0.52574	2.62E-15
SUPV3L1	-0.39785	2.67E-15
LINC00052	2.513875	2.70E-15
PDLIM4	0.603548	2.70E-15
AXL	-0.25059	2.71E-15
TTC25	0.818151	2.80E-15
PAIP2	-0.27946	2.82E-15
SF3B3	-0.22811	2.94E-15
ATP6V0C	-0.32087	2.98E-15
CCNG2	-0.86718	2.98E-15
DDX47	-0.25399	3.02E-15
TOMM20	-0.24915	3.13E-15
SYNC	1.478529	3.15E-15
ZFP36	-0.48949	3.16E-15
MYL12B	-0.21012	3.18E-15
ITPKA	0.67866	3.29E-15
TNPO3	-0.34005	3.30E-15
ACSL4	0.390577	3.30E-15
ZNF398	-0.40472	3.46E-15
PPP4C	-0.32069	3.62E-15
ASB7	-0.47829	3.66E-15
ANKRD40	-0.37333	3.80E-15
KHDRBS3	0.938492	3.90E-15
RNF40	-0.24516	3.95E-15
B3GNT7	2.017853	3.98E-15
MARS2	-0.51118	4.01E-15
POLM	0.432157	4.03E-15
ZNF770	-0.32364	4.07E-15
KIAA1467	0.522579	4.08E-15
RPS6KL1	0.494443	4.08E-15
ZBED8	0.848651	4.08E-15
ADSS	-0.29581	4.12E-15
PIGS	-0.35573	4.14E-15
KANK2	0.327238	4.14E-15
MID1	-0.25902	4.21E-15
NOL9	-0.38677	4.30E-15
MRPS30	-0.30305	4.31E-15
PTBP1	-0.19864	4.42E-15
GAL3ST1	0.9407	4.62E-15
LINC00941	0.830438	4.63E-15

TMEM43	-0.35353	4.64E-15
LOC1019273	0.452679	4.65E-15
RBM12	-0.32459	4.67E-15
CEP95	-0.3217	4.70E-15
CYP2J2	0.757325	4.77E-15
DNAJA3	-0.26836	4.93E-15
XDH	2.079233	4.95E-15
TBC1D8	-0.40275	5.01E-15
MAP1LC3B	-0.2873	5.08E-15
MAPKAPK5	-0.33193	5.18E-15
ADC	1.131389	5.21E-15
STRN3	-0.35768	5.49E-15
F2RL1	0.328313	5.51E-15
PPP2CB	-0.35651	5.53E-15
DVL3	-0.2675	5.55E-15
RAC2	0.855505	5.60E-15
GTPBP4	-0.23127	5.60E-15
KCNMB4	0.807697	5.72E-15
SNX10	0.510471	5.87E-15
DNAH6	1.588165	5.93E-15
SLC30A3	0.74533	5.93E-15
ACSS2	0.309407	6.03E-15
SOCS1	1.422756	6.05E-15
ZNF629	-0.29258	6.11E-15
CDCA5	-0.27573	6.50E-15
PLEKHG1	2.22458	6.50E-15
NDEL1	-0.27691	6.56E-15
PSMC4	-0.27025	6.62E-15
JAG1	0.298669	6.76E-15
FOXL2NB	0.866114	6.81E-15
SRD5A3	0.605472	6.96E-15
HTRA1	0.511769	6.96E-15
PLEKHO1	0.648176	7.02E-15
ATG16L1	-0.33934	7.12E-15
TFAM	-0.28844	7.16E-15
ZNF268	-0.34567	7.39E-15
NCBP1	-0.24428	7.59E-15
CDCA2	-0.37161	7.62E-15
RNF139	-0.4499	7.84E-15
TMEM164	0.38824	7.89E-15
DMBT1	1.578088	7.89E-15
KIAA2013	-0.30708	7.89E-15
SNHG17	-0.28199	8.03E-15
HIRA	-0.32957	8.20E-15
EMILIN2	0.788889	8.41E-15
SIT1	0.78766	8.63E-15
BCAS2	-0.33074	9.03E-15

MICAL1	0.468554	9.14E-15
LLGL2	0.253995	9.37E-15
ATP6V0D1	-0.22625	9.38E-15
SRSF11	-0.2545	9.40E-15
ELP5	-0.28734	9.43E-15
MRPL32	-0.40042	9.52E-15
DDX19A	-0.24906	9.57E-15
SNAI2	0.381939	9.61E-15
TAF4	-0.37417	9.71E-15
HMGCR	-0.26576	9.84E-15
PSMD11	-0.24325	9.91E-15
ZNF175	-0.49689	9.92E-15
CHIC2	-0.59498	1.00E-14
MT2A	0.290606	1.03E-14
CELSR1	0.384478	1.04E-14
OVOL2	1.979788	1.04E-14
SCAF1	-0.38726	1.05E-14
ZNF746	-0.3413	1.06E-14
KIF26A	1.709286	1.06E-14
TAOK1	-0.35961	1.06E-14
FAM53B	0.369339	1.07E-14
GOLPH3L	-0.41324	1.09E-14
BAP1	-0.32858	1.12E-14
GOLT1B	-0.34969	1.13E-14
NES	0.260149	1.15E-14
SLBP	-0.25536	1.21E-14
SF1	-0.22924	1.23E-14
PRDM1	-0.56349	1.26E-14
SART1	-0.27004	1.29E-14
TMEM55B	-0.46332	1.31E-14
TGIF2	0.445553	1.31E-14
FARSA	-0.26813	1.32E-14
KCNJ11	1.234449	1.32E-14
CAP2	0.550899	1.37E-14
BFSP1	0.76659	1.38E-14
SESTD1	0.446424	1.40E-14
SRSF1	-0.25742	1.43E-14
RUSC2	0.447986	1.46E-14
SLC45A1	2.125956	1.49E-14
ITGB5	-0.24284	1.50E-14
SHC1	-0.24279	1.66E-14
XIAP	-0.27696	1.70E-14
EFR3A	0.341643	1.71E-14
SUV420H1	-0.27437	1.72E-14
ZMYND11	-0.28235	1.73E-14
EIF2S2	-0.22117	1.75E-14
GRHL1	0.57626	1.77E-14

AASDHPPT	-0.25694	1.82E-14
AKAP13	0.385152	1.83E-14
PLEKHG6	1.777112	1.83E-14
CNKSR3	0.57094	1.85E-14
SLC26A2	0.633223	1.86E-14
TSG101	-0.2638	1.88E-14
ZNF7	-0.53586	1.88E-14
ATAD2B	-0.28782	1.90E-14
SGSH	0.668835	1.98E-14
SSR4P1	1.443173	2.00E-14
RLN2	1.700291	2.01E-14
MTG2	-0.28761	2.08E-14
ZFP69	0.700344	2.11E-14
CAD	0.269253	2.15E-14
C16orf45	1.785804	2.16E-14
TDRD7	0.499483	2.16E-14
EML1	1.346731	2.17E-14
IGFBP2	0.559124	2.18E-14
TMC6	0.406803	2.22E-14
SNRNP40	-0.26804	2.24E-14
ATP6V0E2	0.418924	2.28E-14
C10orf54	0.443707	2.29E-14
WNT3	0.90422	2.31E-14
ZNHIT6	-0.27249	2.33E-14
CREBZF	-0.26858	2.35E-14
ADRB2	1.42736	2.37E-14
PABPC1	-0.21018	2.42E-14
UBN1	-0.29575	2.42E-14
KRR1	-0.26034	2.43E-14
POLR3F	-0.38338	2.52E-14
SERINC1	-0.28603	2.56E-14
BCLAF1	-0.26872	2.57E-14
ICAM2	0.579224	2.58E-14
ROCK2	-0.30766	2.59E-14
TM7SF3	0.258753	2.60E-14
SLC25A23	0.534427	2.62E-14
CNOT1	-0.27732	2.63E-14
SEC14L1	-0.32165	2.64E-14
UTP14C	-0.4418	2.66E-14
SDCBP2-AS1	0.81125	2.66E-14
GPR137B	1.24271	2.69E-14
CDC42EP2	0.529692	2.70E-14
TFAP2E	1.012595	2.71E-14
WNT6	-0.63392	2.75E-14
TRIM59	0.393619	2.91E-14
ST6GALNAC6	0.838018	2.98E-14
ACE	0.942298	2.98E-14

PNN	-0.26983	3.12E-14
OPA3	-0.40345	3.21E-14
GTF2IRD1	0.344741	3.40E-14
SLC25A25	-0.26205	3.40E-14
LARP6	0.491158	3.44E-14
MGEA5	-0.24011	3.49E-14
POLI	0.66595	3.52E-14
TMEM171	2.026749	3.53E-14
DHX30	-0.24028	3.69E-14
BSDC1	-0.35101	3.71E-14
C21orf91	-0.38055	3.80E-14
RAB21	-0.3059	3.96E-14
ASB1	-0.41048	4.01E-14
SLC37A1	0.649186	4.01E-14
STX12	-0.35515	4.17E-14
ATXN7L3	-0.31607	4.30E-14
ACO2	-0.24977	4.31E-14
TNKS2	-0.26297	4.43E-14
RNF144B	1.201429	4.46E-14
ZBTB17	-0.41921	4.55E-14
CMYA5	0.853657	4.59E-14
AKNA	0.688082	4.63E-14
NANOS1	1.285383	4.65E-14
FGFR2	2.215814	4.80E-14
RASGEF1A	1.405764	4.86E-14
CD47	0.408489	4.89E-14
LIN52	-0.47895	4.90E-14
ADCY3	0.330043	5.01E-14
COX10	-0.39941	5.07E-14
C8orf58	-0.4616	5.12E-14
KIAA0922	0.503083	5.27E-14
RPUSD4	-0.33782	5.35E-14
SOCS7	0.425162	5.36E-14
NHSL1	0.379497	5.36E-14
KLHDC8B	1.015332	5.44E-14
SETD2	-0.34978	5.55E-14
PCYOX1	0.344074	5.66E-14
RPF2	-0.29314	5.67E-14
ABCF2	-0.24941	5.73E-14
C9orf114	-0.3051	5.76E-14
B3GNT1	0.552565	5.79E-14
EAPP	-0.36488	6.05E-14
RBM14	-0.25139	6.16E-14
C17orf96	0.437197	6.38E-14
WDR66	0.619434	6.41E-14
PLK4	-0.37104	6.59E-14
APOBEC3F	0.545144	6.69E-14

PIK3C2B	0.527441	6.89E-14
C12orf4	-0.36047	6.96E-14
PITPNM1	0.483488	7.09E-14
RASGRF1	0.824646	7.15E-14
EPDR1	0.263726	7.24E-14
FTSJ3	-0.25142	7.27E-14
KIAA1429	-0.30005	7.38E-14
TSC1	-0.45723	7.46E-14
ARHGEF10L	0.376501	7.57E-14
PPM1G	-0.23438	7.63E-14
PADI1	0.684406	7.66E-14
CCDC110	1.47658	7.66E-14
ATXN2L	-0.2372	7.78E-14
BMP7	0.291542	7.80E-14
CDK13	-0.29679	8.24E-14
TGM4	1.293987	8.29E-14
CABYR	1.191073	8.38E-14
UBP1	-0.2526	8.55E-14
CREG1	0.242463	8.56E-14
PLEKHA1	0.362998	8.73E-14
MAP1S	-0.38061	8.94E-14
PEX13	-0.44635	9.41E-14
ZNF805	-0.70224	9.48E-14
IFT57	0.395715	9.51E-14
B4GALT6	1.294901	9.55E-14
NMD3	-0.25143	9.59E-14
EDRF1	-0.28808	9.62E-14
STIM1	0.284585	9.71E-14
ING1	-0.2706	9.84E-14
NHS	2.279998	9.89E-14
PSMD6	-0.26239	9.90E-14
EML5	0.682079	9.95E-14
KDM1B	0.326726	1.01E-13
PLAA	-0.27919	1.03E-13
LRP5	-0.25729	1.04E-13
CNIH3	0.727931	1.05E-13
POLDIP3	-0.22287	1.10E-13
CLTA	-0.24566	1.12E-13
IGF2BP3	-0.31196	1.13E-13
RP2	-0.33009	1.17E-13
CENPN	-0.24691	1.17E-13
SLC39A10	0.345481	1.23E-13
TBK1	-0.31783	1.24E-13
ZNF18	0.456673	1.24E-13
TRPV2	0.361649	1.25E-13
C20orf194	0.468013	1.25E-13
RNF41	-0.28612	1.25E-13

PTER	0.456426	1.28E-13
MCRS1	-0.27821	1.28E-13
MPP2	0.700683	1.29E-13
TPCN1	0.423874	1.30E-13
CLPTM1L	-0.24236	1.31E-13
NUP50-AS1	0.712253	1.32E-13
RHOD	0.311035	1.32E-13
XK	2.065842	1.32E-13
PHC3	-0.28007	1.39E-13
RRS1	-0.3334	1.41E-13
UBE2B	-0.3483	1.41E-13
HBEGF	0.342447	1.44E-13
MT1M	0.801525	1.50E-13
MTRR	-0.26182	1.50E-13
COPS3	-0.26255	1.53E-13
TMEM138	-0.4409	1.59E-13
TXNL1	-0.32989	1.60E-13
PTPN3	0.396653	1.60E-13
ZNF644	-0.27539	1.67E-13
SLC35G1	0.599684	1.69E-13
UBIAD1	-0.41031	1.73E-13
MBD6	-0.35323	1.74E-13
YARS2	-0.28491	1.76E-13
ZNF594	0.449931	1.81E-13
MTMR9	-0.43617	1.86E-13
RNF157	0.375808	1.92E-13
TTK	-0.28989	1.92E-13
ORAOV1	-0.29974	1.92E-13
BECN1	-0.31002	1.93E-13
LIN7C	-0.34877	1.94E-13
FUT8-AS1	1.440745	1.98E-13
SEC61A1	-0.2393	2.00E-13
FAM212B	0.581689	2.01E-13
SOWAHC	0.437917	2.03E-13
DOLPP1	-0.28051	2.07E-13
USP7	-0.26088	2.15E-13
MAOA	1.404696	2.19E-13
CACUL1	-0.30792	2.21E-13
FAM50B	1.252817	2.21E-13
PIK3CA	-0.4008	2.24E-13
HECTD2	1.056645	2.29E-13
FBXL16	0.569221	2.31E-13
SUPT5H	-0.22866	2.31E-13
FOXD2-AS1	2.083351	2.33E-13
ARHGAP25	0.607065	2.37E-13
CCNO	0.946736	2.40E-13
HAUS3	-0.38073	2.42E-13

RGL1	-0.28981	2.46E-13
RNF168	-0.41439	2.48E-13
RAE1	-0.2342	2.48E-13
ALOX12B	1.722064	2.49E-13
TSPAN13	0.412496	2.54E-13
NELFA	-0.29851	2.60E-13
SMG5	-0.22647	2.61E-13
PIP4K2C	-0.28508	2.62E-13
ATL3	0.554802	2.66E-13
BYSL	-0.36191	2.68E-13
WDR55	-0.32131	2.71E-13
GLIDR	0.404887	2.79E-13
DOCK11	0.518376	2.82E-13
TBC1D13	-0.39197	2.82E-13
VIL1	0.231435	2.82E-13
FIZ1	-0.38456	2.91E-13
ADD3	0.301274	2.92E-13
ANKRD11	-0.32404	3.25E-13
CACNB1	0.571662	3.26E-13
LANCL2	-0.27493	3.28E-13
TBC1D9	1.525251	3.32E-13
SETD8	-0.26455	3.49E-13
ZNF786	0.387543	3.49E-13
PMPCA	-0.27304	3.51E-13
RAB37	1.560622	3.55E-13
RPL36AL	-0.26196	3.55E-13
G3BP2	-0.25327	3.56E-13
LRRC34	0.784751	3.58E-13
MLLT3	0.807795	3.58E-13
CARKD	-0.28297	3.59E-13
TBC1D30	0.796218	3.59E-13
DNAJA4	0.420913	3.66E-13
EMC6	-0.30857	3.71E-13
TUFT1	-0.29975	3.86E-13
CYB561	0.368155	3.86E-13
ZNF589	0.369601	3.91E-13
B3GALT5	1.171903	3.91E-13
DDX49	-0.29532	4.02E-13
SLC35D3	1.468134	4.02E-13
GPR160	0.982528	4.07E-13
NCS1	0.355636	4.12E-13
ABCA5	0.538385	4.16E-13
TRUB1	-0.31049	4.16E-13
SUPT4H1	-0.26759	4.16E-13
DIABLO	-0.28577	4.23E-13
CDK11B	-0.39988	4.31E-13
RAB3A	1.685595	4.36E-13

MFSD5	-0.51785	4.40E-13
LOC642236	0.594147	4.46E-13
LRRC8D	0.297716	4.51E-13
DUSP7	0.393798	4.61E-13
RBM27	-0.25885	4.63E-13
SYT13	0.736594	4.65E-13
TAF1A	-0.41223	4.74E-13
TANK	-0.37903	4.82E-13
RSF1	-0.27205	4.85E-13
GDPD5	0.380777	4.99E-13
SETDB1	-0.32197	4.99E-13
SKIDA1	1.684465	5.02E-13
PLEKHH3	1.420069	5.08E-13
DACT3	2.157573	5.18E-13
BTG1	-0.26194	5.29E-13
RAVER1	-0.29812	5.35E-13
ZNF385C	1.213675	5.36E-13
PSMD5	-0.27834	5.46E-13
UNC5A	1.589501	5.49E-13
ASB6	-0.3389	5.54E-13
CA13	1.923296	5.64E-13
CCNI2	2.131154	5.64E-13
CENPL	-0.5103	5.68E-13
BSPRY	1.861079	5.79E-13
IGF2BP2	-0.23065	5.84E-13
DENND5B	0.461309	5.97E-13
ALCAM	0.818652	5.98E-13
LRRC49	0.456803	5.99E-13
NFE2L3	0.361969	6.07E-13
COASY	-0.27766	6.14E-13
FMOD	0.977492	6.19E-13
MT1E	0.307569	6.29E-13
ATP13A1	-0.27563	6.37E-13
SNHG1	-0.22151	6.58E-13
CSRP1	0.239292	6.63E-13
SLC41A2	0.590755	6.87E-13
RALGPS2	0.604735	6.90E-13
ZNF451	-0.28757	7.00E-13
OCEL1	0.711825	7.03E-13
FAM174A	-0.56783	7.04E-13
MARK2	-0.23217	7.10E-13
SYNCRIP	-0.23027	7.14E-13
IGSF3	1.646452	7.23E-13
EXPH5	0.519648	7.31E-13
ECEL1	1.24845	7.31E-13
NOP58	-0.25098	7.40E-13
YME1L1	-0.24107	7.58E-13

DOC2B	1.780632	7.59E-13
PITHD1	-0.25785	7.80E-13
NRGN	0.843016	7.83E-13
FAM217B	0.504762	7.84E-13
SBF2-AS1	0.740112	7.92E-13
MAPK13	0.339555	8.06E-13
GPR39	0.8498	8.17E-13
RAPGEFL1	0.674164	8.42E-13
POF1B	0.291417	8.48E-13
KCTD15	0.566568	8.63E-13
CEP350	-0.28983	8.64E-13
FOXL1	0.882093	8.64E-13
PDIA3P1	0.972753	8.84E-13
KDF1	1.232822	8.85E-13
CHMP7	-0.2801	8.87E-13
SAR1B	-0.34003	8.93E-13
CACHD1	0.293559	9.17E-13
AP3D1	-0.22136	9.20E-13
GRB10	1.015615	9.23E-13
ZC3H18	-0.22821	9.28E-13
EFNA1	-0.72854	9.36E-13
NOTCH2NL	0.635806	9.36E-13
ISM1	1.019738	9.44E-13
RAB20	0.346978	9.44E-13
SLC31A2	0.807507	9.44E-13
ACIN1	-0.2024	9.52E-13
MBD1	-0.31233	9.56E-13
LIPA	0.302996	9.56E-13
POMGNT2	0.537536	9.56E-13
ZNRD1	-0.44106	9.72E-13
FAM32A	-0.25507	9.72E-13
HNRNPL	-0.20365	9.88E-13
ARAP3	0.478267	1.01E-12
SVIL	-0.28475	1.01E-12
HNRNPAB	-0.23302	1.05E-12
CPEB4	-0.30631	1.07E-12
TESC	0.341842	1.10E-12
OXCT2	0.990164	1.10E-12
RASL11A	1.431342	1.15E-12
PITPNB	-0.25258	1.16E-12
LMTK3	1.738551	1.16E-12
LAMA5	0.404436	1.16E-12
ICT1	-0.28912	1.18E-12
TPRA1	-0.43697	1.18E-12
AP5M1	-0.34728	1.22E-12
STARD9	0.480459	1.23E-12
TMX2	-0.25223	1.25E-12

RFK	0.362778	1.27E-12
ELF4	-0.25037	1.27E-12
CIR1	-0.34399	1.27E-12
CHTOP	-0.22353	1.27E-12
PPFIA3	0.689586	1.33E-12
ACTR1A	-0.2437	1.35E-12
TIMM44	-0.27322	1.37E-12
E2F4	-0.26282	1.39E-12
TTLL4	-0.28849	1.46E-12
ZNF25	0.771778	1.50E-12
MRPS18C	-0.37584	1.52E-12
COL4A3BP	-0.33748	1.53E-12
C2orf54	0.360362	1.54E-12
CROCCP2	-0.40593	1.58E-12
HERC2	0.405641	1.58E-12
TIAM1	0.486065	1.60E-12
CPT1A	0.227807	1.64E-12
TTC9	0.476171	1.65E-12
DISP2	0.800987	1.66E-12
IGF1R	0.274734	1.68E-12
ZFAND2A	-0.4345	1.70E-12
PQBP1	-0.26506	1.71E-12
YIPF3	-0.27498	1.73E-12
SNX21	0.722103	1.73E-12
CCDC59	-0.26665	1.75E-12
HNRNPLL	-0.284	1.76E-12
HIF1AN	-0.27612	1.78E-12
PGM5P2	0.664541	1.80E-12
ABCE1	-0.24575	1.82E-12
LPIN2	0.379451	1.82E-12
SHISA5	0.393595	1.90E-12
STON1	1.748881	1.90E-12
CLDN9	1.520204	1.91E-12
TAF5L	-0.29674	1.94E-12
JPH1	0.477386	1.96E-12
TSPYL4	0.346077	1.97E-12
DCAF7	-0.20851	1.97E-12
SGCB	0.346356	1.97E-12
SLC33A1	-0.42452	1.98E-12
DHDDS	-0.32074	2.01E-12
LINC01123	1.350578	2.14E-12
FGF9	0.734534	2.17E-12
PNO1	-0.28125	2.23E-12
SLC25A28	-0.80304	2.25E-12
PISD	-0.32566	2.27E-12
ZNF318	0.300916	2.29E-12
NCBP2-AS2	-0.39123	2.30E-12

DCLK2	1.84668	2.32E-12
ATG14	-0.54136	2.36E-12
ERC2	2.100198	2.41E-12
LLPH	-0.28596	2.43E-12
DUSP4	-0.3197	2.46E-12
KIAA1161	0.498026	2.48E-12
PSMG2	-0.29984	2.48E-12
MED6	-0.30039	2.48E-12
SLC35B1	-0.31791	2.50E-12
TOR1AIP1	-0.25141	2.50E-12
GTF3C4	-0.43887	2.51E-12
MTM1	-0.37551	2.54E-12
LMO7	-0.19663	2.60E-12
PHACTR3	0.953771	2.61E-12
C9orf40	0.426877	2.64E-12
TMBIM6	-0.20318	2.66E-12
MAP3K15	0.549453	2.67E-12
FADS1	0.244466	2.67E-12
HMGXB4	-0.29908	2.67E-12
AFF1	0.263694	2.67E-12
KISS1R	1.080076	2.68E-12
CLPTM1	-0.2613	2.73E-12
MMP17	0.523674	2.80E-12
CUL2	-0.29087	2.81E-12
ILKAP	-0.32089	2.82E-12
PELI3	0.94755	2.84E-12
SECISBP2L	-0.26425	2.85E-12
ABCC9	0.472143	2.85E-12
OASL	2.095248	2.87E-12
ERVMER34-1	0.727485	2.96E-12
PANK2	-0.25484	2.98E-12
UBE2W	-0.32881	2.98E-12
C11orf95	0.405784	3.02E-12
PARP2	-0.31915	3.04E-12
KCTD2	-0.34287	3.06E-12
EPSTI1	1.110551	3.08E-12
SLC12A7	-0.21127	3.21E-12
BZW1	-0.21885	3.23E-12
CREG2	1.870066	3.26E-12
FBXL19	0.438751	3.27E-12
KLHL15	-0.41877	3.28E-12
CCAR2	-0.20517	3.30E-12
COPS5	-0.30017	3.36E-12
C22orf46	0.33207	3.38E-12
ERBB2	0.414353	3.38E-12
TTC9C	-0.3697	3.41E-12
LSM1	-0.33399	3.50E-12

CLASRP	-0.23863	3.52E-12
CRISPLD1	1.081183	3.56E-12
PSMD12	-0.29429	3.69E-12
ZNF174	0.466849	3.70E-12
C19orf44	0.435751	3.76E-12
FAM115C	2.004958	3.78E-12
SMIM6	0.509143	3.79E-12
RAD21	-0.21667	3.81E-12
RIC8A	-0.24576	3.88E-12
EHHADH	0.41043	3.94E-12
GPNMB	1.841084	3.95E-12
BROX	-0.30947	4.06E-12
METAP2	-0.20513	4.13E-12
LAMP3	1.837438	4.14E-12
SMCO4	1.02183	4.14E-12
L3MBTL2	-0.25528	4.16E-12
NOP16	-0.41064	4.17E-12
ANO1	0.365434	4.18E-12
IGFBP4	0.208192	4.21E-12
GPN2	-0.39034	4.28E-12
ACOT11	0.845563	4.28E-12
SIMC1	0.457619	4.38E-12
ANGPTL2	1.062802	4.47E-12
NAA30	-0.32895	4.47E-12
ZNF557	-0.57401	4.52E-12
TNRC18	-0.27495	4.53E-12
SERPINE2	0.328031	4.54E-12
PROC	1.365323	4.62E-12
SEL1L3	0.377171	4.90E-12
C5orf22	-0.27043	4.92E-12
C17orf58	0.3329	4.97E-12
TRIM10	0.824238	5.04E-12
BSN	0.480034	5.11E-12
CNBP	-0.20602	5.11E-12
PLEKHF1	1.35344	5.17E-12
HMGB2	-0.2191	5.22E-12
ZNF274	0.287575	5.23E-12
LOC1005061	1.082337	5.31E-12
LINC00624	1.217587	5.35E-12
CDKN1C	0.761381	5.40E-12
RNF6	-0.30975	5.44E-12
PRPF40B	0.559766	5.47E-12
DPP8	-0.29562	5.48E-12
LOC1001293	-0.28862	5.49E-12
FAM102A	0.448615	5.50E-12
PLEC	-0.23459	5.51E-12
CERS4	0.561918	5.53E-12

T	0.71634	5.66E-12
GOLGA6C	1.097885	5.94E-12
MIS12	-0.24377	5.95E-12
IKZF5	-0.40721	5.95E-12
MAP7D2	0.417583	5.96E-12
PSMC6	-0.31394	6.01E-12
NRDE2	-0.427	6.01E-12
PRRC2A	-0.22461	6.02E-12
CCNA2	-0.35793	6.07E-12
FAM129B	0.245907	6.08E-12
SPATA7	0.592076	6.09E-12
RAVER2	0.336886	6.10E-12
FAM179B	0.398591	6.17E-12
ADIPOR1	-0.24092	6.19E-12
SMTNL2	1.894912	6.25E-12
BRSK2	1.175476	6.29E-12
LCOR	-0.36185	6.34E-12
PIK3R3	0.379289	6.43E-12
RCHY1	-0.33235	6.44E-12
FAM104A	-0.26068	6.46E-12
POLDIP2	-0.25354	6.46E-12
DEPDC5	0.40552	6.56E-12
FAM171B	1.409537	6.63E-12
ATP11A	0.314837	6.68E-12
DCP1A	-0.27845	6.71E-12
ATP1B1	0.284701	6.73E-12
SENP6	-0.23948	6.76E-12
MAN1A1	0.393071	6.95E-12
RINT1	-0.29349	6.95E-12
SORBS3	0.310695	7.08E-12
FAM122B	-0.21884	7.11E-12
DPF1	0.443698	7.37E-12
PRR3	-0.28092	7.39E-12
USP11	-0.25054	7.39E-12
HIST1H2BD	0.381028	7.45E-12
FAM107B	0.418017	7.45E-12
HECTD1	-0.33371	7.47E-12
PLEKHG4B	0.294268	7.50E-12
GATAD2B	-0.35612	7.58E-12
SCO1	-0.28765	7.61E-12
NSFL1C	-0.21434	7.72E-12
EIF6	-0.19923	7.83E-12
SH3BGRL2	-0.25688	7.92E-12
ATP1A3	1.180732	7.98E-12
PPAP2A	0.477252	8.10E-12
GNAO1	1.053436	8.23E-12
GMPR	1.084554	8.25E-12

CXorf40B	-0.45947	8.31E-12
ATAD5	-0.32074	8.35E-12
DOCK6	0.266793	8.35E-12
C1QTNF2	1.745258	8.39E-12
GPKOW	-0.34667	8.39E-12
TTC33	-0.44461	8.66E-12
UBE2K	-0.22224	8.66E-12
B3GAT1	0.693898	8.85E-12
C11orf24	-0.25157	8.98E-12
CAPN7	-0.26786	9.11E-12
CYTH1	0.283273	9.12E-12
PLIN3	0.256823	9.14E-12
AMDHD1	0.948143	9.21E-12
KRT40	-0.2955	9.31E-12
CD4	0.449143	9.33E-12
SPRED1	-0.26977	9.39E-12
PDZD8	-0.33542	9.49E-12
MT1DP	2.05786	9.53E-12
SLC46A3	0.914282	9.98E-12
F2RL3	0.590233	1.02E-11
SLC4A1AP	-0.30755	1.03E-11
ABCC3	0.44189	1.09E-11
LINC01003	0.752252	1.09E-11
SH3KBP1	0.290455	1.10E-11
ISG20L2	-0.27969	1.11E-11
CYP27A1	0.696471	1.11E-11
SHROOM2	0.44766	1.12E-11
NDUFS3	-0.29731	1.12E-11
SH2B3	0.411928	1.12E-11
ZNF772	1.078181	1.13E-11
ARHGAP29	-0.24156	1.13E-11
NPLOC4	-0.20683	1.13E-11
GGACT	0.863084	1.15E-11
PARP14	0.400576	1.16E-11
IVNS1ABP	-0.26822	1.19E-11
NOP56	-0.17405	1.20E-11
IGDCC3	0.673816	1.23E-11
IQCC	-0.56232	1.25E-11
MYO5B	0.324221	1.29E-11
HBP1	-0.74908	1.31E-11
RIOK1	-0.25519	1.32E-11
ABHD13	-0.32495	1.34E-11
USP47	-0.25689	1.36E-11
TMEM45A	0.691734	1.37E-11
SFXN5	0.473656	1.37E-11
SLC25A43	0.440026	1.38E-11
BLOC1S3	-0.79033	1.42E-11

TMEM158	0.771248	1.42E-11
SNX30	0.353739	1.43E-11
TCF7	0.269305	1.44E-11
FNDC5	1.802735	1.46E-11
STXBP1	0.300006	1.47E-11
EVPL	0.334116	1.49E-11
PTGES	1.656067	1.51E-11
PNRC1	-0.5238	1.52E-11
FAM179A	1.103841	1.52E-11
AP3B2	0.899053	1.53E-11
FKBP7	0.619034	1.56E-11
SNX5	-0.22602	1.57E-11
CLK4	-0.43978	1.57E-11
RTFDC1	-0.2207	1.57E-11
ASH1L	-0.39988	1.58E-11
MX2	0.394889	1.58E-11
PTPRJ	0.245819	1.58E-11
DNAJB12	-0.23711	1.59E-11
DAZAP2	-0.18631	1.61E-11
ETV6	0.384051	1.68E-11
ILDR2	0.33914	1.69E-11
TRPM4	0.733004	1.70E-11
SLC25A3	-0.20724	1.70E-11
UPF1	-0.21575	1.72E-11
PDK3	0.476745	1.72E-11
LIPH	1.186269	1.75E-11
NUP98	-0.21865	1.79E-11
PLEKHG4	0.363318	1.79E-11
C6orf1	0.490966	1.79E-11
NAPA	-0.28233	1.79E-11
MESDC2	-0.29341	1.83E-11
ZNF567	-0.55048	1.88E-11
MTF1	-0.31857	1.92E-11
PSMC2	-0.24727	1.94E-11
GNL2	-0.24112	1.94E-11
USPL1	-0.48053	2.00E-11
ZCCHC6	-0.2776	2.04E-11
EEF1A2	0.259775	2.07E-11
ATP6V1C1	-0.29643	2.16E-11
KAT6A	-0.27935	2.16E-11
SRA1	-0.27694	2.21E-11
SERINC3	-0.24336	2.24E-11
NR1H2	-0.35959	2.24E-11
UACA	0.293898	2.25E-11
HDAC11	0.545411	2.26E-11
PPP1R2	-0.25003	2.30E-11
CDAN1	-0.3256	2.31E-11

FAM57B	1.663606	2.31E-11
UBE2J1	-0.20192	2.31E-11
HERPUD1	-0.40738	2.33E-11
OSBP2	0.455072	2.34E-11
MAP7	0.312604	2.34E-11
TEFM	-0.58268	2.44E-11
MRPS35	-0.24552	2.45E-11
SNX4	-0.31125	2.46E-11
FASTKD3	-0.40692	2.46E-11
ASXL1	-0.22835	2.47E-11
TAF11	-0.31567	2.52E-11
ARHGAP1	-0.24153	2.58E-11
SMARCD1	-0.20565	2.59E-11
ZC3H15	-0.24062	2.63E-11
TSPAN9	0.30903	2.64E-11
ATP2B1	0.376475	2.65E-11
SLC39A7	-0.24593	2.68E-11
IGDCC4	0.827075	2.72E-11
LRRC57	-0.4097	2.75E-11
HTRA3	1.319313	2.75E-11
FAR2P2	0.912628	2.76E-11
DYRK2	0.482219	2.78E-11
DIS3	-0.19364	2.89E-11
LRR1	-0.38998	2.89E-11
VPS39	-0.24113	2.90E-11
USP19	-0.25233	2.91E-11
GMEB2	-0.25714	2.93E-11
HOXD11	2.160655	2.94E-11
REEP6	0.593954	2.98E-11
TRIM58	1.401252	3.00E-11
TICRR	-0.38653	3.00E-11
OTUD6B	-0.27451	3.00E-11
DYNC2H1	0.481476	3.01E-11
CCDC87	1.571944	3.02E-11
PTPRE	0.277018	3.10E-11
DTL	-0.26579	3.14E-11
CALHM2	0.919589	3.20E-11
MT1F	0.516369	3.21E-11
ZFYVE26	-0.37918	3.23E-11
SRP19	-0.32082	3.25E-11
CRCP	-0.25992	3.30E-11
ZNF639	-0.38464	3.32E-11
CAPN6	1.590418	3.33E-11
COQ10A	0.548055	3.34E-11
LIN9	-0.33522	3.42E-11
PRPF6	-0.19622	3.42E-11
TRAPPC4	-0.30887	3.43E-11

EIF1	-0.20261	3.44E-11
PPM1H	0.419284	3.46E-11
GPR19	1.556509	3.51E-11
TRAPPC13	-0.3004	3.52E-11
TENC1	0.341924	3.54E-11
NUP35	-0.30037	3.56E-11
ZNF862	0.462835	3.63E-11
PHAX	-0.28073	3.65E-11
NDUFV3	-0.31518	3.74E-11
LZTFL1	-0.36581	3.79E-11
LIMK1	0.366401	3.83E-11
RTN2	0.722242	3.87E-11
KSR1	0.746608	3.88E-11
ZBTB8A	0.542437	3.88E-11
HELZ	-0.33145	3.89E-11
PGBD2	0.633475	3.94E-11
TMED7	-0.29837	3.98E-11
GOLM1	0.250418	4.04E-11
ENPP4	0.385499	4.11E-11
TMEM165	-0.29089	4.16E-11
FLAD1	-0.29152	4.22E-11
XRN1	-0.31378	4.29E-11
PKP2	0.220942	4.34E-11
WASF3	0.525136	4.37E-11
KBTBD3	0.825019	4.38E-11
SPNS3	0.482431	4.40E-11
ZNF707	-0.53541	4.51E-11
CCDC117	-0.42584	4.60E-11
DLST	-0.20116	4.63E-11
DHX37	-0.20932	4.68E-11
PRKG2	0.488209	4.72E-11
GOLGA5	-0.31712	4.84E-11
ANKEF1	0.386206	4.89E-11
ENTPD8	0.476185	4.89E-11
FOXP1	0.42206	4.89E-11
RASL10B	0.497014	4.92E-11
AQP3	0.353676	5.01E-11
TRMT61B	-0.4812	5.01E-11
MALSU1	-0.41253	5.02E-11
BTF3L4	-0.26545	5.11E-11
RARS	-0.21842	5.14E-11
NCKAP5L	-0.25728	5.16E-11
SETDB2	0.311711	5.19E-11
PITPNM3	0.576977	5.21E-11
RNF31	-0.34617	5.24E-11
MYH9	-0.18444	5.29E-11
PCIF1	-0.34032	5.29E-11

PCDHAC2	1.825609	5.34E-11
TM9SF2	-0.20982	5.36E-11
ACVR1	0.515031	5.39E-11
ZBED6CL	0.368659	5.40E-11
MFI2	0.493468	5.53E-11
CUL5	-0.32642	5.64E-11
CNOT7	-0.21282	5.67E-11
SORT1	0.260564	5.79E-11
KIAA1551	0.302942	5.83E-11
C4orf32	0.466292	5.95E-11
GRAMD4	0.874051	6.05E-11
RBM3	-0.19052	6.06E-11
TIMM10B	-0.28357	6.13E-11
SMIM13	0.464262	6.16E-11
RELB	-0.45565	6.18E-11
STRN4	-0.28034	6.25E-11
DAG1	0.295073	6.26E-11
PSCA	1.475243	6.30E-11
KLHL9	-0.33916	6.34E-11
ADAMTS7	1.922081	6.44E-11
DMXL2	0.368675	6.45E-11
ZNRF1	0.336005	6.53E-11
PARD6A	0.5421	6.65E-11
DNAJC3	-0.26821	6.87E-11
LOC646903	1.889247	7.13E-11
ERLEC1	-0.33476	7.22E-11
PEX16	-0.31713	7.34E-11
CYBRD1	0.919784	7.34E-11
ABCB9	0.895678	7.40E-11
PBX3	0.917246	7.41E-11
RGS14	0.353501	7.44E-11
SAMHD1	0.306963	7.47E-11
CHMP1A	-0.22414	7.52E-11
SEL1L	-0.26737	7.52E-11
NREP	0.789241	7.61E-11
TECPR2	0.369588	7.61E-11
C6orf48	-0.20983	7.63E-11
GPX3	1.393704	7.92E-11
MBD4	-0.28046	7.95E-11
UBQLN1	-0.19105	7.98E-11
HDAC7	-0.3023	8.08E-11
FBXO45	-0.27481	8.19E-11
HMGA2	-0.45416	8.27E-11
ARHGAP40	0.631549	8.27E-11
SLC7A8	0.276595	8.28E-11
ZBTB33	-0.25278	8.32E-11
ACVR1B	0.283178	8.48E-11

PMS2CL	-0.44934	8.57E-11
KIF13A	0.323862	8.63E-11
FGFBP3	0.92582	8.78E-11
SUN2	0.2821	9.05E-11
ITPRIPL2	0.473537	9.10E-11
PITPNA	-0.19105	9.27E-11
SYTL4	0.879122	9.36E-11
PES1	-0.19927	9.36E-11
STPG1	0.408712	9.62E-11
NAB2	0.370191	9.69E-11
TMEM206	0.417689	9.69E-11
IKZF2	1.244749	9.71E-11
RAB3B	1.317053	1.01E-10
TMEM167B	-0.34457	1.02E-10
RASL11B	0.930278	1.02E-10
CLTC	-0.22496	1.03E-10
SAMD13	1.648388	1.03E-10
CNNM4	0.408909	1.04E-10
CCDC43	-0.30866	1.04E-10
WDR36	-0.23561	1.05E-10
LOC642852	0.594343	1.05E-10
WDR73	-0.27517	1.05E-10
NKRF	-0.26325	1.06E-10
SLCO2B1	1.943138	1.07E-10
TOP3B	-0.29055	1.08E-10
TRIM45	0.927473	1.08E-10
TM9SF4	-0.22413	1.09E-10
C17orf85	-0.23606	1.09E-10
CDK20	0.893173	1.12E-10
NEURL4	-0.3129	1.17E-10
U2AF1	-0.22152	1.18E-10
C18orf21	-0.50883	1.19E-10
DNAJC6	0.224514	1.20E-10
MGME1	-0.27907	1.21E-10
TBX20	1.01547	1.23E-10
SS18L1	-0.35307	1.23E-10
E2F3	-0.30637	1.27E-10
GLTPD1	-0.50906	1.30E-10
CHD2	-0.23996	1.32E-10
HMHA1	0.396141	1.33E-10
C1orf220	1.263275	1.34E-10
SNCB	1.752937	1.34E-10
TSPAN33	0.499683	1.38E-10
P4HA2	0.584882	1.38E-10
TYSND1	0.435861	1.38E-10
POLE3	-0.21217	1.41E-10
LAMTOR3	-0.45725	1.42E-10

MMGT1	-0.28575	1.42E-10
FNIP1	-0.37473	1.42E-10
WDR48	-0.2797	1.42E-10
TOPBP1	-0.23033	1.44E-10
PRSS22	1.213412	1.44E-10
ZBTB5	-0.41535	1.46E-10
MR1	0.600746	1.46E-10
DHX34	-0.31908	1.46E-10
POLR3D	-0.31206	1.47E-10
SHCBP1	-0.26191	1.48E-10
ZSWIM5	0.346295	1.49E-10
SLC22A3	0.511927	1.49E-10
KRT86	0.673317	1.50E-10
SENP1	-0.23325	1.52E-10
C1orf50	-0.4239	1.54E-10
DNAJB9	-1.4084	1.54E-10
PDCD6	-0.22938	1.54E-10
FBXL2	0.554192	1.56E-10
ABHD2	-0.21843	1.58E-10
ZNFX1	-0.38863	1.60E-10
PTAFR	1.049449	1.60E-10
DUSP5	-0.30139	1.61E-10
TFRC	-0.21811	1.61E-10
PSD	1.670823	1.65E-10
RLTPR	0.282465	1.66E-10
CITED2	-0.28507	1.68E-10
PER2	-0.42469	1.68E-10
NAA25	-0.2592	1.68E-10
ABAT	0.87302	1.71E-10
EMC3	-0.32733	1.71E-10
SLC1A7	1.56909	1.71E-10
TTYH3	0.197068	1.72E-10
MKL1	-0.22685	1.72E-10
BTBD1	-0.2103	1.73E-10
EPHA4	0.301044	1.73E-10
TCERG1	-0.22033	1.76E-10
TAB2	-0.19998	1.81E-10
PRKD3	0.246725	1.83E-10
PRKAB2	-0.40228	1.86E-10
RAB40C	-0.4907	1.87E-10
GSPT1	-0.20192	1.91E-10
ABCC2	0.361241	1.91E-10
TAF13	-0.50961	1.91E-10
UBE2L3	-0.19481	1.93E-10
MED28	-0.4238	1.93E-10
SASS6	-0.34759	1.97E-10
ORAI2	0.286091	2.06E-10

DOCK9-AS2	1.542045	2.09E-10
PPP1CC	-0.18981	2.13E-10
EPN2	0.352429	2.15E-10
C22orf29	0.325234	2.16E-10
LMAN2L	-0.36823	2.16E-10
OSBPL2	-0.30454	2.20E-10
KLLN	1.062108	2.23E-10
CNOT2	-0.22038	2.23E-10
C3orf17	-0.25918	2.23E-10
LOC1019273	1.096488	2.30E-10
ADCK2	0.535635	2.30E-10
CTDNEP1	-0.21508	2.30E-10
CHST11	0.29884	2.32E-10
FBXO3	-0.36699	2.32E-10
ZNF605	0.670039	2.36E-10
SOCS4	-0.26897	2.42E-10
LGR5	-0.28337	2.46E-10
PCGF1	-0.37853	2.48E-10
PPP4R1	-0.21293	2.48E-10
SAPCD2	0.214171	2.50E-10
SH2D2A	1.215916	2.53E-10
TSFM	-0.32508	2.53E-10
PPTC7	-0.36523	2.53E-10
INPPL1	0.215292	2.53E-10
FZD3	0.567282	2.57E-10
ZNF598	-0.29009	2.57E-10
KCNH2	0.503093	2.61E-10
MED10	-0.23033	2.69E-10
SRSF7	-0.2255	2.70E-10
EIF3B	-0.18707	2.72E-10
GOSR2	-0.25885	2.73E-10
TOR1A	-0.27958	2.76E-10
FBXO6	0.929782	2.79E-10
CSDE1	-0.20288	2.80E-10
ACTR8	-0.29079	2.81E-10
TUT1	-0.37108	2.82E-10
C1orf198	-0.25076	2.83E-10
PDE3B	0.420456	2.86E-10
EIF4G1	-0.17561	2.86E-10
BAG1	-0.24381	2.88E-10
KATNAL1	0.915065	2.92E-10
STK11	-0.437	2.94E-10
HSPA9	-0.17711	2.99E-10
MREG	0.54122	3.02E-10
IFT46	0.360357	3.07E-10
ZDHHC2	0.439217	3.14E-10
ARHGEF4	0.529809	3.15E-10

R3HDM4	-0.22697	3.15E-10
NBEAL2	0.33218	3.15E-10
TMEM64	0.625715	3.18E-10
FBXO8	-0.43618	3.18E-10
BCR	0.294415	3.19E-10
LDOC1L	0.229767	3.20E-10
TRIM5	0.295774	3.22E-10
ZNF501	0.579924	3.23E-10
MTMR3	-0.29152	3.23E-10
ZNF32	0.447054	3.25E-10
RNF223	1.717732	3.25E-10
DYRK3	0.375129	3.26E-10
ERMP1	0.328184	3.26E-10
ITPKC	-0.47425	3.27E-10
HNRNPD	-0.17521	3.28E-10
BTBD7	-0.47755	3.28E-10
PCID2	-0.20966	3.29E-10
DDX21	-0.20174	3.37E-10
TMEM185B	0.312963	3.42E-10
ZNF204P	1.280678	3.42E-10
C7orf13	0.892217	3.45E-10
XKRX	1.402615	3.46E-10
ZNF254	-0.32265	3.50E-10
AFAP1L1	1.318119	3.51E-10
LRCH1	-0.28659	3.53E-10
LINC00341	0.734605	3.55E-10
FCF1	-0.24972	3.59E-10
CWF19L1	-0.29522	3.60E-10
UGDH	-0.26289	3.66E-10
GYG2	1.406973	3.69E-10
LYPD6	1.277415	3.73E-10
CCDC86	-0.21275	3.74E-10
CACNA1H	0.415087	3.78E-10
ANKRD1	0.372299	3.79E-10
ARCN1	-0.19364	3.82E-10
CROT	0.644356	3.91E-10
AK7	1.148412	3.92E-10
KIF2C	-0.20678	3.99E-10
NPTN	-0.24598	4.09E-10
ARHGEF2	0.266763	4.12E-10
CIRH1A	-0.18879	4.19E-10
KIAA0232	-0.35231	4.21E-10
JUND	0.195617	4.22E-10
SSH3	0.294429	4.27E-10
RUFY3	0.396769	4.36E-10
CGNL1	0.399493	4.36E-10
FMO5	1.411255	4.36E-10

KCNK6	0.604306	4.39E-10
STARD10	0.377294	4.49E-10
MEGF9	0.479859	4.49E-10
TBRG1	-0.22222	4.50E-10
DKK3	1.419201	4.53E-10
MICAL2	0.492386	4.53E-10
PDPK1	-0.23276	4.54E-10
GPATCH4	-0.25266	4.55E-10
SGSM1	0.960654	4.65E-10
NR2F6	0.379438	4.66E-10
PAXBP1	-0.21586	4.72E-10
MGA	0.342341	4.74E-10
TMEM150C	1.358407	4.84E-10
SCN1B	1.211646	4.84E-10
TRPM6	1.62083	4.98E-10
TMPRSS2	0.25695	5.11E-10
SHANK2-AS1	1.80673	5.11E-10
DNTTIP1	-0.24683	5.12E-10
CHKA	-0.17834	5.17E-10
DCTN6	-0.26798	5.25E-10
WIBG	-0.25991	5.27E-10
ADAT2	-0.57501	5.32E-10
ABHD8	0.77914	5.51E-10
PRDM8	1.908649	5.57E-10
RNH1	-0.22893	5.63E-10
ZNF483	1.624593	5.69E-10
LOXL3	0.523657	5.83E-10
SP2	-0.26819	5.85E-10
EDN1	-0.25326	5.92E-10
MECP2	-0.23058	5.94E-10
TRMT12	-0.54023	5.98E-10
RAB8A	-0.23683	6.06E-10
PWP2	-0.28859	6.12E-10
RIC8B	0.315358	6.25E-10
REEP2	0.512917	6.30E-10
TMEM38A	1.162057	6.33E-10
JUN	-0.18157	6.33E-10
TTC30A	0.677379	6.33E-10
NEK4	-0.2866	6.34E-10
RAB29	0.669136	6.36E-10
PTTG1IP	-0.165	6.42E-10
CREB1	-0.26253	6.53E-10
PMAIP1	-0.28502	6.54E-10
SEC24B	-0.26906	6.63E-10
RAB23	0.379948	6.65E-10
NEDD4L	-0.23273	6.66E-10
MTFR2	-0.43736	6.66E-10

PCDHB14	0.951278	6.68E-10
HAUS2	-0.21846	6.71E-10
LRRC16B	1.187224	6.71E-10
FOXO3B	0.731518	6.86E-10
NCOA6	-0.22674	6.87E-10
RNF185	-0.30324	6.87E-10
LSM14A	-0.21458	6.91E-10
ENTPD7	-0.34268	6.93E-10
TMEM161B	-0.34348	6.93E-10
UTP11L	-0.236	7.08E-10
LRRC41	-0.25245	7.18E-10
ANKRD49	-0.35494	7.28E-10
CDC42	-0.18675	7.30E-10
PCYT2	-0.2344	7.31E-10
DNALI1	1.075485	7.41E-10
CCNC	-0.27686	7.47E-10
CITED1	1.294211	7.68E-10
SEC14L4	0.597342	7.71E-10
KIAA1324	1.235713	7.72E-10
ARPC4	-0.22683	7.74E-10
TIMM17A	-0.23687	7.98E-10
IGSF9B	0.96298	8.00E-10
MID2	0.660367	8.06E-10
CHORDC1	-0.2057	8.07E-10
NDUFV2	-0.21615	8.08E-10
SLC22A18	0.634353	8.14E-10
FEM1C	-0.35025	8.16E-10
TNK2	0.28872	8.17E-10
PXDN	1.168101	8.29E-10
SF3A1	-0.17543	8.31E-10
GAB1	1.650843	8.36E-10
ZC3H4	-0.24191	8.38E-10
TFB2M	-0.39491	8.43E-10
MUM1L1	0.303067	8.48E-10
ARNT2	0.277378	8.79E-10
PURB	-0.36663	8.92E-10
DCLRE1C	-0.29203	9.10E-10
TPRG1L	-0.52077	9.26E-10
LIG4	0.248144	9.27E-10
CRLS1	-0.27794	9.36E-10
INCENP	-0.27039	9.41E-10
CTBP1-AS2	0.627421	9.42E-10
GPSM2	0.376034	9.43E-10
ENTHD2	-0.36573	9.46E-10
EYA3	-0.26881	9.49E-10
TIGD4	0.684809	9.52E-10
EML6	1.195171	9.54E-10

BATF	0.489612	9.72E-10
PLXNA1	0.265407	9.80E-10
ODC1	-0.18899	9.81E-10
HDAC9	-0.35675	9.91E-10
MRTO4	-0.21658	9.99E-10
PCDH19	0.504237	1.01E-09
METTL16	-0.23777	1.02E-09
YIPF6	-0.22339	1.02E-09
USP43	0.327318	1.03E-09
SMARCA1	0.295487	1.04E-09
ABCC8	1.329288	1.04E-09
TMEM25	0.565771	1.05E-09
ASB13	0.361927	1.05E-09
CNOT8	-0.2715	1.05E-09
UQCRFS1	-0.23569	1.07E-09
SRCAP	-0.25921	1.07E-09
PRKAB1	-0.24475	1.08E-09
ZNF823	-0.46075	1.09E-09
UNC13D	0.449934	1.09E-09
WWC1	0.316526	1.09E-09
MOB2	-0.39311	1.09E-09
DNAJC16	-0.37767	1.09E-09
GPR161	1.587745	1.10E-09
ADORA2B	0.432217	1.12E-09
ATN1	-0.19276	1.12E-09
JAZF1	0.951894	1.12E-09
HIATL1	-0.27439	1.14E-09
LOC1019282	1.685318	1.14E-09
NPC1	-0.27663	1.15E-09
NF2	-0.28225	1.20E-09
NAA35	-0.29017	1.21E-09
GLS	-0.23362	1.21E-09
CRHR1-IT1	0.955982	1.21E-09
TAF2	-0.21508	1.21E-09
FYN	-0.22224	1.22E-09
LIMS1	-0.23028	1.24E-09
IL18	0.75326	1.24E-09
PAK1	0.21829	1.26E-09
ZC3H7A	-0.24625	1.27E-09
RAB5C	-0.21296	1.30E-09
LGR6	0.29088	1.32E-09
EPB41L1	0.308151	1.37E-09
DDX18	-0.19514	1.37E-09
TCTA	0.369928	1.37E-09
MST1R	0.261965	1.38E-09
AEN	-0.27054	1.38E-09
RBM17	-0.2056	1.39E-09

SLC23A2	0.298374	1.39E-09
MANEAL	0.396219	1.41E-09
DGCR2	-0.24311	1.42E-09
ZNF430	-0.31955	1.44E-09
FSD1L	0.398521	1.45E-09
FASTK	-0.30405	1.45E-09
MAP3K2	-0.25676	1.48E-09
MICB	0.341493	1.48E-09
DSE	0.996952	1.48E-09
TMEM222	-0.30545	1.49E-09
C15orf38	0.337842	1.49E-09
ANLN	-0.21668	1.49E-09
GTF3C2	-0.21358	1.53E-09
REPIN1	-0.20196	1.53E-09
EIF1B	-0.26355	1.55E-09
BUD31	-0.21461	1.56E-09
MCF2L-AS1	0.893	1.58E-09
TEX10	-0.30028	1.59E-09
ZZZ3	-0.24283	1.60E-09
ZBTB6	-0.44273	1.60E-09
KCNQ4	0.950401	1.61E-09
TMEM54	0.487988	1.64E-09
ECE1	0.232943	1.67E-09
UBE2N	-0.18552	1.70E-09
THNSL2	1.192245	1.71E-09
DHCR24	0.247167	1.71E-09
VAT1L	1.540135	1.71E-09
FBLIM1	0.39319	1.73E-09
CLRN3	0.562442	1.73E-09
MERTK	0.372539	1.73E-09
PRSS3	0.642933	1.73E-09
KBTBD7	1.051808	1.74E-09
EID2	-0.3677	1.74E-09
THSD4	0.404985	1.75E-09
PDE9A	0.602533	1.78E-09
YES1	-0.27211	1.80E-09
DYNLL1	-0.23856	1.84E-09
TYRO3	0.292133	1.84E-09
ASXL2	-0.32129	1.84E-09
YEATS2	0.214535	1.85E-09
PDCD11	-0.22925	1.85E-09
KIAA1191	-0.25284	1.87E-09
SPTB	1.06133	1.88E-09
ABCA7	0.407667	1.90E-09
DGKE	0.36573	1.90E-09
C1R	0.594474	1.91E-09
BTBD10	-0.22305	1.91E-09

ATP6V0A1	0.322948	1.93E-09
MFAP2	0.307593	1.96E-09
NECAP2	-0.29958	1.97E-09
DDX39A	-0.22899	1.97E-09
GOT1	-0.23828	1.99E-09
LYSMD4	0.616866	2.00E-09
PAIP1	-0.20293	2.01E-09
TRIP10	0.343043	2.03E-09
LINC01268	0.81452	2.03E-09
KCTD10	-0.31602	2.06E-09
TERF2	-0.18348	2.07E-09
FAT1	0.324581	2.08E-09
ZNF26	-0.39913	2.12E-09
GLYR1	-0.21774	2.21E-09
AQR	-0.18949	2.22E-09
BRD7	-0.17464	2.23E-09
KIF1C	-0.21345	2.27E-09
SPCS2	-0.21956	2.28E-09
ZNF16	-0.45287	2.28E-09
LTBP2	1.16945	2.30E-09
KCTD9	-0.25948	2.30E-09
BTN2A2	0.406675	2.32E-09
TBP	-0.31439	2.36E-09
MDK	0.286007	2.37E-09
STRAP	-0.17733	2.38E-09
PATL1	-0.20699	2.40E-09
SRRT	-0.18084	2.42E-09
CPE	0.773852	2.42E-09
PRDM15	-0.35191	2.42E-09
PRDM4	-0.25299	2.43E-09
LOC90246	1.623047	2.43E-09
CTNNAL1	-0.2159	2.46E-09
TRIP12	-0.21709	2.47E-09
TRAPPC12	-0.27211	2.47E-09
SLC2A14	0.940092	2.47E-09
ZFAND4	0.445008	2.48E-09
ANAPC13	-0.28021	2.52E-09
CDC14B	0.330372	2.53E-09
PPP4R2	-0.33472	2.55E-09
CSNK1G1	-0.24518	2.57E-09
WDR75	-0.20781	2.59E-09
BMS1	-0.18969	2.61E-09
RAPGEF2	0.281174	2.64E-09
RSL1D1	-0.19136	2.69E-09
DPH1	-0.30525	2.70E-09
MYZAP	1.026711	2.72E-09
TRIM27	-0.21982	2.72E-09

SSX2IP	0.387826	2.77E-09
SH3GL1	-0.25002	2.83E-09
SLC35A4	-0.26315	2.84E-09
DHX16	-0.2045	2.86E-09
SLC2A11	0.471369	2.88E-09
MAGEB6	1.185488	2.90E-09
KIAA1524	-0.23154	2.95E-09
GOLGA1	-0.30123	2.97E-09
LOXL1	0.549327	2.98E-09
SEPHS2	-0.24279	3.01E-09
SERP1	-0.23347	3.01E-09
ARRB2	0.29866	3.01E-09
ARRB1	0.455947	3.03E-09
GPANK1	-0.46029	3.06E-09
ST3GAL1	1.890814	3.08E-09
RIMKLB	0.977434	3.08E-09
KPNA1	-0.20945	3.09E-09
AFG3L2	-0.20491	3.09E-09
WNT10A	0.556312	3.10E-09
SMAD6	0.455501	3.11E-09
EI24	-0.21901	3.14E-09
GGN	1.722256	3.17E-09
BOK	1.155301	3.19E-09
TMC8	0.441311	3.26E-09
FAM175A	0.382549	3.30E-09
TSPAN1	0.635432	3.34E-09
EN2	-0.36165	3.40E-09
UBE3A	-0.20532	3.42E-09
LPP-AS2	0.870877	3.44E-09
LOC1019269	0.951953	3.47E-09
AHCTF1	-0.23266	3.48E-09
KLF2	0.319034	3.49E-09
ATG4D	-0.26608	3.49E-09
ZBTB9	-0.36242	3.52E-09
ACOT13	0.422963	3.53E-09
ESPN	0.660298	3.55E-09
SMYD4	0.2968	3.57E-09
NAPG	-0.29117	3.58E-09
MOB1A	-0.39493	3.59E-09
NAV1	0.325311	3.59E-09
ALDH1A3	0.310332	3.65E-09
ZFP64	0.326852	3.65E-09
CHST13	0.870099	3.68E-09
BMS1P4	-0.3934	3.69E-09
TANC1	0.303573	3.73E-09
NUP62	-0.26524	3.74E-09
MORF4L2	-0.18121	3.80E-09

LAPTM4B	0.223884	3.82E-09
FMNL3	0.35557	3.84E-09
PSEN1	-0.25603	3.89E-09
CBY1	-0.40831	3.91E-09
HNRNPK	-0.15595	3.92E-09
TSHZ1	0.858579	3.93E-09
CHUK	-0.22766	3.93E-09
PEX11B	-0.40044	3.94E-09
CABP4	0.504225	3.96E-09
SMPD1	-0.39782	4.01E-09
DCP2	-0.31545	4.02E-09
SLC35E1	-0.26245	4.04E-09
TRAF6	-0.24762	4.09E-09
DNAJB5	0.701333	4.11E-09
SGTB	0.307493	4.12E-09
PTCD3	-0.20172	4.25E-09
RAB31	1.081792	4.29E-09
ZNF700	-0.34921	4.30E-09
ZSCAN12P1	0.751107	4.36E-09
DDX58	0.473382	4.39E-09
ALAD	0.25574	4.51E-09
DIEXF	-0.26157	4.51E-09
COL9A3	0.373877	4.55E-09
PLXNA2	0.672169	4.60E-09
NDUFAF5	-0.26472	4.61E-09
PRPF40A	-0.18199	4.61E-09
PAOX	0.827071	4.70E-09
SAMD9	0.476954	4.70E-09
DNAJB6	-0.20855	4.71E-09
SUCO	-0.28438	4.72E-09
FAM168B	-0.18486	4.74E-09
CD2BP2	-0.22808	4.78E-09
SSSCA1	-0.3684	4.99E-09
TMEM144	0.595794	5.00E-09
RAC1	-0.17915	5.08E-09
ABCG1	0.65518	5.10E-09
SDHD	-0.21231	5.16E-09
IFIT1	1.410294	5.20E-09
GRK5	0.434788	5.21E-09
IPO7	-0.19941	5.21E-09
MPP7	0.363488	5.21E-09
RNF115	-0.27417	5.26E-09
PPP2R3A	0.798152	5.29E-09
DNAJB14	-0.37229	5.29E-09
POFUT2	-0.42495	5.32E-09
CSRNP1	-0.24531	5.34E-09
RNPS1	-0.17408	5.34E-09

SEC23B	-0.20029	5.37E-09
PYGB	0.177738	5.37E-09
LINC00673	-0.18668	5.43E-09
SREK1IP1	-0.26519	5.43E-09
CHMP4B	-0.22267	5.44E-09
SMCR8	-0.32818	5.52E-09
NIPBL	-0.25567	5.53E-09
TAPT1	-0.27033	5.56E-09
DRG1	-0.26583	5.59E-09
GEMIN7	-0.4691	5.66E-09
FAM227A	0.724068	5.74E-09
RTF1	-0.2353	5.77E-09
STMN3	0.390876	5.78E-09
WDR1	-0.15897	5.92E-09
BLOC1S2	-0.2797	5.92E-09
MATN3	0.785088	5.96E-09
PRPF38A	-0.19031	6.04E-09
MAP3K3	-0.25297	6.07E-09
CLDN1	-0.1864	6.10E-09
F3	0.512201	6.12E-09
OBSCN	0.786959	6.13E-09
EIF5B	-0.19548	6.14E-09
CXADR	0.322553	6.27E-09
MDGA1	0.672743	6.27E-09
C1orf109	-0.31845	6.32E-09
CSRP2	0.265706	6.36E-09
INO80	-0.22439	6.40E-09
PROS1	0.401449	6.40E-09
RANBP3	-0.19892	6.55E-09
ADA	0.278512	6.59E-09
IK	-0.18462	6.64E-09
ARMC9	0.353272	6.71E-09
SPPL3	-0.34135	6.75E-09
NUPL2	-0.29773	6.76E-09
CAMKK1	0.455324	6.76E-09
PSKH1	-0.23441	6.83E-09
GJB1	0.244898	6.87E-09
CNTNAP3B	1.193946	6.88E-09
ZNF883	0.964981	6.95E-09
SLC4A7	-0.18594	6.97E-09
PRPF39	-0.24461	7.16E-09
EIF2B5	-0.20442	7.17E-09
TRIM4	-0.25282	7.21E-09
RHOBTB3	0.288135	7.23E-09
MFSD6	0.299519	7.28E-09
NFE2L1	-0.2039	7.43E-09
RAB32	0.459138	7.45E-09

ARNTL	0.351628	7.45E-09
EGFR	1.28833	7.47E-09
NOSIP	-0.2579	7.51E-09
MYNN	-0.33178	7.53E-09
LOC440173	0.997094	7.56E-09
YAF2	-0.27734	7.58E-09
PUF60	-0.17617	7.65E-09
RAD51AP1	-0.23016	7.70E-09
KIAA2026	-0.47518	7.71E-09
TPD52L1	0.315915	7.73E-09
TMC7	0.291433	7.89E-09
CDC40	-0.26555	7.92E-09
SV2A	0.450216	7.92E-09
TMED2	-0.19006	7.95E-09
ZSWIM1	-0.38097	8.05E-09
CNOT11	-0.2117	8.19E-09
MMD	0.588809	8.20E-09
GON4L	-0.20317	8.24E-09
SLC48A1	0.408454	8.25E-09
ANKRD13D	0.30375	8.25E-09
NOD1	0.527762	8.26E-09
ADNP2	-0.33258	8.27E-09
AKAP11	0.314917	8.35E-09
GOLGA2	-0.21756	8.35E-09
RTKN2	0.322555	8.48E-09
UBE3C	-0.21574	8.55E-09
SETD7	0.434098	8.55E-09
ARHGEF16	0.41069	8.64E-09
TIGD7	0.737604	8.69E-09
SOX13	0.206554	8.85E-09
RBM42	-0.26306	8.86E-09
EREG	0.295434	8.98E-09
ARAF	-0.23829	9.01E-09
ACP6	0.283646	9.03E-09
MRPL15	-0.2876	9.16E-09
MYEF2	0.254384	9.27E-09
ZEB1-AS1	0.624073	9.35E-09
CNN3	-0.18258	9.36E-09
NEDD4	0.250515	9.38E-09
CAPZA1	-0.17678	9.38E-09
GTSE1	-0.22778	9.56E-09
SMIM7	-0.23818	9.61E-09
POLR2E	-0.18923	9.92E-09
TMEM30A	-0.23806	9.92E-09
COL9A2	1.05449	9.95E-09
BAZ1B	-0.16854	1.01E-08
TP53	-0.20457	1.01E-08

DROSHA	-0.21622	1.02E-08
ZBED4	-0.26755	1.02E-08
PKIB	1.392483	1.03E-08
CHTF8	-0.1702	1.04E-08
MGAT1	-0.28922	1.04E-08
TUBB3	0.220137	1.05E-08
ASUN	-0.16734	1.07E-08
UPP1	0.240311	1.08E-08
AGA	0.382101	1.08E-08
ABHD14B	0.236243	1.08E-08
PIK3AP1	0.68964	1.08E-08
EIF4B	-0.16112	1.10E-08
ITGB1	-0.19689	1.11E-08
RNF39	0.625425	1.11E-08
DDX5	-0.19425	1.12E-08
SH3PXD2A	0.418544	1.13E-08
PRPF4B	-0.19658	1.13E-08
ARHGAP10	0.389741	1.14E-08
LOC493754	-0.30169	1.15E-08
TBX4	0.85511	1.15E-08
XPO1	-0.16026	1.15E-08
RAB11FIP3	0.313543	1.18E-08
PCSK6	0.428608	1.19E-08
SCRN1	-0.16946	1.19E-08
PSMC3	-0.1838	1.19E-08
C5orf24	-0.23625	1.19E-08
TPRN	-0.45874	1.21E-08
ATP2B4	0.791204	1.22E-08
RPGRIP1L	0.374844	1.27E-08
C9orf72	0.770805	1.27E-08
ALDH6A1	-0.30736	1.27E-08
SOX12	0.347534	1.31E-08
HTATSF1P2	0.462137	1.33E-08
BTAF1	-0.23576	1.33E-08
SLC39A8	0.532801	1.34E-08
CTNNBIP1	0.219987	1.35E-08
C17orf53	-0.39245	1.37E-08
SPTLC1	-0.19781	1.37E-08
SYNGAP1	0.508894	1.40E-08
ARID2	-0.24913	1.41E-08
RARB	1.071752	1.41E-08
LINC01116	0.87455	1.42E-08
NEK2	-0.23442	1.44E-08
RAPGEF6	-0.23911	1.45E-08
LEF1	0.551507	1.47E-08
C12orf43	-0.29358	1.47E-08
TNFAIP8L1	0.512528	1.48E-08

NAPEPLD	0.407682	1.48E-08
HOXA7	-0.31128	1.50E-08
CCDC151	1.252303	1.50E-08
SYNRG	-0.23457	1.50E-08
SLC22A5	0.399661	1.51E-08
LRRC37A11P	1.552387	1.52E-08
EIF4A3	-0.18323	1.53E-08
LCA5	0.399438	1.53E-08
RRP15	-0.25144	1.54E-08
MMP11	1.260498	1.56E-08
SRFBP1	-0.315	1.56E-08
ARMC1	-0.22609	1.56E-08
RNF138	-0.29154	1.57E-08
MORC2	0.316097	1.58E-08
FLCN	-0.48986	1.62E-08
HAUS6	-0.2278	1.63E-08
STAT5A	0.712579	1.63E-08
CUEDC1	0.240089	1.65E-08
ZC3H11A	-0.18874	1.66E-08
ALDH1A2	0.199648	1.66E-08
SSU72	-0.24884	1.69E-08
ZBTB42	1.179998	1.70E-08
DNMT3A	0.306558	1.70E-08
TRIB3	0.30618	1.70E-08
UBXN7	-0.27077	1.74E-08
FASLG	-0.59356	1.78E-08
ALDH1L2	1.172121	1.80E-08
LOC1019268	0.805303	1.81E-08
C12orf36	0.405049	1.82E-08
GOLGA3	-0.29235	1.83E-08
SNHG15	-0.25324	1.86E-08
SOS1	-0.24089	1.88E-08
FAM189A2	1.605156	1.89E-08
TBX6	1.205611	1.89E-08
AP4E1	-0.25114	1.90E-08
RHOF	0.284539	1.90E-08
TMBIM1	0.340878	1.91E-08
SH3BP1	0.28403	1.93E-08
HIST3H2BB	1.02605	1.95E-08
NHP2L1	-0.17588	2.00E-08
PIK3R4	-0.2496	2.01E-08
LRRC59	-0.18852	2.03E-08
SARNP	-0.22109	2.06E-08
HIST1H2AC	0.294775	2.08E-08
C3orf52	0.540287	2.08E-08
SATB1	0.538765	2.09E-08
KIFC1	-0.27026	2.10E-08

GOLGA2P7	0.359788	2.11E-08
NOA1	-0.24628	2.13E-08
FSTL4	0.402414	2.14E-08
RELT	-0.25851	2.15E-08
ZDHHC3	-0.20127	2.15E-08
DYRK1B	-0.33281	2.16E-08
ASRGL1	0.445254	2.17E-08
UBXN1	-0.19417	2.18E-08
MRPS12	-0.26063	2.21E-08
LONP2	-0.21322	2.21E-08
FASTKD2	-0.22297	2.22E-08
RHOG	-0.27023	2.26E-08
RAB11FIP4	0.393793	2.26E-08
EIF4H	-0.15515	2.28E-08
PPARA	1.038542	2.30E-08
THRAP3	-0.16498	2.30E-08
WHAMMP2	0.696406	2.30E-08
ELOF1	-0.23696	2.31E-08
SMU1	-0.2222	2.31E-08
FAM207A	-0.29879	2.31E-08
SH2D5	1.433271	2.31E-08
AUP1	-0.20102	2.31E-08
OTUD6B-AS1	-0.26867	2.33E-08
VPS4B	-0.3352	2.33E-08
MRPS7	-0.20835	2.35E-08
ASAP2	-0.23524	2.37E-08
TNPO2	-0.20752	2.42E-08
NFKBIA	-0.28953	2.45E-08
TMEM159	1.667288	2.47E-08
ZNF131	-0.23715	2.50E-08
DEDD	-0.22664	2.52E-08
NT5DC3	0.355055	2.52E-08
LOC285696	1.264229	2.53E-08
VPS53	-0.21862	2.54E-08
ELL2	0.217822	2.54E-08
PHF20L1	-0.23269	2.55E-08
FOSL2	-0.20136	2.56E-08
CDK5R1	0.358031	2.57E-08
IL17RA	0.263467	2.68E-08
EPHB3	0.219592	2.72E-08
JADE2	0.266841	2.78E-08
BAIAP3	0.56143	2.78E-08
BRMS1	-0.21609	2.78E-08
ARAP1	0.225608	2.78E-08
RPE	-0.25133	2.80E-08
FLJ37201	1.433092	2.82E-08
AAGAB	-0.19013	2.85E-08

PRRG1	0.266425	2.87E-08
LRRCC1	0.332429	2.88E-08
MAPK14	-0.22424	2.92E-08
FAM110C	0.374144	2.94E-08
RHNO1	-0.22254	2.95E-08
NR4A1	0.274205	2.95E-08
NOD2	1.047967	2.96E-08
MRPL38	-0.23282	2.98E-08
CHST7	0.656484	3.03E-08
RGS12	0.286352	3.06E-08
NIPA1	0.258571	3.06E-08
C3orf18	0.990634	3.06E-08
TMEM74B	1.077799	3.07E-08
FAM53A	0.889273	3.08E-08
RNF103	-0.38755	3.10E-08
HIST1H2AE	1.034169	3.11E-08
DNAJB11	-0.18116	3.13E-08
ZNF219	0.479113	3.14E-08
POLR2B	-0.19551	3.16E-08
ZFPL1	-0.28827	3.16E-08
ATAD2	-0.18278	3.17E-08
PCBP2	-0.14118	3.23E-08
MRPS23	-0.24879	3.23E-08
FAHD1	-0.3644	3.24E-08
HSP90AB1	-0.14564	3.25E-08
TRAPPC8	-0.27704	3.25E-08
CD82	0.367257	3.27E-08
FAM204A	-0.28619	3.28E-08
TRIAP1	-0.31161	3.28E-08
MEOX2	0.931165	3.30E-08
CREM	0.409348	3.30E-08
RAB3GAP1	-0.19578	3.38E-08
NXT1	-0.30876	3.38E-08
CXCL8	-0.27516	3.40E-08
UBAP2	-0.20415	3.44E-08
LEPRE1	-0.24876	3.46E-08
PTEN	-0.19211	3.48E-08
WDR78	0.867861	3.54E-08
PTPRB	1.501717	3.54E-08
WDR77	-0.23612	3.63E-08
ACBD4	0.531975	3.64E-08
C7orf49	-0.21531	3.67E-08
PSMA1	-0.20854	3.70E-08
DERL1	-0.25962	3.71E-08
METTL7A	0.731232	3.72E-08
GCNT2	0.437543	3.76E-08
LYPLA2	-0.1986	3.79E-08

SDPR	-0.19891	3.80E-08
SEMA4C	-0.37484	3.80E-08
LINC00667	0.374627	3.82E-08
CAND1	-0.17388	3.82E-08
CYP2S1	0.288252	3.85E-08
PITX2	0.390751	3.87E-08
C1orf174	-0.40658	3.89E-08
RNF128	0.260862	3.89E-08
TCF7L1	0.658166	3.90E-08
TIMM23	-0.23054	3.90E-08
LINC01128	-0.39731	3.98E-08
TTPA	1.057231	3.98E-08
PIP5K1C	0.267687	4.04E-08
EDC3	-0.20696	4.08E-08
FBXW5	-0.20519	4.09E-08
TBC1D31	0.283452	4.12E-08
RHOQ	0.672963	4.14E-08
MAP2K3	-0.22735	4.22E-08
SMIM5	1.564215	4.23E-08
MTMR11	0.697647	4.24E-08
UAP1L1	0.32751	4.24E-08
STAT2	-0.25183	4.28E-08
CERCAM	0.517825	4.32E-08
ERGIC2	-0.32233	4.42E-08
FAIM2	1.527533	4.47E-08
GTF3C6	-0.24522	4.47E-08
C11orf84	-0.18128	4.48E-08
RBPMS	-0.26039	4.50E-08
TMCO3	-0.22659	4.63E-08
MTL5	0.541196	4.64E-08
MAGEB2	0.409691	4.68E-08
KRTAP2-3	0.476444	4.72E-08
NFKB1	-0.28277	4.77E-08
MICAL3	0.227826	4.78E-08
MYL12A	-0.16009	4.79E-08
SYS1	-0.2485	4.83E-08
SMAGP	0.2882	4.83E-08
NUDT18	0.516483	4.84E-08
ARHGEF17	0.227648	4.88E-08
TOX3	0.215565	4.92E-08
AKAP3	1.361462	5.03E-08
LOC285074	-0.4205	5.03E-08
GNAI3	-0.19433	5.05E-08
SLMO2	-0.20919	5.08E-08
DDX56	-0.19848	5.10E-08
SNX13	-0.28994	5.11E-08
FGD1	0.83417	5.16E-08

ATP6V0A2	-0.26052	5.19E-08
PAK1IP1	-0.22098	5.25E-08
NLGN2	0.312188	5.28E-08
PON2	0.213713	5.28E-08
CLASP1	-0.18806	5.31E-08
HOXC11	0.889615	5.38E-08
C12orf5	-0.25942	5.43E-08
PRR7	0.405355	5.46E-08
SYF2	-0.28189	5.51E-08
LUC7L3	-0.20785	5.55E-08
PPP1R12A	-0.16997	5.55E-08
RNASEH1	-0.28911	5.63E-08
SRSF5	-0.17474	5.63E-08
ELAVL1	-0.17761	5.65E-08
SIAH1	-0.30305	5.65E-08
ATP6V1H	-0.2611	5.70E-08
ARRDC3-AS1	0.841752	5.72E-08
DUS3L	-0.22685	5.75E-08
ZGPAT	-0.28426	5.78E-08
FAM177A1	-0.30221	5.80E-08
ZSCAN29	-0.25098	5.84E-08
HIST1H2AI	1.149356	5.86E-08
U2AF2	-0.16112	5.88E-08
EWSR1	-0.15009	5.89E-08
HS3ST5	1.357518	5.91E-08
GFOD1	0.748517	5.95E-08
DOPEY2	0.287012	5.98E-08
SLC7A5	-0.15811	6.01E-08
GTF3C3	-0.22643	6.01E-08
GOLT1A	0.632127	6.09E-08
DCAF5	-0.20157	6.10E-08
ARMC8	-0.31832	6.10E-08
LOC145783	1.035296	6.12E-08
RAD23A	-0.16732	6.16E-08
PRDM11	0.445894	6.22E-08
MAU2	-0.22494	6.23E-08
NAA15	-0.21877	6.23E-08
ZC3HC1	-0.23927	6.23E-08
RBMX	-0.15242	6.27E-08
MED23	-0.22102	6.27E-08
SEMA3C	0.639161	6.28E-08
DHX29	-0.20768	6.29E-08
ETV7	1.549871	6.34E-08
RRP1B	-0.20464	6.39E-08
DBR1	-0.24758	6.50E-08
RAET1L	0.806356	6.50E-08
IL10RB	0.340699	6.53E-08

ZFAS1	-0.16906	6.56E-08
PEX12	-0.42937	6.59E-08
C5orf15	-0.19102	6.68E-08
ZNF396	1.18335	6.69E-08
C10orf88	-0.31025	6.75E-08
RSPH1	1.414987	6.75E-08
SPG7	-0.31521	6.75E-08
C19orf66	0.601533	6.80E-08
FOXJ3	-0.26077	6.80E-08
LINC00886	0.555373	6.85E-08
SMPD2	0.389431	6.90E-08
CCDC112	0.276781	6.93E-08
DOK1	0.336447	6.94E-08
RBM26-AS1	0.650866	6.97E-08
PLEKHA5	0.23499	6.99E-08
GSK3B	-0.259	7.05E-08
OTUD5	-0.18095	7.11E-08
RNU86	-0.52061	7.12E-08
C6orf15	0.343018	7.15E-08
MAPKAPK3	0.203186	7.25E-08
LINC00337	1.305321	7.30E-08
HIST1H4H	0.77624	7.31E-08
TAF6L	-0.28455	7.42E-08
SLC16A13	0.865763	7.42E-08
RRP36	-0.21571	7.43E-08
ZNF484	-0.42102	7.48E-08
CHAC2	0.474547	7.54E-08
ATP1A1-AS1	0.979332	7.57E-08
YWHAZ	-0.14581	7.58E-08
FAM192A	-0.17921	7.70E-08
NIPSNAP3B	1.569937	7.74E-08
ESF1	-0.20063	7.79E-08
IRAK1BP1	0.569877	7.82E-08
GOLGA7B	0.72249	7.89E-08
ATP1A1	0.156855	7.99E-08
LAMB1	0.228828	8.03E-08
OLFML3	0.479416	8.06E-08
HELQ	-0.30672	8.07E-08
TMEM154	1.186418	8.11E-08
CTSV	0.47334	8.28E-08
RGL3	0.363663	8.32E-08
CKB	0.342627	8.34E-08
SMIM12	-0.326	8.43E-08
GPATCH2	-0.3802	8.44E-08
TMPRSS3	0.521994	8.47E-08
LZTS3	0.627	8.48E-08
TRAM2-AS1	0.482578	8.49E-08

PCSK1N	0.52645	8.50E-08
CDK18	0.64681	8.51E-08
ILF2	-0.15999	8.60E-08
GAP43	0.463925	8.63E-08
KDELC2	0.211725	8.72E-08
STYX	-0.34345	8.85E-08
GSE1	-0.17207	9.08E-08
WDR41	0.273208	9.08E-08
NDOR1	-0.31958	9.08E-08
ZNF711	0.232823	9.08E-08
LYSMD2	0.568661	9.09E-08
LMO4	0.280563	9.10E-08
ZNF554	0.964014	9.12E-08
ADCK4	0.297989	9.29E-08
IRX5	0.442241	9.29E-08
LOC93622	0.398957	9.35E-08
ARPP19	-0.1989	9.64E-08
COMMD2	-0.23319	9.65E-08
MRPL9	-0.18639	9.77E-08
SNRNP27	-0.2365	9.84E-08
GAS6	-0.27286	9.84E-08
FAM89A	0.60949	9.84E-08
ENO4	1.382026	9.91E-08
IGIP	0.895123	9.94E-08
FCHO1	0.37849	9.95E-08
MGC57346	0.429842	1.01E-07
ATXN2	-0.19602	1.02E-07
GAK	-0.18412	1.03E-07
MYCBP2	0.226925	1.03E-07
OPRL1	1.252263	1.03E-07
IMP3	-0.26157	1.05E-07
HELZ2	0.317425	1.06E-07
BCL2L15	0.258764	1.06E-07
TCAIM	0.303477	1.07E-07
LINC01011	1.02906	1.07E-07
KEAP1	-0.2445	1.10E-07
DENND1B	0.44506	1.12E-07
UBAC2-AS1	0.889755	1.12E-07
BEX5	0.551696	1.13E-07
PRAF2	0.289276	1.16E-07
RRAGC	-0.3626	1.16E-07
PLCG2	0.738688	1.16E-07
EPT1	-0.20135	1.16E-07
TXNL4B	-0.22275	1.17E-07
CBR4	0.289392	1.17E-07
CCDC125	0.340864	1.18E-07
LINC00649	0.425092	1.18E-07

TPX2	-0.14841	1.19E-07
METTL1	-0.29537	1.19E-07
DHX32	-0.27762	1.20E-07
NUDT19	-0.26079	1.21E-07
DOLK	-0.54385	1.21E-07
FLII	-0.16198	1.21E-07
LINC00491	0.377768	1.23E-07
RHOBTB2	0.302141	1.25E-07
EIF5A2	0.342876	1.25E-07
KLHL21	0.269588	1.25E-07
MCFD2	-0.18861	1.25E-07
RFFL	0.635753	1.26E-07
HARS	-0.18251	1.26E-07
ADAM17	-0.28333	1.26E-07
MTMR12	-0.21332	1.27E-07
COPA	-0.15423	1.28E-07
FLVCR2	0.905915	1.30E-07
ZMAT2	-0.18667	1.30E-07
ERAL1	-0.18371	1.31E-07
SUMF2	0.18278	1.32E-07
SESN2	0.361834	1.32E-07
LOC643406	0.602766	1.33E-07
ZNF547	-0.67755	1.33E-07
MFSD11	-0.28338	1.34E-07
FGFR1	0.20746	1.34E-07
METTL14	-0.31897	1.34E-07
GABRR1	0.939146	1.35E-07
HSD11B2	0.31043	1.36E-07
RPS6KA1	0.241353	1.36E-07
ANTXR1	0.255176	1.37E-07
FAIM3	1.397958	1.38E-07
FAM199X	-0.1903	1.38E-07
GFER	-0.23409	1.39E-07
TRMT11	-0.28571	1.40E-07
OSBPL5	0.383944	1.41E-07
RPL14	-0.16332	1.41E-07
OAF	0.317432	1.41E-07
FJX1	-0.26732	1.41E-07
MCAT	-0.28668	1.42E-07
NFIL3	0.286857	1.43E-07
PPIG	-0.23967	1.43E-07
GNA12	0.229568	1.44E-07
SLC25A29	0.340396	1.45E-07
SLC35D2	-0.24948	1.47E-07
RNF38	-0.44389	1.48E-07
FAM53C	-0.17616	1.48E-07
MAP7D3	0.226179	1.52E-07

HAUS4	0.388752	1.52E-07
NDUFA12	-0.21049	1.52E-07
PNPLA6	0.29291	1.52E-07
DMTF1	-0.19589	1.54E-07
CD109	0.215312	1.55E-07
TBX15	0.862207	1.55E-07
THNSL1	0.477573	1.57E-07
FAM111A	0.393926	1.58E-07
PIGC	-0.33189	1.60E-07
TCEA2	0.370218	1.61E-07
NXPH4	1.277955	1.61E-07
TMEM106C	0.178963	1.61E-07
CYLD	-0.32176	1.62E-07
TIFA	0.619202	1.66E-07
PITX1	-0.28369	1.66E-07
GDI2	-0.15087	1.66E-07
CLSTN1	0.189495	1.68E-07
KLF5	-0.14339	1.68E-07
LINC01184	0.468001	1.69E-07
RASD1	0.539553	1.72E-07
RALGAPA2	0.323493	1.72E-07
VWA5A	0.442698	1.73E-07
LINC00847	0.772518	1.74E-07
YWHAB	-0.15623	1.74E-07
SH3BP5L	-0.35424	1.76E-07
LBX2-AS1	0.665388	1.77E-07
MAPKAPK2	-0.18099	1.77E-07
COPS8	-0.18868	1.78E-07
CCDC47	-0.15502	1.78E-07
TMEM109	0.18956	1.79E-07
MTMR2	-0.16628	1.79E-07
RPARP-AS1	0.675687	1.80E-07
KCTD1	0.408473	1.81E-07
CRB2	1.116903	1.83E-07
LINC01106	0.72976	1.85E-07
IL10	1.155352	1.85E-07
VPS37D	1.516075	1.87E-07
MPZL2	0.397894	1.88E-07
POLR3E	-0.25362	1.88E-07
KLB	1.117177	1.88E-07
ANKRD17	-0.17628	1.88E-07
ZNF44	-0.32461	1.89E-07
PRKAR1A	-0.16684	1.90E-07
CIC	-0.30496	1.91E-07
FKBP4	0.177537	1.93E-07
KIAA1671	0.209257	1.94E-07
UBE2Q1	-0.16741	1.95E-07

UBA2	-0.17578	1.96E-07
KIF16B	0.267673	1.98E-07
PSAT1	0.22368	1.99E-07
SZRD1	-0.1534	2.00E-07
CARD11	0.784399	2.01E-07
IWS1	-0.19842	2.03E-07
FAM206A	-0.21483	2.06E-07
CTNNB1	-0.1541	2.07E-07
TXLNB	1.36651	2.08E-07
NAT10	-0.17145	2.09E-07
UBE2C	-0.16206	2.10E-07
ALKBH3	-0.28455	2.10E-07
RPP25L	0.33479	2.12E-07
ZSCAN32	-0.29439	2.12E-07
WDR37	-0.35802	2.12E-07
RNF167	-0.18285	2.14E-07
NDUFAF7	-0.32099	2.15E-07
HLA-F	0.872186	2.16E-07
ZBTB25	0.784182	2.17E-07
PTK6	0.435872	2.21E-07
HNRNPH1	-0.16141	2.22E-07
TEAD4	-0.21125	2.23E-07
ZNF75A	0.279296	2.23E-07
GINS1	-0.25231	2.24E-07
KALRN	0.806005	2.28E-07
ATF1	-0.2622	2.30E-07
MTMR1	-0.24841	2.33E-07
DCAF10	-0.22654	2.34E-07
FRK	0.523689	2.35E-07
PGS1	-0.22523	2.35E-07
SMKR1	0.895642	2.35E-07
SNRPB2	-0.17742	2.36E-07
FKBP1B	0.75908	2.36E-07
ZNF507	-0.25561	2.37E-07
GPR153	0.398831	2.38E-07
LPP	0.278293	2.40E-07
ARL8B	-0.19909	2.40E-07
GPR173	0.836112	2.41E-07
EFHC1	0.310864	2.42E-07
ZFYVE16	-0.20041	2.42E-07
TMEM14A	0.379955	2.45E-07
TNFRSF9	1.246169	2.46E-07
TMEM44	0.350544	2.47E-07
HERC1	0.250773	2.47E-07
PLEKHG5	0.40819	2.49E-07
REXO4	-0.21718	2.53E-07
MAPT	1.145253	2.54E-07

HDGF	-0.13926	2.54E-07
PSMB9	1.205774	2.56E-07
FOPNL	-0.26254	2.59E-07
ZNF526	-0.39707	2.59E-07
ASNS	0.257007	2.59E-07
EIF4ENIF1	-0.31572	2.60E-07
PIKFYVE	-0.27135	2.60E-07
ATXN7L2	-0.39408	2.62E-07
PRKRIR	-0.20095	2.63E-07
MEX3C	-0.33007	2.65E-07
ZNF607	0.567422	2.68E-07
ZNF614	-0.37648	2.71E-07
GRIN2D	0.811461	2.71E-07
ASB2	0.592648	2.72E-07
METTL7B	0.276219	2.77E-07
ZNF543	-0.53314	2.79E-07
CEP104	-0.28415	2.81E-07
MED27	-0.28482	2.87E-07
CCL28	0.54479	2.87E-07
CDCA4	-0.2761	2.88E-07
ITGB6	0.838979	2.90E-07
CA12	1.408909	2.95E-07
LOC284412	0.542334	2.98E-07
AHR	0.179931	2.98E-07
PPP2R5E	-0.21062	2.98E-07
IGF2R	0.181571	3.02E-07
MIR3648	0.734532	3.03E-07
NSA2	-0.18715	3.06E-07
PIAS1	-0.2943	3.08E-07
ISG20	0.914439	3.11E-07
SLC15A2	0.748537	3.12E-07
ADAMTS20	0.915918	3.13E-07
CSPP1	-0.25048	3.13E-07
TRUB2	-0.22416	3.15E-07
NUDCD3	-0.20514	3.15E-07
SLAIN2	-0.20858	3.21E-07
TCP1	-0.22794	3.24E-07
SNW1	-0.16321	3.25E-07
ADAM10	-0.18661	3.29E-07
MID1IP1	0.202823	3.30E-07
ZNF330	-0.27228	3.31E-07
ZNRF3	-0.19942	3.32E-07
EPHA10	0.575638	3.34E-07
ACTN1	-0.14185	3.34E-07
CORO6	0.807203	3.35E-07
NTSR1	0.231475	3.36E-07
FAM13B	-0.21477	3.36E-07

NOP14-AS1	-0.45312	3.37E-07
CCDC103	-0.70525	3.39E-07
ZBED5-AS1	0.398441	3.40E-07
GPX7	0.58019	3.41E-07
GOLGA7	-0.23972	3.42E-07
POU2F2	0.584728	3.43E-07
SMURF2	-0.30355	3.43E-07
RYR1	1.357053	3.43E-07
CDC5L	-0.17547	3.45E-07
PANX1	-0.32045	3.49E-07
SLC6A6	0.224402	3.50E-07
SLC7A2	1.349627	3.50E-07
PPME1	-0.17747	3.50E-07
CPSF2	-0.19661	3.51E-07
PKD2	0.304171	3.52E-07
LINC00999	0.370155	3.58E-07
POP7	-0.23198	3.60E-07
CDC42EP3	-0.17973	3.63E-07
LHFPL2	0.35875	3.65E-07
SULT1C2	0.638868	3.65E-07
THAP9-AS1	-0.234	3.67E-07
HNRNPA1L2	-0.38183	3.68E-07
CCL20	-0.6842	3.71E-07
ACKR2	1.375503	3.74E-07
LINC00511	0.370678	3.77E-07
FRY	1.067212	3.78E-07
UNC5CL	0.51854	3.78E-07
MBOAT1	0.522086	3.80E-07
E4F1	-0.31127	3.80E-07
NPM2	0.681146	3.82E-07
ITPK1	0.18707	3.82E-07
SUFU	0.287691	3.83E-07
PFKP	0.167063	3.83E-07
SBNO2	-0.19461	3.84E-07
WRAP73	0.274672	3.91E-07
GLTSCR1	-0.34035	3.96E-07
TBCCD1	-0.34289	3.99E-07
LRRC47	-0.20492	4.01E-07
CTDSPL2	-0.29754	4.02E-07
PBX4	0.424412	4.03E-07
GNB3	1.334204	4.06E-07
LOC286437	0.655475	4.06E-07
ZNF230	0.420313	4.07E-07
LUC7L	-0.29627	4.08E-07
MRPS18A	-0.23588	4.10E-07
CSNK2B	-0.18756	4.10E-07
NAT14	0.295508	4.11E-07

RBM38	0.233845	4.11E-07
HIST1H2BF	0.973437	4.14E-07
IMP4	-0.23391	4.14E-07
ALDH3A2	0.238211	4.17E-07
FAM86JP	0.654269	4.19E-07
PSMC1	-0.16593	4.22E-07
C12orf75	0.301841	4.24E-07
AP2A1	-0.21442	4.27E-07
PRMT5	-0.1619	4.32E-07
FAM83G	0.231784	4.33E-07
SGOL1	-0.3421	4.33E-07
GTPBP1	-0.23182	4.33E-07
NUFIP1	-0.27567	4.33E-07
FMN1	0.284726	4.34E-07
VWA1	0.252409	4.35E-07
HACL1	-0.36779	4.37E-07
SHC3	0.362103	4.38E-07
ATP6V1E1	-0.18201	4.40E-07
POPDC3	0.844245	4.42E-07
SIK3	-0.18291	4.46E-07
MEX3A	0.248348	4.47E-07
SEZ6L2	0.427208	4.47E-07
NDUFB9	-0.24743	4.50E-07
CLN5	0.242432	4.51E-07
TOLLIP	-0.25246	4.56E-07
NUP160	-0.17851	4.59E-07
ASNSD1	-0.20294	4.64E-07
USP16	-0.25031	4.68E-07
PSMD7	-0.1667	4.78E-07
ZNF818P	0.97001	4.80E-07
HLA-DQB1	0.907838	4.82E-07
WDR31	1.540465	4.84E-07
BBS10	0.255932	4.98E-07
GPN1	-0.21011	5.02E-07
FSCN1	0.174305	5.06E-07
RPS19BP1	-0.23149	5.08E-07
AMN1	0.471005	5.18E-07
MAP3K8	0.612976	5.24E-07
CCT6P1	-0.30933	5.25E-07
MADD	-0.16834	5.30E-07
MRPS31P5	-0.33678	5.33E-07
MORC3	-0.21865	5.34E-07
WDR62	-0.1825	5.35E-07
FUBP3	-0.18049	5.35E-07
HIST1H3H	0.801974	5.36E-07
RTCB	-0.17775	5.41E-07
CHRNB1	-0.18058	5.48E-07

IL15RA	1.282445	5.51E-07
USP39	-0.15682	5.52E-07
FAM86FP	0.496314	5.55E-07
ST3GAL4	0.37543	5.59E-07
PTCHD2	0.652278	5.63E-07
NARG2	-0.21671	5.64E-07
LPCAT1	-0.22219	5.65E-07
HNF1A	0.259198	5.72E-07
HDHD3	0.452932	5.73E-07
RSL24D1	-0.19823	5.75E-07
ASPRV1	0.818694	5.77E-07
ZFP28	0.620794	5.77E-07
HGD	0.509399	5.82E-07
HIP1	0.202398	5.84E-07
ZC4H2	0.502816	5.89E-07
MRPS31	-0.26511	5.90E-07
WDR54	0.341456	5.91E-07
CC2D1A	0.178793	5.94E-07
ZNF781	1.137742	5.94E-07
TRNT1	-0.25189	6.00E-07
ZDHHC17	-0.24013	6.02E-07
TUBA3FP	0.805023	6.05E-07
SAMD4A	0.362533	6.07E-07
HNRNPM	-0.13735	6.10E-07
CDC123	-0.17378	6.11E-07
FUT8	0.258987	6.19E-07
CACNA1F	0.748454	6.30E-07
PARP9	0.425247	6.33E-07
NFYB	-0.21149	6.35E-07
SLC17A5	0.351285	6.40E-07
SNAP47	-0.35187	6.47E-07
CLIP4	1.00621	6.48E-07
E2F2	0.454282	6.53E-07
DDX60L	0.855732	6.53E-07
GPS2	-0.22091	6.59E-07
SF3A2	-0.22766	6.66E-07
VWA9	-0.2144	6.75E-07
FAM171A1	0.89096	6.76E-07
NMU	1.233148	6.78E-07
HNRNPC	-0.13826	6.85E-07
SIX2	1.413287	6.90E-07
DDX41	-0.19711	6.91E-07
CREBBP	-0.20821	6.94E-07
KHDRBS1	-0.14356	6.97E-07
PCGF2	0.266546	6.98E-07
TRIM26	-0.22857	7.02E-07
ABHD10	0.263298	7.06E-07

CHD9	0.242016	7.07E-07
HIST1H2BC	0.338662	7.10E-07
DDX24	-0.14843	7.10E-07
IMPAD1	-0.1747	7.11E-07
IFIT2	0.766134	7.13E-07
ALAS1	-0.19589	7.14E-07
NFKB2	0.264696	7.15E-07
NUP133	-0.19835	7.20E-07
NMT1	-0.15181	7.28E-07
ARID5B	-0.26595	7.28E-07
TAF1	-0.1814	7.34E-07
TMEM65	0.443197	7.37E-07
HOXC8	0.399935	7.45E-07
VEZT	-0.18861	7.55E-07
CSNK2A1	-0.16084	7.59E-07
NOLC1	-0.15404	7.63E-07
RTCA	-0.23888	7.65E-07
MMP7	1.489049	7.66E-07
IQCH-AS1	0.655324	7.68E-07
GPT2	0.185949	7.68E-07
VAT1	0.220232	7.75E-07
LRRC8E	0.364826	7.80E-07
NCOA2	0.29183	7.85E-07
ZNF2	-0.3676	7.96E-07
DUSP11	-0.28797	7.98E-07
RBM18	-0.21613	7.99E-07
HES1	0.270804	7.99E-07
AP1B1	-0.16077	8.15E-07
AADAT	0.244657	8.20E-07
GPR155	0.248852	8.31E-07
DENND4A	-0.23063	8.35E-07
POU2F3	1.384547	8.35E-07
NUDT21	-0.16311	8.47E-07
PSMD2	-0.14341	8.53E-07
TRAFD1	-0.1918	8.65E-07
SELENBP1	0.76474	8.66E-07
ARHGAP17	-0.21364	8.68E-07
FAM134C	-0.33697	8.76E-07
KIAA1432	-0.34324	8.76E-07
GTPBP10	-0.24572	8.81E-07
KLHL29	0.881637	8.81E-07
EIF1AX	-0.1773	8.82E-07
AASS	0.386221	8.85E-07
BSN-AS2	1.206476	8.85E-07
UBXN4	-0.15507	8.85E-07
LRRC73	1.415552	8.86E-07
MDFI	0.369462	8.93E-07

DUSP9	0.909625	9.07E-07
HOOK1	0.288216	9.07E-07
ZER1	-0.26128	9.10E-07
PARD6G	0.951545	9.38E-07
KCNH6	0.92161	9.42E-07
LPPR2	0.611585	9.43E-07
TOM1L1	0.225858	9.54E-07
USP21	-0.28252	9.55E-07
GABARAPL1	0.683092	9.57E-07
C19orf43	-0.15975	9.61E-07
MSANTD3	-0.25595	9.62E-07
PAN3	-0.25743	9.69E-07
FAM120AOS	-0.25286	9.73E-07
EGLN1	-0.21272	9.80E-07
NPAS2	0.318162	9.81E-07
TOMM40	-0.16412	9.83E-07
SUCLA2	-0.2389	9.92E-07
TYW3	-0.20808	9.96E-07
H2AFY2	0.167343	1.01E-06
CASP8	0.492762	1.01E-06
DNAJC13	-0.20514	1.01E-06
FAM69A	0.375208	1.02E-06
AGFG1	-0.17244	1.03E-06
ARFIP1	-0.21359	1.03E-06
ZSWIM3	-0.34099	1.03E-06
LYSMD3	-0.21265	1.05E-06
LIPT2	0.531947	1.05E-06
ARNTL2	0.225845	1.06E-06
CIDECP	-0.34582	1.06E-06
NAT1	-0.332	1.06E-06
KAT6B	-0.22109	1.07E-06
HCG11	-0.3529	1.07E-06
CHRNB2	0.983409	1.08E-06
OPA1	-0.17548	1.09E-06
TXNDC11	-0.1916	1.10E-06
MPDU1	-0.17793	1.10E-06
ELK4	-0.26202	1.11E-06
VPS37A	-0.24888	1.11E-06
MRPL14	-0.34715	1.11E-06
P2RX4	0.289944	1.12E-06
C1orf216	0.415492	1.12E-06
PLEKHB1	0.170923	1.14E-06
PGAM5	-0.17096	1.14E-06
HYOU1	-0.15187	1.16E-06
SQRDL	0.261623	1.17E-06
SSUH2	1.332405	1.17E-06
PAXIP1-AS1	0.399158	1.18E-06

RPL7L1	-0.18163	1.18E-06
TANC2	0.18207	1.19E-06
USP9X	-0.21547	1.20E-06
TMEM212	0.47445	1.20E-06
MLLT11	0.2227	1.20E-06
RASSF4	0.354574	1.20E-06
RCAN1	0.373442	1.21E-06
RIN3	0.318313	1.21E-06
SPEG	1.196381	1.21E-06
CEP85L	0.460169	1.21E-06
TBX2	-0.28017	1.21E-06
PLEKHM2	-0.22556	1.22E-06
CASC3	-0.16078	1.23E-06
SDHAP1	-0.46942	1.23E-06
ZMYM5	-0.23951	1.28E-06
IDH2	0.213793	1.28E-06
ARHGAP5	-0.19366	1.29E-06
RFXAP	0.423921	1.30E-06
PDIK1L	-0.31127	1.30E-06
CDK11A	-0.36569	1.30E-06
KIF24	-0.26524	1.30E-06
TVP23B	-0.46848	1.31E-06
CC2D1B	-0.19855	1.32E-06
CPT2	-0.21308	1.33E-06
ACTR3	-0.16594	1.36E-06
COTL1	0.197608	1.38E-06
FOXRED2	0.20424	1.38E-06
KBTBD4	-0.26059	1.39E-06
CDT1	-0.26267	1.39E-06
ZNF292	-0.2662	1.41E-06
CGREF1	0.2496	1.41E-06
TNRC6A	-0.18776	1.41E-06
MMP14	0.598492	1.41E-06
SOCS5	-0.40865	1.42E-06
PSMD1	-0.15077	1.42E-06
GALK1	0.382591	1.43E-06
BUB1B	-0.178	1.43E-06
FLNA	-0.14951	1.44E-06
PPFIA1	-0.14847	1.45E-06
SNED1	1.038639	1.45E-06
TMEM259	-0.23926	1.46E-06
PPAP2C	0.274412	1.46E-06
TRIM39	-0.33628	1.47E-06
BAHCC1	0.450341	1.47E-06
KATNBL1	-0.36124	1.49E-06
ANKRD34A	0.670672	1.49E-06
ITGAV	0.257758	1.49E-06

ZDHHC13	0.252115	1.50E-06
PRB3	1.123718	1.50E-06
TAF7	-0.19975	1.51E-06
VSIG1	0.574055	1.51E-06
CTSH	0.497365	1.52E-06
TMED10	-0.1562	1.53E-06
ZNF271	-0.28962	1.53E-06
USP37	-0.26076	1.53E-06
PPM1J	1.070239	1.54E-06
RRP1	-0.22226	1.55E-06
STK24	-0.13475	1.55E-06
DUSP19	0.764728	1.55E-06
GTF3C1	-0.17159	1.57E-06
SLC25A16	-0.33167	1.57E-06
ARMC6	-0.2089	1.58E-06
CDC34	-0.19317	1.58E-06
MAP4K4	0.173198	1.58E-06
LINC00963	0.422478	1.58E-06
MRPL22	-0.21588	1.60E-06
POLG	-0.19142	1.60E-06
PP7080	0.349567	1.60E-06
TNNI3	1.275144	1.61E-06
VPS33A	-0.22276	1.61E-06
NAF1	-0.27361	1.62E-06
MRPL1	-0.23584	1.62E-06
CSTF2	-0.18052	1.62E-06
SNRPA1	-0.19971	1.62E-06
DENND2C	1.376981	1.63E-06
RBM10	-0.1499	1.64E-06
BOLA1	0.562641	1.64E-06
PVR	0.159316	1.65E-06
SLC25A37	0.321229	1.65E-06
CSPG4	1.181095	1.65E-06
LOC1019271	0.521358	1.65E-06
RBL1	0.166738	1.66E-06
CNTNAP3	0.513136	1.69E-06
WSB1	-0.25143	1.70E-06
AGPAT4	1.262569	1.71E-06
LOC1019297	0.784703	1.72E-06
DYNC1I1	0.894362	1.73E-06
ANXA11	-0.13604	1.74E-06
PAK4	-0.19388	1.74E-06
PLCB3	0.247216	1.77E-06
BEAN1	0.542946	1.77E-06
PANK1	-0.3313	1.79E-06
OGFRL1	0.434158	1.79E-06
TBC1D16	0.176231	1.81E-06

SYT17	0.503323	1.83E-06
RBM4	-0.2834	1.86E-06
TAF3	-0.21549	1.88E-06
ZNF563	0.639675	1.89E-06
RCAN3	0.436964	1.95E-06
CDK5RAP1	-0.20117	1.96E-06
NLRP11	1.270081	1.96E-06
HLTF	0.24049	1.97E-06
HFM1	0.687588	1.98E-06
PAK2	-0.16175	1.98E-06
AQP11	1.180236	1.99E-06
TMEM8A	-0.21874	1.99E-06
SNHG12	-0.21486	1.99E-06
CUL4B	-0.16279	2.01E-06
ANKFY1	-0.17538	2.04E-06
MIA3	-0.18998	2.04E-06
TTYH2	0.507984	2.04E-06
SCAF11	-0.18562	2.06E-06
SDSL	0.661911	2.06E-06
LRRFIP2	-0.21269	2.09E-06
MAPK12	0.314095	2.11E-06
XKR8	0.445965	2.12E-06
SLC16A4	0.575641	2.14E-06
GRB2	-0.1701	2.15E-06
CTPS1	-0.16037	2.17E-06
ATP6V1G1	-0.21593	2.17E-06
MAGT1	-0.19397	2.18E-06
MRPS17	-0.21569	2.18E-06
PCDHGC3	0.392	2.19E-06
STIP1	-0.12437	2.20E-06
AP5Z1	-0.24199	2.23E-06
CDC27	-0.16823	2.23E-06
PLCD3	0.172239	2.23E-06
VWA2	0.253408	2.24E-06
C5	0.412476	2.26E-06
PHGDH	0.301752	2.27E-06
SSR1	-0.16536	2.27E-06
GDAP2	-0.30396	2.28E-06
GAR1	-0.35052	2.28E-06
TXN2	-0.19897	2.28E-06
CHAF1A	-0.20784	2.32E-06
SPATA2L	0.417394	2.32E-06
RIPPLY3	0.998125	2.33E-06
RC3H2	-0.27502	2.34E-06
DPP9	-0.16837	2.34E-06
BTBD16	0.456428	2.38E-06
CGRRF1	-0.41716	2.38E-06

C11orf58	-0.16562	2.39E-06
UHRF1	0.158111	2.39E-06
EIF3F	-0.16129	2.41E-06
RNF166	-0.3011	2.42E-06
BUB1	-0.1713	2.42E-06
TTC7A	0.23329	2.44E-06
IL20RA	0.386193	2.46E-06
NT5C2	-0.15911	2.46E-06
DESI1	-0.18317	2.46E-06
ANXA5	-0.14033	2.46E-06
ELF1	-0.19706	2.49E-06
LOC389641	0.395759	2.49E-06
PSMD3	-0.14785	2.50E-06
FMO3	1.148132	2.51E-06
MAML2	0.216351	2.52E-06
FAM83D	-0.24185	2.55E-06
MAOB	1.115632	2.56E-06
ST6GALNAC5	0.665432	2.56E-06
CYP3A5	0.811566	2.57E-06
TDRKH	0.430864	2.59E-06
HIVEP2	0.238384	2.60E-06
FLJ20021	0.757285	2.60E-06
VPRBP	-0.19242	2.61E-06
XPA	-0.2565	2.61E-06
PRR5	0.462568	2.65E-06
CORO2B	0.99707	2.67E-06
LINC01270	1.039586	2.71E-06
AIFM2	0.162942	2.71E-06
DNAJC7	-0.16115	2.71E-06
MLLT1	-0.17248	2.72E-06
GABBR1	0.488838	2.74E-06
EIF5A	-0.12445	2.78E-06
HEATR5A	0.251318	2.78E-06
CDC6	-0.1582	2.81E-06
SYNGR1	0.499378	2.81E-06
TARS	-0.14039	2.83E-06
DHRS4-AS1	0.276177	2.85E-06
WDR17	0.494052	2.86E-06
EPB41L4A-AS	-0.31001	2.89E-06
BAG6	-0.14065	2.89E-06
MKRN2	-0.18604	2.89E-06
INTS10	-0.17717	2.91E-06
NDUFAF4	-0.26865	2.93E-06
FOXN3	0.354583	2.95E-06
SEC22B	-0.19518	2.96E-06
GJC2	0.574086	2.97E-06
GSTA4	0.450455	3.02E-06

SORBS1	0.573744	3.02E-06
VASH2	0.689392	3.05E-06
GLIS3	1.035122	3.06E-06
ZNF763	0.663078	3.06E-06
MPI	0.22656	3.10E-06
EPHB2	0.188406	3.11E-06
ANKRD52	0.161468	3.12E-06
RAP2B	-0.28225	3.13E-06
KDM4A	-0.17513	3.15E-06
FAM216A	0.246787	3.18E-06
NEK9	0.174346	3.18E-06
RPUSD1	-0.24934	3.20E-06
TCF3	0.14982	3.21E-06
ATG4B	-0.22853	3.23E-06
PPIL1	-0.17445	3.23E-06
ZADH2	-0.35305	3.25E-06
LOC284581	0.731601	3.29E-06
LEO1	-0.17271	3.31E-06
RAB11A	-0.16435	3.31E-06
RARS2	-0.20853	3.37E-06
ZNF276	-0.33114	3.40E-06
TRMT61A	-0.188	3.40E-06
SERBP1	-0.13168	3.41E-06
CD9	0.163499	3.41E-06
RNF11	-0.2377	3.43E-06
AP2M1	-0.14173	3.43E-06
NGFRAP1	0.192119	3.43E-06
DCAF15	-0.18391	3.43E-06
HPS3	0.169841	3.44E-06
GGNBP2	-0.18834	3.48E-06
KARS	-0.13589	3.49E-06
SPON1	0.972254	3.50E-06
TMEM63C	0.676232	3.53E-06
SPINT1	0.161472	3.61E-06
VPS25	-0.17596	3.62E-06
SNX24	0.357355	3.63E-06
ST3GAL4-AS1	1.018701	3.66E-06
ZNF672	0.205162	3.70E-06
MRPS16	-0.15567	3.70E-06
PSMF1	-0.15186	3.70E-06
DDHD2	0.232702	3.73E-06
GORAB	-0.21688	3.74E-06
PTK2B	0.350558	3.77E-06
C2orf70	0.636179	3.80E-06
ABCD3	-0.18138	3.80E-06
LAMP1	-0.14379	3.87E-06
PRDM10	-0.32436	3.87E-06

3-Mar	0.514592	3.88E-06
TMED6	0.987942	3.92E-06
ATL2	-0.18768	3.92E-06
CBFA2T2	0.213273	3.92E-06
RFESD	0.872598	3.95E-06
SEPT7P2	-0.2333	3.95E-06
TMEM55A	0.310454	4.02E-06
LOC150776	-0.20339	4.03E-06
BCL7B	-0.17289	4.06E-06
PDGFC	1.008302	4.07E-06
ZKSCAN7	0.6592	4.10E-06
LGMN	0.229347	4.12E-06
TUBB	-0.11975	4.12E-06
CEP290	0.205167	4.14E-06
LINC00659	0.711014	4.15E-06
SNX25	-0.20281	4.16E-06
DPH3	-0.2134	4.17E-06
SLC25A46	-0.1996	4.17E-06
CSGALNACT2	0.162393	4.18E-06
ASIC1	0.293779	4.19E-06
HS1BP3-IT1	1.21889	4.20E-06
POLR1B	-0.20013	4.22E-06
PPP3R1	-0.19728	4.22E-06
WWC2	-0.1784	4.28E-06
HILPDA	0.584889	4.28E-06
SLC27A4	0.24531	4.28E-06
NOXA1	0.467976	4.32E-06
EPN1	-0.19392	4.36E-06
ZNF561	-0.2456	4.40E-06
HSPA13	-0.25442	4.43E-06
SKP1	-0.16769	4.50E-06
RETSAT	0.237019	4.55E-06
BOLA3-AS1	0.627852	4.55E-06
PSIP1	0.174744	4.57E-06
C9orf78	-0.18506	4.59E-06
C6orf106	-0.18072	4.60E-06
NEO1	0.187761	4.63E-06
GNPDA2	0.313952	4.64E-06
WDR53	-0.33583	4.65E-06
KBTBD6	0.457696	4.67E-06
HARS2	-0.19153	4.76E-06
SOAT2	0.534841	4.77E-06
STX1A	0.270199	4.78E-06
INPP5D	0.190799	4.79E-06
FGGY	0.267494	4.89E-06
PDE8A	0.175907	4.89E-06
NUP85	-0.19368	4.96E-06

PKHD1	0.46237	4.98E-06
HOXA10-AS	-0.36324	4.98E-06
TMEM132A	0.238335	4.99E-06
TBC1D24	0.405474	5.02E-06
NCOR2	0.133598	5.03E-06
LGALSL	0.267706	5.04E-06
ZC3H10	0.390449	5.07E-06
TATDN2	-0.20533	5.09E-06
KIAA2018	-0.35085	5.11E-06
MEAF6	-0.18543	5.12E-06
SCAI	0.342113	5.13E-06
SLC12A9	0.403597	5.26E-06
KCTD17	0.363981	5.26E-06
ZNF84	0.284782	5.32E-06
INADL	0.253061	5.45E-06
CTR9	-0.15662	5.48E-06
MOK	-0.37527	5.49E-06
ZBTB48	-0.24335	5.51E-06
KIF1A	0.581832	5.52E-06
MRPL2	-0.2204	5.56E-06
LEMD2	-0.20508	5.56E-06
YLPM1	-0.16481	5.58E-06
MSRB3	0.200136	5.61E-06
LOC1019278	0.630019	5.61E-06
UPF2	-0.17719	5.63E-06
ZNF571	0.52786	5.64E-06
ZRSR2	-0.27795	5.65E-06
SH3BP4	-0.14869	5.67E-06
KHK	0.354438	5.68E-06
DHX33	-0.18865	5.70E-06
RNF170	0.295688	5.71E-06
IRF9	-0.53192	5.74E-06
ZDHHC23	0.546393	5.75E-06
C1D	-0.31811	5.78E-06
GOLIM4	0.233602	5.79E-06
ZNF564	-0.27216	5.80E-06
NDRG1	0.546007	5.81E-06
ROR1	0.496733	5.81E-06
ZNF845	-0.24781	5.82E-06
RXRB	-0.25958	5.82E-06
VIMP	-0.24076	5.83E-06
ANKRD55	0.761049	5.84E-06
TES	-0.15874	5.96E-06
IBTK	-0.19042	5.96E-06
VWA5B2	0.333115	5.97E-06
TUBD1	-0.37887	6.02E-06
FGF1	1.129479	6.05E-06

KLHL22	0.408159	6.07E-06
VANGL1	0.27904	6.07E-06
ERC1	0.185407	6.08E-06
ORC6	-0.16401	6.08E-06
SFN	-0.19811	6.09E-06
ZNF593	-0.22256	6.14E-06
CHN1	0.208409	6.16E-06
AZI2	-0.20162	6.20E-06
CSTF2T	-0.18209	6.20E-06
IPO4	-0.17532	6.25E-06
RASSF2	1.103066	6.25E-06
PSMA6	-0.15557	6.26E-06
MEMO1	-0.23147	6.27E-06
SDCBP	0.163741	6.27E-06
ELMO3	-0.68697	6.30E-06
TAF1C	-0.33383	6.30E-06
ZBTB7C	0.363383	6.32E-06
LINC00668	1.18764	6.34E-06
DUSP1	-0.21971	6.36E-06
TRIM28	-0.17092	6.37E-06
LRRC37A6P	1.186916	6.38E-06
MAPKAPK5-/	-0.32065	6.43E-06
CXCL3	-0.4838	6.45E-06
ABLIM3	0.362574	6.48E-06
ATG3	-0.186	6.55E-06
ZBTB44	-0.22263	6.55E-06
CTBP1	-0.19145	6.56E-06
ADAP1	0.261496	6.56E-06
SLC43A1	0.231345	6.56E-06
CALM1	0.160331	6.62E-06
CBFB	-0.13526	6.67E-06
ZNF397	0.549818	6.67E-06
DNAJC11	-0.14677	6.71E-06
BCCIP	-0.17858	6.74E-06
SSBP2	0.248972	6.75E-06
FGFR1OP	-0.31528	6.76E-06
DNAH12	0.653933	6.80E-06
PLIN2	0.154645	6.82E-06
UBE2I	-0.13856	6.93E-06
ATP1B2	1.011795	6.94E-06
LINC01152	1.323582	6.97E-06
PSMB2	-0.14109	7.01E-06
ZNF433	0.613502	7.04E-06
ANKRD31	1.001516	7.11E-06
PJA2	-0.17094	7.12E-06
PROX1	0.262069	7.17E-06
MRPS11	-0.2062	7.17E-06

LPAR2	0.483867	7.19E-06
RPN1	-0.13918	7.24E-06
TRPM2	0.460593	7.24E-06
PTPN2	-0.17018	7.27E-06
BACE2	0.167293	7.28E-06
LINC01061	0.49131	7.29E-06
DECR2	0.335456	7.29E-06
SPRY4	-0.1876	7.30E-06
CCDC134	0.518255	7.33E-06
DLL3	1.047962	7.33E-06
LUZP1	0.179282	7.34E-06
MNX1-AS1	0.583668	7.34E-06
DHX15	-0.12997	7.40E-06
PRODH2	0.57731	7.40E-06
TEC	0.471538	7.47E-06
LOC1019295	0.373904	7.53E-06
SUV39H2	-0.2442	7.55E-06
TBC1D2	0.282882	7.55E-06
USP18	0.328303	7.55E-06
LOC399815	0.74079	7.63E-06
SPRYD4	-0.47484	7.67E-06
CAPN12	0.78393	7.71E-06
DHX9	-0.13627	7.71E-06
ZNF827	0.368981	7.76E-06
ARPC1A	-0.18016	7.77E-06
CAPN15	-0.3488	7.77E-06
KIAA1804	0.269327	7.78E-06
GUSBP5	1.031789	7.85E-06
EFNB3	0.798954	7.91E-06
TCF25	-0.17142	8.01E-06
NUDC	-0.15121	8.14E-06
RAP1B	-0.15105	8.14E-06
GBF1	-0.14778	8.20E-06
EIF3G	-0.14805	8.21E-06
CHD7	0.220518	8.22E-06
AFF4	-0.17429	8.29E-06
CCDC85B	0.202691	8.29E-06
ZCCHC2	0.388658	8.32E-06
ABCB6	0.32439	8.33E-06
DGCR8	-0.23561	8.33E-06
ZFYVE1	-0.31092	8.33E-06
FMR1	-0.19591	8.39E-06
API5	-0.1442	8.41E-06
MCF2L	0.296575	8.42E-06
CEACAM19	0.585366	8.46E-06
C1orf43	-0.1372	8.56E-06
ZMPSTE24	-0.18371	8.57E-06

FAM134A	-0.23194	8.66E-06
CCT6A	-0.12367	8.68E-06
SPOCD1	1.014249	8.70E-06
ARL14EP	-0.25306	8.72E-06
RYK	-0.17921	8.76E-06
MGAT5B	0.17171	8.76E-06
ZNF337	0.330997	8.77E-06
MARK4	0.329686	8.80E-06
LOC1019283	0.971548	8.84E-06
NDRG3	0.249806	8.85E-06
LAMTOR1	-0.18025	8.87E-06
VDAC1	-0.15942	8.91E-06
SEC63	-0.17225	8.94E-06
BIRC2	-0.16193	8.95E-06
MRPL36	-0.23653	8.95E-06
ATP5F1	-0.14197	8.96E-06
CREB3	-0.21722	9.00E-06
MCTP2	0.45324	9.03E-06
MRPL45	-0.19682	9.04E-06
ZNF687	-0.18283	9.06E-06
CUL9	0.268317	9.10E-06
SLC35C2	-0.1824	9.11E-06
ACOX1	-0.18594	9.13E-06
FKBP9P1	0.470473	9.17E-06
OGDH	0.151057	9.17E-06
ARIH2	-0.17003	9.18E-06
POGZ	-0.14037	9.22E-06
EFTUD2	-0.13686	9.27E-06
SLC47A1	0.933879	9.31E-06
PSMB4	-0.15093	9.32E-06
FAM109B	0.839142	9.37E-06
KIF2A	-0.19877	9.45E-06
SCAMP1-AS1	0.297421	9.47E-06
FKBP15	-0.19847	9.49E-06
DDB2	0.201479	9.50E-06
UBE2D2	-0.14744	9.52E-06
TULP3	0.187901	9.58E-06
SGK2	0.649958	9.64E-06
EGR3	0.166435	9.74E-06
PIM2	-0.21592	9.83E-06
PYCR2	-0.18152	9.89E-06
ASF1A	-0.1675	1.00E-05
LINC00506	0.841934	1.00E-05
HEATR1	-0.14813	1.01E-05
MED16	-0.21088	1.01E-05
LINC00493	-0.29557	1.01E-05
VAMP1	0.448937	1.02E-05

NAA20	-0.21969	1.02E-05
TGFBI	-0.17557	1.02E-05
RWDD1	-0.18458	1.03E-05
RHEB	-0.15035	1.03E-05
ST7	-0.18867	1.03E-05
NUP107	-0.15073	1.04E-05
C1orf56	0.552242	1.04E-05
ZNF653	-0.36416	1.04E-05
CORO1C	-0.13545	1.05E-05
GXYLT2	0.916317	1.06E-05
MAPRE1	-0.15394	1.07E-05
NOTCH3	0.809433	1.07E-05
MFSD1	-0.27099	1.07E-05
ALDH1B1	0.217551	1.07E-05
LOC1002870	0.646487	1.08E-05
TNFRSF19	0.267471	1.08E-05
ZNF630	0.613793	1.09E-05
NUCB2	0.197124	1.10E-05
TOP3A	-0.17836	1.10E-05
TOE1	-0.18006	1.10E-05
POLD3	-0.18099	1.10E-05
HIST2H2BF	0.583944	1.10E-05
MAGED2	0.149845	1.11E-05
ATG12	-0.17709	1.11E-05
ZNF490	-0.36714	1.11E-05
SLC29A4	0.910264	1.15E-05
HIC2	-0.36902	1.15E-05
ZBTB3	-0.75322	1.16E-05
ZNF628	-0.32027	1.16E-05
GALT	0.294819	1.16E-05
NBPF3	0.287446	1.16E-05
PHYKPL	0.395303	1.16E-05
SOX30	0.932552	1.17E-05
ZFP62	0.278121	1.17E-05
IDH1	0.230774	1.17E-05
RAD54B	0.271378	1.17E-05
ENO3	0.275603	1.17E-05
THG1L	-0.23191	1.20E-05
HSF1	-0.21249	1.20E-05
SLC35F6	-0.22363	1.20E-05
LUCAT1	0.644582	1.21E-05
WDR11	-0.18862	1.21E-05
SMPDL3B	0.611928	1.21E-05
FAM92A1	0.289378	1.21E-05
INPP5K	-0.23369	1.22E-05
TTI1	-0.17774	1.22E-05
GNS	-0.23621	1.23E-05

MLXIPL	0.415768	1.23E-05
TXNDC9	-0.20209	1.23E-05
B3GALNT1	0.47285	1.23E-05
CCDC74A	0.455966	1.24E-05
PHACTR4	-0.20515	1.24E-05
OBFC1	0.271259	1.24E-05
COMMD5	-0.29833	1.24E-05
10-Sep	0.183578	1.25E-05
COL11A2	0.808227	1.25E-05
EXOSC3	-0.21194	1.26E-05
FAM21A	-0.16829	1.28E-05
PSPC1	-0.1575	1.30E-05
CDKN2C	-0.28437	1.31E-05
ZNF223	-0.34903	1.31E-05
ZNF395	0.20468	1.31E-05
PSMB6	-0.16061	1.31E-05
FAM86DP	0.337567	1.32E-05
GNL3L	-0.18034	1.32E-05
UNC13A	1.072311	1.32E-05
RAD17	-0.24046	1.32E-05
FIP1L1	-0.1444	1.33E-05
RCSD1	0.37447	1.35E-05
SMARCE1	-0.14028	1.35E-05
ZBTB26	-0.49223	1.36E-05
TMEM186	-0.54379	1.36E-05
PWWP2A	-0.19226	1.37E-05
EMP3	0.273725	1.37E-05
EHD4	-0.13149	1.37E-05
MYO10	0.213797	1.38E-05
CUL4A	-0.14683	1.38E-05
NBPF10	1.068965	1.38E-05
C5orf34	-0.20132	1.38E-05
FLJ37453	0.497569	1.39E-05
POLR2C	-0.14514	1.39E-05
ARHGEF39	0.266505	1.40E-05
C1GALT1	-0.1865	1.41E-05
GOLGA4	-0.19941	1.41E-05
FADS2	0.325556	1.42E-05
PPIC	0.564532	1.43E-05
GRK6	-0.19956	1.43E-05
PHF13	0.556728	1.43E-05
ZNF514	0.693494	1.43E-05
FBXO18	-0.19839	1.44E-05
C21orf67	0.851271	1.44E-05
ASAH1	0.222226	1.45E-05
SHISA4	0.420378	1.46E-05
HAS3	0.181469	1.47E-05

ALS2	-0.21905	1.47E-05
PRR14	0.236648	1.47E-05
SEC31A	-0.15486	1.47E-05
FLT3LG	0.830232	1.47E-05
ARHGEF25	0.376249	1.48E-05
OTULIN	-0.2128	1.48E-05
PNKD	0.252884	1.50E-05
MRPL18	-0.15948	1.50E-05
DCUN1D5	-0.15704	1.51E-05
ZNF302	0.226235	1.51E-05
BRIP1	-0.18047	1.52E-05
CATSPER1	0.53359	1.52E-05
STAT5B	0.280035	1.53E-05
RHOA	-0.13871	1.53E-05
CTDP1	-0.22894	1.54E-05
NSUN6	-0.23592	1.54E-05
LOC1019277	0.788004	1.55E-05
PRKCG	1.193732	1.56E-05
NSRP1	-0.16905	1.56E-05
ZNF100	-0.22305	1.56E-05
RMND5A	-0.20859	1.57E-05
ZNF233	0.621684	1.58E-05
IGBP1	-0.21875	1.59E-05
PPP3CC	-0.20913	1.59E-05
PROSER1	-0.19165	1.59E-05
APEX2	-0.16048	1.59E-05
XPO4	-0.21269	1.59E-05
HSD3B7	0.338647	1.59E-05
ZNF841	-0.32469	1.60E-05
TMEM9	0.224498	1.60E-05
TNFRSF10A	0.313897	1.60E-05
RAD51AP2	1.018589	1.61E-05
RAPGEF3	0.562018	1.62E-05
POFUT1	0.147695	1.63E-05
FSCN2	1.115563	1.64E-05
FNTA	-0.16824	1.66E-05
ARHGAP44	0.693988	1.67E-05
LINC01186	0.731199	1.69E-05
ARPC2	-0.13622	1.69E-05
RUNDC3A	0.773737	1.70E-05
SYNE2	0.366125	1.73E-05
IFNAR1	-0.19992	1.73E-05
MYO18A	0.164269	1.74E-05
ST7-AS1	0.709804	1.74E-05
SF3B2	-0.1327	1.76E-05
LOC284454	-1.0056	1.78E-05
VEPH1	0.783973	1.78E-05

RBM23	-0.16903	1.79E-05
DBT	-0.19291	1.81E-05
EXOC1	-0.19488	1.82E-05
UTP23	-0.179	1.82E-05
GCLM	0.255349	1.83E-05
ACSF2	0.547066	1.83E-05
RECQL5	-0.21061	1.83E-05
CLTCL1	0.731385	1.84E-05
NABP2	0.159293	1.84E-05
SYT8	0.738907	1.84E-05
ALKBH2	0.374361	1.84E-05
SELK	-0.28771	1.85E-05
PRELID2	0.415217	1.87E-05
BEND7	0.850575	1.88E-05
FLJ44511	0.845351	1.88E-05
HIST1H1T	1.050353	1.88E-05
ADAT3	0.418806	1.88E-05
C19orf52	-0.29757	1.90E-05
DAAM1	-0.20546	1.90E-05
HIST1H2BK	0.186291	1.92E-05
MAST4	-0.23409	1.92E-05
PSMB1	-0.1568	1.92E-05
VMP1	-0.16347	1.92E-05
MLLT4	0.170376	1.93E-05
ATP6V0E1	-0.19404	1.94E-05
RPL13AP20	0.894157	1.95E-05
SLC9A5	0.601745	1.96E-05
NOP14	-0.13248	1.96E-05
NEMF	-0.16653	1.97E-05
PLAC1	1.032526	1.98E-05
SRSF8	-0.15174	1.99E-05
PRICKLE2	0.686989	1.99E-05
TFCP2	-0.15377	1.99E-05
SNRPB	-0.13595	1.99E-05
GNRHR2	0.797678	1.99E-05
LOC1003792	0.984873	1.99E-05
LOC400863	1.011781	2.01E-05
ZNF646	-0.1904	2.01E-05
CCDC7	0.699235	2.02E-05
HCFC1R1	0.427361	2.02E-05
TBC1D25	-0.26505	2.11E-05
HIST1H3E	-0.8789	2.11E-05
FUT2	1.091761	2.14E-05
NSMAF	-0.16418	2.15E-05
GNA11	-0.17878	2.15E-05
AATK	1.015248	2.15E-05
PTP4A2	-0.14301	2.16E-05

LENG9	0.751996	2.17E-05
TP53I13	0.402865	2.17E-05
PLK1	-0.184	2.20E-05
CCDC121	0.465108	2.21E-05
DCAF13	-0.16275	2.21E-05
BAMBI	-0.19286	2.23E-05
LINC01234	0.393308	2.23E-05
LOC1005059	0.677369	2.28E-05
CENPO	-0.16059	2.28E-05
CES2	-0.1492	2.30E-05
ERV3-1	0.444833	2.32E-05
DDX46	-0.1427	2.33E-05
CCDC40	0.315261	2.34E-05
ZNF236	0.310528	2.34E-05
NAGPA	-0.29689	2.35E-05
CHPF	-0.18476	2.36E-05
LOC1027238	0.541311	2.37E-05
TTC18	0.925958	2.38E-05
CFL1	-0.12388	2.38E-05
CSMD3	0.429692	2.39E-05
SYDE2	0.593689	2.40E-05
CDKN2AIP	-0.16427	2.41E-05
KLHL42	0.170804	2.41E-05
DDN	0.693198	2.42E-05
CRTC2	-0.19285	2.42E-05
GTF2H5	0.229595	2.42E-05
WASH1	-0.31511	2.43E-05
DPH2	-0.1705	2.44E-05
IFT81	0.18712	2.45E-05
SLC29A2	0.404657	2.46E-05
DEPDC7	0.182599	2.47E-05
RBPJ	-0.18834	2.47E-05
CERS6	0.172466	2.47E-05
DDX51	-0.17381	2.47E-05
DYNLT3	0.182504	2.48E-05
TAX1BP1	-0.14482	2.48E-05
CST7	0.202048	2.49E-05
ARMC2	0.675387	2.50E-05
RNFT2	0.653624	2.52E-05
EVA1C	0.40242	2.54E-05
PRPS2	0.189145	2.55E-05
ZNF470	0.452925	2.56E-05
QRSL1	-0.20193	2.56E-05
PCED1A	0.28527	2.58E-05
PNPLA2	-0.18495	2.58E-05
KIAA1211	0.263692	2.58E-05
MPG	0.186739	2.60E-05

LOC440300	0.999833	2.61E-05
PTBP3	-0.15385	2.61E-05
ANKMY2	0.204153	2.64E-05
VPS33B	-0.24843	2.66E-05
FMNL1	0.187794	2.66E-05
PLA2G4D	0.943878	2.67E-05
SBF2	0.155505	2.67E-05
SAP18	-0.15663	2.69E-05
SEC24D	0.291272	2.70E-05
ANPEP	1.024518	2.70E-05
LRRC58	-0.17652	2.70E-05
CADM4	0.27812	2.71E-05
CES1	1.026924	2.71E-05
RFX1	-0.25744	2.71E-05
C1orf105	0.82057	2.71E-05
ZNF326	-0.15574	2.72E-05
MOGS	-0.26471	2.73E-05
MYLIP	0.383007	2.73E-05
8-Mar	-0.24441	2.74E-05
QPCT	0.370205	2.74E-05
CARS	-0.14108	2.74E-05
GIGYF1	-0.37373	2.76E-05
CAMK4	0.528519	2.76E-05
MMP25	-1.02288	2.77E-05
TAF9	-0.15584	2.80E-05
CCDC88A	0.173922	2.82E-05
MZF1	0.388143	2.84E-05
AOC3	-0.72285	2.84E-05
CSRP2BP	0.406348	2.85E-05
BBX	0.239005	2.85E-05
CREBL2	-0.20735	2.87E-05
CUX1	0.164367	2.88E-05
LASP1	-0.12298	2.88E-05
BUB3	-0.13819	2.88E-05
IQCE	0.192664	2.89E-05
ATAD3B	-0.20036	2.90E-05
GCH1	0.237988	2.91E-05
CD44	0.348239	2.93E-05
SHANK2	0.309617	2.93E-05
ZNF48	0.234665	2.93E-05
CHD3	0.182452	2.95E-05
MAP4K2	0.336905	2.97E-05
LOC202181	0.487451	3.00E-05
AMER1	0.334811	3.00E-05
PELP1	-0.15006	3.04E-05
CAV1	-0.13598	3.05E-05
MED7	-0.33021	3.05E-05

GNA13	-0.14882	3.05E-05
HSF2	-0.16273	3.08E-05
B3GALTL	0.328662	3.09E-05
LOC1019290	1.029074	3.11E-05
PLA2G15	-0.17626	3.11E-05
FOXRED1	-0.19157	3.12E-05
UXS1	0.218768	3.19E-05
PRKCE	0.52639	3.20E-05
SELPLG	0.840163	3.20E-05
FAXC	0.313983	3.21E-05
RUFY1	-0.17677	3.23E-05
ADPGK	-0.16495	3.23E-05
SIPA1L3	0.163217	3.26E-05
ARL3	0.254939	3.30E-05
SKI	-0.18725	3.30E-05
RBM28	-0.18346	3.32E-05
SNX11	-0.21897	3.38E-05
TUBG2	0.397177	3.40E-05
SULT1A1	0.328252	3.41E-05
PPM1M	0.445447	3.41E-05
PIPSL	-0.93653	3.45E-05
TRAIP	-0.22528	3.45E-05
ABCB4	0.860601	3.46E-05
CD22	0.873697	3.46E-05
GTF3C5	-0.15653	3.48E-05
AAAS	-0.17676	3.50E-05
FBLN1	0.216967	3.52E-05
ZSCAN2	0.350826	3.53E-05
SLC52A2	-0.21925	3.54E-05
ERI2	-0.20795	3.57E-05
BATF3	1.155087	3.57E-05
C18orf8	-0.16886	3.57E-05
NLRP1	1.077605	3.58E-05
METTL2A	-0.24502	3.58E-05
C6orf223	0.933292	3.59E-05
EPHX1	0.224652	3.63E-05
C9orf89	0.255013	3.65E-05
CCDC84	-0.19492	3.67E-05
FCHSD1	0.264313	3.73E-05
ZNF391	0.730053	3.73E-05
CCDC64	0.301989	3.76E-05
AVL9	-0.20414	3.77E-05
PLEKHA4	0.259093	3.78E-05
RACGAP1	-0.13181	3.78E-05
SPNS2	-0.1281	3.82E-05
GGT5	0.994396	3.82E-05
CCDC42B	0.953503	3.82E-05

AOC1	0.531337	3.83E-05
PPWD1	-0.17126	3.84E-05
LARS2	-0.14828	3.85E-05
TTC3	0.134804	3.86E-05
ABCA2	0.295141	3.86E-05
TP53INP2	0.255691	3.89E-05
ETV5	-0.14802	3.92E-05
NQO1	0.131668	3.94E-05
ZNF708	-0.40257	3.95E-05
MXD4	-0.26482	3.96E-05
FMO4	0.812697	3.96E-05
ALYREF	-0.13366	3.98E-05
COG1	-0.15734	4.02E-05
PCLO	0.285105	4.02E-05
APTR	0.352066	4.04E-05
LOC1005069	0.437858	4.06E-05
SET	-0.11399	4.10E-05
NRF1	-0.18194	4.10E-05
UBE2D1	-0.1679	4.13E-05
ZFHX4	0.675557	4.14E-05
KIAA1549	0.252908	4.14E-05
NKD1	-0.12427	4.14E-05
ACP2	-0.17327	4.19E-05
DNAH1	0.408735	4.20E-05
PARP6	-0.1993	4.21E-05
MFAP3	-0.22232	4.22E-05
TRAF1	0.848385	4.25E-05
SLC25A42	0.302616	4.27E-05
ZFPM1	-0.40252	4.30E-05
PEX14	-0.21348	4.32E-05
FITM2	0.617586	4.34E-05
ANAPC2	-0.25104	4.37E-05
MIER3	-0.18609	4.39E-05
ZNF513	-0.34333	4.40E-05
ERCC3	-0.14231	4.40E-05
SUB1	-0.15974	4.43E-05
DSG2	0.163783	4.43E-05
DUS1L	-0.14376	4.45E-05
BZRAP1-AS1	0.556057	4.48E-05
EDEM3	-0.24431	4.49E-05
ZNF354B	0.346618	4.49E-05
SFXN1	0.155853	4.51E-05
PYROXD2	0.431652	4.51E-05
NARF	-0.1854	4.51E-05
JMY	-0.33548	4.52E-05
TYW1	-0.23492	4.53E-05
CMTM7	0.214196	4.54E-05

RBP1	0.127595	4.57E-05
CYB5D2	0.226589	4.58E-05
SPRYD7	0.304861	4.60E-05
AHSA1	-0.13878	4.60E-05
LOXL1-AS1	0.58119	4.62E-05
DLGAP5	-0.14474	4.62E-05
KCNC4	0.795958	4.67E-05
HINFP	-0.23538	4.72E-05
RBM26	-0.15386	4.76E-05
NUDT7	0.431966	4.77E-05
GID4	-0.52217	4.81E-05
CDC42EP4	0.357736	4.82E-05
VTI1B	-0.17254	4.82E-05
TOMM22	-0.13866	4.83E-05
TCEAL1	0.197948	4.85E-05
KIF3B	-0.1599	4.86E-05
PDZD7	0.418182	4.90E-05
PTPN23	-0.16628	4.91E-05
KIF14	-0.21668	4.92E-05
PTGES3	-0.15144	4.93E-05
MTHFR	0.201244	4.95E-05
PLOD1	0.158509	4.95E-05
ZW10	-0.18462	4.97E-05
AFTPH	-0.18415	4.98E-05
ACTR2	-0.15093	4.98E-05
LOC388849	1.002815	5.00E-05
ANKRD20A8I	0.663351	5.02E-05
SPATA6L	0.811385	5.03E-05
ZNF784	-0.53514	5.03E-05
B3GNT5	-0.26007	5.05E-05
MUT	-0.1821	5.05E-05
TNNC1	0.716046	5.06E-05
FAM98A	-0.18703	5.06E-05
PPP6R3	-0.12023	5.06E-05
QPRT	0.237995	5.08E-05
ANKRD9	0.700869	5.10E-05
CTU1	-0.40429	5.11E-05
NVL	-0.15657	5.11E-05
CCNB2	-0.14788	5.11E-05
LINC01232	0.845153	5.13E-05
TRPM7	-0.16775	5.13E-05
GOT2	-0.12523	5.16E-05
SULT1A2	1.003749	5.16E-05
KLHL30	0.549769	5.19E-05
PDCD10	-0.17883	5.22E-05
DENR	-0.13475	5.26E-05
PIR	0.420137	5.26E-05

FARSB	-0.13865	5.27E-05
SDHA	-0.12568	5.28E-05
NELFCD	-0.12002	5.29E-05
VPS13B	0.196582	5.32E-05
COL6A1	0.205998	5.32E-05
SOCS6	-0.33077	5.34E-05
PTPN4	0.282156	5.40E-05
C14orf166	-0.14065	5.42E-05
PMEPA1	-0.16212	5.44E-05
PCM1	-0.15284	5.44E-05
TMEM63A	0.324295	5.46E-05
PHF5A	-0.18049	5.46E-05
HKR1	-0.19741	5.48E-05
CASP7	0.201023	5.55E-05
EIF3M	-0.12356	5.56E-05
AGMAT	0.248797	5.60E-05
HEBP1	0.23631	5.61E-05
TMEM62	0.228436	5.72E-05
TTC39C	0.290894	5.77E-05
ANKDD1A	0.527624	5.78E-05
SYTL2	0.766457	5.81E-05
MIEF2	-0.33084	5.82E-05
USP28	0.167725	5.83E-05
MRPL4	-0.17738	5.85E-05
RNF126	-0.18891	5.86E-05
MCM8	-0.16893	5.88E-05
LRCH4	-0.14963	5.90E-05
UCHL3	-0.1687	5.90E-05
MLEC	-0.13935	5.92E-05
SMARCAD1	-0.15788	5.94E-05
TUBGCP2	-0.14744	5.97E-05
PEF1	-0.17359	5.97E-05
SH3BGRL	0.161384	5.98E-05
BTN2A3P	0.413859	6.03E-05
MAP3K5	0.318499	6.03E-05
CNTRL	0.168242	6.04E-05
GYS1	0.24205	6.04E-05
MFSD3	0.399026	6.09E-05
ZNF681	0.300522	6.10E-05
MYOM3	0.807291	6.11E-05
STAM	-0.28998	6.11E-05
CHD4	0.113182	6.14E-05
AGL	0.168394	6.14E-05
MARVELD1	0.39828	6.16E-05
HOXA9	-0.3858	6.20E-05
GK	0.217188	6.29E-05
LRRK1	0.22238	6.29E-05

WDR3	-0.15845	6.29E-05
PSMB8	0.427643	6.30E-05
ACTR6	-0.25316	6.30E-05
TRIP13	-0.15201	6.32E-05
SLC4A11	0.214066	6.32E-05
FURIN	-0.1907	6.32E-05
INPP1	0.214061	6.32E-05
ING5	-0.22143	6.34E-05
ADCY7	0.394308	6.35E-05
MAP2K7	-0.17919	6.36E-05
ALDH3A1	0.311758	6.36E-05
IL7R	0.464407	6.41E-05
CYP4V2	0.3124	6.54E-05
LINC00638	0.815855	6.58E-05
ACTL10	0.596854	6.60E-05
RNPEPL1	-0.20563	6.61E-05
RAB3IP	0.187768	6.62E-05
LOC1005072	0.915117	6.64E-05
KSR2	0.669168	6.65E-05
LOC1019281	0.484653	6.65E-05
HMGXB3	-0.14913	6.70E-05
MUC1	0.24505	6.70E-05
NACC2	-0.19606	6.70E-05
TMEM88	-0.80758	6.70E-05
DTX3L	0.220798	6.74E-05
XRCC6	-0.12175	6.74E-05
RASGEF1B	1.029168	6.75E-05
OGFR	-0.20812	6.79E-05
JARID2	0.243268	6.80E-05
STXBP3	-0.17154	6.88E-05
CBX7	0.628089	6.91E-05
TM7SF2	0.254971	6.92E-05
AKAP7	0.484591	6.92E-05
CDCA7L	0.140014	6.94E-05
NOTUM	-0.17365	6.94E-05
DUSP14	0.19599	6.94E-05
GRSF1	-0.15488	6.96E-05
PGPEP1	0.314612	7.05E-05
INTS1	-0.14659	7.05E-05
F8A1	0.609558	7.07E-05
ORAI3	0.30522	7.08E-05
ADO	-0.16719	7.08E-05
DOT1L	-0.22933	7.10E-05
U2SURP	-0.11226	7.12E-05
KCTD11	-0.19619	7.13E-05
HNRNPA2B1	-0.12035	7.13E-05
VPS13A	0.192463	7.17E-05

LINC00346	0.76644	7.19E-05
TUBGCP3	-0.13573	7.22E-05
IGFBP6	0.204781	7.25E-05
FN3K	0.483191	7.27E-05
TRPC4AP	-0.13042	7.27E-05
LOC1027244	0.927338	7.28E-05
MCU	-0.16079	7.32E-05
EXOSC4	-0.31549	7.34E-05
PPIP5K2	-0.19823	7.37E-05
ZNF595	-0.36942	7.41E-05
CPA5	0.549494	7.43E-05
COQ2	-0.23543	7.46E-05
RNF219	-0.15307	7.47E-05
BANP	-0.25949	7.47E-05
LEPR	0.31711	7.50E-05
LOC1019273	0.726635	7.53E-05
RASAL2	0.213567	7.57E-05
FXR1	-0.14344	7.57E-05
LTBP4	0.277786	7.61E-05
ZDBF2	0.272309	7.61E-05
LRP5L	-0.27718	7.62E-05
FOXM1	-0.13016	7.62E-05
ATP10A	0.829188	7.65E-05
FAM135A	0.288341	7.68E-05
ARL6	0.349361	7.70E-05
DHX36	-0.13355	7.70E-05
RPP30	-0.18532	7.71E-05
SNX1	-0.14513	7.71E-05
MFI2-AS1	0.65558	7.71E-05
RNF169	-0.19178	7.73E-05
CEP19	0.356859	7.73E-05
HIPK2	0.24286	7.84E-05
MLYCD	-0.27374	7.86E-05
HOXB7	0.219476	7.86E-05
VASN	0.300734	7.95E-05
EXOG	-0.20712	7.96E-05
THAP4	-0.22375	7.97E-05
IPO11	-0.19548	8.02E-05
KIF27	0.512186	8.12E-05
ABHD3	0.295716	8.16E-05
FBXO34	-0.24065	8.18E-05
CRIP1	0.473499	8.23E-05
ZBTB39	-0.18028	8.24E-05
APOPT1	-0.20363	8.24E-05
PRRC2B	0.137531	8.28E-05
IL17RB	0.177883	8.28E-05
C14orf23	-0.65253	8.28E-05

SNX19	-0.15099	8.33E-05
PI4K2A	-0.17258	8.36E-05
USH1C	0.211551	8.41E-05
TRIM32	0.235792	8.43E-05
SH2B2	0.721963	8.43E-05
FAM214B	-0.46913	8.44E-05
RAPH1	0.158612	8.44E-05
LOC1001307	0.292548	8.45E-05
LSM8	-0.17117	8.45E-05
PDPR	-0.16744	8.50E-05
ERBB2IP	-0.1357	8.51E-05
LYAR	-0.16684	8.55E-05
OGFOD1	-0.13926	8.56E-05
NDUFS8	-0.17083	8.62E-05
OXSR1	-0.14299	8.66E-05
LONP1	-0.13732	8.70E-05
RAD50	0.149198	8.73E-05
ADAMTSL4	0.70683	8.74E-05
STK16	-0.26149	8.74E-05
RALA	-0.14964	8.76E-05
RAB8B	0.2638	8.77E-05
BHLHA15	0.894185	8.79E-05
ELFN1	0.730006	8.80E-05
CRIPT	-0.35478	8.86E-05
NOL10	-0.19951	8.87E-05
PGAP1	0.268001	8.89E-05
SLC25A32	-0.15093	8.91E-05
BTN2A1	-0.17026	8.92E-05
FBF1	0.255488	9.06E-05
ZNF570	-0.42282	9.08E-05
TET2	-0.24705	9.09E-05
CES3	0.717424	9.09E-05
ZNF503	-0.17047	9.11E-05
NCOA5	-0.14456	9.12E-05
SNCG	0.772513	9.12E-05
MAPK7	-0.29608	9.16E-05
BCL2L13	-0.16974	9.18E-05
FAM3C	0.178328	9.19E-05
ACRBP	0.760645	9.19E-05
ZNF551	-0.28476	9.23E-05
BCL7A	0.381387	9.23E-05
SPDL1	-0.18715	9.25E-05
FIBCD1	0.186021	9.27E-05
CD97	0.127879	9.29E-05
ALG11	-0.46441	9.34E-05
GIPC1	0.155183	9.34E-05
SGOL2	-0.19559	9.36E-05

APOBEC3D	0.815056	9.39E-05
TMEM79	-0.63145	9.40E-05
CALML3-AS1	0.681645	9.41E-05
RILPL2	0.30295	9.47E-05
GALNT16	0.3999	9.52E-05
CLIC1	-0.13696	9.58E-05
SYTL5	0.952702	9.66E-05
EIF2AK4	0.204512	9.70E-05
ZHX3	0.31098	9.72E-05
REL	-0.26324	9.73E-05
LOC645166	0.483678	9.76E-05
NAALADL1	1.006469	9.76E-05
ABCC4	0.19678	9.79E-05
BOD1L1	-0.15566	9.88E-05
ILK	-0.13029	9.89E-05
C7orf63	-0.34523	9.92E-05
LOC1005068	0.522401	9.93E-05
PAX6	-0.2682	9.94E-05
DOPEY1	0.198134	9.94E-05
LTBR	-0.14193	9.96E-05
