# Supplementary material for: Small molecule/ML327 mediated transcriptional de-repression of E-cadherin and inhibition of epithelial-to-mesenchymal transition
Source: Oncotarget. 2015 Jun 10;6(26):22934–48. doi: 10.18632/oncotarget.4473 (PMC4673210; doi:10.18632/oncotarget.4473)
Supplement: Supplementary file 3 [file oncotarget-06-22934-s003.pdf]

H520_CHX ML327 treatment differential expressed genes from RNA seq analysis

gene	logFC	FDR
FILIP1L	2.540865	9.74E-171
LDLR	-1.01154	3.19E-156
NEFL	1.138033	3.48E-156
DMRT2	1.478854	5.42E-146
FAM46A	2.062942	6.65E-145
DNAJB1	-1.06482	8.39E-143
TUBB4B	-0.88529	1.28E-138
RASL11B	-0.97512	3.21E-138
ID2	-0.94329	1.57E-136
SLITRK3	5.882672	1.88E-131
SALL1	3.07429	5.71E-130
MED26	-1.24626	2.22E-124
ARID5B	1.698039	5.55E-117
ID3	-0.73718	6.83E-116
CCNL1	-0.77092	5.25E-115
EPDR1	1.100808	2.05E-111
BRD2	-0.86285	9.89E-111
EOMES	2.809427	1.16E-109
MKNK2	-0.8042	1.59E-107
TOB1	-1.0973	1.97E-107
L3MBTL3	1.759703	1.86E-106
SLC30A2	3.679663	1.78E-105
EPHA2	1.434825	2.05E-100
STMN4	5.492688	9.38E-100
SIX1	2.143772	1.22E-97
CLK1	-0.90779	3.75E-97
HERPUD1	-0.84822	2.45E-96
SKI	-0.84388	5.00E-96
LPPR4	1.039522	7.32E-96
LRIF1	1.108387	1.79E-95
KCTD1	1.744849	5.60E-95
HSPA8	-0.99883	2.09E-92
EGR2	1.688644	8.17E-92
TOB2	-1.23483	1.04E-91
FAM84A	2.1687	1.36E-91
ADAMTS1	4.224635	1.42E-91
PCDH20	4.075904	2.32E-91
SPRY1	4.102849	1.19E-89
RREB1	1.180407	5.90E-89
AARD	1.041752	1.19E-87
TSPYL2	-0.88206	1.09E-86
BCL11B	2.250221	2.12E-86
CSNK1A1	-0.76848	2.18E-86
ZBTB2	-0.77962	3.66E-86
PCF11	-1.19182	9.93E-86

AXIN2	-0.67766	1.48E-83
ZNF574	-1.12482	4.32E-83
RBM39	-0.73615	5.81E-82
CHD7	0.807364	1.96E-80
ZNF408	-1.06725	3.40E-80
SOX3	2.725573	2.16E-77
PCDH18	0.731509	3.30E-77
SOX11	2.362746	5.33E-77
NKX6-1	1.916292	1.24E-76
TWIST1	1.333773	1.61E-76
DKK1	1.643763	2.88E-76
DDIT4L	2.674399	3.67E-76
TBX2	-0.97206	5.69E-76
ZNF365	1.728648	1.40E-75
DDIT3	-1.22542	3.46E-75
CCDC71L	1.682307	7.93E-74
IER2	-0.77706	3.69E-72
SOCS2	2.985707	1.63E-71
GEM	3.034624	2.19E-70
PITX2	2.043727	1.93E-69
NAP1L5	2.090456	2.05E-69
SNAI2	1.700357	1.04E-68
FAM110B	1.79474	2.71E-68
SGK1	0.954654	6.25E-68
PTF1A	1.48959	6.25E-68
NGFR	2.076823	1.09E-67
CCNT2	-0.80101	1.13E-67
ETV1	1.113112	3.39E-67
SP5	0.904114	6.98E-67
KCNJ2	2.110951	1.20E-66
ENPP4	1.95321	1.85E-65
RND3	1.178004	2.15E-65
MXD1	-0.85307	1.35E-64
HCFC1	-0.62161	3.50E-64
PCDH10	3.455013	5.45E-64
BRPF1	-0.76964	1.55E-63
KDM6B	-0.99443	3.69E-63
SNIP1	-1.09409	4.07E-63
SH2D3C	2.97123	7.24E-63
SDE2	-0.70721	1.11E-62
SEMA4C	-0.66464	1.98E-62
PPARGC1A	1.63101	2.25E-62
KANSL2	-0.78707	3.04E-62
LRRTM1	1.081654	3.39E-62
RSRP1	-1.11998	5.95E-62
LRAT	3.305124	7.09E-62
ZIC1	1.517355	3.18E-61

ISL1	1.941984	3.26E-61
RSRC2	-0.70506	6.00E-61
GATA3	2.975829	6.06E-61
C1orf115	1.209347	6.36E-61
ARHGAP20	2.246948	7.16E-61
SHC4	3.011096	1.05E-60
CHST2	2.495475	1.22E-60
SLC40A1	4.152024	1.43E-60
FHDC1	0.89754	1.45E-60
OTX1	3.305815	5.11E-60
PABPC4	-0.60675	1.54E-59
SERTAD3	-1.41816	2.76E-59
RPRM	3.274522	4.48E-59
MT2A	1.05251	7.39E-59
TXNIP	-0.62264	7.95E-59
PDE4DIP	0.701862	1.51E-58
CLU	1.344278	1.72E-58
SFRP4	4.204933	1.90E-58
FILIP1	1.603346	2.88E-58
ELOVL4	1.118072	3.31E-58
SHOX2	1.568097	3.37E-58
EIF5	-0.61426	9.56E-58
HLF	1.565477	2.44E-57
EPC1	-0.61476	2.69E-57
ZFAND5	-0.60321	6.86E-57
FOXC1	1.031833	7.42E-57
TRPS1	0.667849	7.66E-57
SP8	2.447924	1.06E-56
ID4	-0.62198	1.07E-56
GABRG2	1.49543	1.29E-56
KDM2A	-0.58725	3.29E-56
SYT11	0.559939	3.29E-56
RAB39B	1.063833	4.97E-56
CSRP1	0.77878	7.57E-56
NCEH1	1.040605	8.12E-56
CRISPLD1	1.146728	8.54E-56
CCND2	7.591514	8.95E-56
KCNV1	1.617876	9.86E-56
JRKL	1.430315	1.84E-55
BAMBI	-0.56745	1.92E-55
CWC25	-0.71798	2.68E-55
CXCR4	0.756929	6.39E-55
DUSP1	-0.70967	1.18E-54
FLRT3	2.756458	1.30E-54
SUPT6H	-0.53406	1.92E-54
CDC42EP3	0.701324	5.74E-54
SLC46A1	0.778309	2.59E-52

HSPA2	1.749873	3.89E-52
ZNF462	0.936105	3.95E-52
HES1	0.869297	1.28E-51
HSPA1B	-0.89539	1.63E-51
DMRT3	2.874253	1.68E-51
TGFBR2	2.351513	2.01E-51
FGF9	1.682047	3.54E-51
RAB22A	-0.74518	4.24E-51
PCDH1	2.516565	6.65E-51
FGF19	0.629607	1.06E-50
TLX3	0.962583	1.59E-50
FRS2	-0.85446	2.96E-50
FOS	-0.84345	4.37E-50
MIDN	-0.83886	4.90E-50
IDI1	-0.61202	1.23E-49
CYP2U1	1.385996	1.35E-49
PNRC1	-0.93905	1.39E-49
MXD4	-0.81688	1.43E-49
SIRT1	-0.62361	1.71E-49
FOXL2	2.204268	1.85E-49
SSTR1	2.42374	2.48E-49
MED9	-1.00609	4.99E-49
TNFAIP2	-0.88772	8.93E-49
PPP1R3C	3.760324	4.50E-48
PPP1R3B	0.64916	4.54E-48
HOXC5	1.568938	4.97E-48
TRIM32	1.399039	8.12E-48
CDKN1A	0.946772	1.63E-47
EP300	-0.57467	1.78E-47
COQ10B	-0.69924	1.81E-47
RBM12B	0.732774	3.46E-47
PCDH17	0.923061	3.93E-47
NR2F2	1.670308	4.91E-47
TMCC1	0.911072	5.62E-47
ID1	-0.67539	7.21E-47
NAP1L3	1.651362	8.37E-47
NLGN1	2.999712	9.14E-47
CD274	3.414599	9.40E-47
TRIM59	0.851399	2.01E-46
TMEM30B	2.522956	2.59E-46
TMEM136	1.878959	3.09E-46
MBLAC2	1.193324	3.67E-46
NDNF	3.209608	4.72E-46
PPRC1	-0.59438	4.74E-46
BRD1	-0.56528	8.89E-46
GATA6	1.398124	2.37E-45
MSX2	0.823229	2.44E-45

KLF4	-0.53703	3.48E-45
ENC1	2.948032	3.93E-45
FAM83B	2.724217	4.12E-45
EMX2	3.224587	7.44E-45
PLK2	0.949537	9.00E-45
TPRN	-1.11586	1.29E-44
CRAMP1L	-0.68663	1.69E-44
UPF1	-0.51786	1.94E-44
RGMB	0.710897	3.28E-44
SLC35G2	1.464287	4.05E-44
MSL3P1	0.908454	5.57E-44
C3orf58	0.703191	7.46E-44
ELAVL2	0.914471	9.43E-44
CRYBG3	1.432092	9.63E-44
KPNA2	-0.52713	1.14E-43
KCTD5	-0.53356	1.36E-43
AKAP12	0.677144	2.07E-43
SALL2	0.935023	2.25E-43
H1F0	1.154277	2.75E-43
BHLHE40	-0.56857	2.86E-43
REXO1	-0.7125	5.18E-43
INO80	-0.50841	5.82E-43
YTHDF1	-0.61969	6.40E-43
SRSF2	-0.49158	6.61E-43
NAP1L2	1.484173	7.64E-43
EXO5	0.664833	7.66E-43
EVX1	2.401039	1.03E-42
SETD5	-0.54117	2.52E-42
MEIS3P1	1.479384	2.54E-42
AKAP8	-0.57307	2.64E-42
CTTNBP2	2.143028	3.18E-42
RGS16	2.262851	3.48E-42
ZRANB1	-0.53524	3.48E-42
HS3ST1	1.757649	3.53E-42
PPP1R10	-0.57902	3.71E-42
CD24	0.592673	5.13E-42
POLR1C	-0.81104	1.11E-41
LMCD1	1.108323	2.06E-41
ZNF367	-0.75489	2.41E-41
H2AFX	-0.53675	2.95E-41
USP42	-0.65555	3.02E-41
ZNF304	-0.7598	3.10E-41
ZNF394	-0.86832	3.42E-41
C2CD4A	3.68225	3.97E-41
SLITRK4	2.338765	5.42E-41
EGR1	-0.63447	6.23E-41
DNAJA1	-0.50692	7.29E-41

RGMA	2.107916	8.95E-41
SYNM	1.79397	8.98E-41
LOX	2.475991	1.25E-40
TMC5	1.491881	1.49E-40
HOXC13	2.260087	2.35E-40
PLIN2	0.623967	2.35E-40
F2RL1	1.546509	2.89E-40
ARID4B	-0.53747	2.91E-40
OLIG2	1.170943	3.10E-40
DMRTA1	1.596036	3.26E-40
TMEM64	0.81554	4.07E-40
SIPA1L2	1.257622	4.28E-40
NRARP	-0.55793	4.50E-40
MAP10	1.802583	4.82E-40
RBBP6	-0.65288	6.59E-40
CDKN2B	0.840805	6.93E-40
HEXIM1	-0.78526	9.51E-40
KIAA1551	0.748774	1.19E-39
FBXL12	-0.58442	1.19E-39
MPHOSPH1	-0.5689	1.50E-39
BDNF	0.984513	1.57E-39
IL10RA	1.316336	1.57E-39
TPBG	0.82943	1.64E-39
MT1X	0.595107	1.68E-39
HEYL	1.128795	1.97E-39
ZNF518B	0.706139	1.98E-39
MIEF1	-0.54148	2.00E-39
EPHA4	1.003981	3.63E-39
NXF1	-0.84677	3.87E-39
SRRM1	-0.53191	4.20E-39
SVEP1	2.635625	4.90E-39
SNHG1	-0.48728	5.11E-39
SLCO4C1	2.488969	5.91E-39
SYBU	2.773769	6.38E-39
PCDH8	2.485626	7.21E-39
USP44	2.054682	8.54E-39
FOXE1	2.15077	8.99E-39
TOP1	-0.58067	1.28E-38
KCNMB3	1.347138	1.58E-38
HOXD13	2.123734	1.75E-38
GSPT2	1.179882	2.35E-38
UBALD2	-0.81475	2.47E-38
IP6K2	-0.61009	2.73E-38
PRDM8	5.656895	2.99E-38
CCNT1	-0.50546	3.45E-38
ISL2	1.143591	3.50E-38
KLF3	0.54008	5.82E-38

CCNJL	1.791582	1.02E-37
DNAJB9	-1.31975	1.44E-37
TBC1D9	0.935763	1.47E-37
FAM181B	3.389062	1.49E-37
ZFHX2	1.724591	1.53E-37
PPP1R15B	-0.53922	2.57E-37
CSNK1D	-0.47526	2.86E-37
SEC24A	-0.51411	3.32E-37
IGFBP5	0.731116	3.33E-37
TIMP3	0.887908	3.80E-37
SFSWAP	-0.57655	4.05E-37
ZNF207	-0.43408	4.12E-37
WHAMM	-0.60121	4.13E-37
EIF1AD	-0.60944	5.96E-37
ZFHX4	2.408213	6.52E-37
LOC100507	-0.52882	8.62E-37
ZNF184	0.686393	1.10E-36
CCNF	-0.58526	1.12E-36
NPR3	0.895809	2.03E-36
ANKRD13C	-0.53465	2.52E-36
GFI1	2.448902	2.71E-36
SIN3A	-0.54184	2.93E-36
SRSF6	-0.45351	2.96E-36
GADD45G	-0.55814	3.37E-36
OLIG1	2.387505	3.45E-36
SRSF7	-0.47172	3.53E-36
IRX4	4.254694	3.99E-36
BASP1	1.440198	4.06E-36
SNX18	0.955464	6.21E-36
CEBPA	1.229257	6.43E-36
KMT2E	-0.57562	7.31E-36
IRF2BPL	0.63607	7.88E-36
SPATA2	-0.84118	8.89E-36
C17orf104	0.619187	9.29E-36
MEPCE	-0.47327	1.30E-35
AKAP8L	-0.62852	1.30E-35
PRRG4	1.357205	1.30E-35
HCP5	1.213575	1.35E-35
NPPC	1.813464	1.56E-35
TBCC	-0.74996	1.56E-35
SPTY2D1	-0.53133	1.60E-35
MT1G	0.900417	1.97E-35
PRKAA2	0.836148	2.14E-35
C17orf96	0.819217	2.23E-35
GATA2	-0.54687	3.86E-35
KDM7A	-0.47407	4.63E-35
RIMKLA	1.209139	5.75E-35

SBNO1	-0.49026	1.03E-34
FASTKD5	-0.59269	1.10E-34
UBN1	-0.47255	1.27E-34
ZNF335	-0.49913	1.38E-34
RANBP10	-0.54222	1.58E-34
IFIT5	1.363106	1.58E-34
CYR61	1.274289	2.07E-34
WTAP	-0.43094	2.09E-34
ING5	-0.59561	2.13E-34
RPP25	1.851491	2.66E-34
TICAM1	0.778343	2.68E-34
SLC25A44	-0.53223	4.71E-34
CLDN1	3.803849	4.97E-34
POM121C	-0.65206	5.47E-34
MCAM	1.666249	5.83E-34
SMAD7	-0.76978	1.13E-33
TMEM74	2.818234	1.15E-33
ARMC5	-0.64597	1.16E-33
CKS2	-0.49649	1.29E-33
PER1	-0.77261	1.31E-33
FGF2	1.048321	1.39E-33
F3	1.778654	1.39E-33
LRRC8A	-0.62893	1.58E-33
LRRC8B	0.803057	1.90E-33
BBS10	1.025511	1.95E-33
TRA2B	-0.47734	2.31E-33
WDR72	0.632025	2.42E-33
TXNDC11	-0.48352	4.15E-33
FAM110C	1.870219	5.90E-33
LIMA1	1.16943	6.62E-33
DCAF12L2	1.114058	1.06E-32
FNBP4	-0.46926	1.35E-32
INA	2.373355	1.40E-32
ILDR1	1.286612	1.41E-32
ZFX	-0.55507	1.44E-32
ZBTB21	0.532621	1.45E-32
PRDM1	1.275926	1.46E-32
UGDH-AS1	0.636902	2.10E-32
CHST10	1.326646	2.14E-32
ATF4	-0.38119	2.46E-32
IRF6	1.439498	2.46E-32
ING1	-0.75234	2.75E-32
FZD8	1.63776	3.11E-32
SEMA6B	2.211628	3.15E-32
INHBB	1.035353	3.15E-32
NEFH	1.709073	3.22E-32
ST6GAL2	1.122952	3.29E-32

TSHZ3	3.058762	3.54E-32
RBM5	-0.4852	3.57E-32
SSTR2	2.592063	3.93E-32
ESYT3	1.030939	3.93E-32
SHH	-0.60656	5.00E-32
ZCCHC3	-0.69254	5.12E-32
B3GALT2	1.413252	5.12E-32
MCL1	-0.41332	6.84E-32
CHD2	-0.51549	7.12E-32
CLP1	-0.91409	7.52E-32
ADRA2A	1.664069	8.86E-32
ZNF263	-0.48564	1.02E-31
ITPRIP	2.168336	1.19E-31
RAPH1	0.736675	2.18E-31
CDH1	1.06876	3.11E-31
PIAS4	-0.67401	3.87E-31
TRA2A	-0.4639	4.25E-31
EIF5A2	0.635316	4.58E-31
MFAP3L	2.752099	5.63E-31
SSFA2	0.518122	5.79E-31
KCTD12	1.335213	6.30E-31
NEUROD1	8.407547	7.47E-31
MIR22HG	-1.44139	7.70E-31
THBD	3.182276	8.11E-31
ZNF598	-0.4544	8.65E-31
CCDC174	-0.57136	9.50E-31
BHLHE41	4.682314	9.50E-31
SMEK1	-0.51747	1.08E-30
TMEM169	1.798065	1.09E-30
HEY2	2.484486	1.26E-30
C5orf30	0.582557	1.53E-30
ZC3H12C	0.88847	1.58E-30
ZNF608	0.56884	1.60E-30
MED13	-0.44707	1.60E-30
CDC34	-0.44203	1.72E-30
PRPF38B	-0.46486	1.75E-30
NR2E1	4.000747	2.39E-30
RNF25	-0.5719	2.55E-30
TNFSF10	1.326268	2.60E-30
TMEM151A	2.577732	2.67E-30
ABCB1	3.096483	2.69E-30
PLS1	0.631456	2.81E-30
RNF10	-0.4705	2.98E-30
ARL4D	1.371716	3.25E-30
SEC16A	-0.44842	3.32E-30
RLF	-0.4621	3.38E-30
SKIDA1	3.459868	3.86E-30

C16orf70	-0.62728	4.38E-30
RBM22	-0.43063	4.50E-30
ZNF92	-0.67972	6.10E-30
SNCAIP	2.290921	6.61E-30
ZNF689	-0.47466	6.84E-30
POLR2A	-0.59265	7.10E-30
YTHDF3	-0.43482	7.28E-30
RNF144B	1.169941	7.36E-30
TAGLN3	2.084945	7.46E-30
SAFB2	-0.50842	8.31E-30
PYGO1	0.728606	9.18E-30
TGFBR3	1.290214	1.05E-29
SIK1	-0.68735	1.09E-29
ARL6IP1	-0.46209	1.09E-29
NAT8L	0.688854	1.64E-29
FANCF	1.207011	1.73E-29
AP1G1	-0.45951	1.90E-29
SLC2A1	-0.41673	2.46E-29
MAB21L3	0.834226	2.83E-29
FZD10	2.600437	2.97E-29
SMNDC1	-0.55204	2.99E-29
SON	-0.44876	3.02E-29
TMCC2	2.772739	3.48E-29
RASD1	-0.89896	3.69E-29
PHLDA1	0.390562	3.71E-29
FOXQ1	-0.48167	3.86E-29
HAS3	0.534272	5.18E-29
ADRM1	-0.46202	5.57E-29
EIF2AK3	-0.53975	5.63E-29
SRSF3	-0.40788	5.91E-29
TERF2IP	-0.44096	7.35E-29
GNL3	-0.4332	7.38E-29
CDK12	-0.42123	7.39E-29
CALCB	1.88643	7.73E-29
ZBTB11	-0.55947	7.75E-29
TMEM55B	-0.55775	8.34E-29
CCNA1	0.935622	9.32E-29
BRAP	-0.48038	1.06E-28
DOHH	-0.48569	1.11E-28
TMEM200A	2.714369	1.62E-28
IGSF11	2.096674	1.63E-28
KCND2	1.135249	1.97E-28
SETD1B	-0.87786	2.52E-28
SRRT	-0.42243	2.85E-28
ZNF691	0.806674	3.32E-28
PRRC2A	-0.37849	3.70E-28
ZKSCAN1	-0.52925	3.78E-28

RBM25	-0.45139	4.29E-28
IRF1	0.769386	5.00E-28
P2RY1	2.384011	5.29E-28
ITPRIPL2	1.047996	6.61E-28
SH3BGRL2	0.646668	7.49E-28
DSG4	2.503046	7.63E-28
RNF139	-0.48922	8.77E-28
DAPK3	-0.51764	9.21E-28
KMT2B	-0.57549	9.61E-28
LRRC8C	0.846946	1.00E-27
SH3GL2	0.991511	1.10E-27
CDC25A	-0.59486	1.15E-27
KCNQ1OT1	0.678561	1.29E-27
RGL1	2.626716	1.38E-27
WEE1	-0.83659	1.49E-27
MICB	0.622751	1.59E-27
MEX3C	-0.49612	1.63E-27
MLLT3	1.115844	1.63E-27
BRF2	-0.51911	2.10E-27
ENPP1	1.110712	2.16E-27
LARP4B	-0.43442	2.77E-27
DYRK1A	-0.49307	2.80E-27
HABP2	0.942594	2.97E-27
HIRA	-0.54059	4.29E-27
RRN3	-0.43587	6.36E-27
GLIDR	0.732103	7.19E-27
CBX4	-0.43681	7.51E-27
NOTCH2	0.568306	7.54E-27
RSPRY1	-0.52735	1.10E-26
MED1	-0.4009	1.12E-26
ZSCAN12	0.964783	1.26E-26
PER2	-0.49843	1.36E-26
SOX1	1.462471	1.36E-26
PABPC5	2.098634	1.42E-26
TMPRSS2	1.690758	1.51E-26
LFNG	-0.45511	1.63E-26
SCAF4	-0.43989	1.79E-26
FBXO31	-0.4724	1.84E-26
HNRNPA0	-0.38753	1.98E-26
SEPP1	1.333042	2.01E-26
FOXF1	1.969971	2.03E-26
PABPC4L	4.054332	2.55E-26
TMEM170I	0.981426	2.74E-26
RABGEF1	-0.5899	2.76E-26
CDCA8	-0.40256	2.91E-26
DLX1	0.655997	3.00E-26
PRICKLE1	2.166492	3.07E-26

TMEM246	1.665634	3.13E-26
MB21D2	1.106772	3.15E-26
TACC2	0.574686	3.52E-26
WDR74	-0.44217	3.64E-26
C1GALT1	0.58008	3.89E-26
MANSC1	0.735726	4.09E-26
RASEF	0.555688	4.18E-26
PRDM13	1.168746	4.35E-26
UBC	-0.55171	4.70E-26
RBM3	-0.41726	4.82E-26
FOXN2	0.699156	5.20E-26
NFX1	-0.42502	5.64E-26
NOTCH1	-0.49952	5.99E-26
CHD9	0.540207	6.13E-26
CYP1A1	0.719283	6.19E-26
WDR89	0.751961	6.72E-26
ZFP42	2.594575	8.17E-26
CCNG2	-1.14168	8.66E-26
STC1	2.683775	8.96E-26
INSIG1	-0.44006	8.97E-26
IFRD1	-0.45185	8.99E-26
TUSC2	-0.62946	9.84E-26
PHKG2	-0.47325	1.12E-25
PAX9	3.318657	1.15E-25
NOP16	-0.45014	1.16E-25
LCA5	1.858587	1.24E-25
PHF23	-0.76579	1.24E-25
ABHD10	0.677033	1.29E-25
RUNX3	1.272043	1.39E-25
NEUROG2	3.331833	1.68E-25
AMMECR1	-0.46327	1.80E-25
PIP5K1A	-0.41913	1.83E-25
CDR2	-0.3708	2.21E-25
NKX2-8	2.933111	2.52E-25
CACTIN	-0.42343	2.64E-25
FBRS	-0.52526	2.70E-25
HSPA5	-0.39504	2.94E-25
HAS2	1.81623	2.95E-25
EIF1	-0.41092	3.10E-25
FJX1	1.326872	3.19E-25
SPP1	0.454777	3.60E-25
KIAA0907	-0.45814	3.69E-25
ATMIN	-0.45543	3.75E-25
BMP3	1.973366	3.75E-25
YY1	-0.44894	3.75E-25
CDKN1B	-0.50828	3.83E-25
EGLN3	2.149229	3.83E-25

RASSF5	2.606424	3.83E-25
NREP	0.658428	3.96E-25
GABPB1	-0.48622	4.08E-25
ZC3H7A	-0.40091	4.08E-25
KL	1.119942	4.71E-25
POM121	-0.49199	5.04E-25
ZNF879	1.482855	5.11E-25
PPM1D	-0.48343	7.09E-25
ATOH1	3.522718	8.10E-25
AKIRIN1	-0.36305	8.82E-25
ZBED8	1.546224	9.51E-25
MAB21L1	2.587285	1.00E-24
NRIP3	1.016192	1.09E-24
COL12A1	3.042257	1.13E-24
C3orf80	1.960923	1.15E-24
THUMPD1	-0.40309	1.16E-24
TBC1D2B	0.730475	1.26E-24
YY1AP1	-0.42914	1.28E-24
TIPARP	-0.40515	1.30E-24
GPATCH3	-0.63553	1.31E-24
ZIC5	1.615106	1.34E-24
MT1M	1.423072	1.34E-24
NTF3	1.744178	1.35E-24
ZNF287	1.191469	1.40E-24
RAB5A	-0.40986	1.44E-24
NOV	2.832183	1.50E-24
CLK3	-0.44528	1.56E-24
CDCP1	0.784975	1.57E-24
NR2F1	3.345564	1.62E-24
HAND1	1.364032	1.72E-24
TTBK1	2.049219	1.81E-24
SRSF4	-0.38304	1.82E-24
RASSF8	0.881483	2.06E-24
RC3H1	-0.57856	2.19E-24
HIVEP2	0.926545	2.41E-24
ZNF710	0.470344	2.52E-24
KRT18	-0.50323	2.52E-24
INTS6	-0.44598	2.62E-24
ARHGEF38	1.418813	2.83E-24
KMT2D	-0.51142	2.97E-24
FOXD3	2.454609	3.05E-24
ASB6	-0.52958	3.14E-24
NOM1	-0.43045	3.22E-24
HOXA5	1.388851	3.79E-24
ZFP82	0.890826	3.82E-24
TUFT1	-0.39366	4.27E-24
ARHGEF26	0.626049	4.57E-24

NES	0.624575	5.03E-24
TSLP	3.696634	5.03E-24
RFWD3	-0.4124	5.52E-24
SHISA2	0.902556	5.53E-24
TIAL1	-0.44602	5.84E-24
CDCA5	-0.38275	6.01E-24
SOX4	0.385169	6.07E-24
EIF4G2	-0.364	6.84E-24
ZBTB43	-0.66924	6.87E-24
KIF23	-0.37113	7.89E-24
TNFRSF11B	0.754811	8.28E-24
PAF1	-0.38731	9.24E-24
RBM24	4.004355	1.05E-23
THEM6	1.177721	1.08E-23
DSC3	1.231895	1.11E-23
RFX7	0.521848	1.29E-23
TIFA	1.162858	1.31E-23
PTGS2	4.433682	1.36E-23
SLU7	-0.45195	1.47E-23
BCLAF1	-0.37001	1.54E-23
PPAP2B	1.671352	1.55E-23
RASSF10	4.399536	1.72E-23
JMJD6	-0.4584	1.73E-23
FBXO28	-0.41382	1.74E-23
UBFD1	-0.37951	1.87E-23
SLFN11	1.429648	1.93E-23
NDP	1.844214	2.19E-23
ARIH1	-0.36786	2.25E-23
CXXC4	2.85171	2.31E-23
LPAR3	3.066521	2.31E-23
ZCCHC12	2.427895	2.32E-23
DNAJC6	0.569868	2.56E-23
SUPT5H	-0.38233	2.94E-23
JAG1	0.456391	3.04E-23
SMG8	-0.49318	3.17E-23
PHF12	-0.54164	3.22E-23
NPLOC4	-0.37615	3.39E-23
GOLGA3	-0.46681	3.59E-23
FAF2	-0.34951	3.65E-23
SBDS	-0.42656	3.92E-23
DDX6	-0.41447	3.95E-23
ZFP69	0.823888	4.39E-23
TUBB6	0.549045	4.46E-23
BEND3	0.915404	4.58E-23
FAM91A1	-0.43269	4.69E-23
SYVN1	-0.61232	4.77E-23
ATF7IP	0.44277	5.22E-23

WDR82	-0.41629	5.25E-23
FGF13	0.670685	5.29E-23
CDK5R2	4.240531	5.54E-23
RBMS1	1.319307	5.92E-23
CD2BP2	-0.38913	6.37E-23
PPP2CA	-0.36274	6.51E-23
KIAA0040	1.122573	7.68E-23
LHX2	2.214964	8.35E-23
SUCO	-0.37789	9.07E-23
DSP	-0.39276	9.20E-23
S1PR3	1.371842	9.48E-23
SF3B1	-0.33373	1.04E-22
FZR1	-0.49824	1.06E-22
PRCC	-0.42658	1.20E-22
DIDO1	-0.54752	1.25E-22
HOXA10	1.079095	1.38E-22
KIAA1462	1.897275	1.41E-22
EIF4A3	-0.36385	1.81E-22
ZNF830	-0.52667	1.88E-22
PCDH9	1.320858	1.97E-22
SETD1A	-0.41697	2.28E-22
EN1	2.307256	2.29E-22
RGAG4	1.334601	2.34E-22
RAG1	2.319772	2.56E-22
CRY2	-0.58079	2.59E-22
CLIC6	0.695783	2.60E-22
MFHAS1	0.557794	2.71E-22
ADAM19	0.544648	2.93E-22
DEPDC7	0.533873	2.93E-22
ARRDC3	0.562393	3.14E-22
LRRC10B	0.936722	3.20E-22
SART3	-0.35922	3.95E-22
ZNF786	0.777844	3.95E-22
ZNF607	0.796194	4.12E-22
TRIB2	0.444506	4.71E-22
NR3C1	0.473984	4.88E-22
USP36	-0.415	4.95E-22
T	2.299268	5.09E-22
EAF1	-0.46753	5.17E-22
PPP1R3D	1.174668	5.45E-22
SLC30A1	-0.36152	5.66E-22
PPP1R9B	0.628289	6.04E-22
GTF2B	-0.53108	6.08E-22
TMEM55A	0.894816	6.22E-22
FEM1C	-0.43473	6.32E-22
KAT6B	-0.43145	6.55E-22
GLUD2	1.367231	6.99E-22

UBE2J2	-0.39576	7.88E-22
TSPYL5	0.699871	7.91E-22
ZBTB38	0.407538	8.37E-22
SRF	-0.43276	8.65E-22
SENP2	-0.34999	9.69E-22
MORC3	-0.45054	9.69E-22
PDPK1	-0.39633	1.01E-21
MET	0.905015	1.11E-21
KBTBD7	1.04232	1.11E-21
CTB-174D1	1.098106	1.12E-21
LRP12	0.88556	1.14E-21
USP38	-0.43069	1.17E-21
EFR3A	0.553295	1.19E-21
FEZF1	2.850272	1.26E-21
UBE2Z	-0.38491	1.33E-21
NACC1	-0.40538	1.34E-21
KCNN2	1.851398	1.34E-21
VGF	2.312446	1.40E-21
CCDC82	0.437917	1.49E-21
GNB4	1.092505	1.61E-21
PLEKHF2	0.761512	1.73E-21
WT1	0.947217	1.82E-21
SERTAD1	-0.59752	1.90E-21
INPP5J	1.131376	1.94E-21
VCP	-0.34708	1.95E-21
AMOTL1	0.974827	1.98E-21
IFNLR1	0.944735	1.98E-21
ZDBF2	0.704008	2.01E-21
KRT20	1.368134	2.24E-21
ZDHHC7	-0.49799	2.36E-21
PAPOLG	-0.44899	2.37E-21
TUBA1C	-0.36359	2.51E-21
PLAGL2	-0.56167	2.64E-21
ZKSCAN3	0.848677	2.77E-21
KLF9	-0.57169	2.99E-21
GATM	1.075279	3.02E-21
PCSK5	0.649212	3.09E-21
SUPV3L1	-0.38183	3.21E-21
C11orf96	1.718387	3.30E-21
JUNB	-0.62709	3.52E-21
KAT6A	-0.39008	3.75E-21
FHL2	0.950526	4.19E-21
BUD13	-0.51366	5.17E-21
FAXC	0.560448	5.70E-21
ZNF7	-0.55877	5.78E-21
SPSB4	1.518271	6.22E-21
DDX3X	-0.35879	6.24E-21

LOC440173	1.527644	6.65E-21
ATG14	-0.68408	6.68E-21
GCC1	-0.49279	6.77E-21
HMX2	2.459836	6.91E-21
FBXO38	-0.41098	6.91E-21
TRPC1	1.876945	6.91E-21
VCPIP1	-0.46742	7.13E-21
CHSY1	0.512389	7.29E-21
HIAT1	-0.46519	8.65E-21
KIAA1024	0.654062	8.68E-21
PPFIA1	-0.39001	8.87E-21
CIC	-0.5903	9.21E-21
RSPO2	4.043929	9.21E-21
ACVR1B	0.691164	9.29E-21
PCYT1B	0.802353	9.35E-21
NRBP1	-0.38051	9.75E-21
GRHL1	1.095302	1.07E-20
RBM15	-0.457	1.07E-20
ASB13	0.567432	1.09E-20
PAQR9	3.259214	1.12E-20
LOC730101	0.550442	1.16E-20
FNIP1	-0.45875	1.19E-20
APLN	2.075649	1.27E-20
SIX2	1.320876	1.29E-20
SLC12A7	-0.47063	1.30E-20
SYN1	2.475954	1.31E-20
MYCL	0.646953	1.35E-20
RPRD1B	-0.36079	1.52E-20
CCDC94	-0.51981	1.59E-20
KLHL15	-0.6144	1.62E-20
BDP1	-0.41886	1.67E-20
PSMD11	-0.31393	1.77E-20
OCLN	0.93076	1.79E-20
ZNF827	0.571043	1.95E-20
MEF2D	-0.5383	2.17E-20
ZIC2	1.288462	2.22E-20
C6orf62	-0.58472	2.37E-20
DNTTIP2	-0.38166	2.50E-20
CRK	-0.36236	2.57E-20
DACT1	0.683508	2.94E-20
ETF1	-0.35757	3.07E-20
DEDD2	-0.41001	3.23E-20
MTF1	-0.45537	3.30E-20
DYRK1B	-0.78144	3.31E-20
KIT	1.170079	3.33E-20
MARK1	0.587575	3.39E-20
TRIM2	0.74687	3.52E-20

LSM12	-0.3583	3.63E-20
CNOT1	-0.34916	3.72E-20
ZNF329	1.528136	3.85E-20
INCENP	-0.35259	3.95E-20
OSBP	-0.34383	3.98E-20
TRAPPC10	-0.49389	4.06E-20
RASGRP1	1.336503	4.31E-20
CDCA2	-0.415	4.32E-20
TMEM51	1.115036	4.74E-20
AKT1S1	-0.44698	4.88E-20
F11R	-0.34069	5.08E-20
KIAA0895	0.763098	5.15E-20
KLF10	-0.42166	5.16E-20
GAK	-0.33208	5.25E-20
MGAT1	-0.42526	5.26E-20
C10orf118	-0.44551	5.26E-20
FZD2	1.352194	5.46E-20
TOPORS	-0.38257	5.67E-20
LIN54	-0.48288	5.68E-20
HAND2	0.934432	5.73E-20
GCNT1	0.528541	6.00E-20
FASN	-0.36783	6.12E-20
SRSF11	-0.34783	6.26E-20
PHRF1	-0.37579	6.95E-20
NECAP1	-0.38329	7.00E-20
EAPP	-0.49098	7.31E-20
LIPG	1.011238	7.40E-20
MEX3B	0.807495	8.46E-20
YRDC	-0.53299	8.47E-20
FAM193A	-0.46365	8.54E-20
FYTTD1	0.389294	8.73E-20
STK40	-0.42337	8.92E-20
LATS1	-0.39676	9.10E-20
HUNK	0.406644	9.44E-20
KIF5B	-0.39929	9.52E-20
NUP50	-0.45161	1.01E-19
HOXA11	2.180096	1.09E-19
ERF	-0.57725	1.16E-19
MT1A	1.924744	1.16E-19
CBX6	0.636625	1.20E-19
AASS	0.751731	1.20E-19
RHOU	1.647859	1.26E-19
B4GALT5	0.452114	1.31E-19
ZNF331	0.57824	1.32E-19
TEX19	1.758525	1.34E-19
NRXN2	1.88569	1.34E-19
EMB	0.656161	1.35E-19

AKAP7	1.082353	1.43E-19
MT1F	0.725695	1.47E-19
POLD3	-0.40027	1.47E-19
TFAP2C	2.880144	1.53E-19
NCOA4	-0.36709	1.54E-19
HOXC6	1.827977	1.54E-19
ZNF202	-0.50088	1.58E-19
CDH6	2.517496	1.99E-19
CHERP	-0.38362	2.19E-19
DDX5	-0.49428	2.20E-19
AMD1	-0.377	2.31E-19
BZW1	-0.33087	2.34E-19
BMP6	0.920133	2.43E-19
ZFP62	0.610328	2.46E-19
RIOK3	-0.40409	2.57E-19
DUSP16	-0.34464	2.64E-19
ELF2	-0.47039	2.65E-19
SDC2	2.00983	2.79E-19
MAP1LC3B	-0.4014	2.95E-19
C1orf52	-0.5045	2.96E-19
MARK3	-0.37777	2.96E-19
ADAMTS15	1.585341	3.18E-19
BMP2	2.390189	3.20E-19
TP53INP2	-0.64476	3.20E-19
FOXP1	0.742497	3.23E-19
TMCC3	1.846513	3.25E-19
SOBP	2.112775	3.29E-19
ZBTB5	-0.3975	3.30E-19
SHF	0.692112	3.31E-19
ARID1A	-0.36838	3.46E-19
TRIM11	-0.39101	3.50E-19
ARID4A	-0.45114	3.51E-19
DNAJC2	-0.36529	3.51E-19
UGT8	1.094293	3.56E-19
OXSM	1.102168	3.64E-19
PHYHIPL	2.243422	3.66E-19
SLITRK5	3.143838	3.80E-19
TAOK2	-0.44854	3.86E-19
SCAMP5	0.901223	4.07E-19
PPP2R2D	-0.48408	4.07E-19
POLDIP3	-0.34282	4.26E-19
ABCF3	-0.34923	4.34E-19
SYNRG	-0.37863	4.45E-19
LOC64236(	1.738897	4.47E-19
TLR6	2.146125	4.60E-19
ZNF282	-0.42182	4.97E-19
MTA2	-0.31426	5.41E-19

ZSWIM5	0.520522	5.44E-19
KCTD21	0.77788	5.85E-19
KBTBD6	0.531449	6.00E-19
CCDC137	-0.43723	6.09E-19
CEP120	0.503636	6.37E-19
PLOD2	0.403818	6.68E-19
PSMD12	-0.37204	6.68E-19
MAD2L1BP	-0.47451	6.72E-19
ZC3H3	-0.48116	6.87E-19
PALLD	0.410257	7.90E-19
SRSF5	-0.4469	8.45E-19
TTF1	-0.46779	8.77E-19
SERPINB9	1.777744	8.95E-19
TBP	-0.35978	9.66E-19
INSM1	0.58938	9.87E-19
MAEA	-0.35559	1.10E-18
FAM83D	-0.35272	1.18E-18
CRMP1	1.352736	1.19E-18
E2F3	-0.37609	1.20E-18
SMEK2	-0.36243	1.24E-18
KIAA0232	-0.65762	1.41E-18
B4GALT6	0.83806	1.52E-18
LURAP1L	3.400128	1.63E-18
HIC2	-0.69642	1.69E-18
MNT	-0.59659	1.73E-18
TMEM9B	-0.66672	1.88E-18
SFMBT2	1.590717	1.91E-18
TPRA1	-0.39785	1.94E-18
VPRBP	-0.36077	2.03E-18
YTHDF2	-0.3433	2.13E-18
NOD2	1.165691	2.16E-18
UBOX5	-0.80743	2.16E-18
PCNXL3	-0.39189	2.27E-18
IL1R1	1.062311	2.31E-18
TSC22D1	-0.34614	2.33E-18
L3MBTL2	-0.40188	2.37E-18
MT1H	2.319801	2.62E-18
PTPRE	-0.40569	2.64E-18
USP8	-0.33632	2.66E-18
IRS2	0.635375	2.68E-18
RRP12	-0.36135	2.68E-18
GSE1	-0.39146	2.69E-18
RIN2	1.680828	2.70E-18
HCCS	-0.38419	2.78E-18
FOXB1	2.911603	2.91E-18
LITAF	0.413442	2.97E-18
BRD7	-0.29237	2.97E-18

AREL1	-0.45901	3.25E-18
TNFRSF19	0.442913	3.48E-18
VCPKMT	-0.59749	3.65E-18
GLYR1	-0.33623	3.82E-18
NIP7	-0.35482	4.02E-18
ALG2	-0.44453	4.15E-18
RUSC2	0.667878	4.19E-18
HNRNPH1	-0.37902	4.19E-18
FAM103A1	-0.47926	4.23E-18
FKBP7	0.902494	4.42E-18
DOK1	0.776637	4.79E-18
LINC00052	0.955549	4.88E-18
ATG101	-0.37962	4.88E-18
FBXO5	-0.48903	5.12E-18
LINC00888	0.927696	5.12E-18
KDM4A	-0.30184	5.25E-18
PRPF4B	-0.36064	5.61E-18
IP6K1	-0.453	5.90E-18
SHCBP1	-0.45501	6.15E-18
RGS2	0.48793	6.26E-18
AKAP10	-0.51931	6.27E-18
VPS11	-0.45017	6.45E-18
ZDHHC5	-0.38826	6.64E-18
MT1E	1.101928	6.85E-18
RIPK2	0.390494	7.51E-18
ZBTB7A	-0.54394	8.01E-18
ZNF532	0.409933	8.17E-18
PPTC7	-0.50938	8.46E-18
CCND1	-0.39769	8.66E-18
ANXA2R	1.680311	9.12E-18
ASPHD2	1.586975	9.12E-18
KCNK5	1.0903	9.15E-18
PRKG2	1.537097	9.33E-18
ABHD17B	-0.49061	9.40E-18
POLR3G	0.453698	1.05E-17
SAP130	-0.39258	1.08E-17
EPC2	-0.355	1.10E-17
ZNF557	-0.63216	1.12E-17
C17orf51	0.691229	1.27E-17
TIGD7	0.669126	1.30E-17
BRIX1	-0.33502	1.48E-17
SCAF1	-0.48073	1.53E-17
TESC	0.667958	1.58E-17
UBAP2	-0.42083	1.75E-17
TBX18	0.525919	1.85E-17
RAB11FIP2	0.567795	1.86E-17
AAR2	-0.37854	1.91E-17

PPP1R15A	-0.50595	1.94E-17
NHLH1	-1.87832	1.95E-17
BEX4	0.641236	1.95E-17
ZNF296	-1.04677	2.05E-17
AHCTF1	-0.33822	2.19E-17
CA2	0.659467	2.25E-17
MTHFR	-0.47378	2.32E-17
EBF3	2.10122	2.59E-17
IRX3	1.0634	2.59E-17
OSER1	-0.5104	2.59E-17
KCNQ5	0.481981	2.64E-17
NCALD	1.422399	2.64E-17
TCERG1	-0.29972	2.82E-17
GPR63	1.132852	2.98E-17
EIF4A2	-0.29823	3.11E-17
LEMD3	-0.42493	3.31E-17
ZNF503	-0.39724	3.32E-17
HELZ	-0.44193	3.39E-17
COG3	-0.40008	3.45E-17
AFF4	-0.36442	3.57E-17
WAC	-0.30771	3.90E-17
ELF1	0.592062	3.93E-17
CRKL	-0.4127	3.94E-17
ZNF75A	0.426713	3.96E-17
CSTF2T	0.417736	3.97E-17
SETD8	-0.33854	4.12E-17
SRSF1	-0.29671	4.25E-17
NSDHL	-0.40326	4.39E-17
ACBD3	-0.35052	4.69E-17
ZFP37	0.83052	4.70E-17
ZNF112	1.100617	4.76E-17
SLC35F2	0.459672	4.78E-17
LOC10050€	1.307806	4.86E-17
ABCC9	0.692386	5.03E-17
LIPT2	0.808038	5.18E-17
NDN	0.855267	5.32E-17
CSRP2	0.700092	5.39E-17
DGKE	0.481942	5.61E-17
CAMK2N1	1.56762	5.61E-17
UBE2G1	-0.42296	5.76E-17
TBC1D31	0.383907	5.97E-17
LRIG3	0.653896	5.99E-17
MED17	-0.38042	6.10E-17
WDR43	-0.33654	6.16E-17
CLCN6	-0.49863	6.33E-17
FOXA2	-0.44635	6.40E-17
LINC00491	0.998477	6.66E-17

ARID3B	-0.75381	7.00E-17
RBM43	1.074803	7.26E-17
LUC7L	-0.47662	7.42E-17
NDEL1	-0.41621	7.78E-17
TPM1	0.437723	7.80E-17
DGCR8	-0.43409	7.81E-17
CNOT3	-0.53404	8.41E-17
ZEB2	2.768125	8.88E-17
SLC25A30	0.697716	8.93E-17
PKIA	2.445328	9.28E-17
CREBBP	-0.32801	9.51E-17
CARKD	-0.43164	1.02E-16
C2orf72	1.399972	1.06E-16
C16orf72	-0.32425	1.06E-16
TICRR	-0.38183	1.08E-16
WAPAL	-0.36564	1.13E-16
KCTD2	-0.46183	1.20E-16
HHEX	1.090692	1.21E-16
TP53	-0.77297	1.23E-16
NRIP1	0.472745	1.24E-16
SLBP	-0.3169	1.27E-16
POFUT2	-0.51665	1.27E-16
SCAF8	-0.32764	1.29E-16
MYC	-0.35085	1.32E-16
LRRK2	1.824002	1.34E-16
DDX39A	-0.34786	1.38E-16
AHR	0.432462	1.38E-16
CLASP2	0.363526	1.39E-16
ZNF721	-0.33521	1.52E-16
RBBP5	-0.39423	1.56E-16
VEGFA	-0.31017	1.58E-16
ZC3H13	-0.35092	1.64E-16
BEX1	0.584463	1.74E-16
MYNN	-0.39987	1.74E-16
PLEKHA1	0.55752	1.79E-16
SPRED3	-1.35784	1.81E-16
C17orf85	-0.45347	1.84E-16
EFCAB14	-0.42104	1.98E-16
PIM2	-0.8505	2.12E-16
MFN2	-0.30195	2.17E-16
NFKBIA	-0.54426	2.25E-16
DHX38	-0.4059	2.28E-16
SULT4A1	1.458804	2.29E-16
AURKA	-0.33668	2.47E-16
ZNF234	1.028265	2.58E-16
HYLS1	0.88274	2.65E-16
SCYL1	-0.39642	2.73E-16

MAFF	-0.5277	2.84E-16
HMGXB4	-0.35302	2.96E-16
ZFP64	0.614143	3.16E-16
RPS6KL1	0.709369	3.28E-16
XDH	2.519594	3.30E-16
LCMT2	0.554946	3.51E-16
ZKSCAN5	-0.39966	3.82E-16
ETNPPL	1.622122	3.86E-16
SNAI1	1.052075	3.88E-16
ZNF239	1.272693	3.95E-16
SPAG17	2.201683	4.11E-16
CANT1	-0.49804	4.11E-16
ESCO1	-0.44969	4.28E-16
CCDC110	1.008862	4.38E-16
CMTM3	0.962197	4.40E-16
KAT5	-0.34938	4.43E-16
LOC65227€	-0.64065	4.66E-16
ZNF280D	0.342839	4.82E-16
SELT	-0.32089	5.23E-16
OSBPL2	-0.45581	5.28E-16
HOXB9	0.523143	5.40E-16
PTCH1	1.261578	5.40E-16
ZNF280A	1.342939	5.85E-16
TCF12	0.319792	5.92E-16
NFIL3	0.558316	5.99E-16
PLK1	-0.32333	6.17E-16
GDPD1	1.265415	6.20E-16
ZBTB49	-0.71288	6.31E-16
DNMBP	0.732306	6.50E-16
TXNL1	-0.34259	6.53E-16
DSC2	0.755319	6.68E-16
RSBN1	-0.55047	7.03E-16
RNF40	-0.30602	7.15E-16
PTP4A1	-0.31054	7.21E-16
ATG4D	-0.37172	7.31E-16
CITED2	0.757544	8.11E-16
MOB3A	0.594691	8.18E-16
IRX2	-0.49249	8.66E-16
UBAP2L	-0.28358	8.69E-16
KCNG3	1.10087	8.84E-16
TAF1	-0.34679	9.49E-16
SLAIN1	0.53624	9.51E-16
SLC25A25	-0.37126	9.79E-16
SPG20	0.856799	9.97E-16
TSC1	-0.50594	1.10E-15
GSK3A	-0.36945	1.10E-15
BCL2L1	-0.30743	1.15E-15

RAP2B	0.445607	1.23E-15
EFNA4	0.921508	1.23E-15
EIF3A	-0.35451	1.25E-15
YTHDC1	-0.29948	1.27E-15
C9orf40	0.634567	1.28E-15
GOLPH3	-0.28175	1.31E-15
TMEM115	-0.47203	1.32E-15
NGDN	-0.47292	1.42E-15
HNRNPAB	-0.2707	1.43E-15
TSPYL4	0.622063	1.46E-15
MBIP	-0.4214	1.54E-15
KDM5C	-0.32303	1.68E-15
CLDN3	0.577558	1.79E-15
TACC3	-0.3426	1.80E-15
PARM1	0.580523	1.81E-15
CDC6	-0.36566	1.85E-15
ZNF232	0.818936	1.91E-15
FTSJ3	-0.32047	1.92E-15
ZNF571	1.095346	1.94E-15
ACVR1C	2.615842	1.99E-15
FIGNL1	0.510071	2.06E-15
SLC26A9	1.639646	2.06E-15
NPTX1	1.960682	2.08E-15
PROK2	2.672401	2.10E-15
TERF2	-0.32363	2.12E-15
SRP68	-0.31839	2.32E-15
ARF4	-0.3223	2.45E-15
CENPN	-0.33435	2.67E-15
UTP14A	-0.35487	2.69E-15
CAPRIN2	0.570867	2.92E-15
ZNF10	0.612327	2.94E-15
RPS6KB1	-0.36864	2.96E-15
TFIP11	-0.49328	3.09E-15
EIF3B	-0.27261	3.25E-15
NRP2	-0.30869	3.26E-15
SF3B4	-0.41326	3.41E-15
EXOC8	-0.44313	3.41E-15
CLCN3	0.404918	3.47E-15
PTGER3	1.613262	3.49E-15
SH2D4A	0.560885	3.49E-15
BMP8A	0.869836	3.51E-15
ITPKC	-0.65358	3.61E-15
ARRDC4	-0.29962	3.74E-15
ZBTB14	0.808608	3.75E-15
FGF4	1.537334	4.20E-15
PHF21A	0.481078	4.25E-15
BSPRY	0.847008	4.67E-15

HRH1	2.544601	5.13E-15
DDX20	-0.36076	5.25E-15
WDR37	-0.61383	5.29E-15
C22orf46	1.061305	5.35E-15
SNHG16	-0.36021	5.63E-15
NRN1	1.064158	5.80E-15
PTGER4	0.463056	6.05E-15
LOC150776	-0.36827	6.32E-15
WDR62	-0.32955	6.40E-15
KIAA0947	-0.33707	6.41E-15
TCEB3	-0.34779	6.43E-15
RAB30	0.465425	6.58E-15
ZNF230	0.751549	7.06E-15
SHB	-0.32948	7.09E-15
FZD4	1.219347	7.13E-15
GORASP2	-0.27935	7.13E-15
ZFP36L1	0.404483	7.41E-15
COTL1	0.81222	7.57E-15
LRRN1	0.961582	7.63E-15
RNF4	-0.3457	7.75E-15
STARD13	0.897488	7.89E-15
KIAA0922	0.614935	8.25E-15
GUCD1	-0.40719	8.48E-15
PTRF	0.846392	8.66E-15
C5orf51	-0.3329	8.73E-15
SPATA5L1	-0.36548	8.73E-15
PIK3R3	-0.31717	9.00E-15
SP2	-0.37748	9.35E-15
TRAM1L1	1.561663	9.41E-15
ATAD2	-0.28134	9.66E-15
ZNF792	1.698738	9.67E-15
TUBB2A	-0.35805	1.03E-14
LHX6	0.799884	1.11E-14
SLC39A9	-0.4067	1.12E-14
DLGAP3	1.437901	1.16E-14
TRIP11	-0.38795	1.17E-14
C10orf126	0.578975	1.24E-14
AK4	0.43735	1.28E-14
CLDND1	0.308133	1.32E-14
SIRT7	-0.50207	1.33E-14
HOXB7	0.566864	1.34E-14
SLC20A2	-0.50384	1.41E-14
WNT11	0.650804	1.42E-14
GPR180	0.464473	1.47E-14
AP3D1	-0.2839	1.47E-14
BRD8	-0.37358	1.47E-14
NEIL3	0.503823	1.48E-14

YME1L1	-0.27846	1.59E-14
BIRC3	2.085223	1.61E-14
ST7	0.486246	1.64E-14
IPPK	-0.5331	1.75E-14
SIX4	0.581992	1.75E-14
NOP58	-0.29349	1.77E-14
KBTBD11	1.416694	1.80E-14
XAB2	-0.31502	1.85E-14
NEU1	-0.44557	2.01E-14
LOC10192(	1.940972	2.09E-14
ZEB1-AS1	1.27072	2.19E-14
ACO2	-0.29512	2.19E-14
POU3F2	0.385694	2.22E-14
ABHD2	-0.37562	2.26E-14
ARID3A	0.891101	2.30E-14
PSMD1	-0.28065	2.51E-14
RCOR2	1.271937	2.53E-14
KDELC2	0.330505	2.71E-14
PLCL2	0.914246	2.72E-14
FBXO41	0.750825	2.83E-14
MRPL44	-0.35689	2.89E-14
VDR	1.021069	2.92E-14
FAM43B	1.171972	2.98E-14
DNAJA2	-0.33087	3.00E-14
FOXD2	2.57075	3.06E-14
PPL	0.665428	3.07E-14
HN1	-0.2634	3.11E-14
CBLL1	-0.3285	3.15E-14
DDAH1	0.44066	3.41E-14
ARL5B	-0.36977	3.48E-14
TGFA	0.698716	3.49E-14
C11orf57	-0.40216	3.54E-14
DHX16	-0.29493	3.55E-14
PNRC2	-0.32343	3.89E-14
SDR16C5	0.469708	3.89E-14
ITGB8	1.328516	3.91E-14
PHAX	-0.30046	4.03E-14
ZNF681	0.90762	4.26E-14
CDC7	0.398213	4.42E-14
CNOT4	-0.41411	4.53E-14
PCNT	-0.30824	4.64E-14
ZNF572	0.963501	4.69E-14
TGIF2	0.50559	4.72E-14
ENPP5	2.385477	4.73E-14
BAG4	-0.32912	4.74E-14
FBXW7	-0.74346	5.03E-14
FUS	-0.25179	5.09E-14

LSM1	-0.29523	5.39E-14
DYNLL1	-0.3182	5.41E-14
ZBTB17	-0.51387	5.46E-14
POLG	-0.28473	5.65E-14
SEMA7A	0.869232	5.67E-14
ZNF485	1.07853	5.76E-14
E4F1	-0.33397	6.22E-14
ARHGAP29	2.112817	6.31E-14
SFPQ	-0.2611	6.69E-14
RYR2	2.133642	7.00E-14
SFRP1	0.599951	7.12E-14
CDC42EP1	1.447415	7.61E-14
BNIP2	-0.33922	7.78E-14
PLAA	-0.34407	7.80E-14
AGPAT9	0.574317	7.99E-14
SQSTM1	-0.26306	7.99E-14
GFER	-0.36605	8.40E-14
KIAA1324L	0.33748	8.59E-14
TOR1AIP1	-0.30458	8.59E-14
SOX2	-0.28418	8.76E-14
NOL8	-0.37172	8.92E-14
FAM46C	0.402485	9.13E-14
HLTF	0.320346	9.39E-14
GOSR1	-0.28693	9.47E-14
FLCN	-0.70291	9.66E-14
SHISA9	0.281683	1.01E-13
HMGCS1	-0.3267	1.06E-13
MREG	0.667012	1.08E-13
C6orf120	0.379958	1.14E-13
FAM115A	0.363558	1.15E-13
SKIL	-0.36651	1.16E-13
GNAI2	-0.30327	1.22E-13
LAMC2	1.143373	1.23E-13
MAX	-0.37694	1.27E-13
IGSF3	0.418767	1.29E-13
GJA3	1.535734	1.35E-13
TM9SF4	-0.28922	1.36E-13
COLGALT2	0.507243	1.37E-13
KPNA6	-0.28776	1.40E-13
C7orf31	1.160922	1.44E-13
GNA11	-0.29547	1.46E-13
PHACTR1	1.509036	1.46E-13
MAGEA3	-0.3147	1.51E-13
ULBP1	0.601383	1.58E-13
EFNB2	0.439294	1.64E-13
BCL6	0.462171	1.71E-13
TOB2P1	1.316159	1.72E-13

ACSL4	0.499082	1.78E-13
CYP26B1	1.944926	1.83E-13
PCDH19	-0.28133	1.91E-13
PLEKHG1	0.374844	1.93E-13
SLC4A8	0.33419	1.93E-13
LOC10037‹	1.388642	2.01E-13
CTCF	-0.28886	2.09E-13
AP1S3	0.521368	2.12E-13
PNMA2	2.165285	2.15E-13
FAM43A	-0.26792	2.23E-13
ZNF622	-0.53886	2.24E-13
RAB11FIP5	1.160759	2.29E-13
HCFC2	-0.67654	2.34E-13
IREB2	-0.2818	2.42E-13
WNT16	1.921948	2.43E-13
QRICH1	-0.31171	2.49E-13
NOL9	-0.40649	2.58E-13
GPNMB	2.107377	2.60E-13
CDC37L1	-0.44423	2.62E-13
CLIP4	0.997007	2.71E-13
RBM42	-0.28859	2.74E-13
NUAK2	1.497214	2.85E-13
STON2	0.678965	2.90E-13
NUMA1	-0.26234	2.96E-13
IL1RAP	1.415599	2.97E-13
C10orf2	-0.44648	3.00E-13
ZNF217	0.307324	3.02E-13
NOP56	-0.26374	3.04E-13
RBM17	-0.27917	3.06E-13
UBAP1	-0.2906	3.17E-13
DNMT3B	0.566009	3.20E-13
ZNF343	0.62756	3.28E-13
WBP11	-0.28716	3.44E-13
DYRK3	1.146556	3.49E-13
SAR1A	-0.27898	3.54E-13
MFSD2A	0.301843	3.54E-13
STOM	1.00592	3.65E-13
SMARCD1	-0.29788	3.68E-13
MOB1A	-0.30198	3.72E-13
NFE2L2	-0.27886	3.73E-13
LOC100862	-0.65049	3.74E-13
DDX42	-0.29106	3.80E-13
FN1	0.414147	3.82E-13
RAB40C	-0.53835	3.89E-13
CEP350	-0.3809	3.90E-13
SRD5A3	0.423576	3.96E-13
PTGER4P2-	2.358769	4.10E-13

SYK	-0.27436	4.19E-13
STK11	-0.30056	4.23E-13
KLF15	1.120454	4.32E-13
NAPEPLD	1.115935	4.33E-13
DCP1A	-0.31424	4.46E-13
NKX3-1	0.749117	4.53E-13
DRAM1	0.478112	4.66E-13
LANCL2	-0.32148	4.70E-13
TSHZ1	0.789888	4.71E-13
SERTM1	2.269543	4.72E-13
PALB2	-0.54477	4.74E-13
STOX1	0.614245	4.74E-13
ABCF1	-0.29197	5.29E-13
GSC	1.894458	5.43E-13
PRRT3	0.845903	5.52E-13
MEX3D	-0.36834	5.60E-13
VAMP3	-0.30349	5.71E-13
LGALSL	0.469454	5.71E-13
ZNF18	0.857624	5.86E-13
GPR27	1.090894	6.02E-13
RLIM	-0.28067	6.18E-13
EIF3J	-0.26083	6.22E-13
WIPF3	2.160658	6.26E-13
PP7080	0.6432	6.32E-13
ZSCAN12P1	0.762057	6.75E-13
EMC8	-0.28112	6.75E-13
EPB41L4A-/	-0.39786	6.81E-13
MYEF2	0.29586	6.87E-13
IL12A	1.407078	6.98E-13
C19orf24	-0.47036	6.99E-13
STARD7	-0.29769	7.26E-13
ORC6	-0.28994	7.32E-13
PUM2	-0.28179	7.59E-13
KIFC1	-0.34512	7.67E-13
HNRNPDL	-0.26567	7.72E-13
C3orf33	0.842363	7.87E-13
TMEM11	-0.41513	7.87E-13
CTR9	-0.28973	8.22E-13
GBX2	0.783504	8.38E-13
CSGALNAC[illegible]	1.028693	8.60E-13
GID8	-0.34349	8.64E-13
RNASEH1	-0.33337	8.92E-13
CASC9	0.443212	9.30E-13
BCAR3	1.042403	9.31E-13
GFPT2	0.459275	9.37E-13
EIF4G1	-0.2311	9.41E-13
ARHGEF2	0.460465	9.52E-13

RRAGC	-0.33448	9.80E-13
HTR1D	1.613603	1.00E-12
POLQ	-0.41509	1.01E-12
CSTF1	-0.36746	1.04E-12
FAM217B	0.403463	1.09E-12
SYNJ1	-0.41122	1.11E-12
SMCR8	-0.35202	1.14E-12
ACBD5	0.413256	1.14E-12
GON4L	-0.3081	1.19E-12
SLC27A2	0.617162	1.20E-12
C16orf80	-0.26418	1.20E-12
GTF2F1	-0.27146	1.22E-12
AXIN1	-0.33977	1.24E-12
LOC10012	-0.51348	1.28E-12
WDR26	-0.31437	1.32E-12
MAST4	1.174785	1.33E-12
USP1	-0.30233	1.43E-12
HAUS2	-0.31929	1.45E-12
PHLDA2	1.223218	1.48E-12
BAZ1B	-0.25812	1.50E-12
TBC1D30	0.623529	1.52E-12
ZNF687	-0.29982	1.53E-12
NAA25	-0.28738	1.53E-12
EFCC1	0.39	1.58E-12
SRCAP	-0.34912	1.59E-12
MSL2	-0.29875	1.62E-12
GADD45A	0.514684	1.70E-12
TEX14	-0.77223	1.72E-12
SOX7	1.01849	1.78E-12
GALNT3	1.897469	1.78E-12
TTC8	0.405474	1.82E-12
PGAP1	0.439711	1.83E-12
LBH	0.311309	1.90E-12
ZFP90	0.511565	1.95E-12
CRTC2	-0.33008	1.99E-12
NOLC1	-0.24046	2.05E-12
CALR	-0.24019	2.06E-12
APOBEC3F	0.606607	2.06E-12
ZNF585A	0.684906	2.06E-12
TBC1D20	-0.477	2.08E-12
PNPLA2	-0.48818	2.08E-12
AZIN1	-0.28015	2.11E-12
TSKU	0.472531	2.22E-12
LRIG1	-0.36295	2.23E-12
GPATCH8	-0.30119	2.28E-12
DUSP14	0.343682	2.28E-12
ZNF266	-0.41448	2.32E-12

ANKLE2	-0.36599	2.33E-12
SETDB2	0.576455	2.34E-12
ETS2	0.493767	2.38E-12
SP140L	0.439708	2.44E-12
PPM1B	-0.30941	2.44E-12
ITPKB	0.708825	2.55E-12
PPM1K	0.690197	2.59E-12
ZZEF1	-0.36263	2.60E-12
ZC3H18	-0.28618	2.70E-12
YARS2	-0.38178	2.70E-12
PPIG	-0.27568	2.72E-12
LPP-AS2	1.533675	2.83E-12
LINC00847	0.83882	2.84E-12
MESTIT1	1.433107	2.88E-12
BAG5	-0.29313	2.88E-12
TBK1	-0.32934	2.93E-12
BYSL	-0.31559	2.93E-12
PCDHB14	0.90088	2.96E-12
SNX5	-0.30953	2.97E-12
C14orf132	0.920206	2.99E-12
CHD4	-0.23841	3.13E-12
CDKL2	0.938489	3.18E-12
GPR176	0.576565	3.20E-12
MYH7B	-0.40831	3.24E-12
ADAMTS20	1.780341	3.27E-12
ABHD15	0.709694	3.28E-12
PRKAB1	-0.34657	3.30E-12
TMC7	0.686225	3.31E-12
MRPL38	-0.34571	3.41E-12
BVES	1.57634	3.46E-12
SAFB	-0.31947	3.47E-12
MICU3	0.477802	3.51E-12
SGMS2	0.793971	3.51E-12
ATG13	-0.33862	3.53E-12
LRRC4	1.28059	3.57E-12
WBP1L	-0.46943	3.57E-12
ZNF654	-0.50755	3.66E-12
HSPA9	-0.24154	3.67E-12
KIAA0513	0.836651	3.74E-12
CDK16	-0.35628	3.77E-12
ZNF12	-0.33518	3.85E-12
PLEKHA8P1	1.76245	3.92E-12
MGA	0.49028	3.94E-12
C15orf38	1.354521	3.95E-12
FZD6	0.379298	4.11E-12
NUP153	-0.28375	4.12E-12
PLEKHM2	-0.27451	4.28E-12

SREBF2	-0.26364	4.32E-12
GMEB2	-0.34201	4.39E-12
WDR24	-0.44351	4.54E-12
NEDD9	0.309409	4.60E-12
BUD31	-0.29898	4.67E-12
GIGYF2	-0.27513	5.07E-12
LIN52	-0.43094	5.24E-12
NETO2	0.292267	5.34E-12
FAM212B	0.871104	5.42E-12
PSMC4	-0.26289	5.44E-12
GRPEL1	-0.33008	5.45E-12
WDR20	-0.38808	5.50E-12
HECTD1	-0.27024	5.62E-12
TMEM59L	1.920886	5.68E-12
DLC1	2.234746	5.70E-12
LAMTOR3	-0.46774	5.73E-12
ARF6	-0.31842	5.86E-12
FAM76B	-0.31551	5.98E-12
FAM107B	0.603219	6.04E-12
CDC14A	0.347085	6.04E-12
CLPX	-0.28539	6.22E-12
ATG2A	-0.45586	6.47E-12
ASB7	-0.46381	6.49E-12
MARK2	-0.32432	6.50E-12
AJUBA	0.957798	6.66E-12
SRSF10	-0.27291	6.68E-12
ADPRHL2	-0.44658	6.74E-12
DSEL	1.043743	6.76E-12
TSR1	-0.27644	6.92E-12
GTPBP4	-0.25776	7.01E-12
CLCN5	0.693251	7.18E-12
PSMD5-AS:	1.080368	7.52E-12
ZNF597	-0.60835	7.62E-12
B3GNT5	0.326188	7.67E-12
TOMM70A	-0.28804	7.69E-12
ANKRD11	-0.3481	7.83E-12
TLK2	-0.26801	7.93E-12
ARPC5L	-0.33485	8.04E-12
ISM2	1.84968	8.04E-12
ATAD5	-0.28983	8.04E-12
BIK	0.818484	8.06E-12
PI4KB	-0.27727	8.27E-12
RFXAP	0.557972	8.37E-12
JUN	-0.24115	8.37E-12
SEC61A1	-0.25012	8.41E-12
SLC3A2	-0.26042	8.44E-12
JAM3	0.611485	8.56E-12

DDX21	-0.25327	8.56E-12
NMT1	-0.24141	8.72E-12
SPTAN1	-0.25865	8.72E-12
CDKN2AIP	-0.31275	8.87E-12
COPS2	-0.24232	9.00E-12
SLC30A3	1.007941	9.05E-12
PRRG1	0.746226	9.48E-12
SLC9A2	0.497956	9.60E-12
CHAC2	0.452084	9.96E-12
HTATSF1P2	0.929492	9.98E-12
GDF6	2.120497	1.00E-11
USP22	-0.24269	1.01E-11
POU4F1	0.737291	1.01E-11
ATG16L1	-0.35333	1.01E-11
PIP4K2A	0.458285	1.02E-11
GOLGA2	-0.28797	1.05E-11
FUBP3	-0.28628	1.08E-11
ZNF174	0.443827	1.14E-11
ANGPTL4	1.246856	1.19E-11
FAM184A	0.361001	1.20E-11
ZSCAN32	-0.45499	1.22E-11
RNMT	-0.34979	1.26E-11
CPSF7	-0.32787	1.26E-11
ITGA4	1.404766	1.26E-11
SAP30BP	-0.36002	1.28E-11
TGFB2	-0.28357	1.30E-11
GDF15	0.692527	1.30E-11
ZBTB24	0.411495	1.32E-11
ITCH	-0.28871	1.33E-11
LRRC20	0.337228	1.37E-11
PSMD3	-0.26779	1.39E-11
NBR1	-0.31648	1.39E-11
TAF1D	-0.27401	1.40E-11
C16orf91	-0.43996	1.40E-11
CNOT2	-0.28138	1.42E-11
NOL6	-0.27479	1.46E-11
TIMM44	-0.28091	1.47E-11
GLCE	0.45595	1.48E-11
BCOR	0.369076	1.48E-11
TINCR	1.457501	1.48E-11
HES2	1.779232	1.49E-11
MNX1	1.125527	1.49E-11
PLEKHA5	0.42587	1.50E-11
PXDC1	0.632649	1.52E-11
GTF3C1	-0.23468	1.52E-11
ZCCHC8	-0.31234	1.54E-11
OSMR	1.197558	1.60E-11

RIC8B	0.37193	1.62E-11
ARMCX4	0.734623	1.63E-11
TMEM221	1.783724	1.64E-11
RNF168	-0.31806	1.64E-11
DGAT2	1.361053	1.71E-11
TBC1D10B	-0.37522	1.80E-11
EIF4E3	0.495197	1.80E-11
AP5Z1	-0.38129	1.83E-11
ZNF554	0.872957	1.84E-11
UBR2	-0.28093	1.84E-11
TMEM222	-0.32729	1.84E-11
PLK3	-0.3311	1.85E-11
GPR37	1.82874	1.88E-11
FZD7	0.915075	1.88E-11
DNAJA3	-0.2791	2.02E-11
PM20D2	0.32889	2.02E-11
RTF1	-0.27609	2.04E-11
ZNF3	-0.34057	2.09E-11
CCDC47	-0.26544	2.13E-11
MLLT11	0.323136	2.13E-11
EMR2	1.448702	2.14E-11
GET4	-0.30369	2.15E-11
HKR1	-0.30007	2.18E-11
PPAPDC2	0.792604	2.29E-11
CCNK	-0.32807	2.37E-11
FRMD4B	1.385018	2.43E-11
ELOVL7	0.539486	2.49E-11
FBXO45	-0.29439	2.49E-11
SART1	-0.29769	2.49E-11
MEIS1	0.999931	2.50E-11
TRIM28	-0.31227	2.55E-11
CDYL	0.293915	2.56E-11
DHX30	-0.30882	2.57E-11
MFSD5	-0.55231	2.61E-11
TMCO3	-0.34759	2.61E-11
PDCD7	-0.32396	2.62E-11
FAM220A	0.676292	2.66E-11
ZNF783	0.737043	2.75E-11
PSMC6	-0.27438	2.78E-11
SNCA	0.951747	2.84E-11
ACIN1	-0.23289	2.96E-11
KANK1	0.300634	2.97E-11
DAB2	1.820022	2.98E-11
TIGD2	1.213222	3.00E-11
B3GALT6	0.530998	3.00E-11
ARMCX5	0.400389	3.04E-11
DPF2	-0.28098	3.04E-11

HUWE1	-0.28276	3.18E-11
SURF6	-0.35228	3.24E-11
HOXA1	1.034507	3.30E-11
MAFG	-0.29326	3.31E-11
ARC	1.143307	3.36E-11
RABGGTB	-0.34444	3.42E-11
KLRG2	1.521691	3.43E-11
ZNF165	0.598868	3.48E-11
ZNF555	0.605659	3.48E-11
ZNF16	-0.56152	3.51E-11
RNMTL1	-0.38415	3.68E-11
TK2	0.866922	3.72E-11
CXXC5	0.821456	3.79E-11
KCNS3	0.57317	3.80E-11
C11orf82	-0.31572	3.86E-11
FAM175B	-0.33363	3.90E-11
ZBED5-AS1	1.136974	3.94E-11
PTPN13	0.453037	4.05E-11
DCLRE1A	0.349573	4.09E-11
BARX2	0.834543	4.10E-11
TFCP2L1	0.777169	4.11E-11
WNT5B	1.262107	4.11E-11
UQCRFS1	-0.26217	4.15E-11
TFAP2A	0.46979	4.15E-11
NCL	-0.23925	4.27E-11
RNF122	0.994909	4.31E-11
FEZF1-AS1	1.768523	4.33E-11
MAPK1IP1l	-0.263	4.37E-11
WNT5A	0.34146	4.44E-11
RAB5C	-0.26418	4.55E-11
FAM169A	0.4418	4.59E-11
ZNF547	-1.07973	4.59E-11
ZNF30	0.731202	4.70E-11
KIF2C	-0.264	4.74E-11
MED15	-0.39179	5.05E-11
MYSM1	-0.38755	5.09E-11
UNC5B	1.036677	5.42E-11
RANBP3	-0.24742	5.45E-11
PPP2R5B	0.631767	5.63E-11
FTSJ2	-0.29569	5.70E-11
NUP214	-0.29318	5.70E-11
KDM3B	-0.25219	5.95E-11
NSUN2	-0.2516	5.95E-11
CDK7	-0.34252	6.00E-11
TADA2B	-0.47107	6.00E-11
FAM124A	1.49265	6.07E-11
TLE4	0.780614	6.18E-11

DHX9	-0.23874	6.19E-11
CAND2	0.962125	6.40E-11
QSOX2	-0.27437	6.48E-11
SLC35E1	-0.26898	6.53E-11
POU3F1	1.340976	6.55E-11
ATXN7L3	-0.3211	6.58E-11
LINC00657	-0.27316	6.76E-11
STRN3	-0.27966	6.95E-11
TMEM98	1.857696	6.97E-11
TXLNG	-0.26871	7.19E-11
YOD1	-0.43116	7.22E-11
ZNF384	-0.27761	7.23E-11
ADAMTSL2	-0.32606	7.23E-11
CYP2S1	0.637217	7.32E-11
HPS5	-0.38713	7.41E-11
MRPS18B	-0.2553	7.52E-11
HSPA4L	0.29923	7.56E-11
IST1	-0.24047	7.56E-11
SENP5	-0.28278	7.58E-11
HIPK3	-0.34057	7.63E-11
MAP1S	-0.33673	7.67E-11
LIFR	0.297291	7.80E-11
PDHX	-0.27868	7.89E-11
MTF2	-0.39505	7.99E-11
CENPC	-0.34658	8.23E-11
GJA1	1.858384	8.24E-11
MTMR9	-0.31167	8.30E-11
CDC23	-0.2538	8.31E-11
SLC38A2	-0.34347	8.33E-11
GZF1	-0.38994	8.33E-11
KMT2A	-0.34853	8.34E-11
MAGEE1	0.908495	8.39E-11
ACTN2	1.522973	8.47E-11
CDK11B	-0.37259	8.47E-11
NUP98	-0.27859	8.55E-11
TAF7	0.322017	8.68E-11
IL20RA	1.24041	8.78E-11
CAMK2N2	0.729004	8.90E-11
MTHFD2P1	0.586502	9.28E-11
DNAJC13	-0.27653	9.58E-11
MRAS	1.602638	9.58E-11
BLOC1S4	0.895189	9.82E-11
SMAD4	-0.30626	9.97E-11
L1CAM	1.691096	1.00E-10
C15orf39	0.371448	1.01E-10
EHHADH	0.528661	1.03E-10
GJB2	1.724777	1.03E-10

KLF7	0.356181	1.03E-10
RHOBTB1	0.744185	1.05E-10
STX5	-0.27662	1.09E-10
DBX1	1.412903	1.10E-10
HAGLR	1.319101	1.12E-10
UTP15	-0.3859	1.17E-10
TMEM182	0.741081	1.19E-10
MYBL1	0.817487	1.20E-10
TRIM65	0.407898	1.20E-10
HSPA14	-0.30616	1.21E-10
TFRC	-0.23886	1.21E-10
METAP2	-0.23312	1.24E-10
MGC57346	0.597642	1.24E-10
TM2D3	-0.50501	1.25E-10
KLHL21	0.37102	1.25E-10
NRAP	0.440248	1.27E-10
DNAJC25	-0.47515	1.28E-10
RBM48	-0.54002	1.30E-10
MISP	0.323053	1.31E-10
MAGEA6	-0.29872	1.35E-10
DTX4	1.3105	1.41E-10
ILKAP	-0.32931	1.45E-10
GDNF	0.433587	1.48E-10
CEP95	-0.29664	1.49E-10
DHX8	-0.31069	1.51E-10
GPBP1	-0.30894	1.52E-10
ZSCAN26	0.619153	1.55E-10
SGCB	0.387354	1.56E-10
ZFHX3	0.416922	1.57E-10
LSG1	-0.22135	1.59E-10
KLHL42	0.298549	1.67E-10
RAB6B	0.664362	1.68E-10
DOLPP1	-0.29469	1.71E-10
TTPA	0.601003	1.72E-10
TMEM229A	1.723375	1.74E-10
KPNA4	-0.23566	1.76E-10
DGCR14	-0.38962	1.78E-10
C9orf114	-0.33317	1.82E-10
DPYSL2	0.272054	1.83E-10
FNDC1	1.534582	1.84E-10
DDA1	-0.27234	1.87E-10
DUS3L	-0.25424	1.87E-10
HSPA12A	0.679406	1.99E-10
DCUN1D3	-0.59942	2.02E-10
FAM53C	-0.24509	2.02E-10
HNRNPM	-0.23929	2.02E-10
SLC35A2	-0.40229	2.05E-10

HNRNPL	-0.23932	2.06E-10
GPBP1L1	-0.32863	2.08E-10
NUDT11	0.985937	2.09E-10
RNF19B	-0.4555	2.17E-10
SNHG12	-0.47198	2.21E-10
NEDD4	0.308159	2.23E-10
LOC64676	0.416955	2.24E-10
CHRM4	1.382039	2.24E-10
ZNF324	-0.77574	2.25E-10
RNF149	0.356778	2.27E-10
PRPF4	-0.36416	2.35E-10
CACHD1	0.37988	2.40E-10
FKBP14	0.427715	2.43E-10
ATF3	-0.24777	2.48E-10
SMG5	-0.2778	2.65E-10
SNHG15	-0.27655	2.66E-10
CDH3	1.459145	2.67E-10
PRPF6	-0.26009	2.70E-10
TRAFD1	-0.29826	2.73E-10
MAPKAPK	-0.35531	2.73E-10
DCTN5	-0.26995	2.73E-10
GPR1	1.935143	2.73E-10
ZFAS1	-0.30157	2.76E-10
ZNF302	0.349406	2.76E-10
DPP9	-0.2643	2.76E-10
EFNA3	1.424547	2.78E-10
SPSB1	0.59317	2.80E-10
SETD2	-0.29949	2.82E-10
ARHGEF37	1.171909	2.84E-10
ZBTB7B	-0.33019	2.86E-10
SOX9	-0.27423	2.90E-10
TMEM185	0.351478	2.91E-10
NOP2	-0.24021	3.00E-10
BBC3	-0.60801	3.06E-10
ATP8B1	-0.24145	3.14E-10
SRRM2	-0.32116	3.17E-10
ZFP36	-0.56925	3.17E-10
RAI2	1.500118	3.29E-10
TSSC4	-0.4241	3.41E-10
C1orf74	0.693549	3.42E-10
SAMD4A	1.409138	3.44E-10
IGFBP7	1.261195	3.48E-10
LPCAT2	0.607785	3.48E-10
NKX2-1	0.783405	3.54E-10
POLR3D	-0.28752	3.54E-10
RNF182	1.975742	3.56E-10
ZFAND2A	-0.60568	3.58E-10

PIGB	0.562729	3.67E-10
PRKAG2	0.959847	3.68E-10
DLST	-0.24463	3.68E-10
TBC1D17	-0.55187	3.71E-10
TLE3	-0.27796	3.83E-10
SLC35G1	1.011194	3.91E-10
KIAA2013	-0.28283	3.92E-10
PDLIM1	0.573716	3.96E-10
GTF2H1	-0.32376	3.97E-10
STX11	1.632177	4.02E-10
GNPDA2	0.564753	4.10E-10
C4orf19	0.86573	4.16E-10
TMEM132E	0.847774	4.20E-10
MORF4L1	-0.23296	4.28E-10
HMG20A	0.253205	4.28E-10
ATF2	-0.29519	4.29E-10
PISD	-0.33661	4.55E-10
FEZ1	0.704698	4.60E-10
SLC25A3	-0.21499	4.67E-10
COIL	-0.30472	4.73E-10
RC3H2	-0.34808	4.75E-10
PRKCQ	0.820944	5.21E-10
PRPF8	-0.24693	5.27E-10
HPS3	0.314374	5.29E-10
SORL1	0.341131	5.33E-10
RNF38	-0.32181	5.40E-10
TOX4	-0.25018	5.40E-10
OTUD5	-0.2847	5.40E-10
CDCA4	-0.54281	5.41E-10
LOC646903	1.155137	5.41E-10
TBC1D1	0.407037	5.42E-10
CENPL	-0.35753	5.47E-10
OSR2	0.862311	5.73E-10
MAPK6	-0.24862	5.83E-10
SRPR	-0.2597	5.86E-10
SNRNP40	-0.30413	5.86E-10
FAM89B	-0.3955	5.92E-10
TAOK1	-0.27545	5.93E-10
MUL1	-0.36757	5.94E-10
KCNK1	0.872338	6.10E-10
FXR2	-0.28591	6.15E-10
MAFA	0.856478	6.18E-10
PDPR	-0.31661	6.19E-10
INPP1	1.005862	6.21E-10
KIF20A	-0.29092	6.26E-10
QTRTD1	-0.29385	6.27E-10
ERMAP	-0.39377	6.32E-10

LRRC59	-0.22359	6.43E-10
NPAT	-0.3243	6.56E-10
SERINC1	-0.2565	6.57E-10
PEG10	0.274191	6.57E-10
CHD1	-0.2446	6.59E-10
CCNL2	-0.65267	6.59E-10
C12orf4	-0.33388	6.66E-10
RNF144A-A	0.747903	6.68E-10
PRR15	0.309141	6.84E-10
SUPT4H1	-0.23852	6.89E-10
USP19	-0.40772	6.90E-10
FAM102B	0.859252	6.96E-10
NABP1	0.454162	6.97E-10
MAU2	-0.33255	7.13E-10
C19orf43	-0.25511	7.17E-10
EMBP1	0.917293	7.23E-10
HMX1	1.050124	7.35E-10
SLC10A4	1.051483	7.36E-10
SNN	0.638469	7.36E-10
KDM3A	-0.25655	7.37E-10
ASXL3	1.363219	7.41E-10
CAMTA1	0.291531	7.51E-10
POMZP3	-0.55246	7.59E-10
KIF18B	-0.25629	7.63E-10
FAM84B	0.280687	7.78E-10
RBM4	-0.36286	8.16E-10
SDF2	-0.31409	8.20E-10
NUP160	-0.22474	8.20E-10
TNFRSF11A	1.525469	8.21E-10
TNFRSF1B	1.620227	8.30E-10
ACSL5	1.038268	8.38E-10
DPYSL3	-0.20565	8.46E-10
NUDT16L1	-0.32758	8.49E-10
AIM1	1.31447	8.57E-10
ZNF503-AS	0.544047	8.63E-10
NTRK2	1.514393	8.73E-10
HOXD1	1.446924	8.73E-10
SERTAD2	0.407245	8.90E-10
UBALD1	-0.32746	9.04E-10
VEZF1	-0.28797	9.16E-10
FOXA1	0.38245	9.64E-10
PLEKHA2	0.339209	9.88E-10
ZNF397	0.720552	9.88E-10
LYPD1	1.361763	1.01E-09
PPARGC1B	0.830712	1.03E-09
ARF1	-0.22054	1.04E-09
CXorf57	0.446976	1.05E-09

GSPT1	-0.20879	1.06E-09
LOC648987	1.167943	1.07E-09
PES1	-0.28012	1.08E-09
HNRNPU	-0.20226	1.11E-09
ADO	0.353854	1.13E-09
TAF5L	-0.30307	1.13E-09
MFSD6	0.562913	1.13E-09
HEG1	0.316277	1.13E-09
DNAJC18	0.42323	1.14E-09
NARF	-0.42061	1.16E-09
RAB3B	-0.28358	1.16E-09
GPR83	1.520255	1.17E-09
ZNF200	-0.37954	1.22E-09
ATXN1L	-0.29496	1.22E-09
CDS1	0.485009	1.23E-09
PTBP1	-0.2421	1.26E-09
LOC399815	0.937151	1.26E-09
ADORA2B	1.129763	1.30E-09
AK6	-0.36733	1.31E-09
STX3	0.454255	1.31E-09
TAF5	-0.47111	1.36E-09
SREBF1	-0.26047	1.39E-09
HILPDA	0.937885	1.39E-09
CABYR	0.298116	1.49E-09
BTBD7	-0.30429	1.50E-09
HMGXB3	-0.23658	1.50E-09
WDR55	-0.29012	1.50E-09
ZNF559	0.566449	1.52E-09
TAF2	-0.2495	1.54E-09
ZBTB41	0.401613	1.55E-09
CMTR2	0.4189	1.58E-09
KPNA1	-0.24233	1.59E-09
DLX3	0.439382	1.61E-09
PTPN21	0.929064	1.61E-09
GRWD1	-0.29139	1.63E-09
SLFN5	0.366475	1.63E-09
MME	0.421117	1.63E-09
ZFYVE26	-0.35671	1.66E-09
SCAMP1-AS	1.085896	1.70E-09
FAM171B	0.672595	1.73E-09
MED10	-0.30555	1.75E-09
TSPAN13	0.295744	1.80E-09
TAF13	-0.63798	1.81E-09
KCNJ11	0.932931	1.81E-09
PSMC3	-0.27029	1.85E-09
KIAA2026	-0.41033	1.85E-09
HNRNPH3	-0.21335	1.89E-09

ZNF410	-0.32414	1.90E-09
TBX3	-0.26814	1.90E-09
USP11	-0.38444	1.92E-09
THRAP3	-0.21912	2.02E-09
ATXN7L2	-0.70981	2.02E-09
IL6ST	0.593414	2.03E-09
ATP6V0D1	-0.28757	2.04E-09
USP15	-0.27478	2.04E-09
PIGA	-0.34212	2.05E-09
MMACHC	0.321455	2.06E-09
POLDIP2	-0.22382	2.06E-09
FBXO18	-0.25781	2.07E-09
SCG2	0.667831	2.07E-09
KLHL24	-0.37821	2.12E-09
ANKRD50	0.47703	2.17E-09
CYP2R1	1.123062	2.29E-09
BCHE	0.755145	2.29E-09
VLDLR	0.614584	2.29E-09
C11orf84	-0.26112	2.31E-09
SMU1	-0.24935	2.31E-09
ZNF84	0.458881	2.34E-09
MAP7D3	0.341366	2.37E-09
AQR	-0.24979	2.38E-09
DR1	-0.27162	2.41E-09
ZNF195	-0.34993	2.46E-09
PARVG	0.593941	2.57E-09
SESN3	0.336757	2.60E-09
SLC1A5	-0.23796	2.62E-09
RCE1	-0.37564	2.64E-09
ZNF709	0.929363	2.65E-09
EGR3	0.55494	2.68E-09
GAREM	0.610511	2.73E-09
TTC39C	0.441162	2.79E-09
SF1	-0.26553	2.84E-09
IFT46	0.383493	2.88E-09
CASC10	1.104784	2.94E-09
IKBKG	-0.33691	2.95E-09
ZNF79	-0.58209	2.98E-09
ZNF189	0.438758	3.00E-09
MAF	1.494552	3.03E-09
ZNF605	0.557544	3.04E-09
RNF111	-0.31437	3.04E-09
BEND4	0.836774	3.09E-09
CBR4	0.391718	3.11E-09
SUSD5	1.818633	3.13E-09
CLDN23	1.488961	3.15E-09
PTPDC1	0.559844	3.15E-09

CLGN	0.726877	3.20E-09
CBR1	0.320028	3.21E-09
CDC20	-0.21432	3.23E-09
TPPP	1.256254	3.34E-09
URB1	-0.2276	3.38E-09
SLC25A53	1.136038	3.38E-09
IL22RA1	0.515765	3.39E-09
GTSE1	-0.25913	3.39E-09
SCRN3	0.460163	3.41E-09
UFD1L	-0.26838	3.51E-09
UNKL	-0.36215	3.68E-09
LETM1	-0.26468	3.71E-09
LUZP1	0.359768	3.73E-09
SPG7	-0.35106	3.85E-09
MGC45800	0.963557	3.99E-09
PMM2	-0.24592	4.00E-09
HAUS8	-0.34009	4.02E-09
CTDSPL2	-0.23247	4.06E-09
KIF18A	-0.26082	4.09E-09
SGPP1	0.428644	4.14E-09
RAD21	-0.21715	4.17E-09
UBE4A	-0.29508	4.27E-09
MAP1A	0.909879	4.28E-09
NASP	-0.23702	4.31E-09
HOXC10	0.355923	4.52E-09
ERRFI1	0.478682	4.53E-09
EPAS1	0.339455	4.63E-09
ZNF180	0.529963	4.66E-09
FOXI3	1.73102	4.69E-09
DYRK2	0.498222	4.71E-09
FAM174B	1.052965	4.72E-09
NRF1	-0.30281	4.74E-09
PIM1	-0.50983	4.81E-09
FAM69A	0.342871	4.82E-09
SYF2	-0.33762	4.90E-09
HDHD3	0.608159	4.99E-09
ELOF1	-0.35667	5.08E-09
ZNF24	-0.31323	5.08E-09
LRRC37A6F	1.643117	5.08E-09
NCOA7	0.565487	5.19E-09
PAFAH1B1	-0.2475	5.20E-09
IGHMBP2	-0.33597	5.20E-09
BCL2L11	-0.39025	5.21E-09
RBMS2	1.221396	5.21E-09
RBM11	1.059836	5.21E-09
NELFA	-0.24816	5.23E-09
VPS4B	-0.27712	5.28E-09

EIF1AX	-0.20453	5.34E-09
RXRB	-0.27715	5.35E-09
SREK1	-0.39371	5.36E-09
HDX	0.599097	5.37E-09
TRAPPC6B	-0.30031	5.37E-09
EHD1	-0.36331	5.41E-09
RPL22L1	-0.49809	5.41E-09
IKZF5	-0.44384	5.48E-09
ATP6V1G1	-0.30474	5.51E-09
RBM34	-0.29067	5.51E-09
TNIP3	1.400874	5.56E-09
TMEM194E	0.747193	5.57E-09
RAE1	-0.24157	5.72E-09
NSFL1C	-0.24138	5.90E-09
FBXL3	-0.26432	5.91E-09
C8orf33	-0.2346	5.94E-09
LIN7C	-0.26503	5.96E-09
LOC93622	0.37579	5.97E-09
USP7	-0.20894	6.17E-09
KLHL11	-0.4598	6.25E-09
ZNF594	1.386089	6.36E-09
NIFK	-0.24394	6.37E-09
ZNF227	0.338584	6.37E-09
MTMR14	-0.27899	6.59E-09
SCNN1A	-0.20151	6.59E-09
MAP2K4	-0.28199	6.61E-09
SCAMP2	-0.296	6.62E-09
PGBD2	0.509947	6.71E-09
DSG2	0.240894	6.72E-09
RNF185	-0.34108	6.84E-09
DPP8	-0.26288	6.88E-09
ADNP	-0.22824	7.05E-09
ETV3	-0.49042	7.05E-09
ITGA2	0.590557	7.06E-09
NUFIP2	-0.28064	7.08E-09
AKAP11	0.358103	7.08E-09
GATAD2B	-0.28367	7.08E-09
TMEM127	-0.34039	7.08E-09
INPP5A	-0.37056	7.19E-09
MED4	-0.2939	7.27E-09
ZNF589	0.695809	7.34E-09
ETV7	1.403266	7.35E-09
DUSP19	1.142689	7.44E-09
ARFGEF2	-0.32555	7.50E-09
PCAT1	1.77149	7.64E-09
PKIB	1.175835	7.79E-09
HEIH	0.430126	7.80E-09

DAZAP2	-0.23008	7.81E-09
ACOX1	-0.30232	7.93E-09
ZNF490	-0.51269	7.94E-09
SUV39H1	-0.35511	8.06E-09
SAYSD1	0.819292	8.06E-09
USP9X	-0.27894	8.09E-09
C8orf88	0.375336	8.24E-09
MPV17L	0.434524	8.29E-09
MSANTD4	0.335796	8.43E-09
LINC00998	0.486685	8.44E-09
HJURP	-0.32626	8.45E-09
TVP23B	-0.52915	8.48E-09
TMEM189	-0.29055	8.73E-09
CCDC59	-0.23868	8.99E-09
LONRF3	0.828424	9.01E-09
UBE3B	-0.34042	9.16E-09
PDGFA	0.715027	9.18E-09
FLNA	-0.20161	9.18E-09
ZCCHC10	-0.30719	9.18E-09
ZNF114	1.292074	9.19E-09
RB1CC1	-0.25978	9.20E-09
MFI2-AS1	1.364455	9.40E-09
SPHK1	1.780012	9.40E-09
PDE12	-0.28278	9.49E-09
C6orf222	1.53177	9.49E-09
TRAM2-AS1	0.623176	9.68E-09
SLC6A14	0.643189	9.68E-09
BEST1	1.421401	9.72E-09
RICTOR	-0.31441	9.75E-09
METTL2A	-0.3196	9.75E-09
C7orf43	-0.49372	9.82E-09
ABT1	-0.35718	1.00E-08
DLG5-AS1	1.795875	1.02E-08
GCN1L1	-0.2204	1.02E-08
PDE3B	0.516172	1.05E-08
HOXD10	1.538139	1.08E-08
ENTHD2	-0.41371	1.10E-08
DNAJB4	-0.47112	1.10E-08
ZNF655	-0.23287	1.11E-08
ZCCHC18	1.40659	1.14E-08
CCDC112	0.299002	1.16E-08
MMD	-0.2548	1.16E-08
SAPCD2	0.353819	1.16E-08
TCEAL8	0.340832	1.17E-08
CROT	0.866712	1.17E-08
ALYREF	-0.23185	1.18E-08
ZNF436	0.475584	1.19E-08

TAF6L	-0.36884	1.19E-08
AMMECR1	0.292061	1.19E-08
GBF1	-0.29243	1.20E-08
LRFN3	1.604328	1.22E-08
B3GNT4	1.329297	1.23E-08
ZNF816	0.56313	1.24E-08
CCNB1	-0.24961	1.26E-08
RAF1	-0.23551	1.26E-08
SAMD5	1.232431	1.27E-08
JADE1	0.276704	1.27E-08
ZNF772	1.250937	1.28E-08
LRRC47	-0.25521	1.29E-08
KIAA1429	-0.24437	1.29E-08
TMEM62	0.316117	1.31E-08
DLL4	0.731541	1.32E-08
TUBB2B	-0.20871	1.32E-08
WIPI2	-0.3272	1.34E-08
FSD1L	0.626859	1.36E-08
MKL1	-0.31023	1.37E-08
GPATCH1	-0.41113	1.38E-08
IK	-0.20924	1.41E-08
BRD4	-0.30678	1.43E-08
SESN2	-0.3909	1.49E-08
SRRM3	1.573049	1.50E-08
CHMP1A	-0.27785	1.52E-08
HOXA3	0.668001	1.53E-08
CLDN12	0.370571	1.59E-08
KEAP1	-0.2678	1.60E-08
KLF16	-0.32872	1.61E-08
MIER3	-0.26768	1.61E-08
USP21	-0.34795	1.62E-08
NCKAP5L	-0.3463	1.64E-08
TMF1	-0.39351	1.66E-08
CLTC	-0.21511	1.66E-08
PLEKHG3	0.552956	1.66E-08
UBR4	-0.32462	1.66E-08
RAD23B	-0.20639	1.66E-08
EDRF1	-0.29585	1.66E-08
SEC31A	-0.2024	1.67E-08
TDG	-0.24098	1.69E-08
ZMAT2	-0.22635	1.71E-08
PLEKHF1	1.151846	1.71E-08
AMOTL2	0.283736	1.72E-08
CALM2	-0.22771	1.76E-08
RNF170	0.538545	1.76E-08
LPAR6	0.593843	1.81E-08
RBM10	-0.245	1.86E-08

STIL	-0.2429	1.89E-08
DDX46	-0.22305	1.91E-08
CIR1	-0.32177	1.91E-08
BMP8B	0.454845	1.91E-08
RAB3A	0.630586	1.92E-08
SLC16A9	1.652661	1.99E-08
PDE4A	1.544755	2.01E-08
LOC101927	0.495677	2.04E-08
C5orf22	-0.25479	2.05E-08
EDC3	-0.23336	2.05E-08
PRPF3	-0.24403	2.06E-08
GCC2	-0.29143	2.07E-08
HMGCR	-0.25869	2.08E-08
SP6	0.787993	2.11E-08
POLR2C	-0.24256	2.12E-08
RPUSD2	-0.36788	2.13E-08
TOR1AIP2	-0.25863	2.14E-08
EN2	0.873503	2.15E-08
EHD3	0.800944	2.16E-08
THEMIS2	1.420935	2.18E-08
EGR4	1.519771	2.19E-08
DNTTIP1	-0.29826	2.20E-08
THAP6	0.474334	2.22E-08
ATXN7L1	0.4939	2.24E-08
TRMT44	-0.51925	2.28E-08
SSU72	-0.24764	2.29E-08
MAP3K11	-0.35403	2.31E-08
MINA	0.424004	2.31E-08
NOL11	-0.24904	2.31E-08
C12orf76	0.65149	2.38E-08
BAG6	-0.20047	2.39E-08
ZFP30	0.490186	2.40E-08
RELA	-0.27475	2.40E-08
CYCS	-0.20409	2.42E-08
TMEM87A	-0.23223	2.45E-08
CHST15	0.344098	2.50E-08
PUM1	-0.24881	2.54E-08
MTFR2	-0.44633	2.58E-08
GCA	0.423477	2.60E-08
EMC3-AS1	0.344869	2.63E-08
CNBP	-0.18795	2.65E-08
WDR45B	-0.24529	2.66E-08
FAM32A	-0.23951	2.66E-08
TNFAIP8	0.68781	2.67E-08
FUT1	0.766535	2.69E-08
COPS3	-0.27496	2.70E-08
FBXL4	0.361541	2.70E-08

SUB1	-0.24477	2.74E-08
THYN1	0.252925	2.76E-08
TBPL1	0.282408	2.77E-08
U2AF2	-0.22797	2.82E-08
CDC42SE2	0.278458	2.82E-08
THBS1	-0.2298	2.84E-08
FOXO3B	0.680921	2.84E-08
PDCD11	-0.2357	2.85E-08
TESK1	-0.44592	2.86E-08
PRTG	0.4658	2.93E-08
EIF2S1	-0.229	2.93E-08
AKAP5	1.298503	2.97E-08
DRG1	-0.279	2.98E-08
E2F5	0.417017	2.98E-08
CITED4	0.612291	2.99E-08
AMDHD1	1.614875	3.05E-08
MAFK	-0.35432	3.08E-08
CNOT8	-0.22676	3.10E-08
RBMXL1	0.331914	3.11E-08
LINC00649	0.66095	3.12E-08
RNF103	-0.28487	3.18E-08
RNF166	-0.38635	3.24E-08
RHOV	0.790507	3.27E-08
ANKRD40	-0.23032	3.29E-08
KDM5A	-0.25468	3.30E-08
LINC00265	0.603066	3.30E-08
DBH-AS1	0.567776	3.32E-08
GOLIM4	-0.19832	3.35E-08
THOP1	-0.21416	3.39E-08
CRNKL1	-0.34554	3.44E-08
FOXO6	0.747013	3.44E-08
ZSWIM8	-0.33531	3.46E-08
PPP4C	-0.23916	3.48E-08
MAML1	-0.2356	3.52E-08
FAM198B	1.466475	3.52E-08
ATP6V0A1	0.300675	3.61E-08
RGCC	0.988436	3.66E-08
CHGB	0.20542	3.68E-08
USP10	-0.20674	3.68E-08
SMAD9	0.431865	3.70E-08
PRRC2C	-0.20984	3.70E-08
GALNT1	0.246659	3.72E-08
KRT8	-0.2348	3.77E-08
FUT4	0.402455	3.85E-08
AFG3L2	-0.24098	3.93E-08
TYSND1	0.288033	3.96E-08
KCMF1	-0.23691	3.97E-08

LRCH1	0.614553	3.98E-08
SLC1A4	0.310456	3.99E-08
FLJ20021	0.857535	4.04E-08
CHML	0.276629	4.05E-08
DTL	-0.23049	4.07E-08
SH3BGRL	0.605213	4.08E-08
GUCY1B3	1.709832	4.15E-08
SOCS3	1.646876	4.15E-08
USP16	-0.26931	4.18E-08
KAL1	1.384136	4.23E-08
RTN4RL2	0.993998	4.23E-08
JDP2	0.693508	4.27E-08
ATL2	-0.23652	4.28E-08
JARID2	0.448191	4.30E-08
APH1B	1.284384	4.44E-08
PPM1G	-0.2378	4.64E-08
SLK	0.275135	4.66E-08
ZNRF3	-0.25408	4.69E-08
EXOC3	-0.36986	4.69E-08
FZD3	0.42735	4.70E-08
ZNF581	-0.61159	4.77E-08
ZNF516	0.55559	4.81E-08
TMEM37	0.540751	4.91E-08
ADIPOR2	-0.20181	4.92E-08
NOL10	-0.25532	4.99E-08
PALM3	0.801824	5.01E-08
HOMER1	0.368604	5.08E-08
UBIAD1	-0.26458	5.09E-08
SEC23IP	-0.25271	5.17E-08
SESTD1	0.26601	5.18E-08
UBE2C	-0.24827	5.20E-08
GLS2	0.868798	5.23E-08
ZNF563	0.881199	5.37E-08
KDM6A	-0.5799	5.52E-08
YWHAH	-0.22003	5.64E-08
CHKA	-0.27379	5.64E-08
MAP1B	0.206343	5.68E-08
TRIM68	0.726237	5.76E-08
PITHD1	-0.22276	5.76E-08
BAG1	-0.28156	5.87E-08
SPPL3	-0.33269	5.87E-08
ORC1	-0.26336	5.89E-08
UNK	-0.2321	5.93E-08
SMURF2	-0.42676	5.98E-08
SHOC2	-0.23989	6.04E-08
MAP3K9	-0.31752	6.06E-08
FGFBP1	0.346328	6.12E-08

ZNF746	-0.35597	6.18E-08
C17orf100	1.457434	6.24E-08
FAM168B	-0.19544	6.37E-08
BHLHE22	1.487134	6.47E-08
ALDH1B1	0.309958	6.47E-08
FMN2	0.841236	6.61E-08
BMS1	-0.2096	6.65E-08
SF3B2	-0.18766	6.65E-08
PHF20L1	-0.24657	6.78E-08
GGNBP2	-0.24519	6.80E-08
ZNF845	-0.39663	6.82E-08
CTDP1	-0.3053	6.85E-08
PRB3	1.105467	6.88E-08
ZNF653	-0.36522	6.90E-08
NUFIP1	-0.26966	6.92E-08
PRPF38A	-0.22137	6.96E-08
TJAP1	-0.30689	7.10E-08
YWHAG	-0.19701	7.12E-08
QKI	0.247744	7.12E-08
DNAJC11	-0.21945	7.17E-08
DNAJB11	-0.19301	7.17E-08
RPP38	-0.31214	7.19E-08
ZNF284	1.526615	7.27E-08
DDX49	-0.35714	7.41E-08
HTR2C	0.666452	7.57E-08
FOXJ3	-0.19788	7.61E-08
ABHD17C	0.349785	7.62E-08
CUL3	-0.20969	7.63E-08
DDB1	-0.18465	7.68E-08
C6orf48	-0.22932	8.10E-08
DNAJB6	-0.22806	8.14E-08
STAU1	-0.24251	8.14E-08
SF3B3	-0.18818	8.16E-08
MARCKSL1	-0.26767	8.33E-08
PRPF40A	-0.20269	8.38E-08
FZD1	0.235795	8.42E-08
HELZ2	-0.3325	8.43E-08
HFE	0.688503	8.57E-08
NCOA6	-0.22423	8.60E-08
LOC653602	1.476605	8.60E-08
LINGO1	0.369664	8.72E-08
DPM1	-0.2559	8.77E-08
PIK3R4	-0.26041	8.77E-08
CDAN1	-0.25748	8.77E-08
ZFP3	0.729258	8.81E-08
ESPN	0.790909	8.83E-08
ZNF260	0.272812	8.88E-08

CKAP4	0.229823	8.89E-08
PSMC2	-0.23931	8.92E-08
9-Mar	0.596472	8.96E-08
ZNF707	-0.43841	8.98E-08
PLA2G7	1.266723	9.03E-08
CCNA2	-0.25662	9.12E-08
PDGFRA	1.395938	9.45E-08
LDLRAP1	0.523774	9.46E-08
ROBO1	0.392723	9.48E-08
AHDC1	0.551645	9.50E-08
ZSCAN16	0.523958	9.69E-08
CSDE1	-0.18644	9.96E-08
PRDM10	-0.43736	1.00E-07
GLIS2	0.485552	1.02E-07
LTN1	-0.28604	1.02E-07
GPR3	0.731429	1.02E-07
GLTPD1	-0.39655	1.03E-07
CGN	0.512166	1.03E-07
FAM86JP	1.339772	1.04E-07
ZBTB1	0.263891	1.06E-07
MYH14	0.272182	1.07E-07
SEMA3F	-0.23187	1.07E-07
YIPF5	-0.23854	1.10E-07
GADD45B	-0.29663	1.10E-07
ZMYND8	0.223217	1.11E-07
DNAJC16	-0.34997	1.12E-07
ZNF483	1.170089	1.14E-07
HS1BP3	0.423543	1.14E-07
ILDR2	0.763453	1.14E-07
RUNX2	0.337456	1.15E-07
FAM13B	-0.29391	1.16E-07
BEX5	0.927675	1.16E-07
LIFR-AS1	1.565711	1.17E-07
POLR3E	-0.2219	1.18E-07
ZHX2	0.44357	1.19E-07
MON1B	-0.25332	1.19E-07
H3F3B	-0.19905	1.21E-07
EPHA7	1.143538	1.22E-07
SYNCRIP	-0.18559	1.23E-07
SLC52A3	1.334257	1.23E-07
BECN1	-0.26194	1.25E-07
RIC8A	-0.26126	1.25E-07
SPTLC1	-0.27439	1.26E-07
COPRS	-0.22527	1.27E-07
PGM5P2	0.875281	1.29E-07
SENP6	-0.21005	1.29E-07
GPR126	0.4351	1.30E-07

SPSB3	-0.4072	1.34E-07
TXNL4B	-0.26347	1.34E-07
DZIP1L	0.887359	1.34E-07
RPUSD1	-0.33902	1.37E-07
NUP85	-0.24281	1.37E-07
PRDM15	-0.32465	1.42E-07
ARHGAP23	0.677842	1.42E-07
H2AFJ	0.360478	1.45E-07
EPB41L3	0.373622	1.45E-07
CCDC12	-0.37684	1.47E-07
SAP18	-0.26745	1.47E-07
RSL1D1	-0.23859	1.48E-07
DDX19A	-0.22078	1.50E-07
RAD51AP2	1.475557	1.55E-07
ADD3	0.286363	1.56E-07
CTBP1-AS2	0.699142	1.56E-07
TRAPPC8	-0.27986	1.57E-07
POLI	0.516347	1.60E-07
NOSIP	-0.29486	1.62E-07
HDGFRP3	0.23198	1.62E-07
SYAP1	-0.22595	1.63E-07
FEN1	-0.21578	1.63E-07
DUSP8	-0.50084	1.63E-07
ZNF264	-0.30032	1.63E-07
CPOX	0.402626	1.64E-07
EEF2	-0.17689	1.67E-07
SLC25A12	0.272415	1.69E-07
ARGLU1	-0.22029	1.70E-07
TWISTNB	-0.26192	1.71E-07
CTGF	0.769617	1.72E-07
RHOF	0.591005	1.74E-07
SBDSP1	-0.28123	1.75E-07
DHX37	-0.23645	1.77E-07
TM7SF3	0.276718	1.78E-07
SLC6A8	-0.26616	1.81E-07
PXYLP1	0.354854	1.84E-07
TMEM45A	1.196746	1.84E-07
TRIM29	1.153028	1.84E-07
SAMD1	0.254886	1.88E-07
OXR1	0.329759	1.88E-07
CEP104	-0.29743	1.90E-07
DPH7	-0.35165	1.92E-07
HECTD2	0.430528	1.92E-07
FAM174A	-0.46069	1.92E-07
CMPK2	0.666115	1.94E-07
NR1H2	-0.34101	1.95E-07
CXCL14	-0.19752	1.95E-07

SOX21-AS1	0.435741	1.95E-07
MAGEA4	-0.19535	2.00E-07
PSMD2	-0.19772	2.00E-07
PLEKHA8	0.395697	2.00E-07
ALKBH1	-0.32193	2.02E-07
CMYA5	1.280125	2.07E-07
ZNF473	0.36722	2.08E-07
SNAPC4	-0.33911	2.08E-07
PPP6R3	-0.21415	2.13E-07
PAXIP1-AS1	0.521297	2.16E-07
PTPN23	-0.32342	2.17E-07
ETNK1	-0.2755	2.17E-07
IWS1	-0.24468	2.17E-07
TBC1D8B	0.734747	2.17E-07
TSC22D2	-0.24825	2.19E-07
SSX2IP	0.290129	2.21E-07
HMGB2	-0.19402	2.22E-07
TEC	0.852948	2.23E-07
2-Mar	0.461965	2.23E-07
ARHGAP1	-0.27841	2.27E-07
UBE2L3	-0.21042	2.28E-07
ZFYVE20	-0.33109	2.29E-07
PVR	0.272646	2.32E-07
LYAR	-0.21571	2.33E-07
NADK	-0.22326	2.35E-07
TSEN15	0.280739	2.35E-07
HACE1	0.514477	2.38E-07
RAVER1	-0.30928	2.40E-07
STRN4	-0.24317	2.41E-07
SDAD1	-0.3044	2.41E-07
FAM149A	0.798543	2.41E-07
UBQLN1	-0.20671	2.45E-07
CDH2	0.210357	2.47E-07
MAP3K1	-0.33862	2.49E-07
CUL2	-0.23325	2.50E-07
POF1B	0.268266	2.50E-07
NECAP2	-0.22174	2.56E-07
SLC8B1	1.09374	2.56E-07
PAPD4	-0.26303	2.59E-07
SLC7A6OS	-0.27849	2.61E-07
PLEKHG5	0.702625	2.62E-07
RIOK2	-0.21987	2.62E-07
ELAVL3	1.317332	2.62E-07
ZNF48	0.325525	2.65E-07
SARNP	-0.2233	2.67E-07
SNHG7	-0.26716	2.70E-07
LOC389641	0.549653	2.71E-07

NFXL1	0.27537	2.75E-07
PLLP	1.352036	2.79E-07
MPZL1	0.200717	2.79E-07
RFX4	1.314348	2.79E-07
MIA3	-0.21008	2.81E-07
CASZ1	0.261019	2.85E-07
FAM126A	0.31583	2.87E-07
6-Mar	-0.25996	2.96E-07
ALX4	0.274556	2.98E-07
MBNL2	0.386893	2.99E-07
COMMD7	0.292947	3.01E-07
EPS8	0.282213	3.11E-07
SBNO2	-0.25114	3.12E-07
VPS4A	-0.21766	3.12E-07
ATAD3B	-0.3045	3.15E-07
FGFR1OP2	-0.34149	3.20E-07
PPP6C	-0.22715	3.23E-07
PRDM2	-0.20922	3.23E-07
EIF2B2	-0.2698	3.24E-07
RNF6	-0.25094	3.28E-07
ATP13A1	-0.29625	3.28E-07
CEND1	0.900613	3.28E-07
LOC102724	0.347609	3.32E-07
OXCT2	0.849376	3.33E-07
ZMYM3	0.242807	3.34E-07
FGFR1OP	-0.2565	3.39E-07
PTPRU	0.396575	3.43E-07
LINC01144	0.99648	3.47E-07
PCYOX1L	0.317308	3.52E-07
RUNX1	-0.19135	3.55E-07
DCTN4	-0.1981	3.58E-07
ASB8	-0.33205	3.59E-07
PPCS	0.240272	3.61E-07
LINC00641	-1.04334	3.67E-07
PGBD1	0.46758	3.69E-07
GPN2	-0.32861	3.71E-07
TMEM79	-0.47221	3.72E-07
TMTC3	0.259481	3.73E-07
ITGA5	1.012479	3.74E-07
ZNF646	0.285394	3.74E-07
TRMT1L	0.257744	3.82E-07
E2F4	-0.26758	3.82E-07
CPEB1	0.714708	3.83E-07
LIG4	0.391485	3.85E-07
HINFP	-0.35945	3.90E-07
EXOSC4	-0.29202	3.91E-07
RNF145	0.260401	3.96E-07

CDK13	-0.26003	3.96E-07
ZNF26	-0.39196	4.03E-07
ZNF619	0.64275	4.08E-07
BTN2A2	0.320887	4.09E-07
COX10	-0.34621	4.14E-07
PI4K2A	-0.2643	4.14E-07
YLPM1	-0.22472	4.15E-07
USF1	-0.26145	4.15E-07
CEP19	0.760089	4.16E-07
L2HGDH	0.30941	4.17E-07
FBXO16	0.605104	4.18E-07
ZBTB8A	0.43594	4.21E-07
KAZN	0.662212	4.23E-07
VANGL1	0.320221	4.25E-07
YIPF3	-0.23267	4.27E-07
EPB41L5	0.276084	4.33E-07
ZNF787	-0.27131	4.38E-07
C11orf71	0.727234	4.41E-07
NOD1	0.733047	4.41E-07
GNL2	-0.23233	4.42E-07
ZFP36L2	0.33017	4.48E-07
PCBP1	-0.21413	4.56E-07
SH3BP5	0.357517	4.57E-07
LOC11323(	0.401041	4.63E-07
DAXX	-0.22045	4.70E-07
HLA-E	0.271249	4.71E-07
CHST7	0.91923	4.73E-07
RAB1A	-0.24735	4.74E-07
ADIPOR1	-0.21666	4.75E-07
ZBTB9	-0.38757	4.77E-07
ZNF254	-0.57117	4.77E-07
LIN9	-0.27403	4.79E-07
C14orf169	0.490136	4.83E-07
CRLF3	0.245118	4.88E-07
RACGAP1	-0.19212	4.92E-07
C1RL	0.555057	4.93E-07
CWC15	-0.25526	4.97E-07
DCSTAMP	1.350804	5.08E-07
NCK1-AS1	0.603694	5.08E-07
NEAT1	1.461589	5.10E-07
GDAP1	0.240899	5.12E-07
NVL	-0.21766	5.13E-07
GTF2A1	-0.23471	5.15E-07
HLA-B	0.404115	5.25E-07
LDOC1L	0.201007	5.27E-07
ALKBH5	-0.2355	5.31E-07
SLC9A1	-0.30418	5.32E-07

WDR1	-0.20342	5.34E-07
SLC35E4	0.905545	5.35E-07
TNPO2	-0.19198	5.39E-07
ZNF160	-0.3512	5.39E-07
BCL10	-0.43475	5.41E-07
KLHL18	-0.33286	5.41E-07
CYYR1	0.338094	5.43E-07
FRRS1	0.934185	5.43E-07
SNAP47	-0.27823	5.44E-07
PPP1R37	-0.3324	5.45E-07
KHSRP	-0.21801	5.51E-07
TIGD6	0.545937	5.64E-07
HAS2-AS1	1.182159	5.77E-07
SMC1A	-0.20903	5.88E-07
MAGT1	-0.18931	5.90E-07
SS18L1	-0.30293	5.93E-07
SP3	-0.19656	5.93E-07
PMPCA	-0.23282	5.95E-07
CENPBD1	0.526968	6.02E-07
KAT7	-0.2027	6.05E-07
RBM8A	-0.19265	6.06E-07
TBC1D10A	-0.62411	6.08E-07
CHMP7	-0.21923	6.08E-07
GMPR	0.405179	6.09E-07
NAGPA	-0.41373	6.15E-07
MTRF1L	-0.22133	6.28E-07
RPF2	-0.2146	6.29E-07
ZMYND19	-0.28043	6.30E-07
TCF25	-0.21098	6.35E-07
NUAK1	0.295755	6.36E-07
ZNF347	1.108404	6.38E-07
C1orf109	-0.225	6.39E-07
COBL	0.249402	6.39E-07
GPCPD1	0.264318	6.41E-07
PAG1	0.697345	6.41E-07
FBXL18	-0.23972	6.48E-07
OGFRL1	0.393381	6.58E-07
DHRS2	1.415711	6.63E-07
EIF4H	-0.17407	6.68E-07
XRN1	-0.257	6.74E-07
CHORDC1	-0.21984	6.77E-07
EID3	0.482637	6.81E-07
DST	0.248702	6.82E-07
WWTR1	0.241694	6.84E-07
SIMC1	0.397588	6.91E-07
PLD1	1.073012	6.93E-07
PTPN3	0.521264	6.93E-07

YAP1	0.268105	6.97E-07
PLA2G4A	1.250364	7.01E-07
NAA30	-0.2033	7.01E-07
IQCH	0.255921	7.03E-07
PARP2	-0.25803	7.05E-07
C11orf30	-0.27648	7.05E-07
HSPA4	-0.18912	7.09E-07
NIPA1	0.225977	7.14E-07
KDM1B	0.321019	7.17E-07
CENPB	0.267213	7.20E-07
LRIG2	0.323976	7.22E-07
ATG4B	-0.21527	7.24E-07
LOC10192¹	1.003685	7.30E-07
FNIP2	0.66603	7.32E-07
CSRNP2	-0.21586	7.41E-07
MKRN2	-0.20737	7.42E-07
NLRX1	0.473853	7.60E-07
CAB39	-0.21831	7.60E-07
ZNF791	-0.28861	7.61E-07
FUBP1	-0.21982	7.61E-07
LRP5	-0.22271	7.65E-07
TBC1D15	-0.31609	7.65E-07
RAB14	-0.23159	7.66E-07
HIPK1	-0.25498	7.68E-07
SF3A1	-0.23565	7.69E-07
EIF2B1	-0.19832	7.70E-07
DZIP1	0.319697	7.81E-07
HIF1A	-0.1978	7.81E-07
RNPS1	-0.17825	7.90E-07
SLC5A3	0.545481	7.91E-07
COPS5	-0.21178	7.95E-07
PDE5A	0.215066	8.08E-07
ERMP1	0.275598	8.12E-07
NPTXR	0.436138	8.35E-07
CHODL	0.881885	8.36E-07
ZPR1	-0.29499	8.41E-07
RHOB	-0.21579	8.53E-07
BPGM	-0.23213	8.60E-07
ARNT2	0.273098	8.77E-07
SCML2	0.285782	8.80E-07
NFKBIL1	-0.42777	8.81E-07
CCRN4L	-0.36041	8.82E-07
HBS1L	-0.22567	8.98E-07
STX12	-0.30687	9.00E-07
CCDC86	-0.20007	9.01E-07
HTT	-0.24508	9.09E-07
SLCO5A1	1.447055	9.16E-07

C6orf223	0.327122	9.45E-07
SNHG17	-0.26647	9.47E-07
HS3ST3B1	0.640294	9.52E-07
CXorf40B	-0.3617	9.57E-07
FLNB	0.303053	9.58E-07
SKP1	-0.19599	9.73E-07
STRIP1	-0.25826	9.75E-07
EWSR1	-0.18	9.78E-07
HOXA6	1.477235	9.86E-07
LHX4	1.157082	1.01E-06
ADM	0.373259	1.01E-06
NDUFS3	-0.24515	1.01E-06
FAM134C	-0.24656	1.02E-06
DCLRE1B	0.37409	1.03E-06
POLE3	-0.22978	1.04E-06
UBE3A	-0.19665	1.04E-06
SPAG1	0.317259	1.04E-06
TMEM168	0.527593	1.05E-06
BFSP1	1.214473	1.05E-06
SUPT16H	-0.18668	1.08E-06
KIF13A	0.380118	1.08E-06
LEO1	-0.20318	1.09E-06
RECK	0.904454	1.10E-06
LACTB2	0.352211	1.10E-06
HBP1	-0.42397	1.12E-06
SF3A3	-0.19339	1.12E-06
PTPRO	0.215189	1.13E-06
PRKCQ-AS1	0.857757	1.14E-06
USP20	-0.33303	1.16E-06
UTP20	-0.23068	1.16E-06
PPP1R32	-0.71684	1.17E-06
ABHD6	0.551771	1.17E-06
ATP2A2	-0.17527	1.18E-06
PIP4K2B	-0.22443	1.18E-06
HPCAL4	0.885956	1.18E-06
IRF2BP2	-0.20205	1.18E-06
PSME3	-0.18189	1.18E-06
KRT19	-0.20638	1.19E-06
CCAT1	0.651911	1.20E-06
FCF1	-0.28086	1.22E-06
ADNP2	-0.25557	1.22E-06
ENDOD1	0.551462	1.24E-06
SBF1	-0.21727	1.25E-06
SOX2-OT	0.420603	1.25E-06
GSTM3	0.457475	1.26E-06
ARHGAP5-/	1.26955	1.26E-06
TTC9C	-0.30983	1.27E-06

SPRED2	-0.24396	1.29E-06
C2orf88	1.343298	1.31E-06
ARID5A	-0.51613	1.31E-06
NEURL4	-0.33062	1.31E-06
ZBTB6	-0.36569	1.31E-06
SGMS1	0.464441	1.31E-06
GEMIN8P4	0.803443	1.32E-06
ZMPSTE24	-0.18036	1.33E-06
DCAF7	-0.19742	1.34E-06
RNFT1	0.489952	1.36E-06
OTUD6B-AS	0.241138	1.36E-06
SCOC	0.360454	1.37E-06
NRDE2	-0.30138	1.37E-06
RAB23	0.26308	1.37E-06
DEGS2	1.177133	1.39E-06
TMEM41B	-0.29136	1.39E-06
AKT3	0.484638	1.41E-06
UBTF	-0.23707	1.42E-06
IGIP	1.105499	1.43E-06
LOC101928	0.759555	1.43E-06
GOLPH3L	-0.28642	1.44E-06
ENTPD8	0.59511	1.45E-06
PLEKHO1	0.471728	1.45E-06
FLJ37453	0.729035	1.47E-06
RUFY1	-0.19515	1.49E-06
KIF11	-0.19031	1.49E-06
DLX6	0.328529	1.50E-06
NANS	-0.20496	1.50E-06
DUSP26	1.311282	1.50E-06
PANK4	-0.26666	1.51E-06
ADAM17	-0.28429	1.52E-06
RAET1K	0.872686	1.53E-06
CHST6	1.091547	1.54E-06
ARHGAP18	0.399208	1.54E-06
CPA2	0.355227	1.54E-06
RBM27	-0.21815	1.55E-06
COPA	-0.17134	1.56E-06
ENAH	0.194089	1.56E-06
MRFAP1	-0.16947	1.59E-06
REG1A	0.236458	1.61E-06
PDLIM3	0.248402	1.61E-06
POLR2B	-0.21593	1.62E-06
SWAP70	0.345407	1.64E-06
TUBGCP5	0.218185	1.66E-06
ZNF362	0.406761	1.67E-06
KDM4B	-0.23592	1.67E-06
NFKBIZ	-0.59472	1.69E-06

RNF2	0.273281	1.70E-06
LOC100507	1.054745	1.70E-06
GAS2L1	0.650983	1.71E-06
ZNF45	0.334655	1.71E-06
ANKFY1	-0.21489	1.71E-06
MOB1B	0.351471	1.71E-06
B4GALT1	0.36512	1.72E-06
PSMB2	-0.21439	1.73E-06
SETX	-0.23798	1.75E-06
LTV1	-0.21277	1.75E-06
ADAMTS18	0.223748	1.75E-06
IER3	0.449102	1.75E-06
IFIH1	0.450468	1.75E-06
VDAC2	-0.23189	1.76E-06
DDX55	-0.22883	1.78E-06
SLC26A2	0.547529	1.80E-06
FGFBP3	0.79819	1.81E-06
CCDC157	-0.60387	1.81E-06
RBM23	-0.19935	1.82E-06
MSANTD2	-0.40943	1.86E-06
PAIP2	-0.19311	1.86E-06
TRIP12	-0.2353	1.86E-06
TTC17	-0.23996	1.88E-06
TIMM17A	-0.18999	1.89E-06
SDHAP2	-0.68926	1.90E-06
RNF216P1	0.363925	1.91E-06
ZNF142	-0.25647	1.92E-06
SEMA4F	0.439181	1.92E-06
C18orf54	0.466971	1.95E-06
CELSR3	0.463229	1.96E-06
ELOVL1	-0.20716	1.97E-06
PCGF1	-0.31708	2.00E-06
PPP4R1	0.269449	2.03E-06
PAQR3	0.262472	2.03E-06
PEX14	-0.24789	2.03E-06
ADAMTS12	0.416016	2.04E-06
ZNF699	0.728276	2.04E-06
C2orf47	-0.2948	2.08E-06
PMS1	0.241811	2.08E-06
ASAP2	0.267754	2.08E-06
TUBB	-0.16263	2.08E-06
KCNE4	0.353652	2.09E-06
DMXL2	0.223441	2.10E-06
RBMX	-0.17875	2.11E-06
PPP6R2	-0.268	2.11E-06
ZBTB48	-0.28877	2.15E-06
NIPBL	-0.20731	2.17E-06

RNF11	-0.25106	2.18E-06
MCF2L-AS1	0.890514	2.18E-06
ATP2B4	0.497843	2.21E-06
TRIM4	-0.28919	2.22E-06
CUL5	-0.2268	2.23E-06
HYOU1	-0.17355	2.23E-06
REV3L	-0.21055	2.26E-06
STIP1	-0.1731	2.27E-06
TPX2	-0.17622	2.27E-06
SNRNP70	-0.2457	2.27E-06
PAQR8	0.45063	2.29E-06
RNF138	-0.24456	2.33E-06
BACH1	-0.33905	2.33E-06
ARFGAP1	-0.24639	2.34E-06
DIS3	-0.19262	2.35E-06
C1orf174	-0.41169	2.39E-06
ACBD4	0.428217	2.40E-06
UPF2	-0.21734	2.42E-06
BCKDHA	-0.24453	2.43E-06
TMEM212	0.695233	2.43E-06
MSL1	-0.23768	2.45E-06
MRTO4	-0.19746	2.46E-06
MADD	-0.18679	2.46E-06
SOX8	0.438257	2.46E-06
C1orf27	-0.24103	2.47E-06
GLI3	1.306462	2.48E-06
CTBS	0.63232	2.49E-06
OGDH	-0.18866	2.49E-06
SNRK	-0.25051	2.51E-06
PDCD6	-0.23428	2.53E-06
ZNF484	0.525297	2.54E-06
ARL6IP5	0.434137	2.55E-06
MAP2K3	-0.2638	2.56E-06
SPAG5	-0.17716	2.57E-06
ASNA1	-0.23112	2.58E-06
MLF1	0.433909	2.59E-06
RBBP8	0.229816	2.60E-06
ENTPD1	0.378316	2.60E-06
ONECUT2	1.107591	2.63E-06
ZNF540	1.694898	2.66E-06
NUP133	-0.21994	2.70E-06
RAB21	-0.24197	2.71E-06
PNPO	0.256823	2.71E-06
PRRC2B	-0.20941	2.74E-06
SEC24C	-0.2151	2.74E-06
MAP3K7	-0.20723	2.77E-06
CREBRF	-0.65589	2.77E-06

CDK17	0.27994	2.77E-06
C7orf26	-0.24809	2.79E-06
COPB2	-0.19772	2.82E-06
TLR3	1.146377	2.84E-06
TMED2	-0.17893	2.87E-06
CHD5	0.585777	2.88E-06
TUBGCP2	-0.23844	2.90E-06
BAZ2A	-0.23035	2.92E-06
TBRG4	-0.23282	2.93E-06
LRPPRC	-0.17825	2.94E-06
SQRDL	0.531437	2.95E-06
ZNF292	-0.23538	2.96E-06
USP25	0.310027	2.96E-06
CCAR2	-0.20186	2.97E-06
RAB8B	0.565343	2.98E-06
BLOC1S3	-0.6306	2.98E-06
GDPGP1	1.190629	2.99E-06
BAP1	-0.21944	3.01E-06
MTMR3	-0.30261	3.01E-06
ESRRG	0.843855	3.03E-06
HOXD11	0.96279	3.03E-06
MCM7	-0.17726	3.05E-06
GLUL	-0.17037	3.09E-06
SASS6	-0.26755	3.09E-06
SMYD4	0.316451	3.10E-06
CDKN1C	0.865519	3.12E-06
TEX15	0.507189	3.21E-06
HNRNPUL1	-0.16899	3.23E-06
CABLES1	0.575378	3.27E-06
NAB1	0.323946	3.27E-06
ZER1	-0.28523	3.28E-06
CEP76	-0.39839	3.29E-06
SERPINB1	0.299953	3.30E-06
NPL	1.205015	3.33E-06
ZNF391	0.679254	3.34E-06
C1orf50	-0.27761	3.35E-06
FOSB	-0.76774	3.36E-06
ZNF318	0.223296	3.38E-06
KIF5C	0.215518	3.40E-06
SLC17A5	0.399002	3.48E-06
BIN3	-0.30545	3.49E-06
SH3PXD2B	0.207358	3.55E-06
NSL1	0.243358	3.56E-06
ZNF395	0.415629	3.57E-06
BCL7B	-0.2104	3.57E-06
WSB1	-0.22153	3.57E-06
NCKIPSD	0.324568	3.60E-06

ARAP2	1.097043	3.70E-06
ZNF717	0.468046	3.70E-06
PRPF39	-0.25079	3.73E-06
MCM3AP-A	1.0225	3.74E-06
ARCN1	-0.17227	3.75E-06
TUT1	-0.26573	3.78E-06
MEIS3	1.149094	3.79E-06
MOBP	0.466586	3.80E-06
CSNK2A1	-0.18077	3.83E-06
TOM1L1	0.205597	3.88E-06
KATNAL1	0.50905	3.89E-06
ZFP91	-0.18281	3.90E-06
ZNF510	0.411091	3.94E-06
CEBPZ	-0.22849	3.99E-06
CHAF1A	-0.1779	3.99E-06
GOLT1B	-0.25229	4.02E-06
HPSE	0.988536	4.06E-06
WBP5	0.26053	4.08E-06
MASTL	-0.25634	4.11E-06
ZNF805	-0.43515	4.13E-06
RDH10	-0.34801	4.19E-06
MPI	0.268901	4.24E-06
PTPN1	-0.23578	4.24E-06
FAS	1.29781	4.26E-06
ATF7IP2	0.575162	4.27E-06
HAUS3	-0.28938	4.27E-06
BAG2	0.195442	4.29E-06
CEP97	0.310964	4.29E-06
MAGI1	0.346336	4.32E-06
GLTSCR2	-0.19093	4.32E-06
BET1L	-0.21149	4.33E-06
AP1B1	-0.21909	4.33E-06
ZNF383	-0.43774	4.38E-06
CTLA4	0.839404	4.40E-06
FBXO43	0.999674	4.42E-06
PSMC1	-0.18154	4.44E-06
KANSL1	-0.19443	4.44E-06
LINC01206	1.301123	4.46E-06
APAF1	0.310045	4.46E-06
ZNFX1	-0.46683	4.46E-06
DLG3	0.248747	4.48E-06
SEC14L5	1.094775	4.53E-06
EPT1	-0.22852	4.55E-06
KLHL5	0.421411	4.61E-06
BIRC6	-0.21562	4.65E-06
RAD50	0.204123	4.65E-06
RER1	-0.21432	4.70E-06

H2AFY2	0.264297	4.72E-06
TNPO3	-0.18952	4.74E-06
SURF4	-0.19238	4.80E-06
PNO1	-0.2018	4.94E-06
ANXA4	0.293888	5.03E-06
ST7-AS1	1.005251	5.05E-06
EXOSC10	-0.17947	5.08E-06
TMEM45B	0.190692	5.09E-06
WDR53	-0.29159	5.10E-06
IMPDH2	-0.19498	5.12E-06
CKAP2L	-0.21409	5.12E-06
TBCEL	-0.31577	5.14E-06
SLC25A33	-0.25605	5.19E-06
PLK4	-0.21691	5.26E-06
FAM73B	-0.48275	5.26E-06
ZNF74	0.484986	5.31E-06
SERPINE2	0.665237	5.33E-06
NR4A2	0.880853	5.37E-06
TAF3	-0.20964	5.37E-06
CSGALNAC	0.289294	5.41E-06
AFAP1L2	0.246047	5.43E-06
MAST3	0.555444	5.49E-06
LPPR5	1.082659	5.51E-06
PDIA3P1	0.802345	5.57E-06
ORAOV1	-0.26161	5.64E-06
LOC101927	0.952014	5.68E-06
TCP1	-0.20639	5.83E-06
L3HYPDH	0.50075	5.85E-06
GPATCH2	-0.24105	5.90E-06
KDF1	0.704928	5.92E-06
NKD1	-0.18103	5.95E-06
TNFAIP8L3	0.232621	6.06E-06
FARP2	-0.3641	6.07E-06
CROCCP2	-0.34514	6.10E-06
FAM117A	-0.31133	6.11E-06
RAD21-AS1	0.47928	6.11E-06
SYNGR3	0.414731	6.14E-06
DNAJC9	-0.17839	6.21E-06
PWWP2A	-0.21017	6.23E-06
KBTBD3	0.808506	6.23E-06
ZNF780B	0.634838	6.26E-06
NBPF3	0.688939	6.34E-06
ACTB	-0.24364	6.35E-06
NBN	0.201562	6.40E-06
PPP1R8	-0.20475	6.44E-06
SLC7A5	-0.1979	6.44E-06
YEATS2	0.181214	6.46E-06

LONRF2	0.390037	6.60E-06
ULK1	-0.33069	6.61E-06
MED7	0.429537	6.69E-06
FXR1	-0.17426	6.73E-06
KCTD15	0.287076	6.73E-06
C7orf25	0.477531	6.75E-06
PHF1	-0.32866	6.81E-06
ING2	-0.42723	6.83E-06
CEP68	0.271555	6.85E-06
IGFBP2	0.255582	6.95E-06
YKT6	-0.19021	6.96E-06
TNKS2	-0.19553	6.96E-06
EIF3J-AS1	0.391904	6.99E-06
ERCC6L2	-0.22091	7.01E-06
TMEM60	0.604006	7.01E-06
SHC1	-0.23433	7.04E-06
ATL3	0.401715	7.05E-06
ALDH1A1	0.246806	7.06E-06
MOGS	-0.2053	7.09E-06
PARP9	0.484419	7.12E-06
SNX16	0.413238	7.24E-06
SHPRH	0.243713	7.28E-06
LETM2	0.405519	7.30E-06
PIGP	0.332212	7.39E-06
ADAP2	1.224342	7.43E-06
BTBD3	0.376661	7.45E-06
POLR2D	-0.18129	7.47E-06
FBXL16	0.683162	7.49E-06
MIS12	-0.20923	7.62E-06
RINT1	-0.22876	7.63E-06
ACAA2	-0.16427	7.64E-06
FERMT2	0.223212	7.67E-06
MYH9	-0.16364	7.68E-06
FIZ1	-0.36627	7.70E-06
ZC3H15	-0.17062	7.71E-06
DHRS4-AS1	0.339513	7.73E-06
TRAM2	0.210137	7.75E-06
GPRC5B	0.198062	7.77E-06
MIOS	0.315733	7.81E-06
SLC7A8	0.30619	7.84E-06
TGS1	-0.19239	7.88E-06
RARS	-0.17892	7.90E-06
ZNF280B	0.744723	7.92E-06
IGF2R	-0.21434	7.94E-06
ZNF222	0.436691	7.94E-06
TMEM65	0.464612	7.95E-06
DOT1L	-0.26886	7.96E-06

CDH8	0.571086	7.99E-06
NUP88	-0.20073	8.08E-06
ELAVL1	-0.17553	8.09E-06
WLS	0.345596	8.11E-06
SYDE2	1.07103	8.13E-06
AKR1E2	0.4271	8.17E-06
M6PR	-0.19055	8.23E-06
CAPZA1	-0.18936	8.24E-06
H6PD	0.372639	8.28E-06
NR1D2	-0.24178	8.34E-06
PNPLA5	0.45702	8.34E-06
KHDRBS1	-0.17162	8.38E-06
ACOT11	0.860516	8.43E-06
BNIP1	-0.28756	8.46E-06
CCDC14	0.223626	8.47E-06
AGA	0.421151	8.52E-06
ACACB	0.342133	8.57E-06
RNF19A	-0.30764	8.62E-06
CCNG1	-0.192	8.62E-06
ADRBK2	0.335505	8.68E-06
SMAD6	0.33065	8.71E-06
AP4B1	0.389095	8.74E-06
AR	0.355138	8.76E-06
BBS7	0.304913	8.79E-06
RNPEP	0.192517	8.81E-06
TMTC4	0.34814	8.81E-06
SGPP2	0.994826	8.85E-06
RBPJ	-0.19701	8.89E-06
CHD8	-0.19667	8.98E-06
NR4A1	-0.2991	9.04E-06
SRA1	-0.19463	9.06E-06
IRAK1BP1	0.58488	9.15E-06
ARHGEF7	-0.21803	9.40E-06
USP14	-0.20577	9.41E-06
LOC100131	0.544967	9.42E-06
PGM2	0.209052	9.44E-06
ISCU	-0.20942	9.58E-06
KDELC1	0.388788	9.62E-06
PAPD7	-0.22053	9.66E-06
MRM1	0.258529	9.68E-06
EIF6	-0.20287	9.68E-06
CEP290	0.244239	9.68E-06
FAM111A	0.452394	9.75E-06
PIAS1	-0.23653	9.77E-06
KCTD6	0.535145	1.00E-05
LINC00525	0.540139	1.00E-05
ACTRT3	0.728218	1.01E-05

STARD5	0.704865	1.01E-05
HSP90AB1	-0.15551	1.01E-05
RAC1	-0.15703	1.01E-05
UBE2S	-0.18379	1.02E-05
TRIAP1	-0.24914	1.02E-05
DDX47	-0.20317	1.02E-05
ARIH2	-0.22919	1.03E-05
DIXDC1	0.467947	1.03E-05
RAB11FIP1	-0.16691	1.03E-05
SIRPA	0.478166	1.03E-05
LOC101927	0.990451	1.04E-05
MLLT10	0.238451	1.05E-05
ARHGEF18	-0.22342	1.05E-05
UBE2D3	-0.16666	1.05E-05
PITPNB	-0.17947	1.05E-05
PDK3	0.54517	1.05E-05
CREB1	-0.22677	1.05E-05
MMGT1	-0.20578	1.06E-05
TARBP2	-0.28179	1.06E-05
BSDC1	-0.19351	1.06E-05
NFKBIB	-0.20367	1.07E-05
LAMP1	-0.17475	1.07E-05
MTOR	-0.18312	1.07E-05
XK	1.175253	1.07E-05
RPUSD4	-0.23306	1.09E-05
ZNF720	-0.27896	1.09E-05
KBTBD2	-0.20441	1.09E-05
POP7	-0.2148	1.09E-05
LINC00963	0.748307	1.10E-05
PHF5A	-0.20416	1.10E-05
PIGC	0.356161	1.11E-05
SEL1L	-0.24023	1.11E-05
TLR2	1.041559	1.13E-05
GGA3	-0.26226	1.13E-05
TRMT10C	-0.23935	1.13E-05
ATP6V0C	-0.31718	1.13E-05
ECD	-0.21156	1.13E-05
FNBP1L	0.218414	1.14E-05
SMC4	-0.15798	1.14E-05
IL27RA	0.36453	1.14E-05
CPNE8	0.272893	1.15E-05
GPR160	0.641446	1.15E-05
PANK3	0.186657	1.16E-05
WDR44	0.488288	1.16E-05
UTP3	-0.2221	1.18E-05
CASP4	0.64961	1.18E-05
DCLRE1C	-0.30377	1.18E-05

SLC9B2	0.464697	1.18E-05
SSBP2	0.790414	1.18E-05
SLC4A1AP	-0.21239	1.19E-05
PAK6	0.622879	1.19E-05
YWHAZ	-0.15239	1.21E-05
IL17RD	0.417183	1.21E-05
PSMD14	-0.20319	1.22E-05
CREG1	0.245783	1.24E-05
GAPVD1	-0.20841	1.24E-05
SMG1	-0.22524	1.24E-05
LMX1B	1.123509	1.25E-05
ACTG1	-0.22261	1.25E-05
NDNL2	0.240529	1.25E-05
OVGP1	-0.54398	1.27E-05
FAM58A	-0.24507	1.27E-05
C2orf44	0.265216	1.27E-05
RFX1	-0.40975	1.29E-05
RPS6KA6	0.598117	1.30E-05
FARSA	-0.177	1.30E-05
DCAF15	-0.21119	1.31E-05
DDX18	-0.18565	1.33E-05
THAP4	-0.21825	1.34E-05
DSE	0.727949	1.34E-05
TMEM177	0.476794	1.35E-05
ZNF32	0.493635	1.37E-05
GOLGA5	-0.25283	1.37E-05
TMEM248	-0.21331	1.39E-05
ANKRD17	-0.19257	1.39E-05
SLC30A5	-0.20414	1.40E-05
DDX51	-0.25492	1.40E-05
TNFRSF21	0.274877	1.40E-05
MIR4697H(	1.300428	1.41E-05
TCF15	0.853809	1.42E-05
BLCAP	-0.22873	1.43E-05
ZNF33B	0.358012	1.45E-05
MBTD1	-0.25764	1.45E-05
SMPD4	-0.17073	1.45E-05
LHX4-AS1	0.753166	1.45E-05
PWWP2B	0.723762	1.45E-05
PUS7L	0.317027	1.46E-05
RAB7A	-0.15457	1.46E-05
PTRH2	-0.22807	1.47E-05
TFAM	-0.16842	1.47E-05
SYT6	0.806141	1.49E-05
FAM214A	-0.33413	1.50E-05
DIABLO	-0.19295	1.52E-05
MRPL15	-0.18695	1.53E-05

LOC344887	0.301225	1.54E-05
RRP15	-0.18681	1.56E-05
CXorf56	-0.24924	1.57E-05
OGDHL	0.482574	1.58E-05
IL4R	0.876576	1.58E-05
RAD54B	0.250027	1.59E-05
OPRK1	0.832528	1.59E-05
PATL1	-0.18764	1.59E-05
CAPN15	-0.31499	1.60E-05
SPR	0.360053	1.61E-05
KLHL14	1.200601	1.62E-05
ZNF354C	0.349937	1.63E-05
NUP107	-0.21071	1.65E-05
ZNF25	0.449875	1.69E-05
KIAA1468	-0.20866	1.69E-05
CCDC126	0.682645	1.69E-05
KIDINS220	-0.18223	1.72E-05
DNAJC8	-0.21564	1.72E-05
PEX16	-0.28543	1.74E-05
KIAA2022	0.484462	1.75E-05
CRY1	-0.28135	1.77E-05
C12orf57	-0.21759	1.77E-05
N4BP1	-0.18104	1.81E-05
TES	0.303861	1.81E-05
CLPTM1	-0.20524	1.81E-05
PURA	0.231573	1.81E-05
VWDE	0.478172	1.82E-05
ZNF644	-0.17328	1.82E-05
HSPA13	-0.228	1.83E-05
LRRCC1	0.302535	1.83E-05
SH3BP4	-0.16955	1.84E-05
LMTK2	-0.24185	1.85E-05
USP13	0.183906	1.85E-05
CALM1	-0.17018	1.87E-05
CDK9	-0.22213	1.87E-05
FEM1B	-0.19477	1.88E-05
ARL8B	-0.17607	1.88E-05
SAMD4B	-0.19153	1.88E-05
SOD2	-0.19383	1.90E-05
KIAA1279	-0.19616	1.92E-05
ZNF337	0.404477	1.92E-05
XYLT2	0.336879	1.94E-05
GPR107	-0.17946	1.95E-05
C1orf56	0.631412	1.98E-05
PUF60	-0.19186	1.98E-05
PDGFC	0.535404	2.00E-05
EIF4ENIF1	-0.2339	2.02E-05

RARB	0.703458	2.03E-05
TUBA1B	-0.15989	2.06E-05
PSMB1	-0.15755	2.06E-05
PELI1	0.212224	2.07E-05
PPIL4	-0.26033	2.08E-05
METTL16	-0.229	2.09E-05
TDRP	0.380525	2.10E-05
CDON	0.529496	2.10E-05
DDX24	-0.16593	2.10E-05
RPS19BP1	-0.17392	2.13E-05
DGKG	0.334127	2.14E-05
BROX	-0.20119	2.15E-05
C2orf15	0.4447	2.15E-05
RNF44	-0.21866	2.18E-05
DNAJC7	-0.16095	2.18E-05
APCDD1	0.232803	2.19E-05
KIF3A	0.345335	2.22E-05
BIRC5	-0.21516	2.22E-05
TRIP4	-0.24591	2.26E-05
KIF24	-0.26939	2.27E-05
SACS	0.2961	2.28E-05
EHBP1L1	-0.53728	2.32E-05
FOXD1	0.323177	2.33E-05
DDX27	-0.18274	2.34E-05
ST3GAL2	0.410748	2.35E-05
KCTD18	0.383204	2.36E-05
GNAI3	-0.18348	2.37E-05
C2orf49	-0.23409	2.38E-05
SLFN12	0.678339	2.39E-05
YWHAB	-0.17428	2.43E-05
IBA57	-0.22864	2.45E-05
GPR124	0.758336	2.50E-05
NSRP1	-0.17665	2.50E-05
BRMS1	-0.22956	2.50E-05
IRAK2	0.28113	2.50E-05
PLEKHB2	-0.19642	2.50E-05
GHITM	-0.16318	2.53E-05
CWC22	-0.18192	2.55E-05
PUS1	-0.24315	2.56E-05
RAD54L2	-0.35282	2.57E-05
NCBP2-AS2	-0.27337	2.60E-05
CHMP1B	-0.24884	2.60E-05
CHST3	0.73635	2.61E-05
ERCC6L	0.289421	2.61E-05
OSBPL3	0.274274	2.63E-05
ZNF226	0.321444	2.64E-05
LRRC49	0.269726	2.65E-05

DROSHA	-0.17815	2.65E-05
C19orf44	0.402232	2.66E-05
POLR3A	-0.18415	2.67E-05
DAB2IP	0.364034	2.67E-05
TMBIM6	-0.1734	2.72E-05
ESAM	0.786584	2.72E-05
URB2	-0.24286	2.73E-05
UBA2	-0.16785	2.74E-05
SH3GL1	-0.16559	2.75E-05
RTKN2	0.236604	2.77E-05
UBA1	-0.16098	2.79E-05
BTG3	0.199205	2.79E-05
TWF1	-0.1964	2.79E-05
KIF3C	0.290836	2.84E-05
MAP2K7	-0.18063	2.88E-05
TPD52L2	-0.18529	2.88E-05
ZFP41	0.38363	2.88E-05
ZNF2	-0.3645	2.91E-05
ASH2L	-0.17171	2.94E-05
ZNF891	0.942884	2.96E-05
ELP5	-0.21823	2.97E-05
SLC30A7	-0.34476	2.99E-05
STK35	-0.22266	3.00E-05
DNMT1	-0.14538	3.01E-05
BCAS2	-0.20245	3.01E-05
BFAR	-0.2524	3.01E-05
PHC3	-0.20333	3.01E-05
AQP11	1.262413	3.02E-05
PAK2	-0.15651	3.03E-05
CSNK2A2	-0.24624	3.05E-05
TMEM144	0.836962	3.05E-05
AP2M1	-0.14558	3.05E-05
FBXO32	-0.53739	3.06E-05
PSMB4	-0.15094	3.07E-05
LOC100506	0.870365	3.08E-05
LOC284023	0.988332	3.10E-05
ZNF592	-0.1935	3.11E-05
NLRC5	0.609025	3.11E-05
EMC1	-0.18855	3.12E-05
ALAS1	-0.26355	3.13E-05
KBTBD4	-0.28207	3.13E-05
LHX1	0.36971	3.13E-05
CKMT1A	0.793554	3.14E-05
RAVER2	0.42758	3.18E-05
PRNP	0.225316	3.19E-05
TOMM40	-0.16555	3.19E-05
MAPK13	0.216646	3.20E-05

SNX25	-0.18613	3.20E-05
TIMM50	-0.19246	3.21E-05
SLC25A21-/	1.156064	3.21E-05
MLF2	-0.16097	3.22E-05
IRAK4	0.378024	3.23E-05
CCNC	-0.1829	3.24E-05
CYP1B1	0.344676	3.27E-05
FCHO2	0.351216	3.28E-05
CDC42	-0.17535	3.28E-05
FZD9	0.581953	3.29E-05
SCYL2	-0.18483	3.29E-05
KIAA1377	0.815682	3.31E-05
CS	-0.15524	3.31E-05
TMEM39A	-0.21987	3.31E-05
NFATC1	1.021252	3.31E-05
ATRIP	-0.34738	3.33E-05
C15orf41	0.310579	3.33E-05
MMP15	0.668071	3.35E-05
STK17B	-0.26188	3.35E-05
SCARNA12	-0.93783	3.35E-05
PLEK2	0.641388	3.35E-05
CDC5L	-0.17126	3.37E-05
RNH1	-0.22799	3.40E-05
CCDC122	0.899147	3.41E-05
GAD1	0.407203	3.45E-05
TOP3A	-0.23441	3.45E-05
INO80D	-0.40268	3.46E-05
ZNF821	0.486019	3.46E-05
ATL1	1.006151	3.47E-05
TAPT1	-0.21939	3.48E-05
GRB2	-0.20205	3.48E-05
CLK4	-0.28248	3.48E-05
ASB16-AS1	0.4579	3.49E-05
PIGG	-0.18673	3.51E-05
SLC25A36	-0.16408	3.53E-05
ZNF451	-0.17152	3.57E-05
MX1	0.500453	3.58E-05
PITPNA	-0.22551	3.58E-05
ASCL5	0.953395	3.59E-05
CSNK1G3	0.195887	3.60E-05
LASP1	-0.17447	3.61E-05
ASH1L	-0.18882	3.61E-05
USP43	0.58293	3.63E-05
RAD54L	-0.22514	3.66E-05
NCR3LG1	0.676707	3.67E-05
KIF1A	-0.17862	3.67E-05
MAGI3	0.237298	3.68E-05

GPR19	1.130189	3.68E-05
KLHL13	0.418768	3.70E-05
METTL22	-0.24987	3.70E-05
UMPS	-0.18814	3.70E-05
C3orf52	0.502684	3.71E-05
SALL3	0.287985	3.72E-05
TRAF3IP1	-0.27702	3.77E-05
RARS2	-0.19542	3.77E-05
LSM14B	-0.23781	3.83E-05
USP3	0.192883	3.85E-05
NUP43	-0.20193	3.85E-05
KDM4D	0.459265	3.88E-05
EMC3	-0.25293	3.88E-05
TMEM86B	-0.91807	3.88E-05
CSNK1G2	-0.16471	3.89E-05
BUB1	-0.15987	3.92E-05
ZNF90	0.598429	3.92E-05
CLASRP	-0.23343	3.94E-05
ZNF212	-0.31142	3.94E-05
SEC23B	-0.21175	3.97E-05
ATP6V1D	-0.27368	3.98E-05
PNMA1	0.250355	3.99E-05
SPEN	-0.19147	3.99E-05
CLN5	0.396291	4.01E-05
RBL1	0.193359	4.01E-05
TRAK1	0.302439	4.02E-05
THNSL1	0.436181	4.02E-05
KIF22	-0.17081	4.05E-05
TRNP1	0.366448	4.06E-05
CPSF3L	-0.16765	4.10E-05
PSMB6	-0.18055	4.12E-05
SMG7	-0.16215	4.21E-05
MRPS2	-0.21805	4.21E-05
TRIM37	-0.21138	4.22E-05
7-Mar	-0.15949	4.27E-05
HMGB3	-0.15549	4.29E-05
XBP1	-0.22897	4.30E-05
PAK1	0.163985	4.31E-05
PSMA5	-0.17423	4.34E-05
TET1	0.558495	4.38E-05
HLA-A	0.170292	4.39E-05
USP35	0.519753	4.40E-05
SLC45A2	1.195434	4.41E-05
PAQR7	0.76921	4.41E-05
TAF4	-0.32094	4.44E-05
ZC3H12A	-0.5749	4.53E-05
DDX23	-0.17659	4.59E-05

UBP1	-0.17663	4.61E-05
NOC3L	-0.21241	4.65E-05
SEPHS2	0.189233	4.67E-05
NOB1	-0.16181	4.67E-05
SNORD55	-0.68378	4.67E-05
SDPR	0.176363	4.68E-05
VEPH1	0.263745	4.72E-05
P4HA2	0.228443	4.75E-05
PARG	-0.2114	4.75E-05
RANBP2	-0.20894	4.76E-05
DUSP10	-0.1642	4.81E-05
MCM3AP	-0.18883	4.81E-05
TMEM30A	-0.15731	4.82E-05
FSTL3	0.479553	4.83E-05
MED6	-0.23866	4.84E-05
FUT11	0.418697	4.84E-05
PTPRG-AS1	0.714186	4.84E-05
KLF13	0.219537	4.85E-05
TRIM56	0.494303	4.86E-05
NCBP1	-0.17148	4.88E-05
LOC101929	0.491106	4.90E-05
SNRNP27	-0.20858	4.91E-05
IRF7	-0.42833	4.93E-05
FERMT1	0.26351	4.95E-05
KIAA1467	0.470893	4.96E-05
AOC3	-1.12324	4.97E-05
TRPM4	0.807894	5.00E-05
SLC39A7	-0.15945	5.00E-05
ARMC7	0.284034	5.00E-05
SDC4	0.698669	5.04E-05
SLAIN2	-0.18894	5.06E-05
LINC01249	1.129414	5.08E-05
WWP2	-0.18505	5.11E-05
XRCC6	-0.15109	5.15E-05
KIAA0753	-0.34971	5.17E-05
SNAPC1	0.421539	5.17E-05
EPM2AIP1	0.929046	5.20E-05
PHLPP2	-0.22114	5.22E-05
FOXO1	0.485052	5.22E-05
PVRL3	0.305082	5.22E-05
KAT8	-0.1879	5.25E-05
MBOAT1	0.418099	5.25E-05
EPG5	-0.25998	5.29E-05
B3GALNT1	0.962315	5.29E-05
DERL2	-0.24883	5.29E-05
ZNF780A	0.475652	5.29E-05
P4HB	-0.14867	5.30E-05

TNFRSF1A	-0.31266	5.37E-05
LOC64671S	0.567885	5.38E-05
UBR7	-0.19219	5.47E-05
ZSCAN25	-0.22287	5.47E-05
POP4	-0.20692	5.47E-05
SBK1	0.452193	5.48E-05
TMEM56	0.556223	5.60E-05
PARP8	0.401526	5.60E-05
SMIM13	0.283861	5.63E-05
IRGQ	0.245455	5.63E-05
STYX	-0.2455	5.68E-05
TMEM17	0.752899	5.69E-05
C9orf78	-0.22299	5.71E-05
SRC	0.195418	5.74E-05
PEF1	-0.20518	5.74E-05
NR2C2	-0.26322	5.75E-05
FASLG	1.014723	5.78E-05
CDK11A	-0.4191	5.79E-05
PGGT1B	-0.203	5.80E-05
TTC9	-0.1875	5.83E-05
RPS6KB2	-0.20013	5.83E-05
PPP1CC	-0.15391	5.83E-05
TM2D2	-0.21768	5.87E-05
HGS	-0.20967	5.89E-05
USP51	0.952588	6.06E-05
TSPYL1	-0.2122	6.08E-05
ZNF658	0.774043	6.12E-05
WIBG	-0.25762	6.17E-05
CHL1	0.148386	6.19E-05
F8A1	0.973907	6.20E-05
MLH3	0.385579	6.22E-05
TMEM97	0.184942	6.23E-05
KIAA1191	-0.16717	6.26E-05
GLCCI1	0.190344	6.31E-05
VAPA	-0.17269	6.32E-05
CEP170	0.183087	6.33E-05
TNFSF9	0.441932	6.35E-05
VIL1	0.803539	6.37E-05
MANBA	0.38941	6.38E-05
C11orf95	0.410191	6.41E-05
YAE1D1	0.329928	6.41E-05
ZNF346	0.201429	6.42E-05
CCNB2	-0.15231	6.45E-05
TFB2M	-0.24429	6.45E-05
LPL	0.263446	6.55E-05
ZNF445	0.398609	6.63E-05
GAS5	-0.15995	6.64E-05

HES4	0.730795	6.65E-05
NACAD	0.626126	6.75E-05
POMGNT2	0.364797	6.78E-05
UBE3C	-0.16976	6.80E-05
CHN2	0.650772	6.81E-05
CNOT6	-0.17736	6.84E-05
CTSC	0.182953	6.84E-05
U2AF1	-0.21714	6.85E-05
PTGFRN	0.228015	6.87E-05
GAB1	0.595072	6.87E-05
ST3GAL4-A	0.659599	6.89E-05
RBAK	-0.20267	6.89E-05
NDOR1	-0.26752	6.92E-05
ZNF662	1.030351	6.95E-05
PRELID2	0.482583	7.00E-05
PTX3	0.202335	7.07E-05
WASL	-0.24654	7.09E-05
C12orf60	0.731721	7.10E-05
LSR	-0.19633	7.20E-05
ZFP1	0.266566	7.23E-05
DPH2	-0.14587	7.23E-05
LARP4	-0.17966	7.23E-05
MESDC1	0.242866	7.23E-05
POLR3GL	0.398716	7.24E-05
ATAD2B	-0.17485	7.25E-05
ARFGAP2	-0.17198	7.25E-05
TMEM171	0.883152	7.29E-05
KPNB1	-0.14723	7.32E-05
HR	0.260859	7.32E-05
FBXO34	0.226557	7.42E-05
ARHGEF3	0.248551	7.46E-05
HERC2P7	0.505263	7.50E-05
SLC22A17	0.476106	7.53E-05
SLC45A1	0.946369	7.58E-05
MAPRE2	0.178511	7.65E-05
ZNF37A	0.24648	7.66E-05
RP9P	0.306178	7.73E-05
WASF1	0.246799	7.77E-05
PCDHGC3	0.45863	7.82E-05
PSMD6	-0.16464	7.82E-05
MRPS23	-0.20869	7.90E-05
NSD1	0.155887	7.94E-05
PNPLA3	0.556648	8.04E-05
ETV6	0.549582	8.10E-05
MDN1	-0.21127	8.14E-05
SUGP1	-0.24493	8.23E-05
LRRFIP2	-0.22932	8.32E-05

DCP1B	0.473024	8.35E-05
TAX1BP1	-0.20723	8.36E-05
SLC9A3R1	-0.28194	8.37E-05
HSPH1	-0.15209	8.41E-05
STX7	0.34697	8.41E-05
YPEL5	-0.33778	8.45E-05
TRIM44	0.205716	8.46E-05
AGPAT4	0.279909	8.47E-05
CKAP5	-0.15305	8.50E-05
ZC3H4	-0.19989	8.53E-05
LINC01184	0.479574	8.57E-05
CAPRIN1	-0.13612	8.58E-05
MANEA	0.203755	8.60E-05
FSCN1	-0.18402	8.62E-05
TMSB15A	1.110772	8.63E-05
ZNF641	0.437174	8.66E-05
WDR3	-0.14199	8.72E-05
PLSCR1	0.452275	8.76E-05
BCL9L	0.651911	8.81E-05
2-Sep	-0.15416	8.86E-05
A4GALT	0.448545	8.87E-05
SMARCC1	-0.16621	8.90E-05
PSMB5	-0.15695	9.09E-05
NCAPH2	-0.2081	9.11E-05
ZMAT1	0.922998	9.12E-05
FABP5	0.177209	9.13E-05
WDR36	-0.17355	9.19E-05
TSC2	-0.22558	9.19E-05
EIF4B	-0.16308	9.25E-05
LAMP3	0.556614	9.33E-05
MPP5	0.187474	9.34E-05
ASPH	0.158681	9.35E-05
SLC25A4	0.166666	9.39E-05
NUPL2	-0.2289	9.40E-05
TRAPPC12	-0.18913	9.42E-05
LIX1L	0.297186	9.42E-05
PKD2	0.284381	9.48E-05
PRKAR1A	-0.16448	9.49E-05
C22orf29	0.326073	9.50E-05
LMBRD1	0.262804	9.51E-05
EXOSC6	-0.19954	9.55E-05
ZBED6	-0.64901	9.56E-05
LPIN2	0.316775	9.61E-05
NUP62	-0.17449	9.67E-05
VPS37A	-0.19079	9.71E-05
PTCD3	-0.16289	9.80E-05
GTPBP1	-0.18214	9.85E-05

RBM33	-0.21229	9.92E-05
SAR1B	-0.17887	9.94E-05
MRPS30	-0.1933	9.98E-05
